# Synthesis of 4-thiouridines with prodrug functionalization for RNA metabolic labeling†

**DOI:** 10.1039/d2cb00001f

**Published:** 2022-02-25

**Authors:** Sarah Moreno, Melanie Brunner, Isabel Delazer, Dietmar Rieder, Alexandra Lusser, Ronald Micura

**Affiliations:** Institute of Organic Chemistry, Center for Molecular Biosciences Innsbruck, University of Innsbruck Innrain 80-82 6020 Innsbruck Austria ronald.micura@uibk.ac.at; Institute of Molecular Biology, Biocenter, Medical University of Innsbruck Innrain 80-82 6020 Innsbruck Austria alexandra.lusser@i-med.ac.at; Institute of Bioinformatics, Biocenter, Medical University of Innsbruck Innrain 82 6020 Innsbruck Austria

## Abstract

Metabolic labeling has emerged as a powerful tool to endow RNA with reactive handles allowing for subsequent chemical derivatization and processing. Recently, thiolated nucleosides, such as 4-thiouridine (4sU), have attracted great interest in metabolic labeling-based RNA sequencing approaches (TUC-seq, SLAM-seq, TimeLapse-seq) to study cellular RNA expression and decay dynamics. For these and other applications (*e.g.* PAR-CLIP), thus far only the naked nucleoside 4sU has been applied. Here we examined the concept of derivatizing 4sU into a 5′-monophosphate prodrug that would allow for cell permeation and potentially improve labeling efficiency by bypassing the rate-limiting first step of 5′ phosphorylation of the nucleoside into the ultimately bioactive 4sU triphosphate (4sUTP). To this end, we developed robust synthetic routes towards diverse 4sU monophosphate prodrugs. Using metabolic labeling assays, we found that most of the newly introduced 4sU prodrugs were well tolerated by the cells. One derivative, the bis(4-acetyloxybenzyl) 5′-monophosphate of 4sU, was also efficiently incorporated into nascent RNA.

## Abstract 2

Synthetic access to 4-thiouridine (4sU) derivatives with monophosphate prodrug patterns creates additional possibilities for metabolic labeling of RNA for different applications.

## Graphical abstract

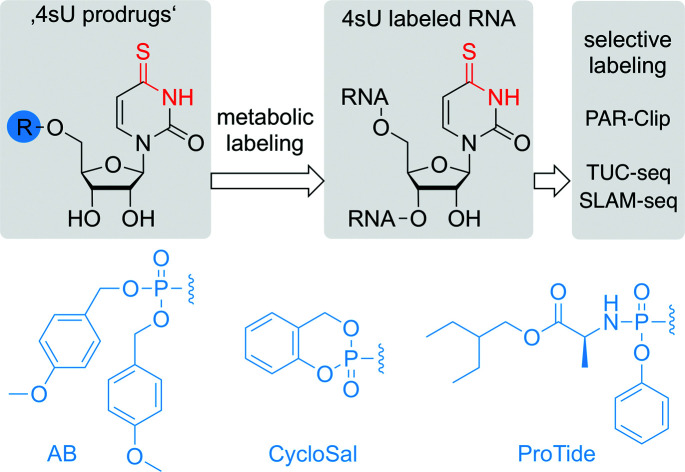

## Introduction

Metabolic labeling has emerged as a powerful tool to endow RNA with reactive handles *in vivo* allowing for subsequent chemical derivatization for a broad range of applications.^1–6^ Thereby, alkyne-, vinyl-, or azide-modified nucleosides are taken up as cell-permeable metabolic precursors that can directly enter the nucleotide salvage pathway for conversion to nucleotide triphosphates (NTPs).^7–9^ After incorporation into different types of RNA during transcription or during polyadenylation, click chemistry is used as the labeling of choice.^10–14^ Moreover, RNA labeled with 4sU has been widely used to capture RNA-binding proteins, due to the intrinsic propensity of 4sU to form covalent links with interacting RNA binding proteins upon UV 365 nm irradiation.^15–17^ Recently, thiolated nucleosides, in particular 4-thiouridine (4sU), have attracted much interest in metabolic labeling-based RNA sequencing approaches (TUC-seq, SLAM-seq, TimeLapse-seq) to study cellular RNA dynamics, such as synthesis and degradation rates.^18–23^ 4sU uptake into cells is mediated by two distinct families of nucleoside transporters.^24^ While cell lines, such as HEK293 or HeLa cells, show robust expression of the major uridine transporters SLC29A1 and SLC29A2, and therefore readily take up 4sU, many other cell types and cell lines do not (*e.g.* ref. 25; see also The Human Protein Atlas^26^ for transporter expression). To broaden the applicability of 4sU for RNA expression dynamics or PAR-CLIP analyses in such cell types, the use of 4sU monophosphate prodrugs may be a promising approach,^27–31^ superior to uptake of the naked nucleoside.^32–41^ Although the exact mechanisms by which nucleoside monophosphate prodrugs enter the cell are not well understood,^42^ it is often implied that passive absorption is involved,^43^ or that they interact with other cellular drug transporters.^44,45^

**Fig. 1:**
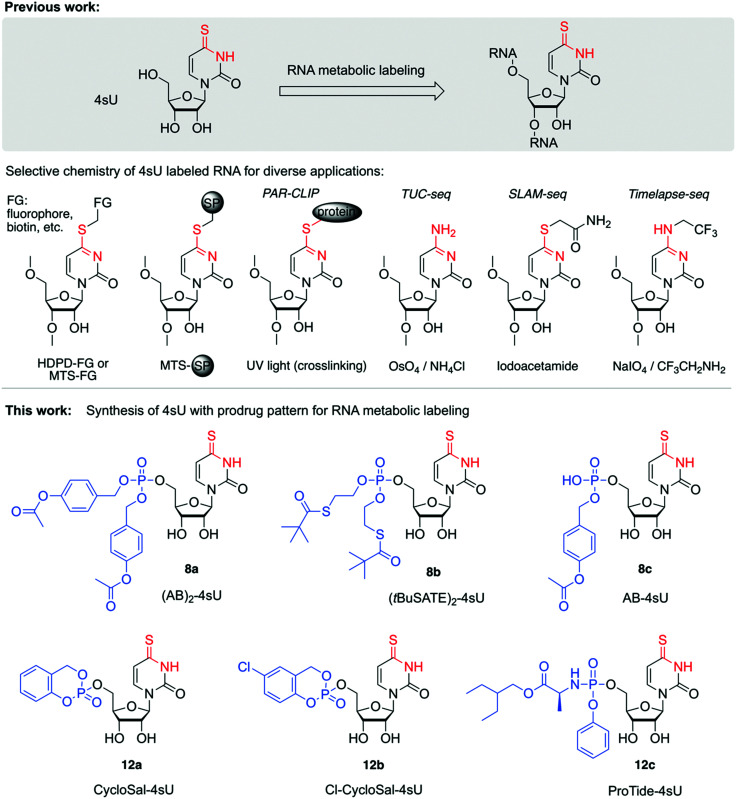
Background and overview of the work described. Upper panel: 4sU is applied for metabolic labeling of RNA to enable thiol-selective chemistry for RNA derivatization. Applications range from covalent labeling with prosthetic groups, fluorophores, biotin for RNA pulldown and enrichment to PAR-CLIP (PhotoActivatable Ribonucleoside-enhanced CrossLinking and ImmunoPrecipitation) to novel RNA sequencing approaches that utilize 4sU derivatization or conversion for defined mutational signatures that shed light on cellular RNA dynamics. Typical reagents are: *N*-[6-(biotinamido)hexyl]-3′-(2′-pyridyldithio) propionamide (biotin-HPDP) for streptavidin pulldown or thiosulfonate (MTS) modifed magnetic beads (SP) for one-step enrichment procedure captures 4sU-RNA (see *e.g.* ref. 69). Additionally, this reagent type is widely used for fluorescent labeling of 4sU containing tRNAs (see *e.g.* ref. 70). The RNA sequencing approaches TUC-seq, SLAM-seq and TimeLapse-seq rely on chemical 4sU transformations using distinct reagents, thereby converting 4sU into cytosine (TUC-seq; osmium tetroxide (OsO_4_) and ammonia), alkylated uridine (SLAM-seq; iodoacetamide) or trifluoroethylated cytidine (TimeLapse; 2,2,2-trifluorethylamine (TFEA) in combination with meta-chloroperoxybenzoic acid (mCPBA) or sodium periodate (NaIO_4_)). Lower panel: Structures of novel 4sU derivatives with monophosphate prodrug pattern that have been synthesized in this study and tested exemplarily in metabolic labeling experiments on HEK293T cells with readout by TUC-seq.

In addition, delivery of 4sU 5′-monophosphate derivatives into the cell allows to circumvent the rate-limiting step of nucleoside monophosphorylation, thus enabling late-stage entry into the nucleotide salvage pathway. Therefore, processing time towards the bioactive di- and triphosphates (4sUDP and 4sUTP) can be shortened which may increase time resolution of RNA labeling experiments.^2,10^ Interestingly, despite the importance and wide-spread use of 4sU metabolic labeling (Fig. 1), 4sU prodrugs or prodrug-like derivatives have not yet been tested, and to the best of our knowledge, the chemical synthesis of 4sU prodrugs has not yet been reported either. Therefore, we set out to create the synthetic foundation for 4sU monophosphate prodrugs and to test their performance in metabolic labeling experiments.

## Results and discussion

### Design of 4sU prodrugs

Most prodrug strategies for nucleoside analogs rely on 5′-*O*-phosphate derivatives. The rationale is to shortcut the stepwise conversion path into the biologically active nucleoside triphosphate because the kinases involved are rather ineffective for the modified nucleoside due to high specificity for their native substrates.^27^ In particular the first phosphorylation is often rate limiting.^2,10^ However, due to the increase in polarity inhibiting penetration of cellular membranes, the use of free nucleoside monophosphates for metabolic labeling is not possible. To circumvent this problem the negatively charged oxygen atoms of the 5′-*O*-monophosphate group are masked with 'biolabile' protecting groups which results in membrane-permeable compounds.

With respect to 4sU, we focused on established prodrug patterns, including 4-acetyloxybenzyl (AB),^29,46^*S*-pivaloyl-2-thioethyl (*t*BuSATE),^47^*S*-acetylthioethyl (SATE),^48^ and cycloSaligenyl (CycloSal) phosphates,^31^ as well as phosphoramidates consisting of an amino acid ester promoiety linked *via* P–N bond to the nucleoside aryl phosphate (ProTide).^28,49^ Depending on the prodrug pattern, different cellular pathways to release the free 5′-monophosphate nucleotide have been described.^50^ Additionally, we considered 4sU 5′-*O*-monophosphates that carry a biolabile moiety at the *S*^4^ atom as potential prodrug candidates and set out for their chemical synthesis.

### Synthesis of 4sU prodrugs

Starting from uridine, we synthesized 4-thiouridine (4sU) in three steps,^51,52^*via* acetylation of the hydroxyl groups (compound 1), transformation of the 4-oxo into the 4-thio group using the Lawesson reagent (compound 2), followed by deacetylation with ammonia in methanol to obtain the free 4sU nucleoside 3 in high yields (Scheme S1, ESI†).

**Scheme 1:**
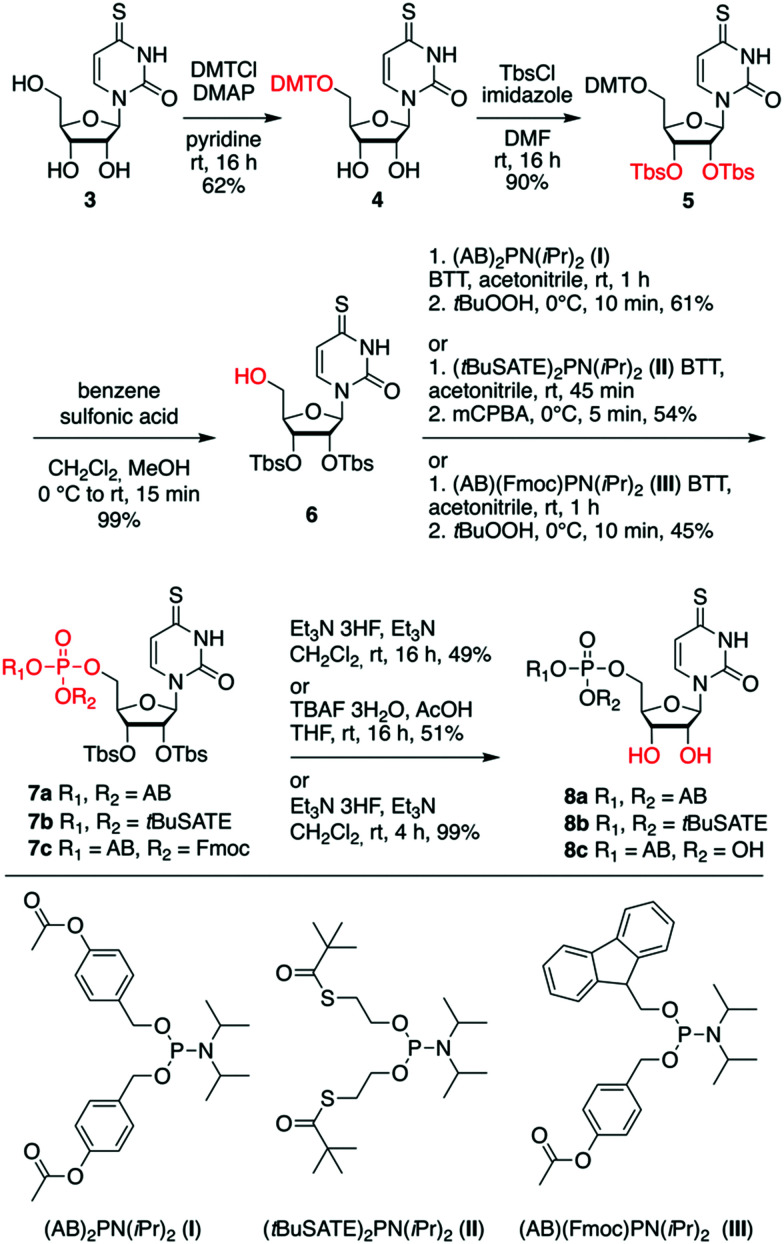
Synthesis of AB and *t*BuSATE 4sU monophosphate prodrugs 8a–8c. Abbreviations: 4,4′-dimethoxytrityl chloride (DMTCl), 4-(*N*,*N*-dimethylamino)pyridine (DMAP), *tert*-butyldimethylsilyl chloride (TbsCl), acetyloxybenzyl (AB), 5-benzylthio-1*H*-tetrazole (BTT), *meta*-chloroperoxybenzoic acid (*m*CPBA), *S*-acetylthioethyl (SATE), *S*-pivaloyl-2-thioethyl (*t*BuSATE), 9-fluorenylmethoxycarbonyl chloride (Fmoc), tetra-*n*-butylammonium fluoride (TBAF).

Targeting the first set of 4sU prodrugs, namely the 4-acetyloxybenzyl (AB)- and *S*-pivaloyl-2-thioethyl (*t*BuSATE) 5′-*O*-monophosphates 8a–c (Scheme 1), we protected the 5′-OH group of 4sU as 4,4′-dimethoxytrityl (DMT) ether using 4,4′-dimethoxytrityl chloride and pyridine to give compound 4. Then, the protection of 2′ and 3′-OH groups was accomplished by reaction with *tert*-butyldimethylsilyl chloride in the presence of imidazole to yield nucleoside 5. Removal of the 5′-*O*-DMT moiety under acidic conditions afforded the crucial precursor 6 for subsequent introduction of the phosphotriester moiety. This step was performed using mild phosphor(iii)amidite chemistry catalyzed by 5-(benzylthio)-1*H*-tetrazole. The required reagents bis-(4-acetyloxybenzyl) *N*,*N*-diisopropylaminophosphoramidite I,^29,53^ bis-(*S*-pivaloyl-2-thioethyl) *N*,*N*-diisopropylaminophosphoramidite II,^53,54^ and 4-[({[bis(propan-2-yl)amino](9*H*-fluoren-9-ylmethoxy)phosphanyl}oxy)methyl]phenyl acetate III,^55,56^ were synthesized as described in the ESI,† largely following known procedures that we optimized further. Importantly, selective oxidation of the phosphite P(iii) triester intermediates to the required phosphate triester P(v) species was accomplished using equimolar amounts of *tert*-butyl hydroperoxide (for 7a and 7c) or 3-chlorperbenzoic acid (for 7b) at low temperature and short reaction times. Potential oxidation of the sulfur moiety was not observed under these conditions. Finally, the 2′-*O* and 3′-*O* silyl ethers were cleaved by treatment with triethylamine trihydrofluoride to obtain prodrugs 8a and 8c, or by tetrabutylammonium fluoride trihydrate in tetrahydrofuran for prodrug 8b.

**Scheme 2:**
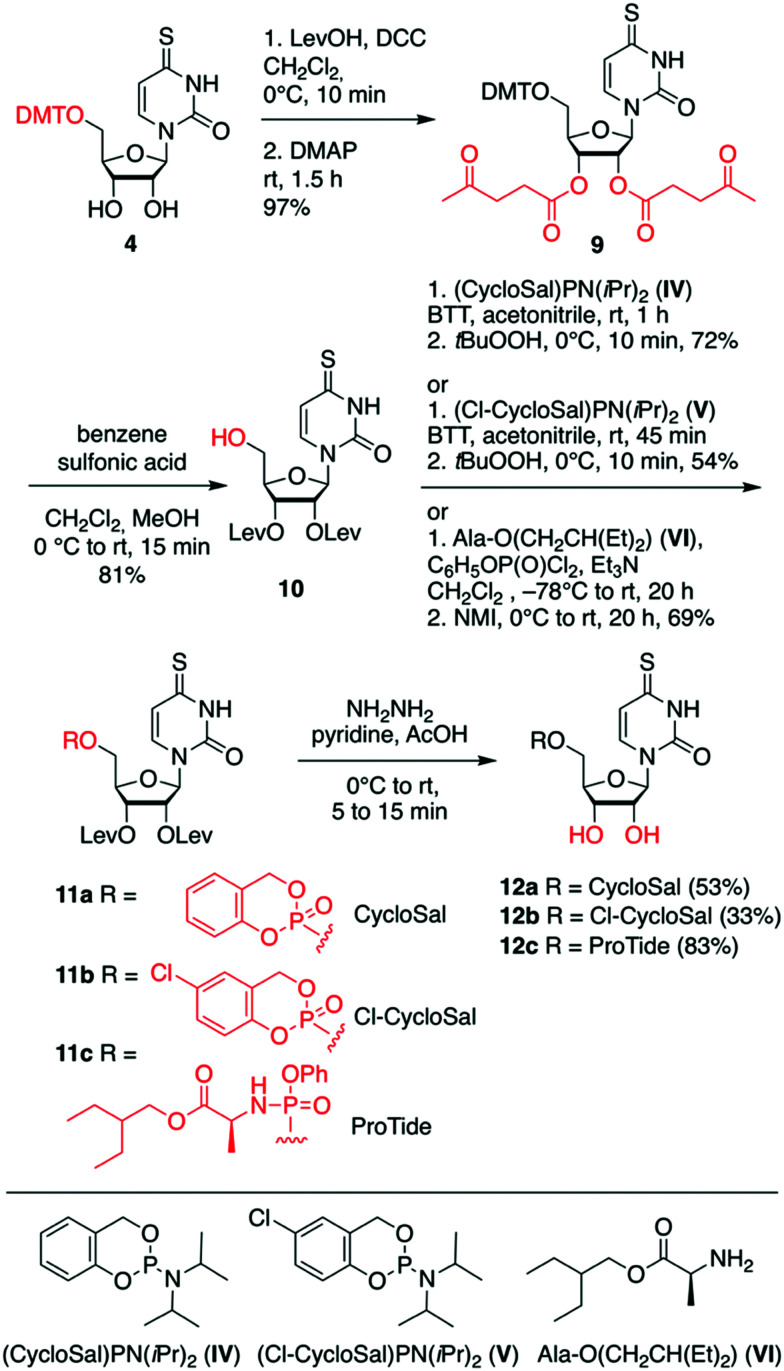
Synthesis of CycloSal, Cl-CycloSal and ProTide 4sU monophosphate prodrugs 12a–c. Abbreviations: levulinic acid (LevOH), *N*,*N*'-dicyclohexylcarbodiimide (DCC), 4-(*N*,*N*-dimethylamino)pyridine (DMAP), 5-benzylthio-1*H*-tetrazole (BTT), *N*-methylimidazole (NMI).

For the second set of 4sU prodrugs (Scheme 2), namely the saligenyl (CycloSal, Cl-CycloSal) and phosphoramidate (ProTide) compounds 12a–c, we started with the 5′-*O*-tritylated nucleoside **4** and protected the 2′ and 3′-OH groups as levulinyl esters to yield compound 9. Detritylation gave nucleoside 10 which was reacted with either saligenyl-*N*,*N*-diisopropylaminophosphoramidite **IV**^57–60^ or 5-chlorosaligenyl-*N*,*N*-diisopropylaminophosphoramidite **V**;^58,59,61,62^ subsequently, oxidation using one equivalent of *tert*-butyl hydroperoxide gave nucleoside 11a and 11b, respectively. The desired prodrugs 12a and 12b were finally obtained using fast treatment with aqueous hydrazine in pyridine and acetic acid and immediate workup. The levulinyl esters were readily cleaved under these conditions leaving the sensitive saligenyl- and chlorosaligenyl moieties intact. To obtain the 4sU ProTide compound 12c (Scheme 2), the required phosphorylation reagent was prepared *in situ* by mixing phenyl dichlorophosphate and 2-ethylbutyl (*S*)-2-aminopropanoate hydrochloride **VI**,^63^ activated with triethylamine. The resulting phosphoryl chloride was reacted with precursor **10** to furnish nucleotide 11c, which was further transformed into the desired prodrug 12c; both steps were high yielding.

**Scheme 3:**
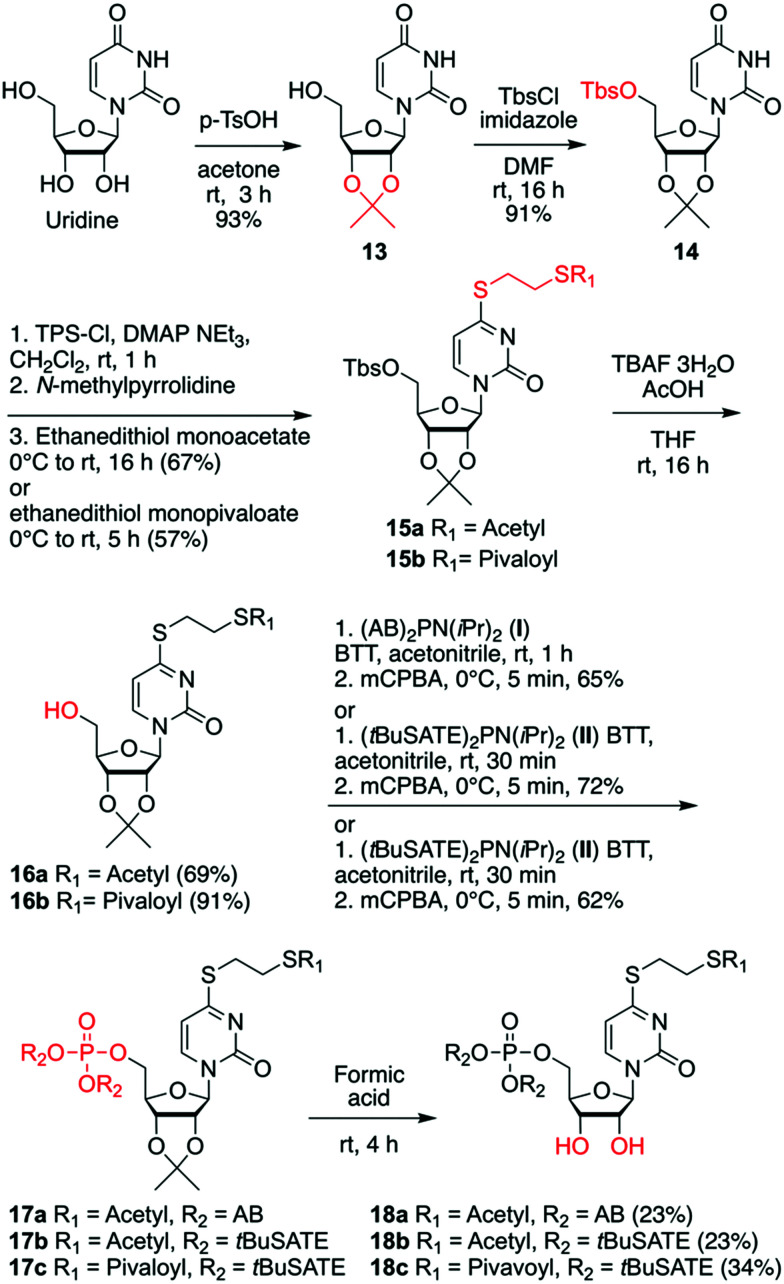
Synthesis of AB and *t*BuSATE 4sU monophosphate prodrugs **18a–c** with biolabile thio protecting group. Abbreviations: *p*-toluenesulfonic acid (*p*-TsOH), *tert*-butyldimethylsilyl chloride (TbsCl), 4-(*N*,*N*-dimethylamino)pyridine (DMAP), 2,4,6-triisopropylbenzenesulfonyl chloride (TPSCl), tetra-*n*-butylammonium fluoride (TBAF), acetyloxybenzyl (AB), 5-benzylthio-1*H*-tetrazole (BTT), *S*-acetylthioethyl (SATE), *S*-pivaloyl-2-thioethyl (*t*BuSATE), meta-chloroperoxybenzoic acid (mCPBA).

For the third subset of 4sU prodrugs (Scheme 3), namely *S*^4^-(*S*-pivaloyl-2-thioethyl)- or *S*^4^-(*S*-acetylthioethyl) functionalized AB and *t*BuSATE prodrugs 18a–c, the synthesis started with simultaneous protection of the 2′ and 3′-OH groups as isopropylidene acetal to yield nucleoside **13**, followed by 5′-OH protection as Tbs ether to give derivative **14**. Subsequently, the *O*^4^ functionality was derivatized as sulfonium ester which was then substituted by either ethanedithiol monoacetate or its pivaloyl analog. The resulting compounds 15a and 15b were then unmasked to provide precursors 16a and 16b. Functionalization of the free 5′-OH group was performed using bis-(4-acetyloxybenzyl) *N*,*N*-diisopropylaminophosphoramidite **I**^29,53^ to obtain intermediate 17a or bis-(*S*-pivaloyl-2-thioethyl) *N*,*N*-diisopropylaminophosphoramidite **II**^53,54^ to obtain intermediates 17b and 17c after selective oxidation. Finally, cleavage of the 2′-*O*, 3′-*O* cyclic acetal with formic acid provided the desired prodrugs 18a–c.

### *In vivo* evaluation of 4sU monophosphate prodrugs

**Fig. 2:**
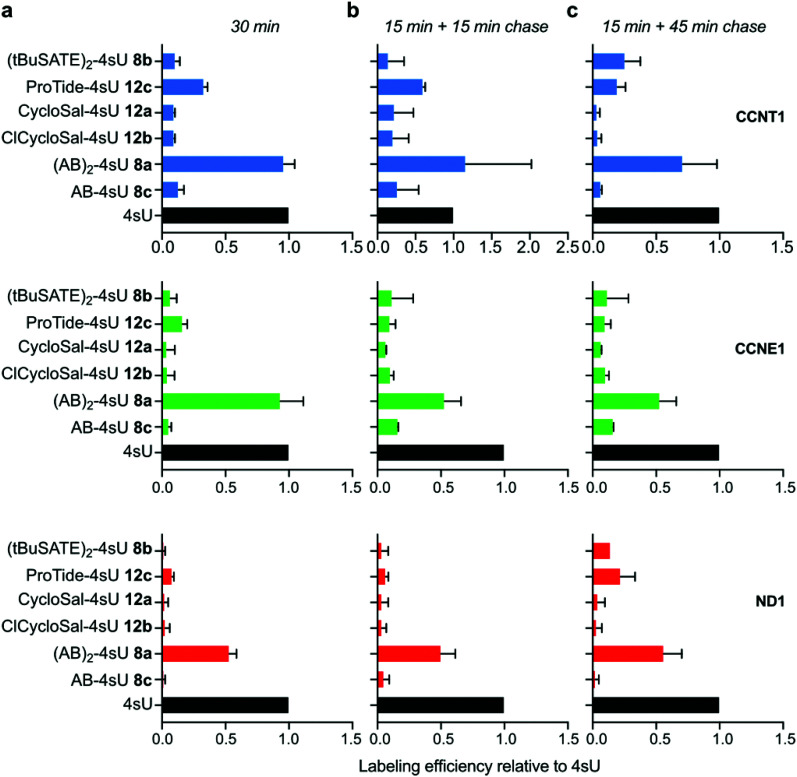
Comparison of mRNA labeling efficiencies using different 4sU prodrugs. HEK293T cells were labeled with 50 μM of the indicated 4sU prodrugs for 30 min (a) or 15 min with a subsequent chase period of either 15 min (b) or 45 min (c) followed by RNA extraction. RNA was treated by TUC-seq chemistry and subjected to amplicon sequencing of the nuclear targets *CCNT1* and *CCNE1* and the mitochondrial transcript *ND1*. Labeling efficiency was calculated relative to 4sU. Mean ± SD is shown (*N* = 2).

To examine the *in vivo* applicability of the synthesized prodrugs, we performed metabolic labeling experiments with HEK293T cells. In a first step, we tested the incorporation efficiency into nascent RNA. To this end, we employed our recently developed TUC-seq method that allows for detection of labeled RNA by chemical conversion of 4sU to cytosine using OsO_4_-mediated chemistry.^20^ Specific target mRNAs were amplified by PCR and subjected to amplicon sequencing to identify labeled transcripts.^23^ We first used a 30 min labeling regime with each prodrug at a concentration of 50 μM and measured incorporation into the two nuclear encoded mRNAs cyclin T1 (CCNT1) and cyclin E1 (CCNE1) as well as the mitochondrially transcribed NADH:ubiquinone oxidoreductase core subunit 1 (ND1) mRNA. For comparison, cells were labeled with 4sU. Of all tested prodrugs, (AB)_2_-4sU 8a performed best with an incorporation efficiency comparable to 4sU (Fig. 2a and Fig. S1, ESI†), while the other reagents (8b,c and 12a,b,**c**) exhibited clearly lower labeling efficiency. For the high turn-over mRNA CCNT1 (ref. 24 and Fig. S1, ESI†), 4sU incorporation was also detected with the ProTide-4sU 12c, albeit at only ∼30% of 4sU. We then shortened the labeling time to 15 min and extracted RNA either 15 or 45 min after replacing the labeling medium with normal medium. The outcome was comparable to the 30 min labeling approach, except that the longer chase period led to increased incorporation of ProTide-4sU 12c as well as (*t*BuSATE)_2_-4sU 8b in all three mRNA targets (Fig. 2b, c and Fig. S1, ESI†). These results suggest that the bioavailability of 4sU released from those prodrugs may be delayed compared to (AB)_2_-4sU 8a. Thus, (AB)_2_-4sU 8a appears to be the most efficient among all tested prodrugs, reaching almost the same levels of incorporation as 4sU.

**Fig. 3:**
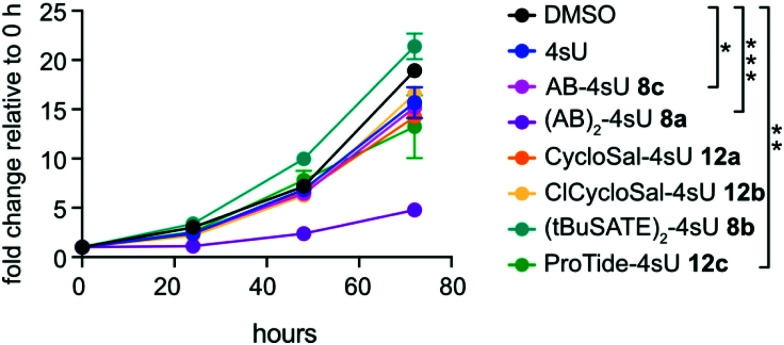
HEK293T cells were labeled with 50 μM of the indicated prodrugs for 30 min followed by incubation in normal media. Cell viability was determined by ATP measurement at 0, 24, 48 and 72 h. Values are expressed as fold-change relative to the 0 h time point for each drug. Mean values ± SD are shown (*N* = 3). Statistical significance between endpoints was calculated by one-way-ANOVA (ns, not significant; *p ≤ 0.05, **p ≤ 0.001, ***p ≤ 0.0001).

To determine cytotoxicity of the diverse 4sU prodrugs, we incubated HEK293T cells at a concentration of 50 μM for 30 min and monitored cell viability over a period of 72 h using an ATP-based assay. Interestingly, the two drugs that exhibited the best 4sU incorporation efficiency ((AB)_2_-4sU 8a, ProTide-4sU 12c; Fig. 2) had the most adverse impact on cell viability, while the prodrugs with low labeling capacity (8b,c and 12a,b) were tolerated well (Fig. 3). We also examined the effects of the *S*^4^-protected 4sU prodrugs (Scheme S2, ESI†) on cell viability. Both AB- as well as *t*BuSATE-derivatized prodrugs (18a–c) showed strong cytotoxicity after 30 min incubation (Fig. S2, ESI†) and therefore underwent no further labeling tests.

Generally, we suspect that certain metabolites released from the prodrugs – *e.g. p*-quinone methide in case of (AB)_2_-4sU 8a – cause the observed adverse effects.^64,65^ AB-4sU 8c which, in contrast to (AB)_2_-4sU 8a, retains negative charge on the phosphate moiety, has a moderate effect on cell viability but at the same time does not lead to 4sU incorporation suggesting that it most likely does not permeate the cell membrane efficiently. For CycloSal (12a) ClCycloSal (12b), and (*t*BuSATE)_2_ (8b) derivatives, the labeling and viability results indicate that 4sU is either very slowly released in the cell or that the compounds do not enter the cells. Studies with CycloSal derivatives of d4T monophosphate rather point towards the former explanation since cycloSal-d4T compounds proved to be stable in RPMI cell culture medium containing 10% fetal calf serum with halflives of *t*_1/2_ = 1.4 to 10.8 hours.^66,67^ Likewise, *t*BuSATE-derivatives of 3′-azido-2′,3′-dideoxythymidine (AZT) had high stability in buffer,^58^ suggesting that the uptake of intact CycloSal (12a,b) and *t*BuSATE 4sU (8b) prodrugs should be possible. By contrast, it was shown that the last step of hydrolysis of *t*BuSATE AZT by cellular enzymes proved to be rate limiting and was in the order of *t*_1/2_ = 3.9 to 25 h.^68^

## Conclusions

In summary, we developed robust synthetic routes towards 5′-*O*-phosphate 4-thiouridines with diverse prodrug masking groups by mastering protection concepts and reaction conditions that allowed for P(iii)-to-P(v) conversions under preservation of the oxidation-sensitive 4-thio moiety. In our set of metabolic labeling experiments limited to HEK293T cells, only the bis(4-acetyloxybenzyl) 5′-monophosphate derivative of 4sU (8a) showed incorporation efficiencies comparable to 4sU, albeit at cost of increased cytotoxicity. Hence, while the monophosphate prodrug derivatives studied here contribute no immediate advantage to metabolic labeling of cells that are amenable to labeling with the 4sU nucleoside, some of the novel prodrugs might be beneficial for the labeling of cells with low expression of the major nucleoside transporters. In those cases, labeling times will likely have to be extended to allow for efficient cell entry and subsequent release of the active compound.

## Experimental

### Synthesis of 4sU prodrugs

Synthetic procedures and analysis data for the synthesis of compounds 1 to 18 and reagents I to VI are described in the ESI.† All compounds synthesized are >95% pure by NMR analysis, and all NMR spectra are depicted in the ESI.†

### Metabolic labeling

1.5 × 10^6^ HEK293T cells were seeded into 3 cm round cell culture dishes and grown for six hours at 37 °C and 5% CO_2_ in culture medium (DMEM/Ham's F-12 + 10% fetal calf serum and GlutaMAX; PAN Bioteck, Gibco). Medium was replaced with culture medium supplemented with 50 μM 4-thiouridine (4sU; Jena Bioscience), 50 μM of the different synthesized prodrugs or vehicle (DMSO). Cells were incubated for 30 min and subsequently harvested. For pulse-chase experiments, labeling medium was replaced with culture medium after 15 min incubation, and cells were further incubated for 15 or 45 min before collection.

### TUC treatment

Total RNA was isolated from labeled and DMSO-treated cells using the innuPREP RNA Mini Kit 2.0 (Analytik Jena) according to the manufacturer's instructions. RNA was incubated with 0.45 mM OsO_4_ and 180 mM NH_4_Cl for 1 h at 40 °C. The reaction was stopped by precipitation with 6 volumes of ice cold ethanol and 2 volumes precipitation buffer (185 mM NaOAc pH 5.2 and 0.25 mg ml^−1^ glycogen).^20^

### Amplicon sequencing and data analysis

TUC-treated RNA was reverse transcribed with GoScript Reverse Transcriptase (Promega) with random hexamer primers according to the manufacturer's instructions. Amplicons of CCNT1 (296 bp), CCNE1 (417 bp) and ND1 (353 bp) were generated by PCR with barcoded primers, purified from 1% agarose gels and pooled at equimolar ratio (primers are listed in Table S1, ESI†). Library preparation from the amplicon pool and sequencing using the Illumina HighSeq platform was performed by Eurofins (Ebersberg, Germany). Processing of raw reads, determination of U-to-C conversion frequency and calculation of labeling efficiency was performed exactly as described,^20,24^ with the addition that we filtered all reads that had >1 unspecific T conversion (T → A|G).

### Viability assay

HEK293T cells were labeled for 30 min as described above followed by replacing the labeling medium with culture medium. Cells were further cultivated for 72 h. Viability was measured at 0, 24, 48 and 72 h using the CellTiter-Glo 2.0 Assay (Promega) according to the manufacturer's instructions. Measurements were performed with technical triplicates and three independent experiments were carried out. All values were expressed as fold change over the 0 h timepoint for each labeling reagent. Statistical analysis was performed by one-way ANOVA using Graphpad Prism 9.

## Conflicts of interest

There are no conflicts to declare.

## Supplementary Material

CB-003-D2CB00001F-s001
