# Supplementary material for: Synthesis of 4-thiouridines with prodrug functionalization for RNA metabolic labeling
Source: RSC Chem Biol. 2022 Feb 25;3(4):447–55. doi: 10.1039/d2cb00001f (PMC8985182; doi:10.1039/d2cb00001f)
Supplement: CB-003-D2CB00001F-s001 [file CB-003-D2CB00001F-s001.pdf]

Electronic Supplementary Material (ESI) for RSC Chemical Biology.
This journal is © The Royal Society of Chemistry 2022

**Supporting Information**

# Synthesis of 4-thiouridines with prodrug functionalization for RNA metabolic labeling

*Sarah Moreno,[†] Melanie Brunner,[‡] Isabel Delazer,[‡] Dietmar Rieder,[§]*
*Alexandra Lusser,*[,‡] Ronald Micura*[,†]*

***Contents***

**1. Chemical synthesis** **1**

**2. Synthesis of 4-thiouridine (4sU, 3)** **2**
2.1. Synthesis of 2´,3´,5´-*O*-triacetyluridine (**1**) 3
2.2. Synthesis of 2´,3´,5´-*O*-triacetyl-4-thiouridine (**2**) 5
2.3. Synthesis of 4-thiouridine (**3**) 7

**3. Synthesis of 4-acyloxybenzyl (AB)- and *S*-pivaloyl-2-thioethyl (*t*BuSATE) 4sU 5'-*O*-monophosphates (8a-c)** **9**
3.1. Synthesis of 5´-*O*-(4,4´-dimethoxytrityl)-4-thiouridine (**4**) 10
3.2. Synthesis of 2´,3´-di-*O*-(*tert*-butyldimethylsilyl)-5´-*O*-(4,4´-dimethoxytrityl)-4-thiouridine (**5**) 12
3.3. Synthesis of 2´,3´-di-*O*-(*tert*-butyldimethylsilyl)-4-thiouridine (**6**) 14
3.4. Synthesis of bis(4-acetoxybenzyl)-5´-*O*-(2´,3´-di-*O*-(*tert*-butyldimethylsilyl)-4-thiouridinyl) phosphate (**7a**) 16
3.5. Synthesis of bis(*S*-pivaloyl-2-thioethyl)-5´-*O*-(2´,3´-di-*O*-(*tert*-butyldimethylsilyl)-4-thiouridinyl) phosphate (**7b**) 19
3.6. Synthesis of (4-acetoxybenzyl)-((9*H*-fluoren-9-yl)methyl)-5´-*O*-(2´,3´-di-*O*-(*tert*-butyldimethylsilyl)-4-thiouridinyl) phosphate (**7c**) 22
3.7. Synthesis of bis(4-acetoxybenzyl)-5´-*O*-(4-thiouridinyl) phosphate (**(AB)$_2$-4sU, 8a**) 25

3.8. Synthesis of bis(*S*-pivaloyl-2-thioethyl)-5´-*O*-(4-thiouridinyl) phosphate ((***t*****BuSATE)$_2$-4sU, 8b**) 28
3.9. Synthesis of (4-acetoxybenzyl)-5´-*O*-(4-thiouridinyl) phosphate triethylammonium salt (**AB-4sU, 8c**) 31

**4. Synthesis of CycloSal-4sU, Cl-CycloSal-4sU and ProTide-4sU (12a-c) 34**
4.1. Synthesis of 2´,3´-di-*O*-levulinoyl-5´-*O*-(4,4´-dimethoxytrityl)-4-thiouridine (**9**) 35
4.2. Synthesis of 2´,3´-di-*O*-levulinoyl-4-thiouridine **(10)** 37
4.3. Synthesis of cyclosaligenyl-5´-*O*-(2´,3´-di-*O*-levulinoyl-4-thiouridinyl) phosphate **(11a)** 39
4.4. Synthesis of cyclo(5-chlorosaligenyl)-5´-*O*-(2´,3´-di-*O*-levulinoyl-4-thiouridineyl) phosphate **(11b)** 42
4.5. Synthesis of 2-ethylbutyl-2-(5´-*O*-(2´,3´-di-*O*-levulinoyl-thiouridinyl)(phenoxy) phosphoryl) alaninate **(11c)** 45
4.6. Synthesis of cyclosaligenyl-5´-*O*-(4-thiouridineyl) phosphate **(CycloSal-4sU, 12a)** 48
4.7. Synthesis of cyclo(5-chlorosaligenyl)-5´-*O*-(4-thiouridineyl) phosphate **(Cl-CycloSal-4sU, 12b)** 51
4.8. Synthesis of 2-ethylbutyl-2-(5´-*O*-(4-thiouridineyl)(phenoxy) phosphoryl)alaninate **(ProTide-4sU, 12c**) 54

**5. Synthesis of 4sU prodrugs with *S*$^4$-biolabile protecting group (18a-c) 57**
5.1. Synthesis of 2´,3´-*O*-isopropylidene-uridine **(13)** 58
5.2. Synthesis of 5´-*O*-(*tert*-butyldimethylsilyl)-2´,3´-*O*-isopropylidene-uridine **(14)** 60
5.3. Synthesis of 4-(*S*-(β-acetylmercapto)ethyl)-5´-*O*-(*tert*-butyldimethylsilyl)-2´,3´-*O*-isopropylidene-uridine **(15a)** 62
5.4. Synthesis of 5´-*O*-(*tert*-butyldimethylsilyl)-2´,3´-*O*-isopropylidene-4-(*S*-(β-pivaloyl-mercapto)ethyl) -uridine **(15b)** 64
5.5. Synthesis of 4-(*S*-(β-acetylmercapto)ethyl)-2´,3´-*O*-isopropylidene-uridine **(16a)** 66
5.6. Synthesis of 2´,3´-*O*-isopropylidene-4-(*S*-(β-pivaloylmercapto)ethyl)-uridine **(16b)** 68
5.7. Synthesis of bis(4-acetoxybenzyl)-5´-*O*-(4-(*S*-(β-acetylmercapto)ethyl)-2´,3´-*O*-isopropylidene-uridinyl) phosphate **(17a)** 70
5.8. Synthesis of bis(*S*-pivaloyl-2-thioethyl)-5´-*O*-(4-(*S*-(β-acetylmercapto)ethyl)-2´,3´-*O*-isopropylidene-uridinyl) phosphate **(17b)** 73
5.9. Synthesis of bis(*S*-pivaloyl-2-thioethyl)-5´-*O*-(2´,3´-*O*-isopropylidene-4-(*S*-(β-pivaloylmercapto)ethyl)-uridinyl) phosphate **(17c)** 76
5.10. Synthesis of bis(4-acetoxybenzyl)-5´-*O*-(4-(*S*-(β-acetylmercapto)ethyl)-uridinyl) phosphate **(18a)** 79
5.11. Synthesis of bis(*S*-pivaloyl-2-thioethyl)-5´-*O*-(4-(*S*-(β-acetylmercapto)ethyl)-uridinyl) phosphate **(18b)** 82
5.12. Synthesis of bis(*S*-pivaloyl-2-thioethyl)-5´-*O*-(4-(*S*-(β-pivaloylmercapto)ethyl)-uridinyl) phosphate **(18c)** 85

**6. Reagent synthesis** **88**
6.1. Synthesis of bis-(4-acetoxybenzyl) *N,N*-diisopropylphosphoramidite (**I**) 88
6.1.1. Synthesis of *N,N*-diisopropylphosphoramidodichloride **(I-a)** 88
6.1.2. Synthesis of 4-acetoxy-benzylalcohol (**I-b**) 90
6.1.3. Synthesis of bis-(4-acetoxybenzyl) *N,N*-diisopropylphosphoramidite (**I**) 91
6.2. Synthesis of bis-(*S*-pivaloyl-2-thioethyl) *N,N*-diisopropylphosphoramidite (**II**) 93
6.2.1. Synthesis of *S*-(2-hydroxyethyl) thiopivaloate **(II-a)** 94
6.2.2. Synthesis of bis-(*S*-pivaloyl-2-thioethyl) *N,N*-diisopropylphosphoramidite (**II**) 95
6.3. Synthesis of (4-acetoxybenzyl)-((9*H*-fluoren-9-yl)methyl)-*N,N*-diisopropyl-phosphoramidite **(III)** 97
6.3.1. Synthesis of 4-acetoxybenzyl bis-(*N,N*-diisopropyl)phosphoramidite **(III-a)** 97
6.3.2. Synthesis of (4-acetoxybenzyl)-((9*H*-fluoren-9-yl)methyl)-*N,N*-diisopropyl-phosphoramidite **(III)** 99
6.4. Synthesis of saligenyl-*N,N*-diisopropylphosphoramidite **(IV)** 101
6.5. Synthesis of 5-chlorosaligenyl-*N,N*-diisopropylphosphoramidite **(V)** 103
6.6. Synthesis of 2-ethylbutyl (S)-2-aminopropanoate **(VI)** 105

**7. Supporting Figures** **106**

Supporting Scheme S1 106
Supporting Figure S1 107
Supporting Figure S2 108
Supporting Table S1 109

**7. References** **110**

## 1. Chemical synthesis

All reactions were carried out under argon gas atmosphere using absolute solvents. Solvents and other reagents were purchased from commercial suppliers in the highest available purity and were used without further purification. Acetonitrile and acetonitrile containing 0.3 M 5-(benzylthio)-1*H*-tetrazole (BTT) were dried over molecular sieves. Solutions of *tert*-butyl hydroperoxide (5.0-6.0 M in decane) were purchased from Sigma Aldrich and diluted with dry toluene to a final concentration of 0.5-1.0 M which was dried over molecular sieves as well before its use in chemical reactions.
Reaction progress was monitored via Polygram Sil G/$UV_{254}$ (Marchery-Nagel) thin layer chromatography plates. Non-fluorescent compounds were visualized using an $I_2$-chamber. Silica gel 60 (70-230 mesh; Fluka) was used for column chromatography.
Integrity of the synthesized compounds was analyzed by NMR spectroscopy and mass spectrometry. NMR spectra were recorded on 300 MHz Bruker Avance spectrometer, 400 MHz Bruker Avance 4 Neo spectrometer and 700 MHz Avance 4 Neo spectrometer and the reported chemical shifts were referenced to deuterated solvents ($^{1}H$: $CDCl_3$ = 7.26 ppm, $d_6$-DMSO = 2.50 ppm, $CD_3CN$ = 1.94 ppm; $^{13}C$: $CDCl_3$ = 77.16 ppm, $d_6$-DMSO = 39.52 ppm). Prodrug candidates were additionally characterized via $^{31}P$-NMR, which shifts are relative to external 85% phosphoric acid. $^{1}H$-$^{1}H$-COSY-, $^{1}H$-$^{13}C$-HSQC- and $^{1}H$-$^{13}C$-HMBC-experiments were recorded to enable signal assignment. High resolution mass spectrometry was carried out on a Thermo Scientific Q Exactive Orbitrap using an electrospray ionization system. Samples were dissolved in methanol and were measured in positive and negative ion mode with a spray voltage of 3.7 kV.

## 2. Synthesis of 4-thiouridine (4sU, 3)

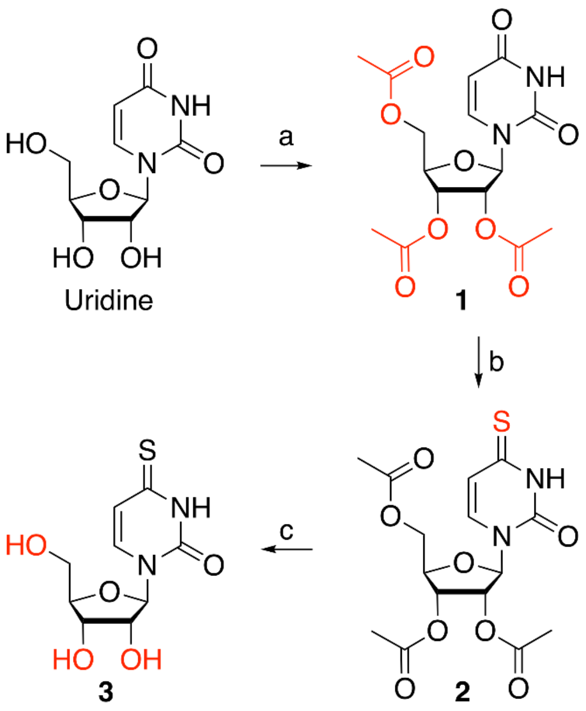

**Overview of the synthesis of 4-thiouridine (4sU, 3)**. Reaction conditions: **a)** 4.0 eq acetic anhydride, 0.1 eq 4-(dimethylamino)pyridine, 4.0 eq $Et_3N$, acetonitrile, 16 h, room temperature (RT), 99%. **b)** 1.5 eq Lawesson reagent, toluene, 2.5 h, 100 °C, 98%. **c)** 7 N $NH_3$ in MeOH, 16 h, RT, 71%. Overall yield: 69%.

**2.1 Synthesis of 2´,3´,5´-*O*-triacetyluridine (1)** [1]

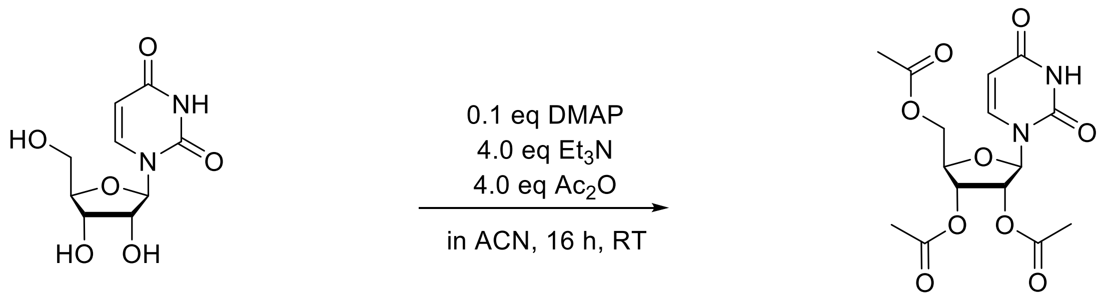


Uridine

**1**

Uridine (1.914 g, 7.84 mmol) was suspended in acetonitrile (39.2 ml) and treated with 4-(dimethylamino)pyridine (0.1 eq, 0.78 mmol, 96 mg) and triethylamine (4.0 eq, 31.35 mmol, 4.37 ml). Acetic anhydride (4.0 eq, 31.35 mmol, 2.96 ml) was added dropwise and the suspension was stirred 16 h at ambient temperature while it became homogenous. The reaction mixture was quenched with methanol and solvent was removed *in vacuo*. The oily residue was taken up in dichloromethane and was washed with saturated sodium bicarbonate solution and brine. Drying over sodium sulfate and evaporation of the solvent delivered a white foam, which was used without further purification. An analytical sample was subjected to column chromatography (0-4% MeOH in $CH_2Cl_2$). Yield: 2.871 g of **1** as white foam (99%). TLC (5% MeOH in $CH_2Cl_2$): 0.43. HR-ESI-MS (m/z): $[M+H]^+$ calculated for $[C_{15}H_{19}N_2O_9]^+$: 371.1085; found: 371.1080. $^1$H-NMR (CDCl$_3$, 400 MHz): δ 2.10, 2.13, 2.14 (3x 3 H, 3x s, 3x OAc); 4.35 (3 H, m, CH (4´), $CH_2$ (5´)); 5.33 (2 H, m, CH (2´), CH (3´)); 5.79 (1 H, dd, $^3J_{HH}$ = 8.14 Hz, $^4J_{HH}$ = 1.19 Hz, CH (5)); 6.04 (1 H, d, $^3J_{HH}$ = 4.78 Hz, CH (1´)); 7.39 (1 H, d, $^3J_{HH}$ = 8.17 Hz, CH (6)); 8.85 (1 H, s, NH) ppm. $^{13}$C-NMR (CDCl$_3$, 101 MHz): δ 20.60, 20.65, 20.92 3x $CH_3$ (OAc); 63.27 C (5´); 70.33 C (3´); 72.85 C (2´); 80.09 C (4´); 87.59 C (1´); 103.56 C (5); 139.38 C (6); 150.22 C (2); 162.64 C (4); 169.77, 170.25 3x C=O (OAc) ppm.

$^1$H-NMR (400 MHz, $CDCl_3$) of compound **1**

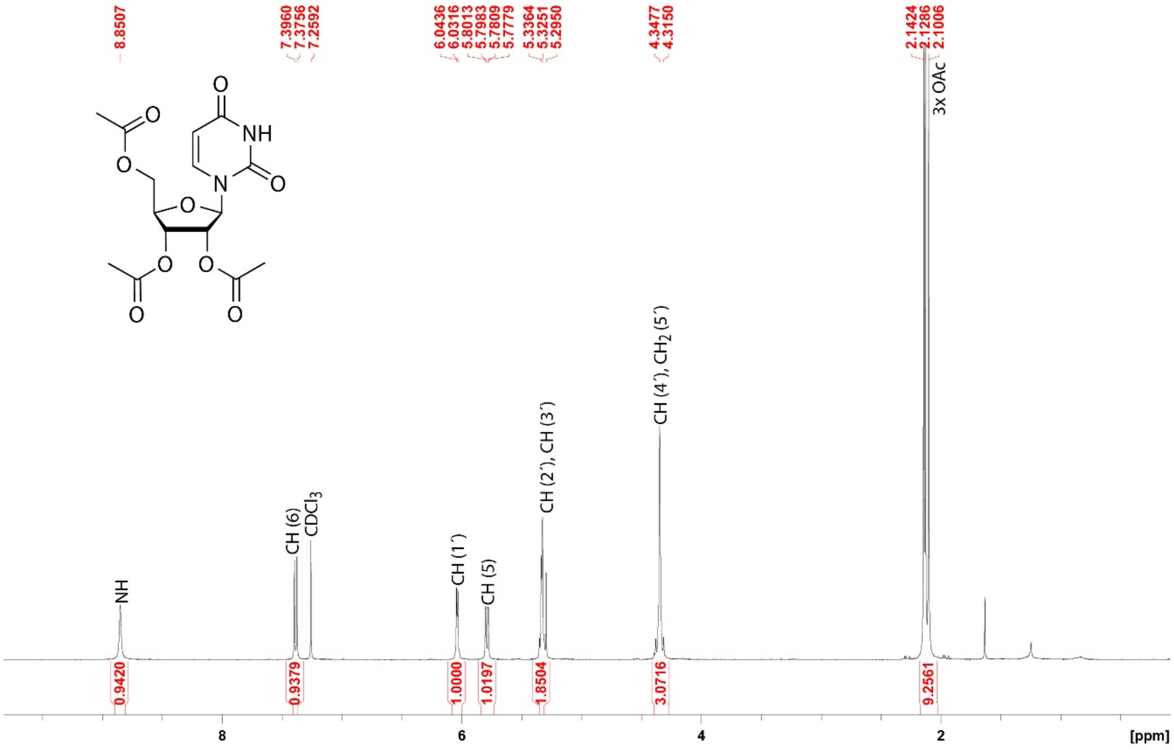


$^{13}$C-NMR (101 MHz, $CDCl_3$) of compound **1**

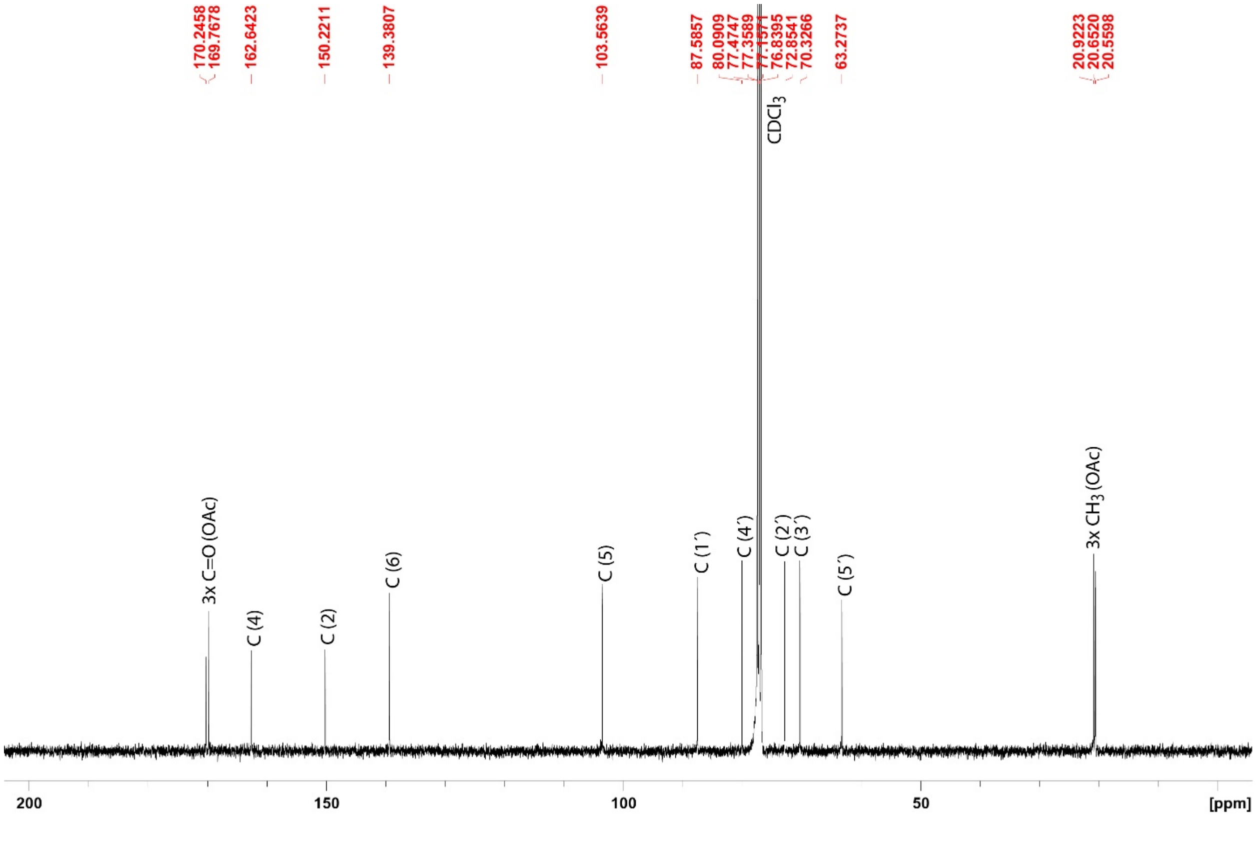

**2.2. Synthesis of 2´,3´,5´-*O*-triacetyl-4-thiouridine (2)** [2]

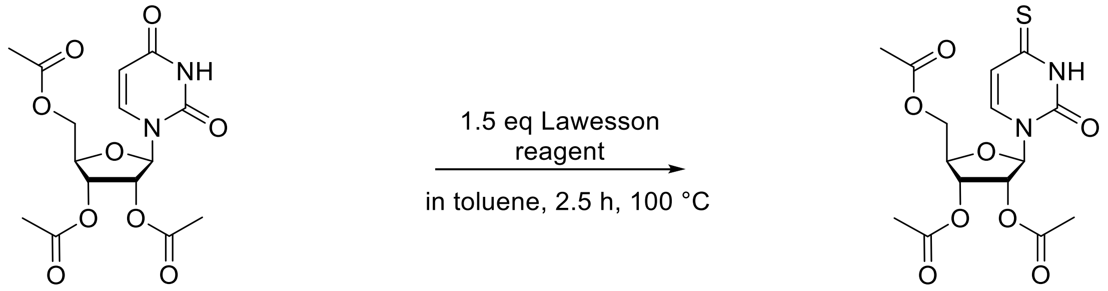


**1** **2**

Compound **1** (973 mg, 2.63 mmol) was dissolved in toluene (30.0 ml) and Lawesson Reagent (1.5 eq, 3.94 mmol, 1.595 g) was added to the reaction mixture which was then stirred 2.5 h at 100 °C. Solvents were evaporated and the oily residue was purified via column chromatography (0-3% MeOH in $CH_2Cl_2$). Yield: 995 mg of **2** as yellow foam (98%). TLC (5% MeOH in $CH_2Cl_2$): 0.55. HR-ESI-MS (m/z): $[M+H]^+$ calculated for $[C_{15}H_{19}N_2O_8S]^+$: 387.0857; found: 387.0849. $^1$H-NMR ($CDCl_3$, 400 MHz): δ 2.11, 2.12, 2.14 (3x 3 H, 3x s, 3x OAc); 4.35-4.39 (3 H, m, CH (4´), $CH_2$ (5´)); 5.29-5.37 (2 H, m, CH (3´), CH (2´)); 5.98 (1 H, d, $^3J_{HH}$ = 4.94 Hz, CH (1´)); 6.43 (1 H, d, $^3J_{HH}$ = 7.68 Hz, CH (5)); 7.23 (1 H, d, $^3J_{HH}$ = 7.71 Hz, CH (6)); 9.74 (1 H, s br., NH) ppm. $^{13}$C-NMR ($CDCl_3$, 101 MHz): δ 20.56, 20.63, 20.92 3x $CH_3$ (OAc); 63.11 C (5´); 70.20 C (3´); 73.01 C (2´); 80.26 C (4´); 88.13 C (1´); 114.07 C (5); 133.68 C (6); 147.50 C (2); 169.76, 169.79, 170.26 3x C=O (OAc); 189.69 C (4) ppm.

$^{1}$H-NMR (400 MHz, $CDCl_3$) of compound **2**

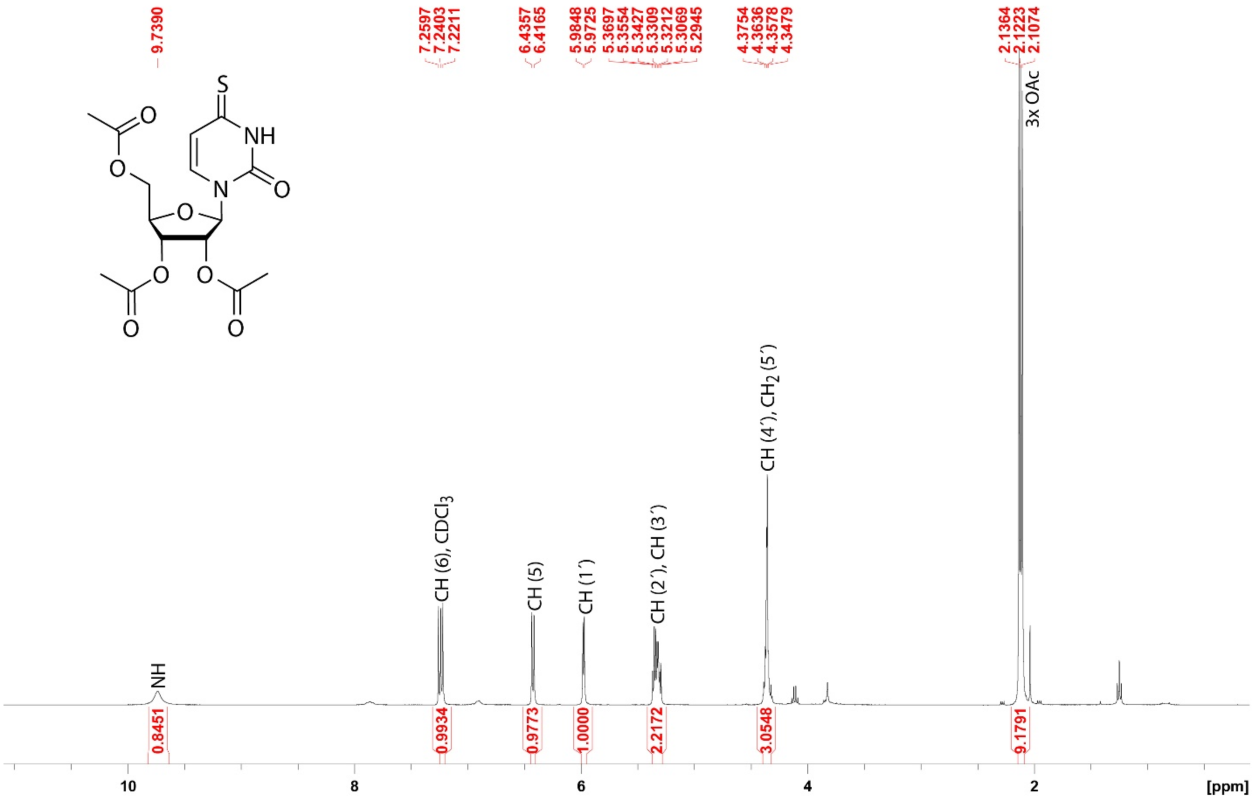


$^{13}$C-NMR (101 MHz, $CDCl_3$) of compound **2**

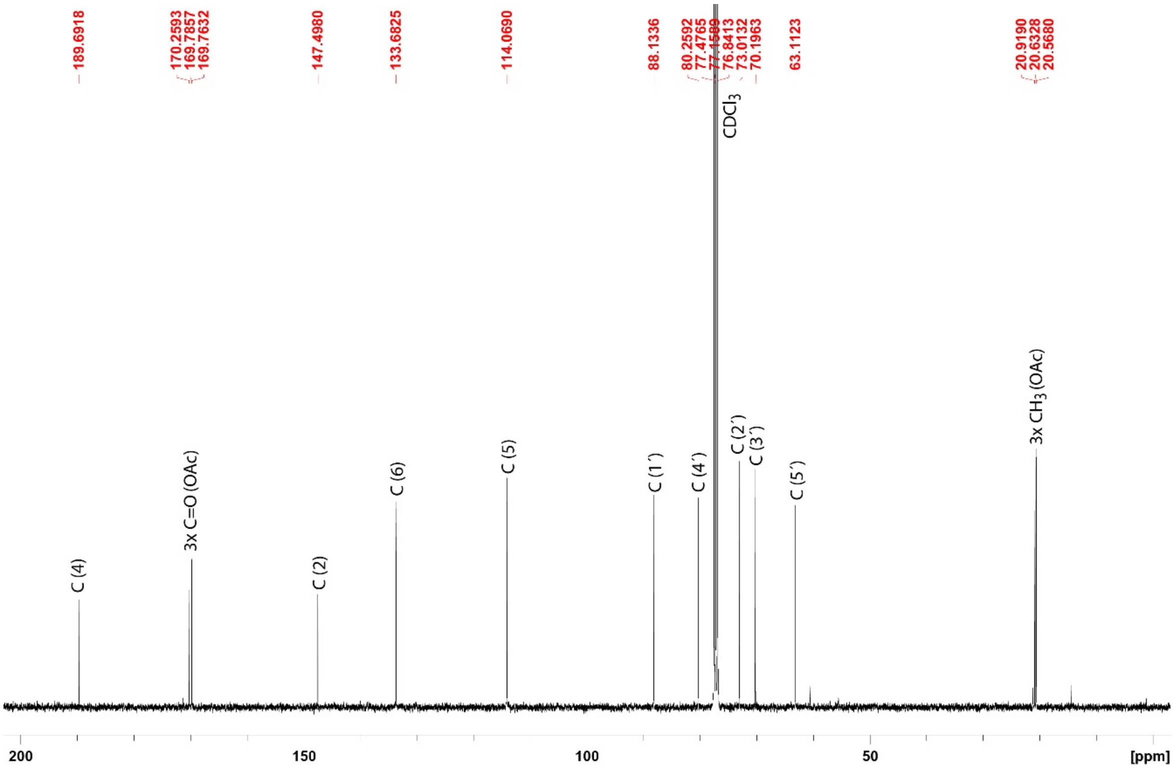

### 2.3. Synthesis of 4-thiouridine (3) [3]

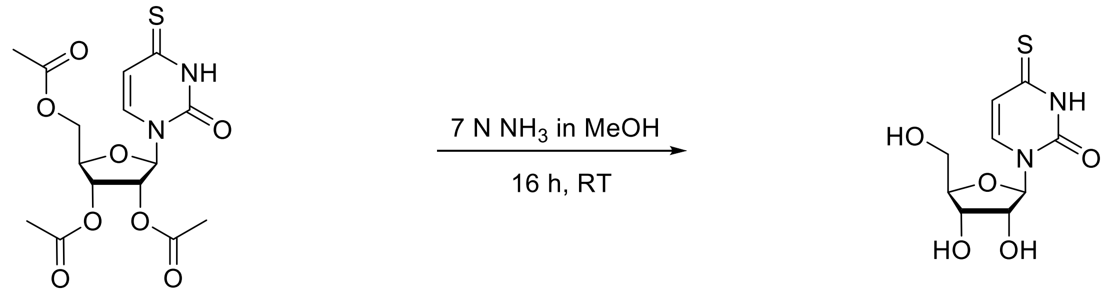


**2** **3**

Compound **2** (995 mg, 2.58 mmol) was dissolved in 7 N ammonia in methanol (30.0 ml) and was stirred 16 h at ambient temperature. Solvents were evaporated and the crude product was purified via column chromatography (3-10% MeOH in $CH_2Cl_2$). Yield: 478 mg of **3** as yellow foam (71%). TLC (20% MeOH in $CH_2Cl_2$): 0.59. HR-ESI-MS (m/z): $[M+Na]^+$ calculated for $[C_9H_{12}N_2O_5SNa]^+$: 283.0359; found: 283.0359. $^1$H-NMR ($d_6$-DMSO, 400 MHz): δ 3.56 (1 H, ddd, $^2J_{HH}$ = 12.04 Hz, $^3J_{HH}$ = 4.53 Hz, $^3J_{HH}$ = 3.39 Hz, $CH_a$ (5´)); 3.65 (1 H, ddd, $^2J_{HH}$ = 12.11 Hz, $^3J_{HH}$ = 4.87 Hz, $^3J_{HH}$ = 3.22 Hz, $CH_b$ (5´)); 3.87 (1 H, dd, $^3J_{HH}$ = 7.16 Hz, $^3J_{HH}$ = 2.96 Hz, CH (4´)); 4.96 (1 H, dd, $^3J_{HH}$ = 9.68 Hz, $^3J_{HH}$ = 4.82 Hz, CH (3´)); 4.03 (1 H, dd, $^3J_{HH}$ = 10.02 Hz, $^3J_{HH}$ = 4.98 Hz, CH (2´)); 5.09 (1 H, d, $^3J_{HH}$ =5.31 Hz, OH (3´)); 5.12 (1 H, t, $^3J_{HH}$ = 4.98 Hz, OH (5´)); 5.45 (1 H, d, $^3J_{HH}$ = 5.41 Hz, OH (2´)); 5.73 (1 H, d, $^3J_{HH}$ = 4.76 Hz, CH (1´)); 6.31 (1 H, d, $^3J_{HH}$ = 7.54 Hz, CH (5)); 7.83 (1 H, d, $^3J_{HH}$ = 7.58 Hz, CH (6)); 12.70 (1 H, s, NH) ppm. $^{13}$C-NMR ($d_6$-DMSO, 101 MHz): δ 60.51 C (5´); 69.57 C (3´); 73.92 C (2´); 84.94 C (4´); 88.48 C (1´); 112.61 C (5), 135.95 C (6), 147.98 C (2), 190.17 C (4) ppm.

<sup></sup>

$^1$H-NMR (400 MHz, $d_6$-DMSO) of compound **3**

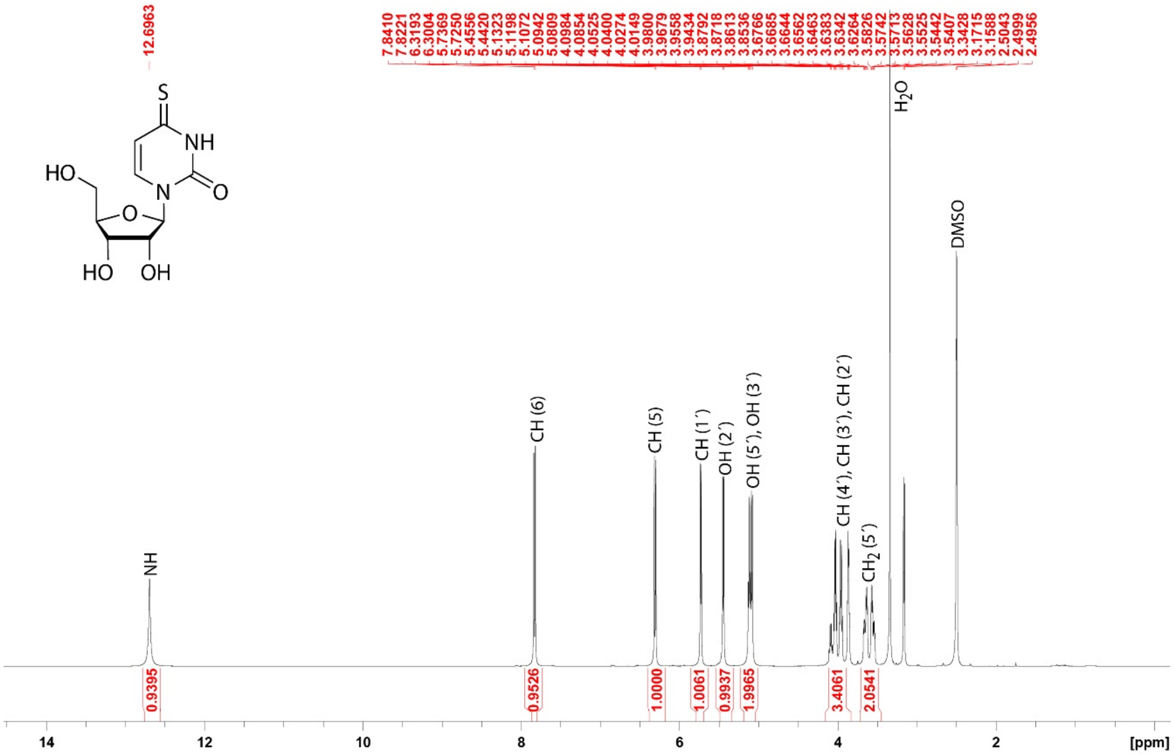


$^{13}$C-NMR (101 MHz, $d_6$-DMSO) of compound **3**

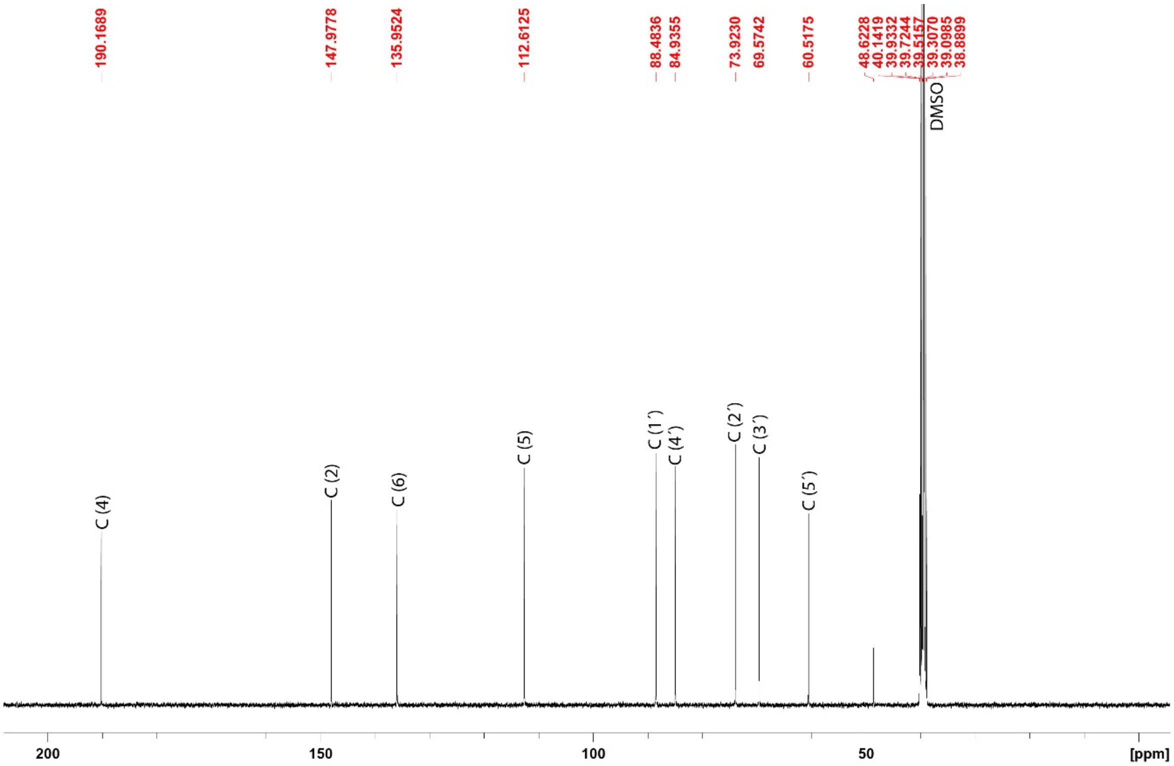

**3. Synthesis of 4-acyloxybenzyl (AB)- and *S*-pivaloyl-2-thioethyl (*t*BuSATE) 4sU 5'-*O*-monophosphates (8a-c)**

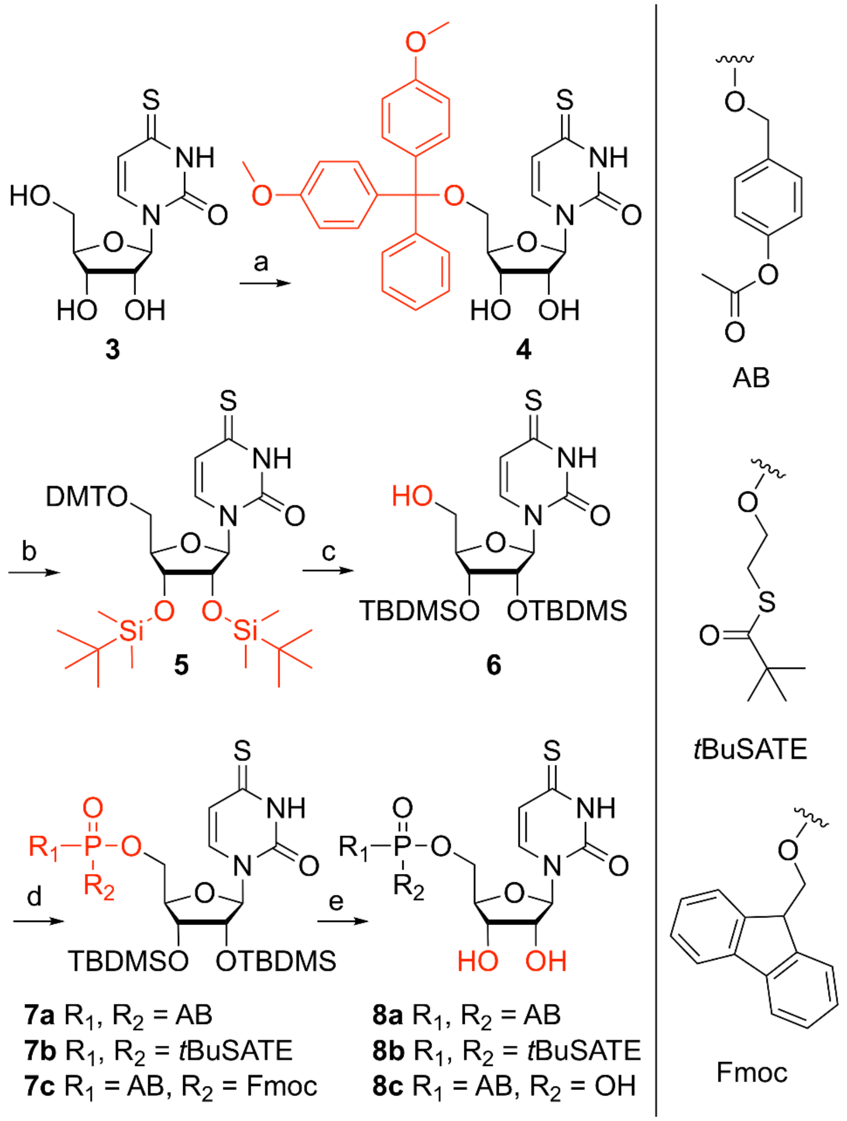


**Overview of the synthesis of (AB)$_2$-4sU 8a, (*t*BuSATE)$_2$-4sU 8b, and AB-4sU 8c**. Reaction conditions: **a)** 1.1 eq DMTCl, pyridine, 16 h, room temperature (RT), 62%. **b)** 8.0 eq imidazole, 4.0 eq TBDMSCl, DMF, 16 h, 90%. **c)** 1.5 eq benzenesulfonic acid, $CH_2Cl_2$/MeOH 9/1, 15 min, 0 °C-RT, 99%. **d)** i) for **7a**: 1.1 eq compound **I**, for **7b**: 1.1 eq compound **II**, for **7c**: 1.1 eq compound **III**; 1.1 eq 5-benzylthio-1*H*-tetrazole (BTT), 45-60 min, RT; ii) for **7a,c**: 1.1 eq *t*BuOOH, 10 min, 0 °C, 61%, 45%; for **7b**: 1.1 eq mCPBA, 5 min, 0 °C, 54%, **e)** for **8a**: 4.2 eq $Et_3N{\cdot}3HF$, 6.0 eq $Et_3N$, $CH_2Cl_2$, 16 h, RT, 49%; for **8b**: 1 M TBAF, 0.5 M HOAc, THF, 16 h, RT, 51%; for **8c**: 4.2 eq $Et_3N{\cdot}3HF$, 6.0 eq $Et_3N$, $CH_2Cl_2$, 4 h, RT, 99%. Overall yield: **8a** (17%), **8b** (15%), **8c** (25%).

### 3.1. Synthesis of 5´-*O*-(4,4´-dimethoxytrityl)-4-thiouridine (4) [4]

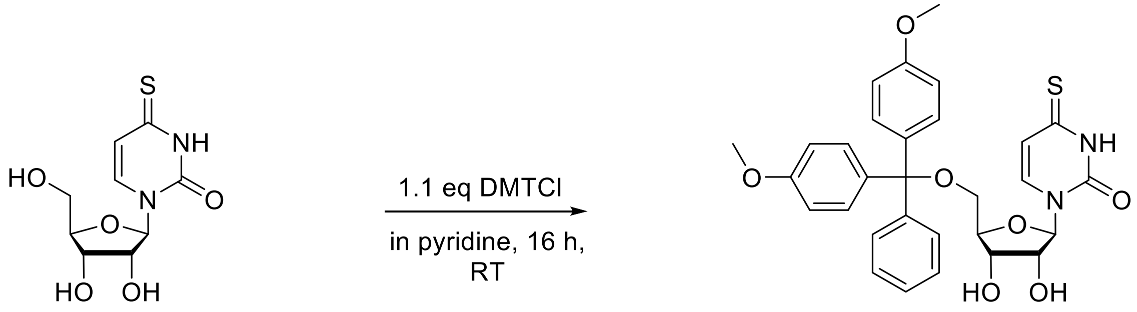


**3** **4**

Compound **3** (388 mg, 1.49 mmol) was treated three times by azeotropic distillation with pyridine and was further dried under vacuum for 45 min. It was then dissolved in pyridine (5.8 ml) and 4,4´-dimethoxytrityl chloride (1.1 eq, 1.64 mmol, 556 mg) was added in two portions over a period of 30 min. The reaction mixture was stirred 16 h at ambient temperature and was then quenched by the addition of methanol. Solvents were removed under reduced pressure, the oily residue was dissolved in dichloromethane and was washed with semi-saturated sodium bicarbonate solution and brine. The crude product was purified by column chromatography (0-4% MeOH in $CH_2Cl_2$). Yield: 546 mg of **4** as yellow foam (62%). TLC (5% MeOH in $CH_2Cl_2$): 0.31. HR-ESI-MS (m/z): $[M+Na]^+$ calculated for $[C_{30}H_{30}N_2O_7SNa]^+$: 585.1666; found: 585.1664. $^1$H-NMR ($CDCl_3$, 400 MHz): δ 3.46 (1 H, dd, $^2J_{HH}$ = 11.72 Hz, $^3J_{HH}$ = 2.80 Hz, $CH_a$ (5´)); 3.51 (1 H, dd, $^2J_{HH}$ = 11.84 Hz, $^3J_{HH}$ = 2.48 Hz, $CH_b$ (5´)); 3.63 (1 H, d, $^3J_{HH}$ = 3.88 Hz, OH (3´)); 3.75 (6 H, s, 2x $OCH_3$ (DMT)); 4.23 (1 H, m, CH (4´)); 4.48 (2 H, m, CH (2´), CH (3´)); 4.97 (1 H, s br., OH (2´)); 5.94 (1 H, d, $^3J_{HH}$ = 2.16 Hz, CH (1´)); 6.07 (1 H, d, $^3J_{HH}$ = 7.64 Hz, CH (5)); 6.84, 7.20-7.39 (13 H, m, aromat. CH (DMT)); 7.77 (1 H, d, $^3J_{HH}$ = 7.64 Hz, CH (6)); 11.23 (1 H, m, NH) ppm. $^{13}$C-NMR ($CDCl_3$, 101 MHz): δ 55.35 2x $OCH_3$ (DMT); 62.21 C (5´); 70.39 C (3´); 75.64 C (2´); 84.25 C (4´); 87.21 aromat. C (DMT); 90.38 C (1´); 113.46 C (5); 113.46, 128.19, 128.20, 130.19, 130.20 aromat. C (DMT); 135.08 C (6); 135.14, 135.34, 144.31 aromat. C (DMT); 149.13 C (2); 158.77, 158.78 aromat. C DMT; 189.52 C (4) ppm.

$^{1}$H-NMR (400 MHz, $CDCl_3$) of compound **4**

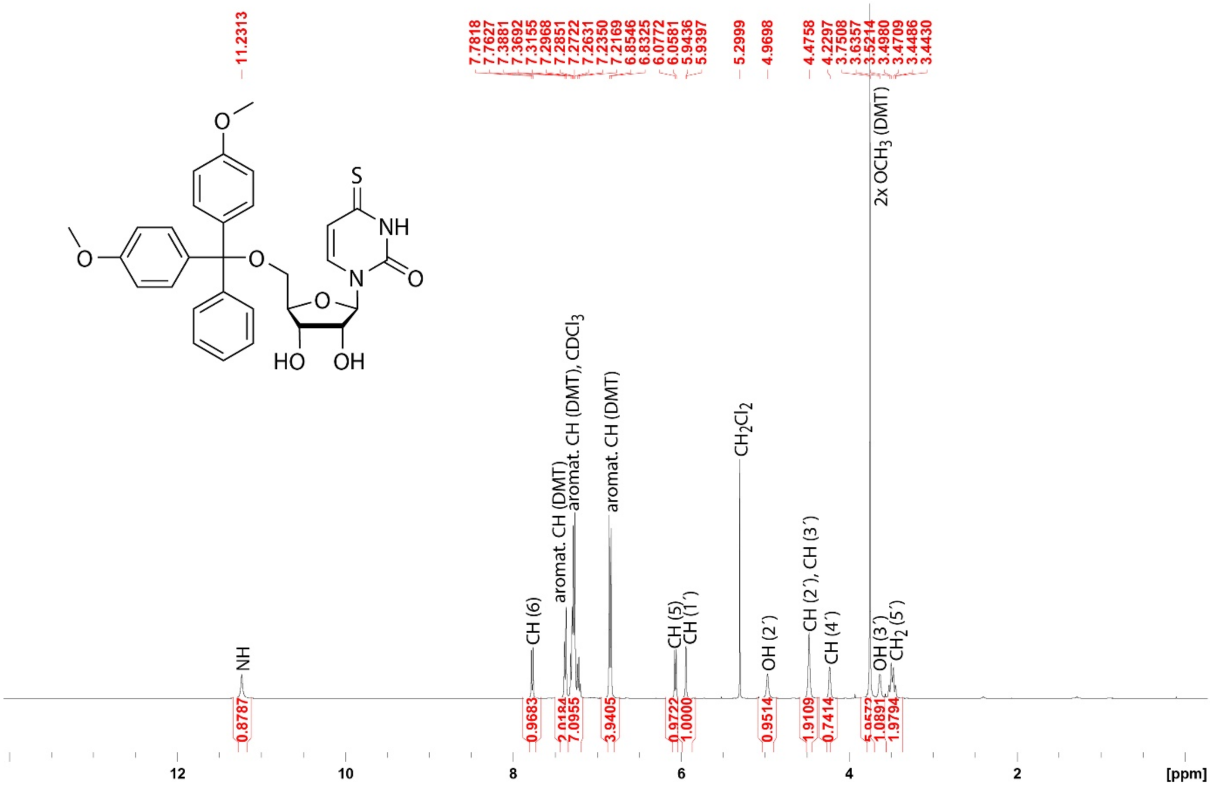


$^{13}$C-NMR (101 MHz, $CDCl_3$) of compound **4**

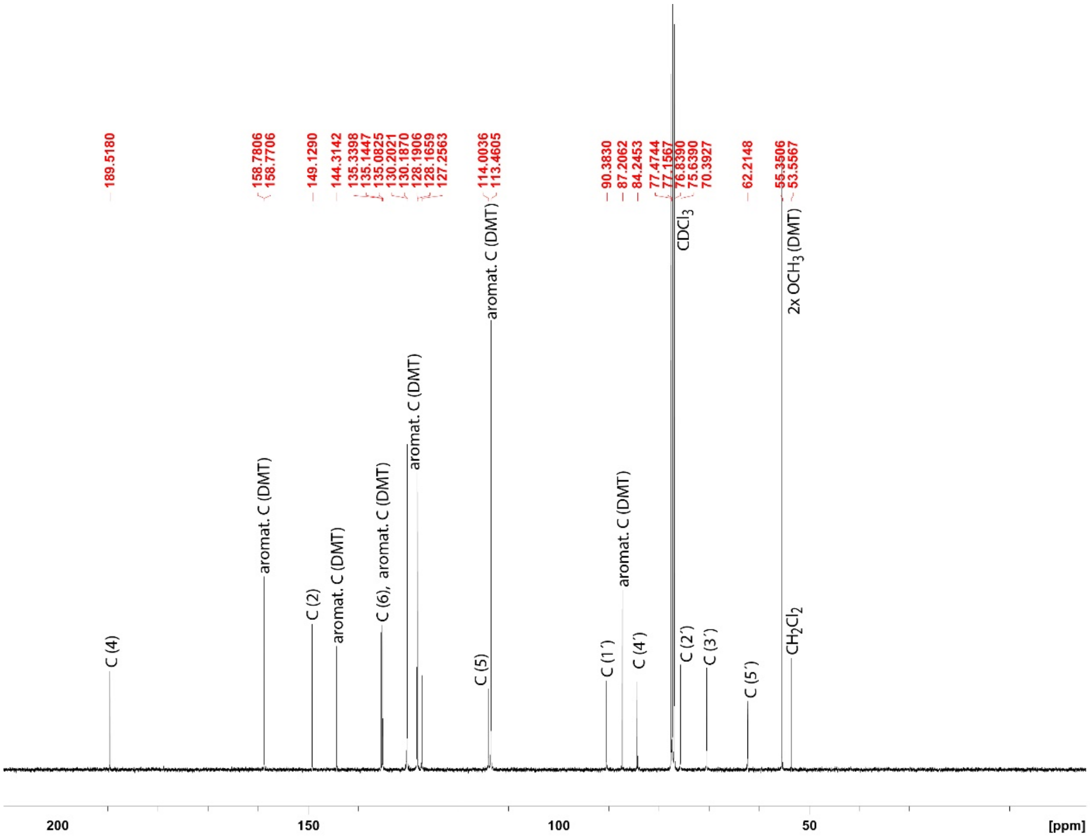

**3.2. Synthesis of 2´,3´-di-*O*-(*tert*-butyldimethylsilyl)-5´-*O*-(4,4´-dimethoxytrityl)-4-thiouridine (5)**

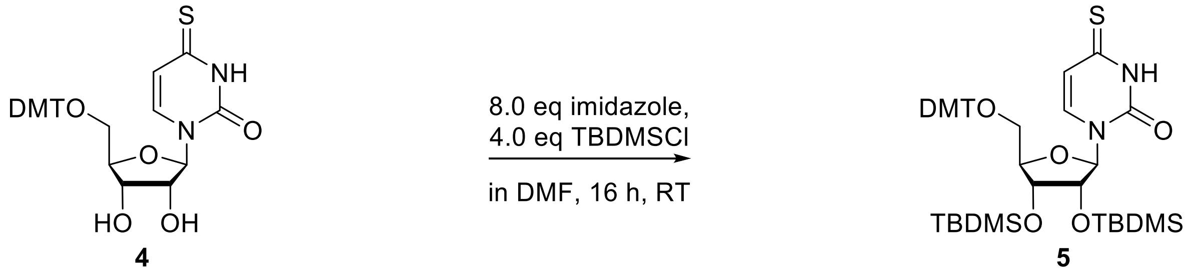

Compound **4** (750 mg, 1.33 mmol) was dissolved in *N,N*-dimethylformamide (3.0 ml) and imidazole (8.0 eq, 10.67 mmol, 726 mg) and *tert*-butyldimethylsilyl chloride (4.0 eq, 5.34 mmol, 804 mg) were added consecutively. The colorless solution was allowed to stir 16 h at ambient temperature followed by dilution with ethyl acetate and extraction with brine. The combined organic layers were dried over sodium sulfate and purified via column chromatography (0-1% MeOH in $CH_2Cl_2$). Yield: 952 mg of **5** as yellow foam (90%). TLC (3% MeOH in $CH_2Cl_2$): 0.65. HR-ESI-MS (m/z): $[M+Na]^+$ calculated for $[C_{42}H_{58}N_2O_7Si_2SNa]^+$: 813.3395; found: 813.3398. $^1$H-NMR ($CDCl_3$, 400 MHz): δ -0.058, -0.032, 0.10, 0.17 (4x 3 H, 4x s, 4x $CH_3$ (TBDMS)); 0.78, 0.90 (2x 9 H, 2x s, 2x *t*-Bu (TBDMS)); 3.36 (1 H, dd, $^2J_{HH}$ = 11.13 Hz, $^3J_{HH}$ = 1.18 Hz, $CH_a$ (5´)); 3.72 (1 H, dd, $^2J_{HH}$ = 11.13 Hz, $^3J_{HH}$ = 1.54 Hz, $CH_b$ (5´)); 3.81 (6 H, s, 2x $OCH_3$ (DMT); 4.17 (3 H, m, CH (2´), CH (3´), CH (4´)); 5.80 (1 H, d, $^3J_{HH}$ = 1.84 Hz, CH (1´)); 5.94 (1 H, dd, $^3J_{HH}$ = 7.64 Hz, $^4J_{HH}$ = 1.50 Hz, CH (5)); 6.83-6.86, 7.14-7.37 (13 H, m, aromat. CH (DMT)); 8.06 (1 H, d, $^3J_{HH}$ = 7.66 Hz, CH (6)); 9.32 (1 H, s, NH) ppm. $^{13}$C-NMR ($CDCl_3$, 101 MHz): δ -4.84, -4.81, -4.27, -3.91 4x $CH_3$ (TBDMS); 18.07, 18.11 2x $C_q$ (TBDMS); 25.87, 25.788 2x *t*-Bu (TBDMS); 55.43 2x $OCH_3$ (DMT); 61.35 C (5´); 70.60 C (3´); 76.26 C (2´); 83.07 C (4´); 87.45 aromat. C (DMT); 90.20 C (1´); 113.10 C (5); 113.37, 113.42, 128.12, 128.52, 130.44, 130.46 aromat. C (DMT); 134.94 C (6); 135.07, 135.29, 143.98 aromat. C (DMT); 147.50 C (2); 158.97 aromat. C DMT; 189.55 C (4) ppm.

$^{1}$H-NMR (400 MHz, $CDCl_3$) of compound **5**

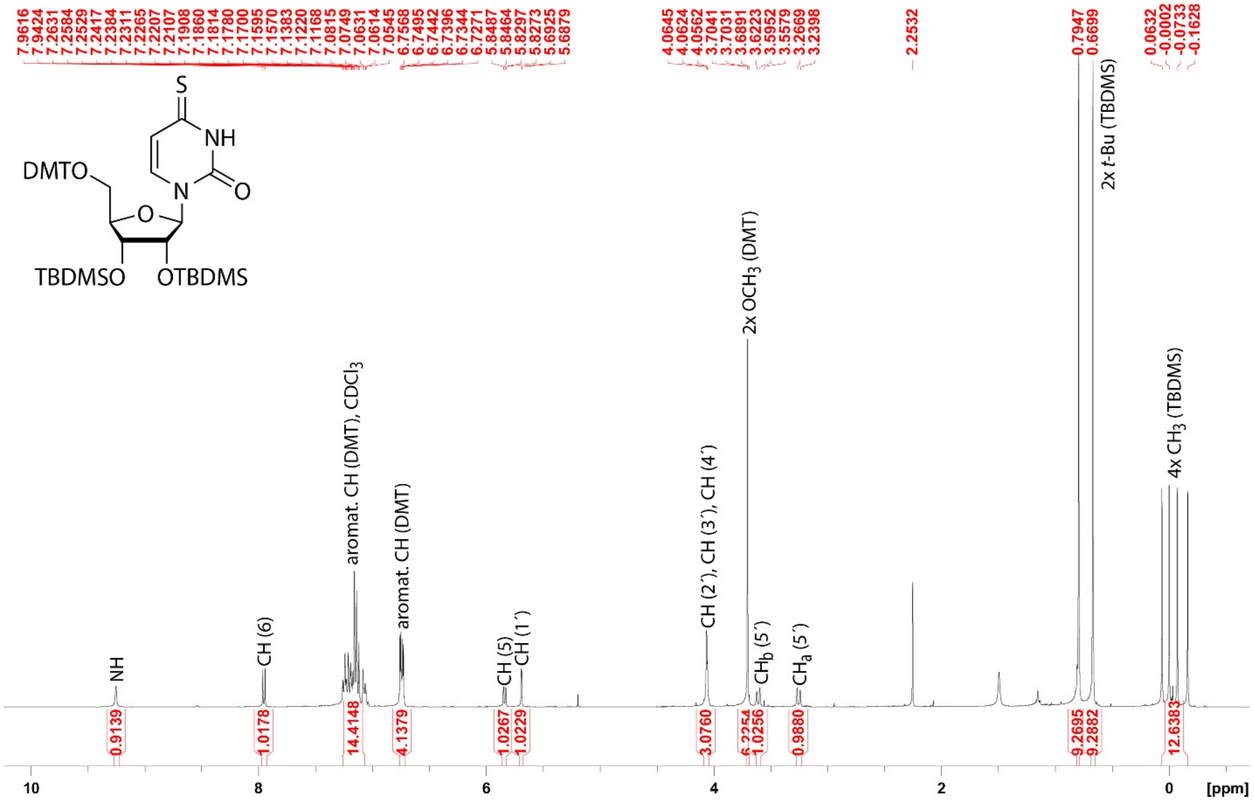


$^{13}$C-NMR (101 MHz, $CDCl_3$) of compound **5**

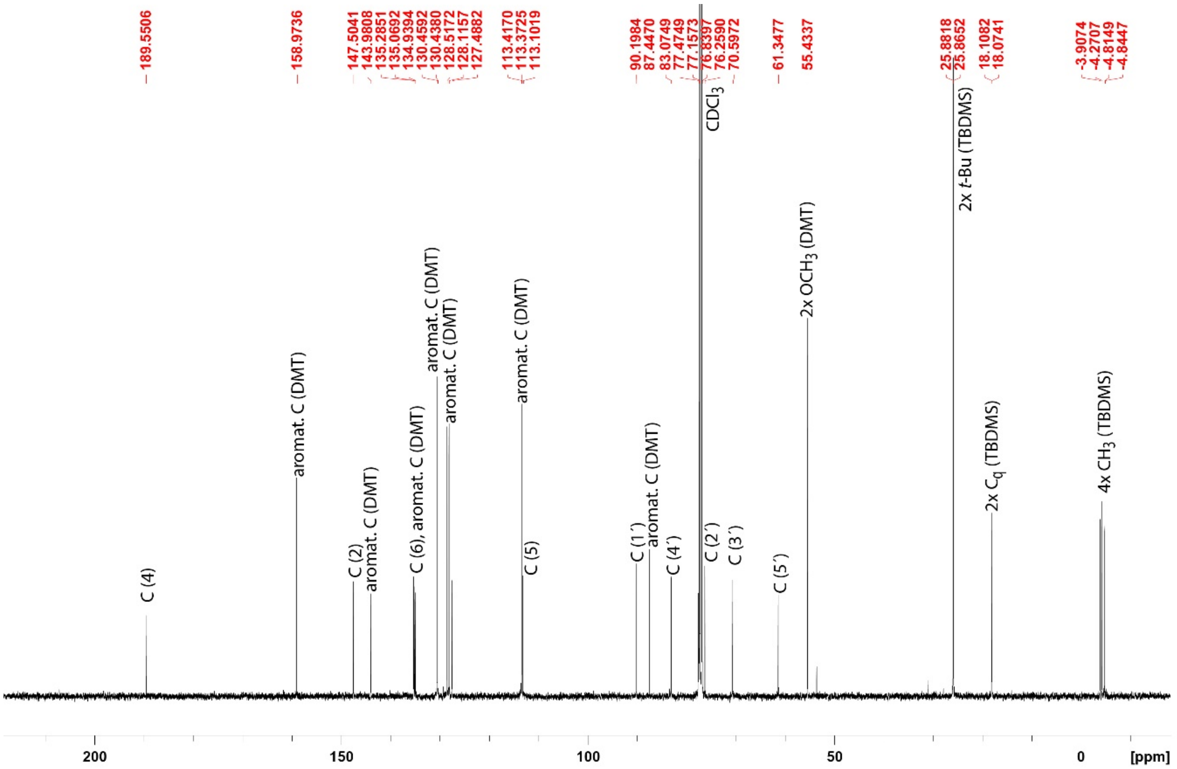

### 3.3. Synthesis of 2´,3´-di-*O*-(*tert*-butyldimethylsilyl)-4-thiouridine (6)

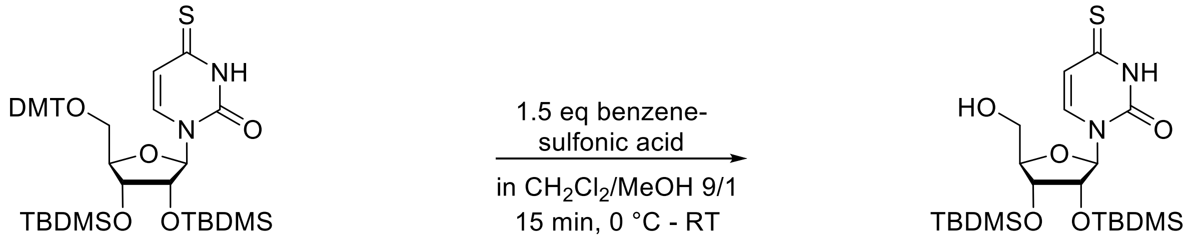


A solution of compound **5** (200 mg, 0.25 mmol) in dichloromethane/methanol (9/1, 3.3 ml) was treated with benzenesulfonic acid (1.5 eq, 0.38 mmol, 60 mg) in dichloromethane/methanol (9/1, 3.3 ml) at 0 °C. The solution immediately turned orange. After 15 min, saturated sodium bicarbonate solution was added and the reaction mixture was stirred for another 5 min. The organic phase was separated and the aqueous layer was washed two times with dichloromethane. The combined organic extracts were extracted with saturated sodium bicarbonate solution and brine. Drying over sodium sulfate was followed by column chromatography (0-4% MeOH in $CH_2Cl_2$). Yield: 122 mg of **6** as yellow foam (99%). TLC (5% MeOH in $CH_2Cl_2$): 0.56. HR-ESI-MS (m/z): $[M+H]^+$ calculated for $[C_{21}H_{41}N_2O_5Si_2S]^+$: 489.2269; found: 489.2242. $^1$H-NMR ($CDCl_3$, 400 MHz): δ -0.064, -0.076, 0.087, 0.093 (4x 3 H, 4x s, 4x $CH_3$ (TBDMS)); 0.89, 0.91 (2x 9 H, 2x s, 2x *t*-Bu (TBDMS)); 2.72 (1 H, s br., OH (5´)); 3.74 (1 H, dd, $^2J_{HH}$ = 12.13 Hz, $^3J_{HH}$ = 2.12 Hz, $CH_a$ (5´)); 3.98 (1 H, dd, $^2J_{HH}$ = 12.17 Hz, $^3J_{HH}$ = 1.74 Hz, $CH_b$ (5´)); 4.11 (1 H, m, CH (4´)); 4.16 (1 H, t, $^3J_{HH}$ = 4.25 Hz, CH (3´)); 4.46 (1 H, t, $^3J_{HH}$ = 4.51 Hz, CH (2´)); 5.49 (1 H, d, $^3J_{HH}$ = 4.69 Hz, CH (1´)); 6.40 (1 H, d, $^3J_{HH}$ = 7.61 Hz, CH (5)); 7.56 (1 H, d, $^3J_{HH}$ = 7.65 Hz, CH (6)); 9.49 (1 H, s, NH) ppm. $^{13}$C-NMR ($CDCl_3$, 101 MHz): δ -4.70, -4.65, -4.56, -4.25 4x $CH_3$ (TBDMS); 18.09, 18.21 2x $C_q$ (TBDMS); 25.91, 25.95 2x *t*-Bu (TBDMS); 61.41 C (5´); 71.36 C (3´); 74.18 C (2´); 85.88 C (4´); 93.75 C (1´); 113.16 C (5); 137.21 C (6); 147.65 C (2); 189.55 C (4) ppm.

$^{1}H$-NMR (400 MHz, $CDCl_3$) of compound **6**

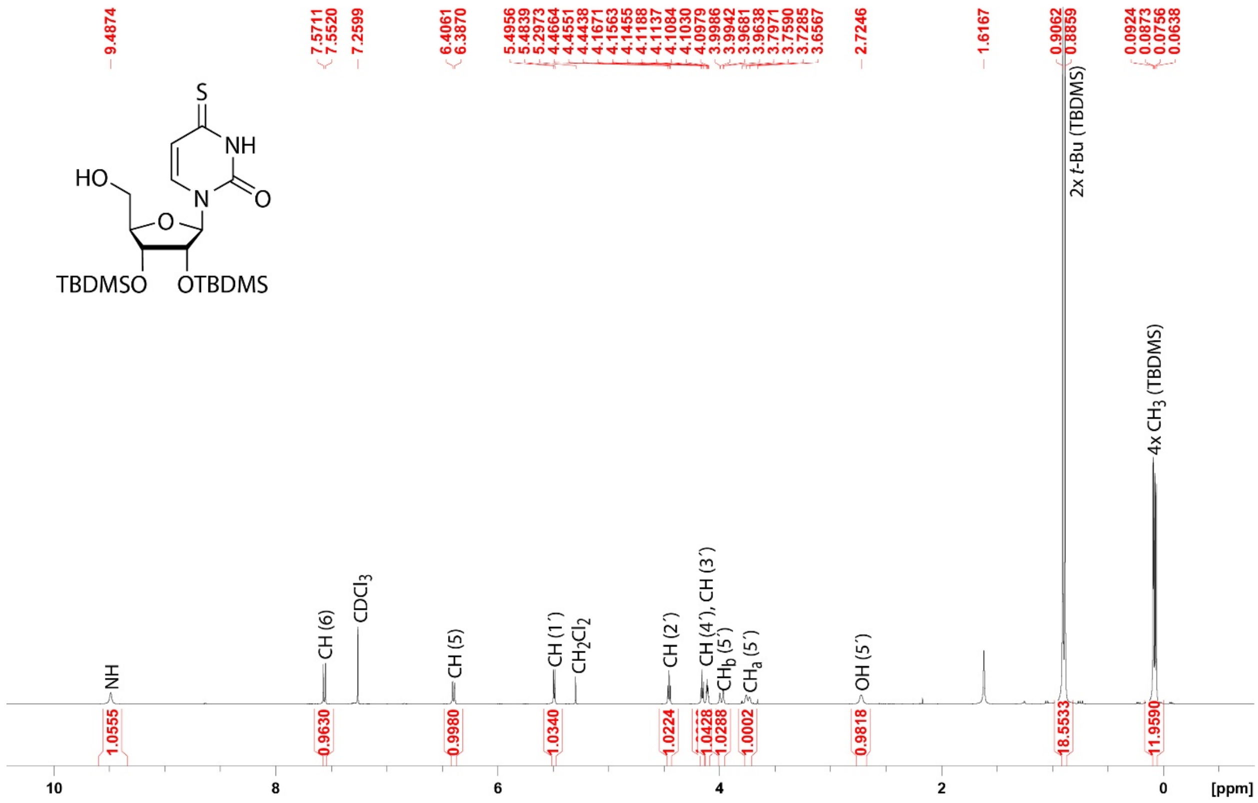


$^{13}C$-NMR (101 MHz, $CDCl_3$) of compound **6**

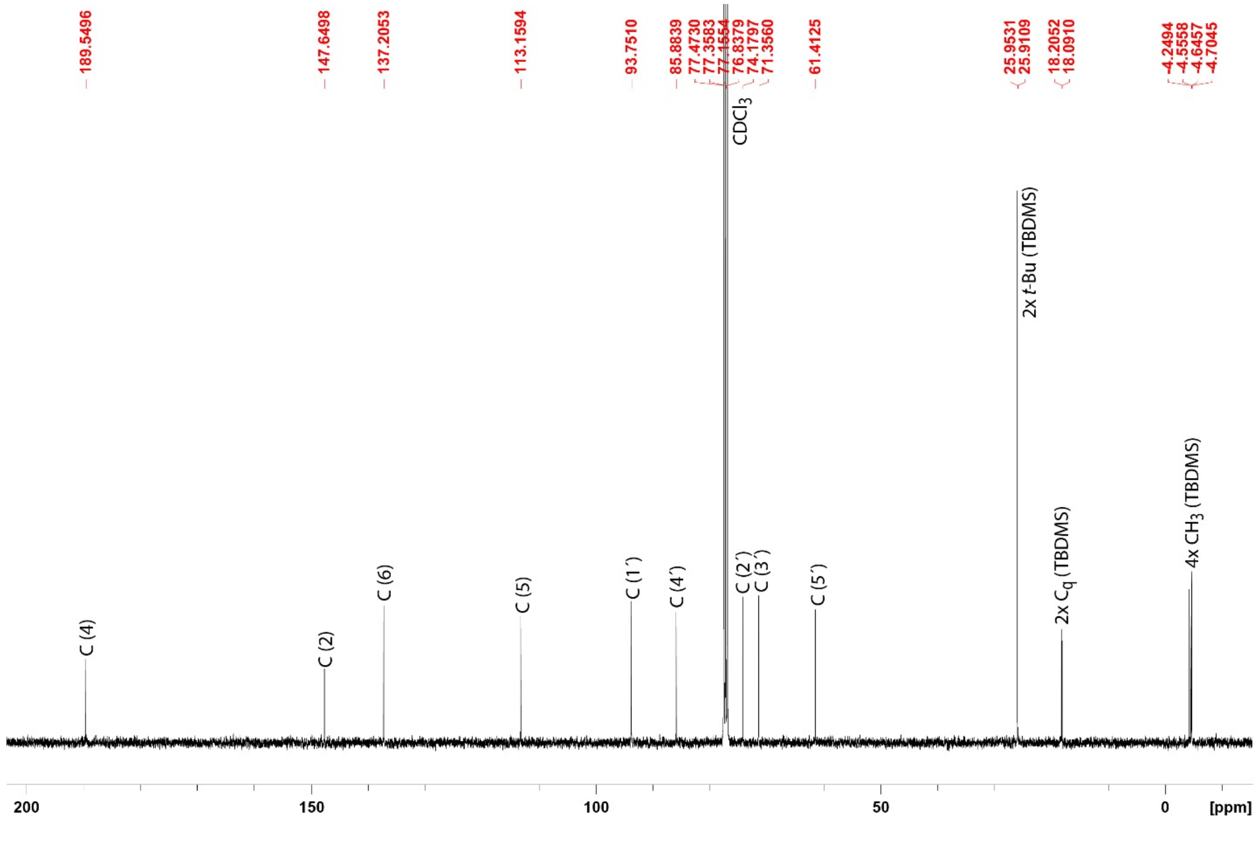

### 3.4. Synthesis of bis(4-acetoxybenzyl)-5´-*O*-(2´,3´-di-*O*-(*tert*-butyldimethylsilyl)-4-thiouridinyl) phosphate (7a) [5,6]

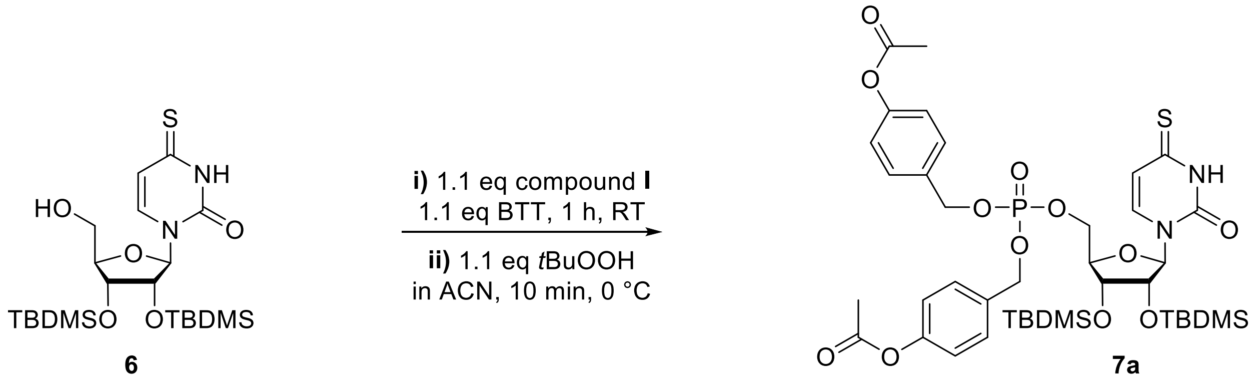


**6** **7a**

Compound **I** (1.1 eq, 0.34 mmol, 158 mg) and compound **6** (0.31 mmol, 152 mg) were coevaporated 3 times with acetonitrile and were subsequently dried under high vacuum for a few minutes. 5-(Benzylthio)-1*H*-tetrazole (0.3 M in acetonitrile; 1.1 eq, 1.14 ml, dried over molecular sieves) was added and the solution was stirred for 1 h at ambient temperature. It was then cooled to 0 °C and a dry solution of *tert*-butyl hydroperoxide (in decane, 1.1 eq, 0.34 mmol, 0.68 ml 0.5 M in toluene, dried over molecular sieves) was introduced dropwise. Solvent was removed under reduced pressure after 10 min and the oily residue purified via column chromatography (10-50% ethyl acetate in c-hexane). Yield: 163 mg of **7a** as yellow foam (61%). TLC (2% MeOH in $CH_2Cl_2$): 0.33. HR-ESI-MS (m/z): $[M+H]^+$ calculated for $[C_{39}H_{58}N_2O_{12}PSi_2S]^+$: 865.2981; found: 865.2942. $^1$H-NMR ($CDCl_3$, 400 MHz): δ 0.042, 0.058, 0.060, 0.089 (4x 3 H, 4x s, 4x $CH_3$ (TBDMS)); 0.87, 0.88 (2x 9 H, 2x s, 2x *t*-Bu (TBDMS)); 2.30 (6 H, 2x s, Acetyl (AB)); 3.98 (1 H, dd, $^3J_{HH}$ = 5.90 Hz, $^3J_{HH}$ = 4.30 Hz, CH (3´)); 4.08-4.16 (3 H, m, $CH_a$ (5´), CH (2´), CH (4´)); 4.30 (1 H, ddd, $^2J_{HH}$ = 11.43 Hz, $^3J_{HH}$ or $^3J_{HP}$ = 5.63 Hz, $^3J_{HH}$ or $^3J_{HP}$ = 2.45 Hz, $CH_b$ (5´)); 4.98-5.04 (4 H, m, $CH_2$ (AB)); 5.67 (1 H, d, $^3J_{HH}$ = 3.14 Hz, CH (1´)); 6.24 (1 H, d, $^3J_{HH}$ = 7.68 Hz, CH (5)); 7.09 (4 H, d, $^3J_{HH}$ = 8.44 Hz, aromat. CH (AB)); 7.34 (4 H, d, $^3J_{HH}$ = 8.12 Hz, aromat. CH (AB)); 7.45 (1 H, d, $^3J_{HH}$ = 7.72 Hz, CH (6)), 9.43 (1 H, s, NH) ppm. $^{13}$C-NMR ($CDCl_3$, 101 MHz): δ -4.88, -4.74, -4.44, -4.13 4x $CH_3$ (TBDMS); 18.10, 18.11 2x $C_q$ (TBDMS); 21.26 2x Acetyl (AB); 25.88, 25.89 2x *t*-Bu (TBDMS); 65.64 (d, $^2J_{CP}$ = 5.09 Hz, C (5´)); 69.33 (d, $^2J_{CP}$ = 5.09 Hz, $CH_2$ ($AB_a$)); 69.38 (d, $^2J_{CP}$ = 5.08 Hz, $CH_2$ ($AB_b$)); 70.51 C (3´); 75.39 C (2´); 81.97 (d, $^3J_{CP}$ = 7.99 Hz, C (4´)); 90.50 C (1´); 113.13 C (5); 122.18, 129.48, 129.53 aromar. C (AB); 132.89-132.87 (2x d, aromat. $C_q$ (AB)); 134.60 C (6); 147.40 C (2); 151.19, 151.21 aromat. $C_q$ (AB); 169.42 2x C=O (AB); 189.50 C (4) ppm. $^{31}$P-NMR ($CDCl_3$, 162 MHz): δ -0.73 ppm.

$^1$H-NMR (400 MHz, $CDCl_3$) of compound **7a**

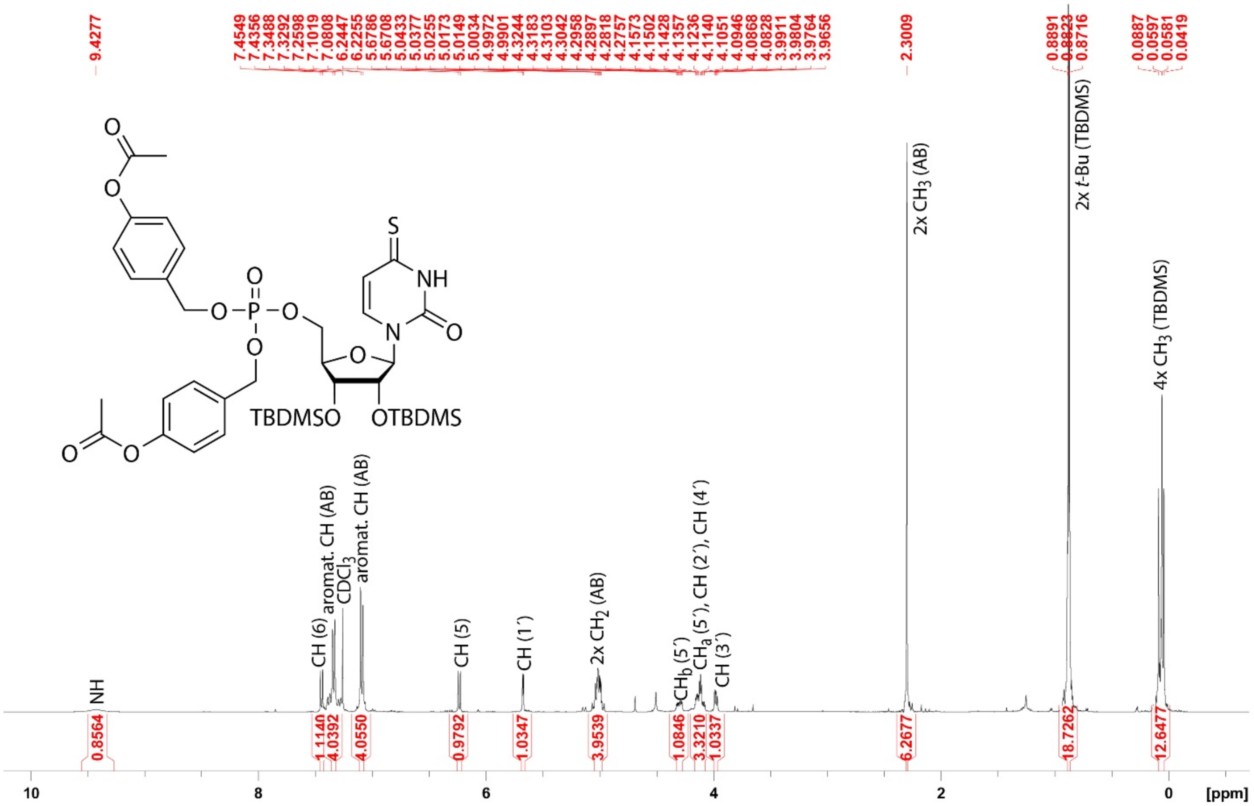


$^{13}$C-NMR (101 MHz, $CDCl_3$) of compound **7a**

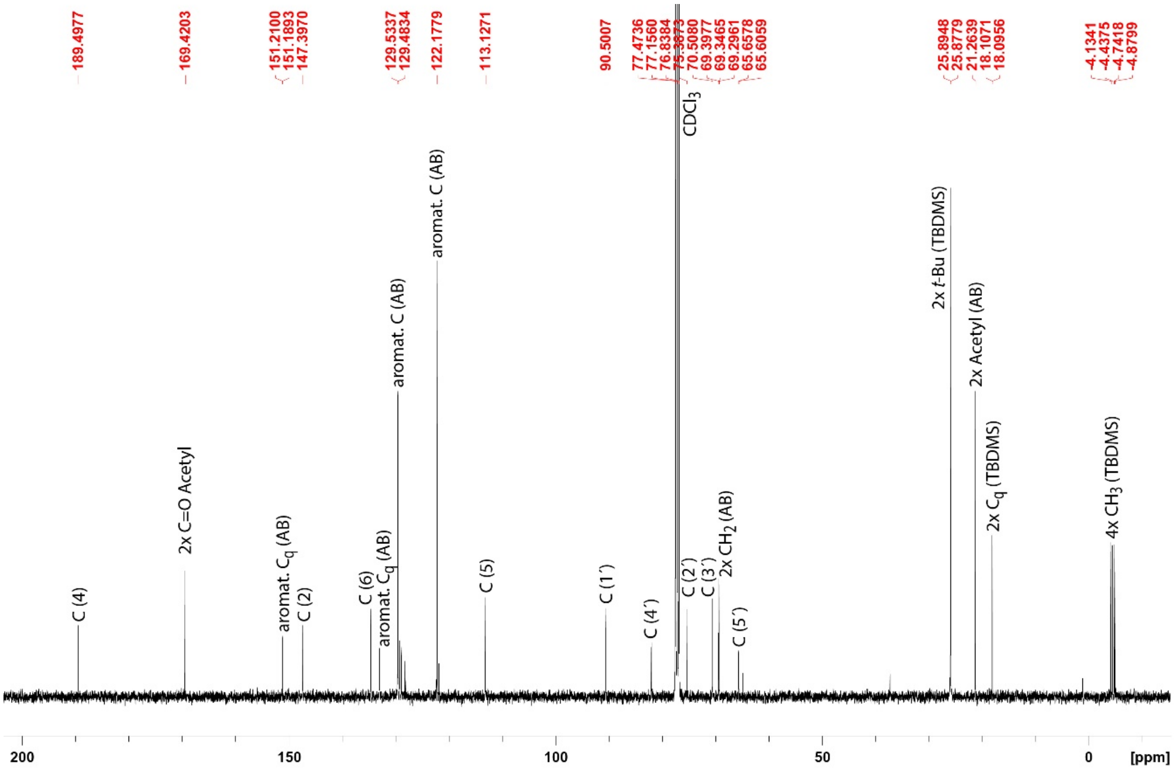

$^{31}$P-NMR (162 MHz, $CDCl_3$) of compound **7a**

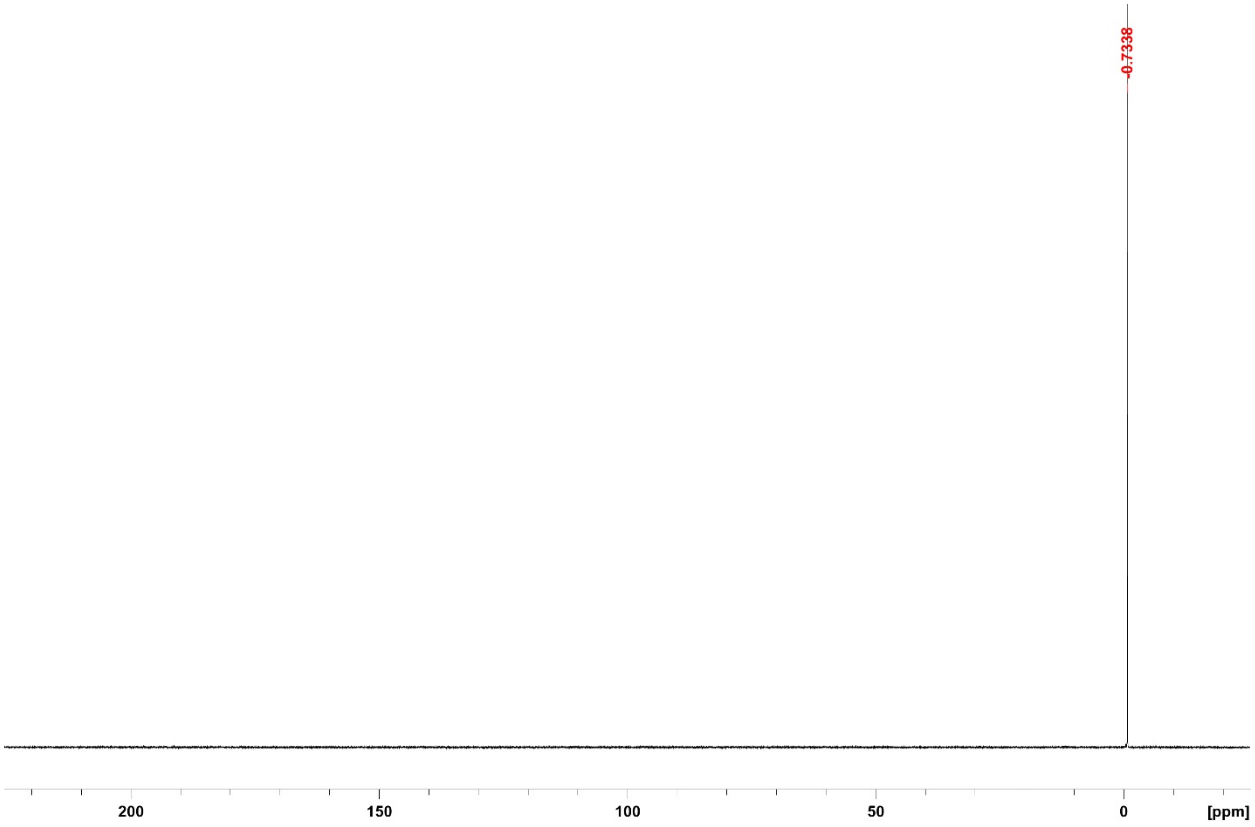

### 3.5. Synthesis of bis(*S*-pivaloyl-2-thioethyl)-5´-*O*-(2´,3´-di-*O*-(*tert*-butyldimethylsilyl)-4-thiouridinyl) phosphate (7b) [5,6]

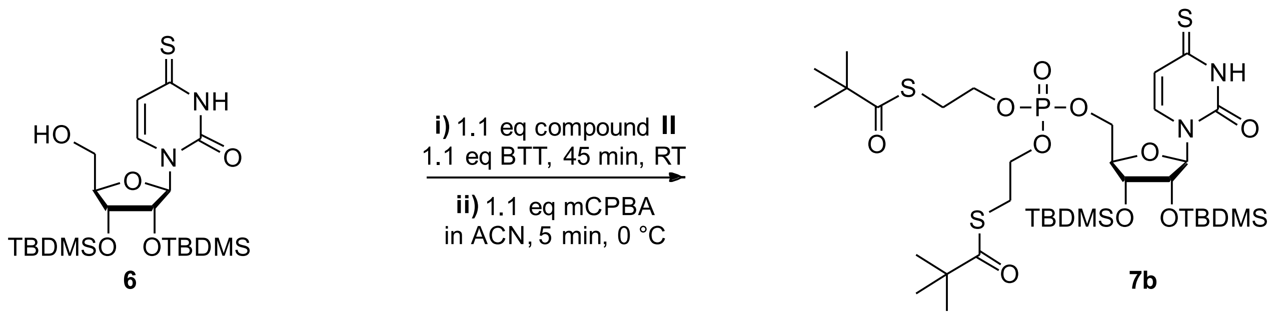


Compound **II** (1.1 eq, 0.23 mmol, 104 mg) and compound **6** (0.21 mmol, 102 mg) were coevaporated three times with acetonitrile and were subsequently dried under high vacuum for a few minutes. 5-(Benzylthio)-1*H*-tetrazole (0.3 M in acetonitrile; 1.1 eq, 0.77 ml, dried over molecular sieves) was added and the solution was stirred for 45 min at ambient temperature. It was then cooled to 0 °C and 3-chloroperbenzoic acid (1.1 eq, 0.23 mmol, 40 mg) was introduced to the reaction mixture. Solvent was removed under reduced pressure after 5 min and the oily residue purified via column chromatography (10-30% ethyl acetate in c-hexane). Yield: 88 mg of **7b** as yellow foam (54%). TLC (2% MeOH in $CH_2Cl_2$): 0.38. HR-ESI-MS (m/z): $[M+H]^+$ calculated for $[C_{35}H_{66}N_2O_{10}PSi_2S_3]^+$: 857.3150; found: 857.3116. $^1$H-NMR ($CDCl_3$, 400 MHz): δ 0.087, 0.092, 0.12 (4x 3 H, 3x s, 4x $CH_3$ (TBDMS)); 0.89, 0.90 (2x 9 H, 2x s, 2x *t*-Bu (TBDMS)); 1.23 (2x 9 H, s, *t*-Bu (SATE)); 3.12-3.16 (4 H, m, 2x $SCH_2$ (SATE)); 4.03 (1 H, dd, $^3J_{HH}$ = 5.69 Hz, $^3J_{HH}$ = 4.31 Hz, CH (3´)); 4.11-4.16 (5 H, m, CH (4´), 2x $POCH_2$ (SATE)); 4.18-4.22 (2 H, m, CH (2´), $CH_a$ (5´)); 4.38-4.43 (1 H, m, $CH_b$ (5´)); 5.71 (1 H, d, $^3J_{HH}$ = 3.08 Hz, CH (1´)); 6.44 (1 H, d, $^3J_{HH}$ = 7.64 Hz, CH (5)); 7.56 (1 H, d, $^3J_{HH}$ = 7.68 Hz, CH (6)); 9.58 (1 H, s, NH) ppm. $^{13}$C-NMR ($CDCl_3$, 101 MHz): δ -4.82, -4.73, -4.37, -4.10 4x $CH_3$ (TBDMS); 18.11, 18.13 2x $C_q$ (TBDMS); 25.89, 25.93 2x *t*-Bu (TBDMS); 27.43 2x *t*-Bu (SATE); 28.65, 28.73 2x $SCH_2$ (SATE); 46.70 2x $C_q$ (SATE); 65.66 (d, $^2J_{CP}$ = 5.81 Hz, C (5´)); 66.63-66.72 (2x d, $POCH_2$ (SATE)); 70.49 C (3´); 75.49 C (2´); 82.01 (d, $^3J_{CP}$ = 8.00 Hz, C (4´)); 90.59 C (1´); 113.25 C (5); 134.57 C (6); 147.52 C (2); 189.58 C (4); 205.61, 205.62 2x C=O (SATE) ppm. $^{31}$P-NMR ($CDCl_3$, 162 MHz): δ -1.41 ppm.

<sup></sup>

$^{1}$H-NMR (400 MHz, $CDCl_3$) of compound **7b**

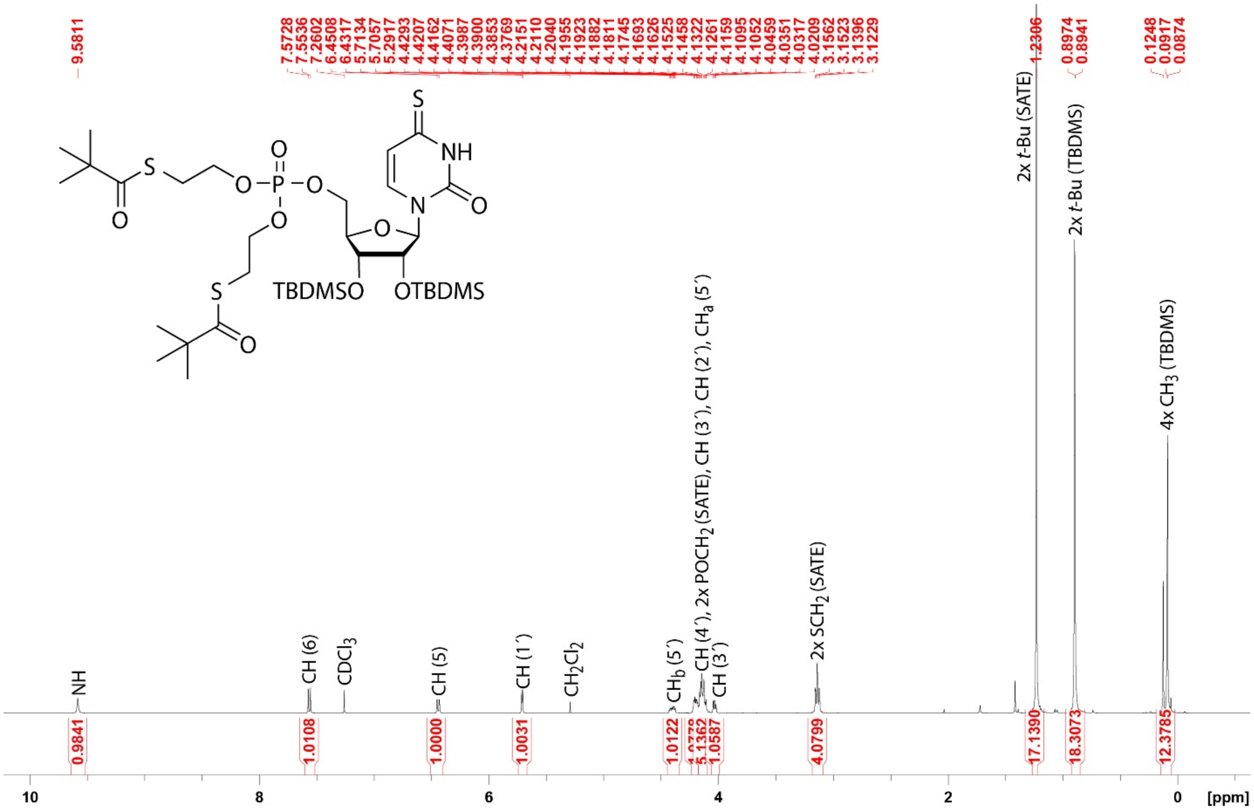

$^{13}$C-NMR (101 MHz, $CDCl_3$) of compound **7b**

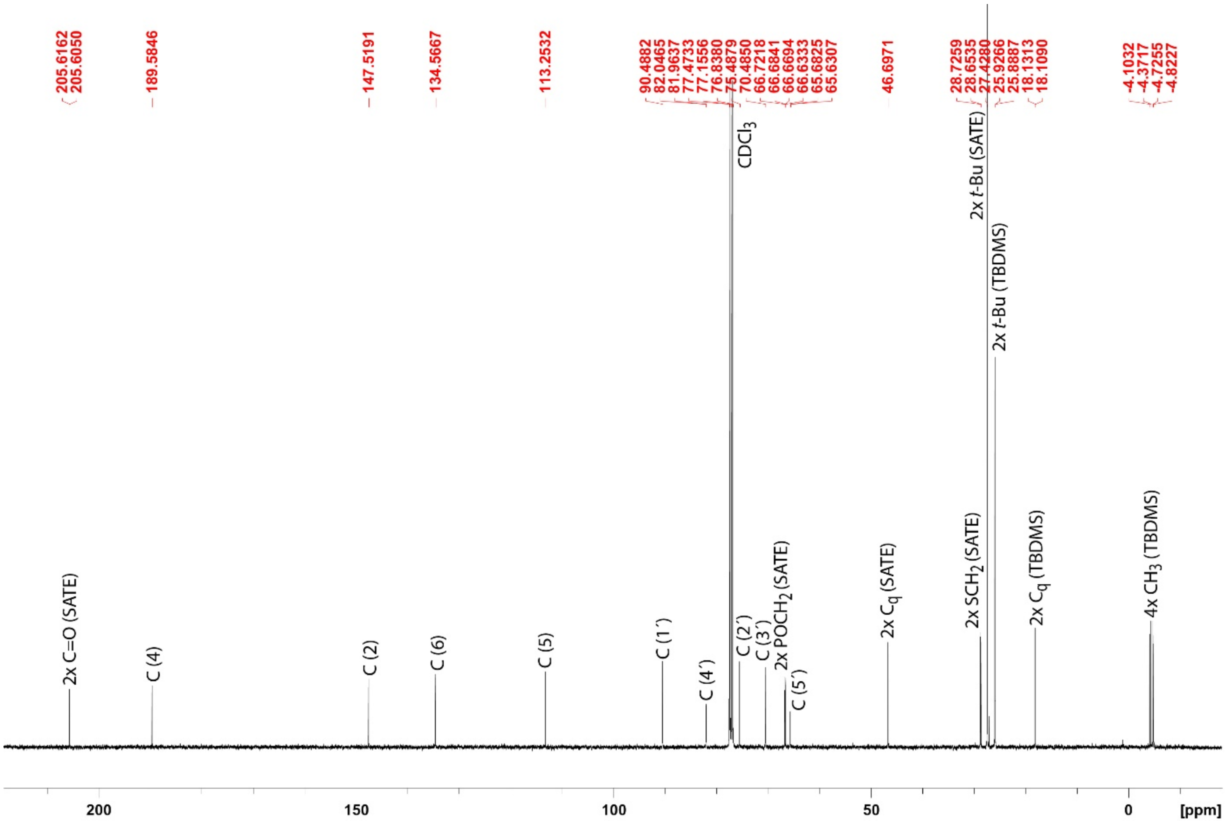

$^{31}$P-NMR (162 MHz, $CDCl_3$) of compound **7b**

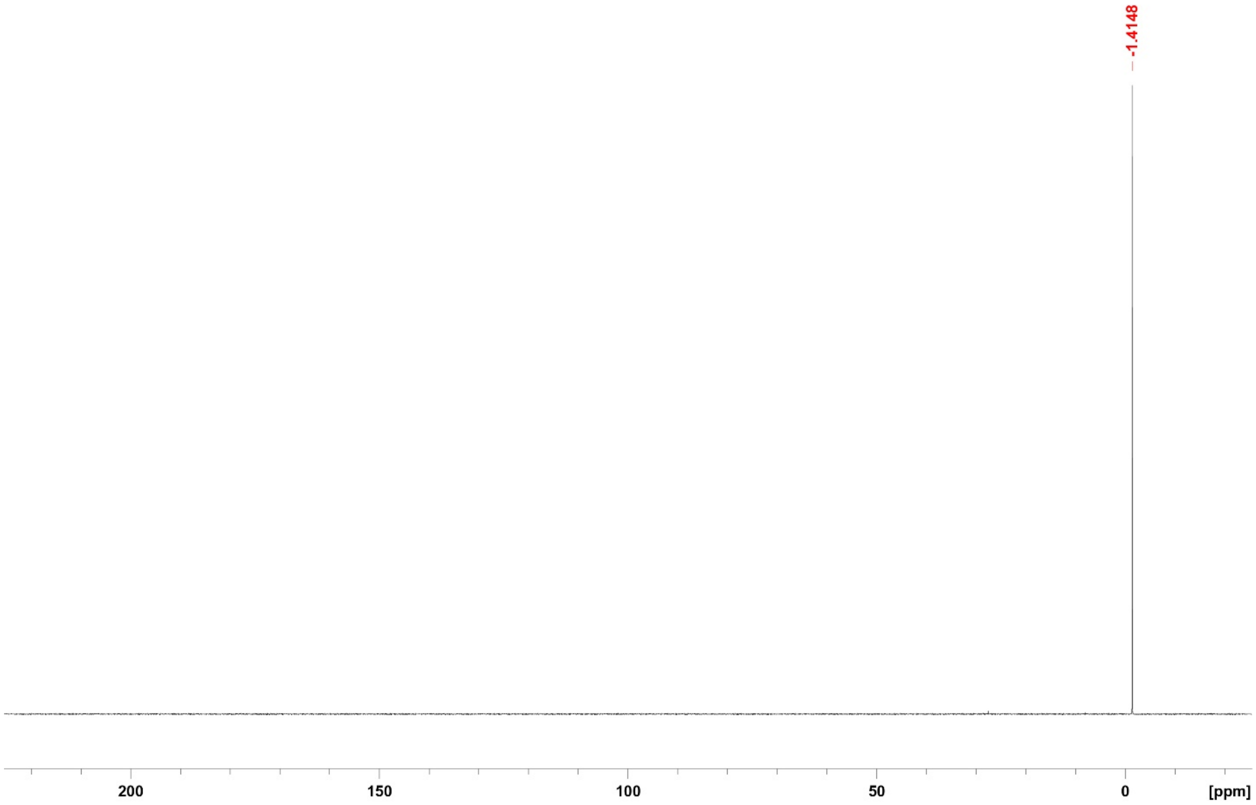

### 3.6. Synthesis of (4-acetoxybenzyl)-((9*H*-fluoren-9-yl)methyl)-5´-*O*-(2´,3´-di-*O*-(*tert*-butyldimethylsilyl)-4-thiouridinyl) phosphate (7c) [5,6]

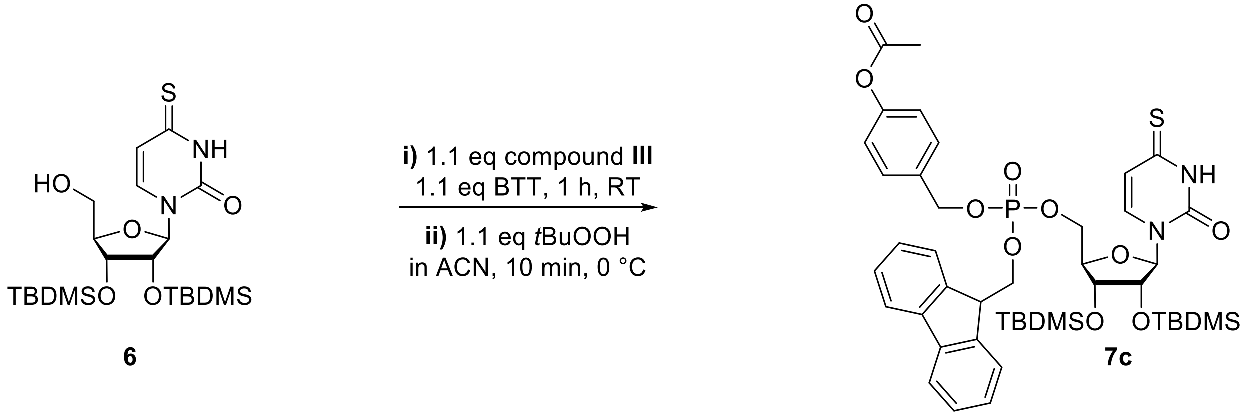


Compound **III** (1.1 eq, 0.24 mmol, 118 mg) and compound **6** (0.22 mmol, 107 mg) were coevaporated three times with acetonitrile and were subsequently dried under high vacuum for a few minutes. 5-(Benzylthio)-1*H*-tetrazole (0.3 M in acetonitrile; 1.1 eq, 0.80 ml, dried over molecular sieves) was added and the solution was stirred for 60 min at ambient temperature. It was then cooled to 0 °C and a solution of *tert*-butyl hydroperoxide in decane (0.48 ml 0.5 M in toluene, dried over molecular sieves) was added dropwise. The solution was stirred 5 min at 0 °C and further 5 min at ambient temperature. Solvent was removed under reduced pressure and the oily residue purified via column chromatography (10-30% ethyl acetate in c-hexane). Yield: 97 mg of **7c** as slightly yellow foam (45%). TLC (5% MeOH in $CH_2Cl_2$): 0.52 (2x diastereomers). HR-ESI-MS (m/z): $[M+H]^+$ calculated for $[C_{44}H_{60}N_2O_{10}PSi_2S]^+$: 895.3239; found: 895.3228. $^1$H-NMR ($CDCl_3$, 400 MHz): δ 0.012, 0.039, 0.043, 0.077, 0.080 (12 H, 5x s, 4x $CH_3$ (TBDMS)); 0.863, 0.864, 0.875, 0.880 (18 H, 4x s, 2x *t*-Bu (TBDMS)); 2.30 (3 H, s, $CH_3$ (AB)); 3.90-4.21 (6 H, m, $CH_2$ (5´), CH (4´), CH (3´), CH (2´), CH (Fmoc)); 4.35-4.49 (2 H, m, $CH_2$ (Fmoc)); 4.77-4.94 (2 H, m, $CH_2$ (AB)); 5.64 (0.5 H, d, $^3J_{HH}$ = 3.12 Hz, $CH_a$ (1´)); 5.66 (0.5 H, d, $^3J_{HH}$ = 3.12 Hz, $CH_b$ (1´)); 6.11 (0.5 H, dd, $^3J_{HH}$ = 7.67 Hz, $^4J_{HH}$ = 1.70 Hz, $CH_a$ (5)); 6.15 (0.5 H, dd, $^3J_{HH}$ = 7.67 Hz, $^4J_{HH}$ = 1.69 Hz, $CH_b$ (5)); 7.06-7.09 (2 H, m, aromat. CH (AB)); 7.26-7.35 (5 H, m, aromat CH (AB + Fmoc), CH (6)); 7.37-7.44, 7.52-7.58, 7.73-7.78 (6 H, m, aromat. CH (Fmoc)); 9.32 (1 H, s, NH) ppm. $^{13}$C-NMR ($CDCl_3$, 101 MHz): δ -4.88, -4.75, -4.44, -4.16 4x $CH_3$ (TBDMS); 18.10 2x $C_q$ (TBDMS); 21.27 $CH_3$ (AB); 25.87, 25.90 2x *t*-Bu (TBDMS); 48.08-48.19 (2x d, CH (Fmoc)); 65.24-65.43 (2x d, C (5´)); 69.04-69.09 (2xd, $CH_2$ (AB)); 69.53 (d, $^2J_{CP}$ = 5.82 Hz, $CH_2$ (Fmoc)); 70.40, 70.45 C (3´); 75.50 C (2´); 81.89, 81.97 C (4´); 90.27, 90.31 C (1´); 113.02, 113.05 C (5); 120.29, 120.33, 120.35, 122.12, 124.93, 124.98, 125.00, 125.08, 127.36, 127.39, 127.42, 127.47, 128.20, 128.25, 129.36, 129.39, 132.98, 133.05 aromat C (AB + Fmoc); 134.32, 134.39 C (6); 141.59-141.71 (2x d), 142.84, 142.90 aromat C (AB + Fmoc); 147.36 C (2); 151.14 aromat. C (AB); 169.39 C=O (AB); 189.41, 189.44 C (4) ppm. $^{31}$P-NMR ($CDCl_3$, 162 MHz): δ -0.89, -0.82 ppm (2 diastereomers).

$^{13}$C-NMR note: multiplicity of aromatic signals is not assignable and the corresponding signals are therefore listed. $CH_2$ (Fmoc) appear as doublet which may derive from $^2J_{CP}$ coupling or emerge from the diastereomeric nature of the molecule. Since an overlay of diastereomeric signals is more likely than an overlay of a $^2J_{CP}$ coupling, we classified this signal as doublet with a coupling constant of 5.82 Hz.

$^{1}$H-NMR (400 MHz, $CDCl_3$) of compound **7c**

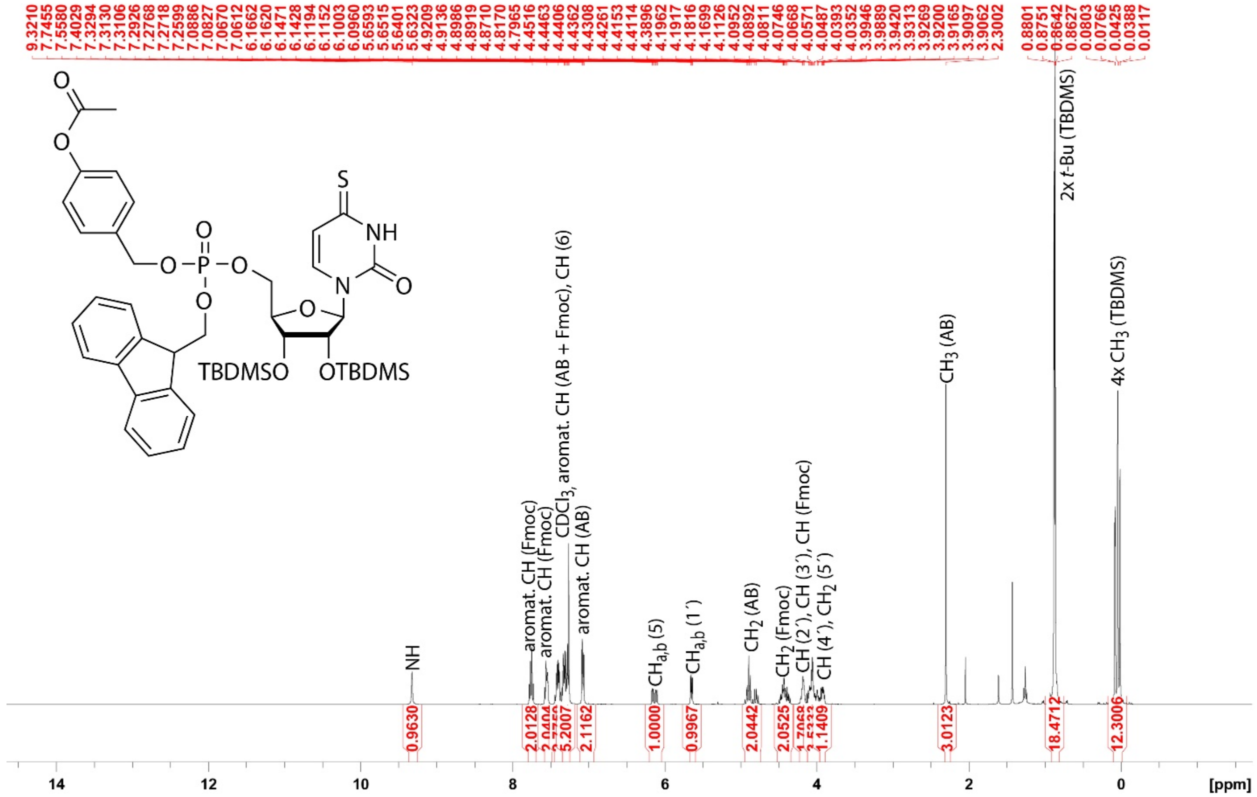

$^{13}$C-NMR (101 MHz, $CDCl_3$) of compound **7c**

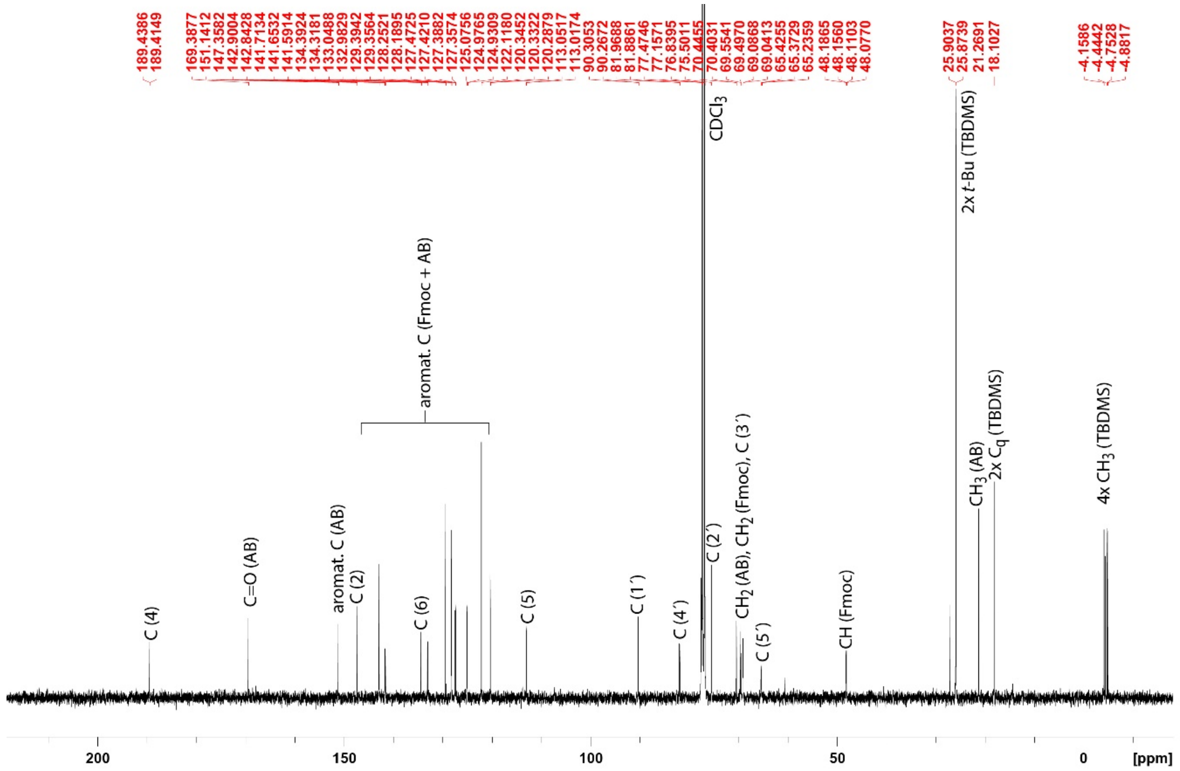

$^{31}$P-NMR (162 MHz, $CDCl_3$) of compound **7c**

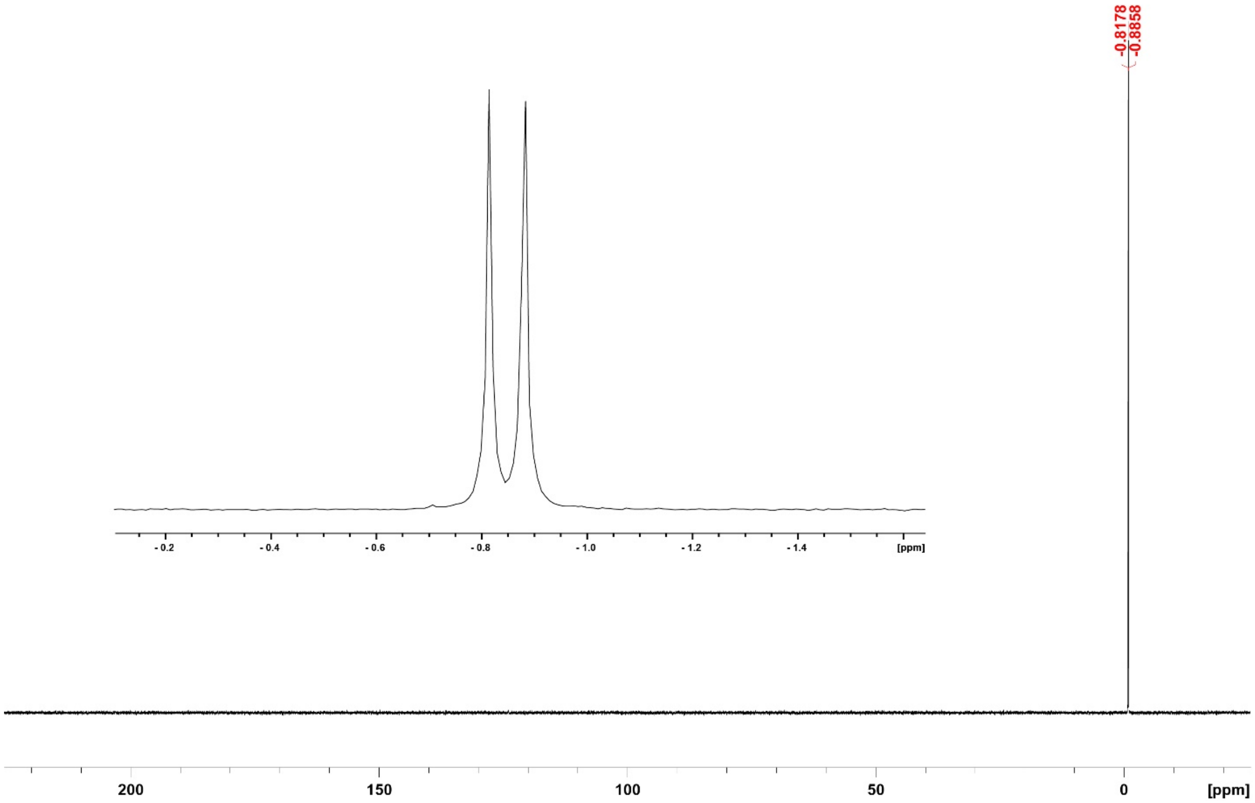

### 3.7. Synthesis of bis(4-acetoxybenzyl)-5´-*O*-(4-thiouridinyl) phosphate ((AB)$_2$-4sU, 8a) [7]

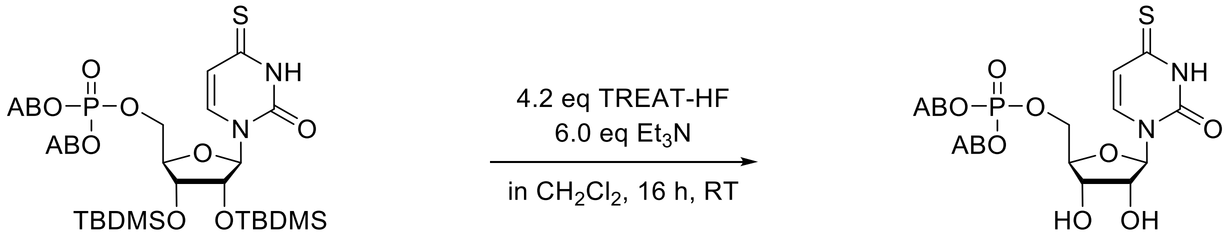


A solution of compound **7a** (33 mg, 0.038 mmol) in dichloromethane (0.40 ml) was treated with triethylamine trihydrofluoride (4.2 eq, 0.16 mmol, 26 µl) and triethylamine (6.0 eq, 0.23 mmol, 32 µl). The reaction mixture was allowed to stir 16 h at ambient temperature followed by evaporation of the solvent and flash chromatography (2-5% MeOH in $CH_2Cl_2$). Yield: 12 mg of **8a** as a yellow oil (49%). TLC (5% MeOH in $CH_2Cl_2$): 0.37. HR-ESI-MS (m/z): $[M+H]^+$ calculated for $[C_{27}H_{30}N_2O_{12}PS]^+$: 637.1252; found: 637.1233. $^1$H-NMR ($CDCl_3$, 300 MHz): δ 2.30 (6 H, 2x s, Acetyl (AB)); 3.91-3.99 (2 H, m, CH (3´), CH (2´)); 4.03-4.20 (3 H, m, $CH_2$ (5´), CH (4´)); 4.95-5.12 (4 H, m, $CH_2$ (AB)); 5.68 (1 H, d, $^3J_{HH}$ = 4.02 Hz, CH (1´)); 6.27 (1 H, d, $^3J_{HH}$ = 7.65 Hz, CH (5)); 7.09 (4 H, dd, $^3J_{HH}$ = 8.48 Hz, $^4J_{HH}$ = 3.41 Hz, aromat. CH (AB)); 7.24 (1 H, d, $^3J_{HH}$ = 7.70 Hz, CH (6)); 7.37 (4 H, d, $^3J_{HH}$ = 8.40 Hz, aromat. CH (AB)) ppm. $^{13}$C-NMR ($CDCl_3$, 101 MHz): δ 21.29 2x Acetyl (AB); 66.44 (d, $^2J_{CP}$ = 5.08 Hz, CH (5´)); 69.43 (d, $^2J_{CP}$ = 5.09 Hz, 2x $CH_2$ (AB)); 70.29 C (3´); 75.01 C (2´); 83.28 (d, $^3J_{CP}$ = 8.06 Hz, CH (4´)); 91.35 C (1´); 113.57 C (5); 122.24, 122.26, 129.61, 129.73 aromat. C (AB), 133.14-133.27 (2x d, aromat. $C_q$ (AB)); 134.71 C (6); 148.27 C (2); 151.09, 151.12 aromat. $C_q$ (AB); 169.95, 170.05 2x C=O (AB); 189.66 C (4) ppm. $^{31}$P-NMR ($CDCl_3$, 162 MHz): δ -0.67 ppm.

$^{1}$H-NMR (300 MHz, $CDCl_3$) of compound **8a**

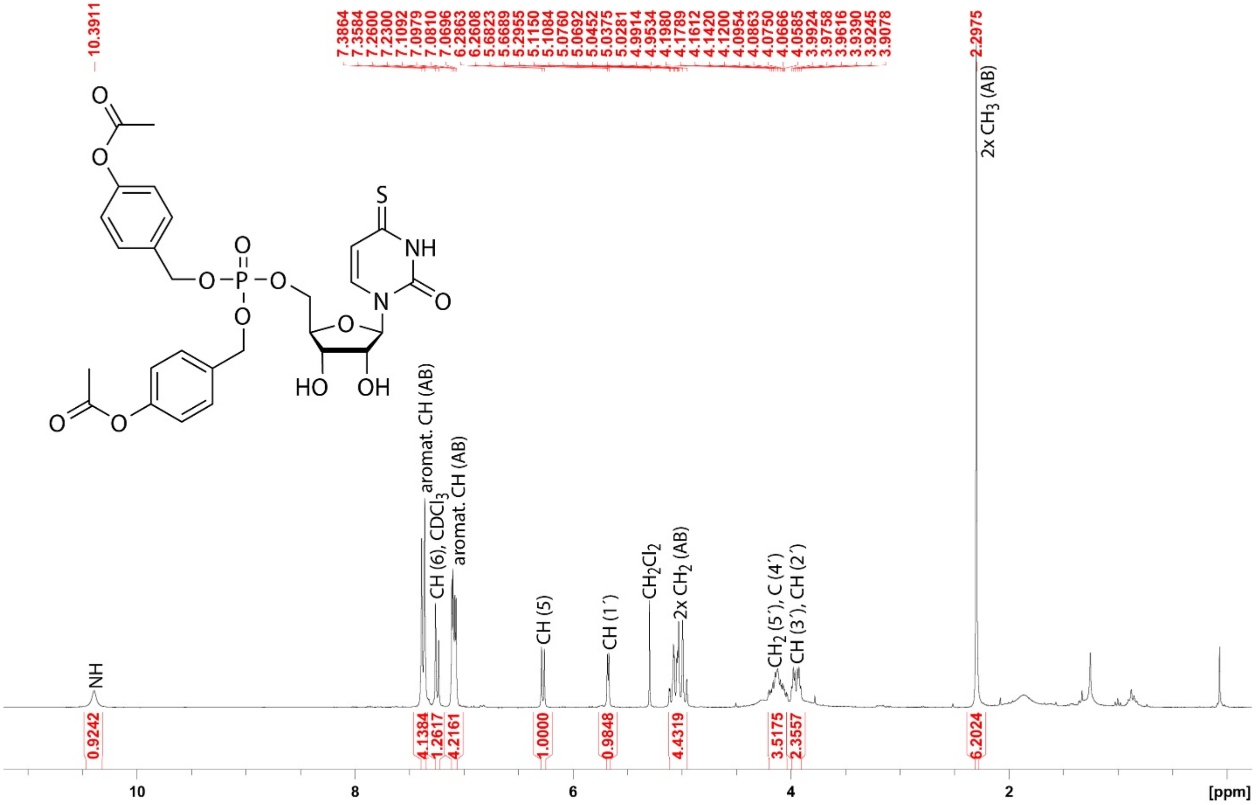


$^{13}$C-NMR (101 MHz, $CDCl_3$) of compound **8a**

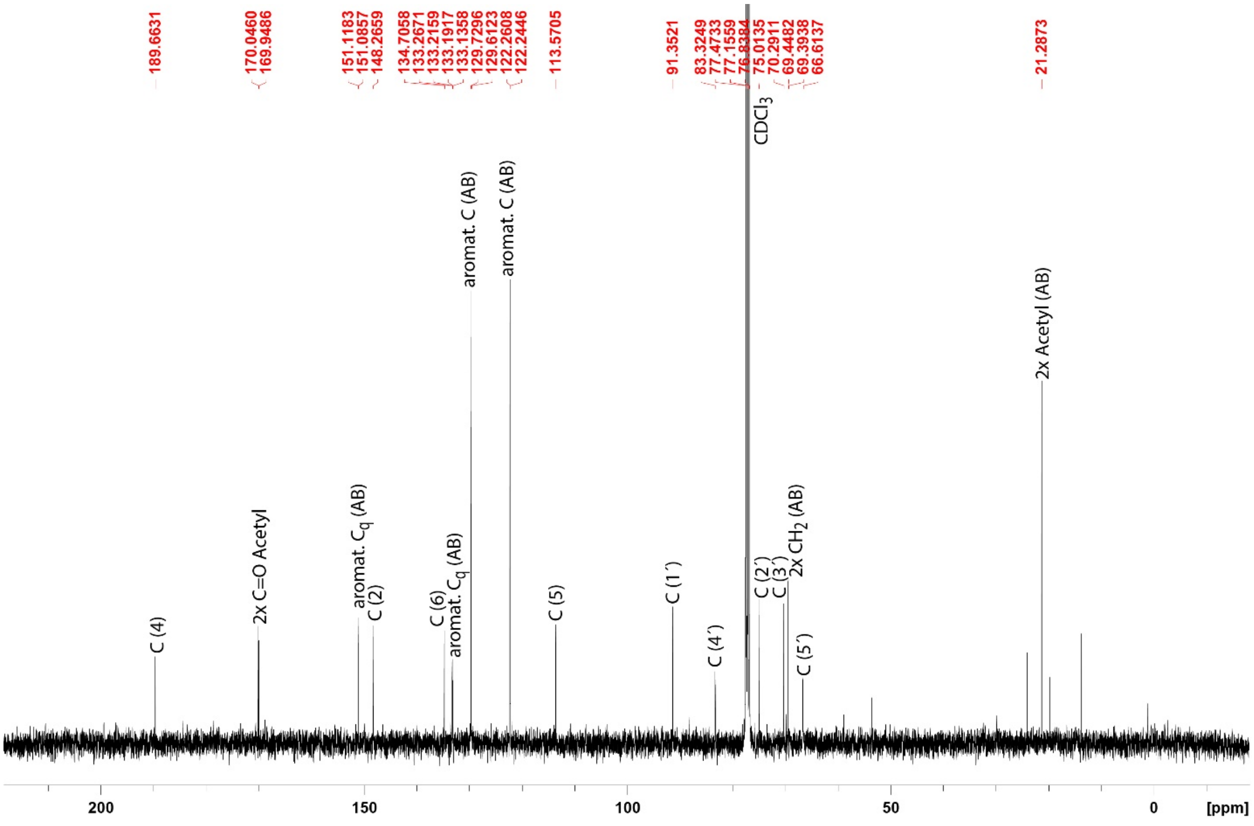

$^{31}$P-NMR (162 MHz, $CDCl_3$) of compound **8a**

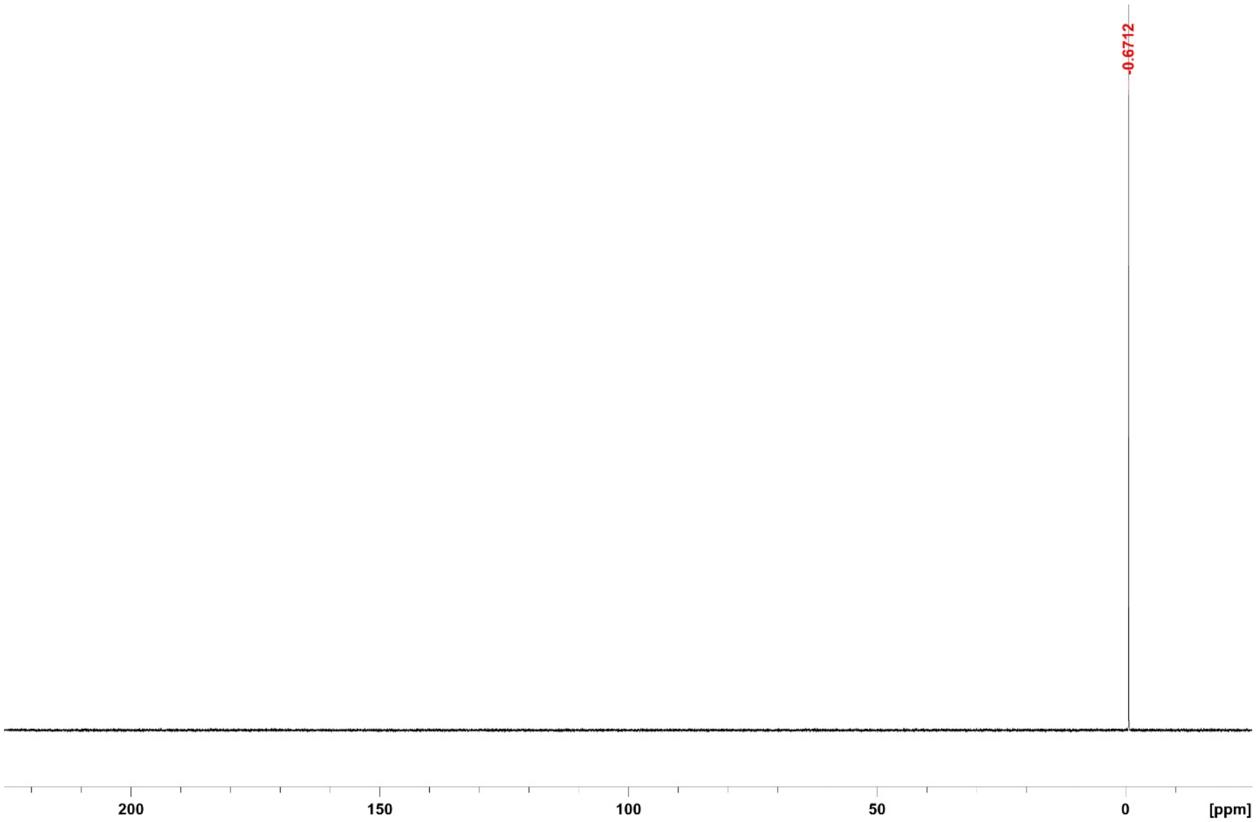

### 3.8. Synthesis of bis(*S*-pivaloyl-2-thioethyl)-5´-*O*-(4-thiouridinyl) phosphate ((*t*BuSATE)$_2$-4sU, 8b)

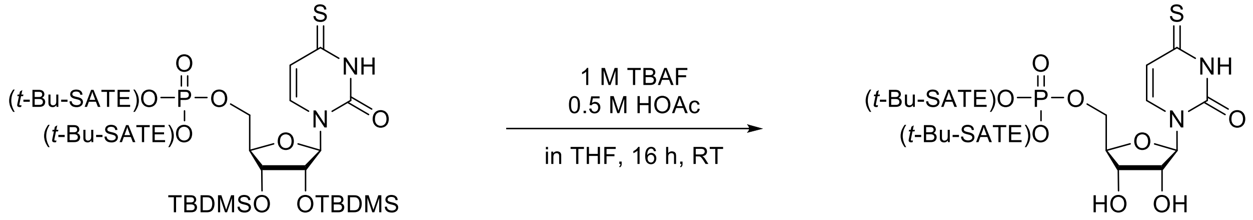


**7b** **8b**

A solution of tetrabutylammonium fluoride trihydrate (1.0 M) and acetic acid (0.5 M) in tetrahydrofuran (0.8 ml) was added to compound **7b** (43 mg, 0.50 mmol) and the reaction mixture was stirred at ambient temperature for 16 h. It was then diluted with dichloromethane, washed with saturated sodium bicarbonate solution and dried over sodium sulfate. Solvents were evaporated and the crude product was purified by flash chromatography (2-3% MeOH in $CH_2Cl_2$). Yield: 16 mg of **8b** as yellow oil (51%). TLC (5% MeOH in $CH_2Cl_2$): 0.39. HR-ESI-MS (m/z): $[M+Na]^+$ calculated for $[C_{23}H_{38}N_2O_{10}PS_3Na]^+$: 651.1240; found: 651.1216. $^1$H-NMR ($CDCl_3$, 400 MHz): 1.22, 1.23 (2x 9 H, 2x s, 2x *t*-Bu (SATE)); 3.11-3.16 (4 H, m, 2x $SCH_2$ (SATE)); 4.09-4.16 (4 H, m, 2x $POCH_2$ (SATE)); 4.25 (1 H, m, CH (4´)); 4.29-4.38 (4 H, m, $CH_2$ (5´), CH (2´), CH (3´)); 5.77 (1 H, d, $^3J_{HH}$ = 3.52 Hz, CH (1´)); 6.44 (1 H, d, $^3J_{HH}$ = 7.64 Hz, CH (5)); 7.41 (1 H, d, $^3J_{HH}$ = 7.64 Hz, CH (6)) ppm. $^{13}$C-NMR ($CDCl_3$, 101 MHz): δ 27.45 2x *t*-Bu (SATE); 28.52-28.64 (2x d, 2x $SCH_2$ (SATE)); 46.73 2x $C_q$ (SATE); 66.73-66.80 (m, $POCH_2$ (SATE), C (5´)); 69.80 C (3´); 74.83 C (2´); 82.80 ($^3J_{CP}$ = 7.26 Hz, C (4´)); 91.70 C (1´); 113.90 C (5); 134.98 C (6); 148.59 C (2); 189.92 C (4); 206.03, 206.13 2x C=O (SATE) ppm. $^{31}$P-NMR ($CDCl_3$, 162 MHz): δ -1.84 ppm.

$^{1}$H-NMR (400 MHz, $CDCl_3$) of compound **8b**

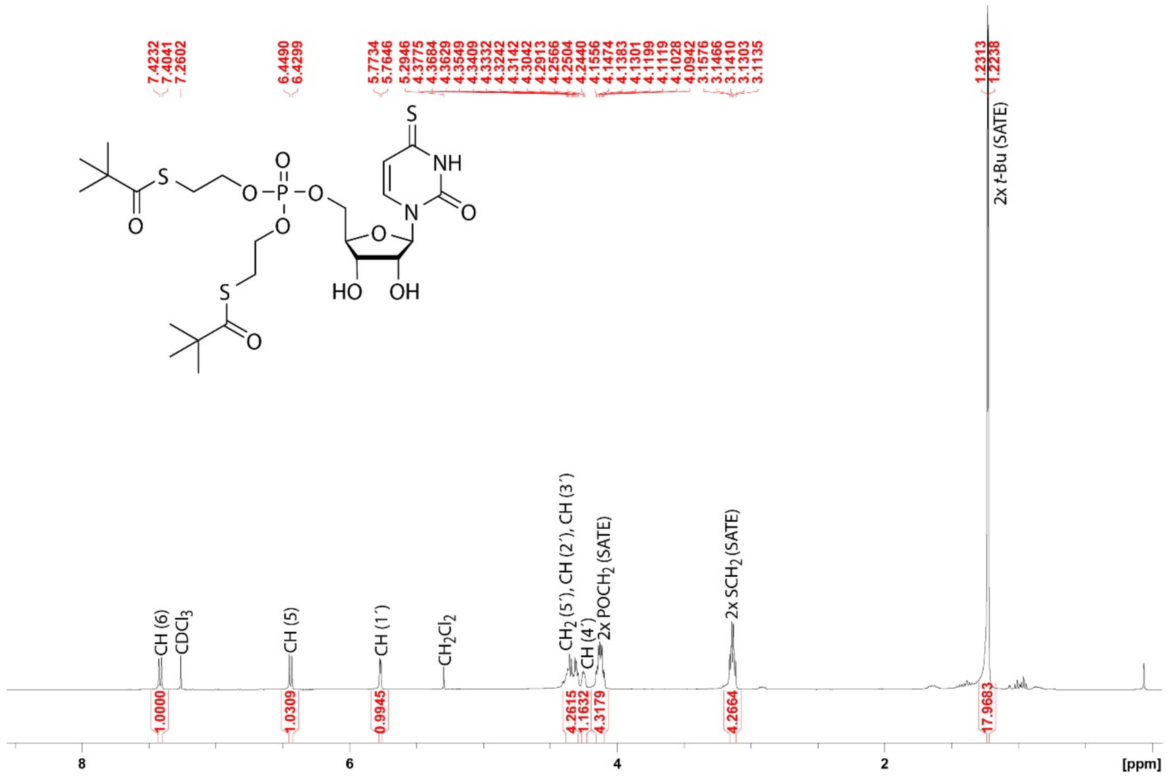


$^{13}$C-NMR (101 MHz, $CDCl_3$) of compound **8b**

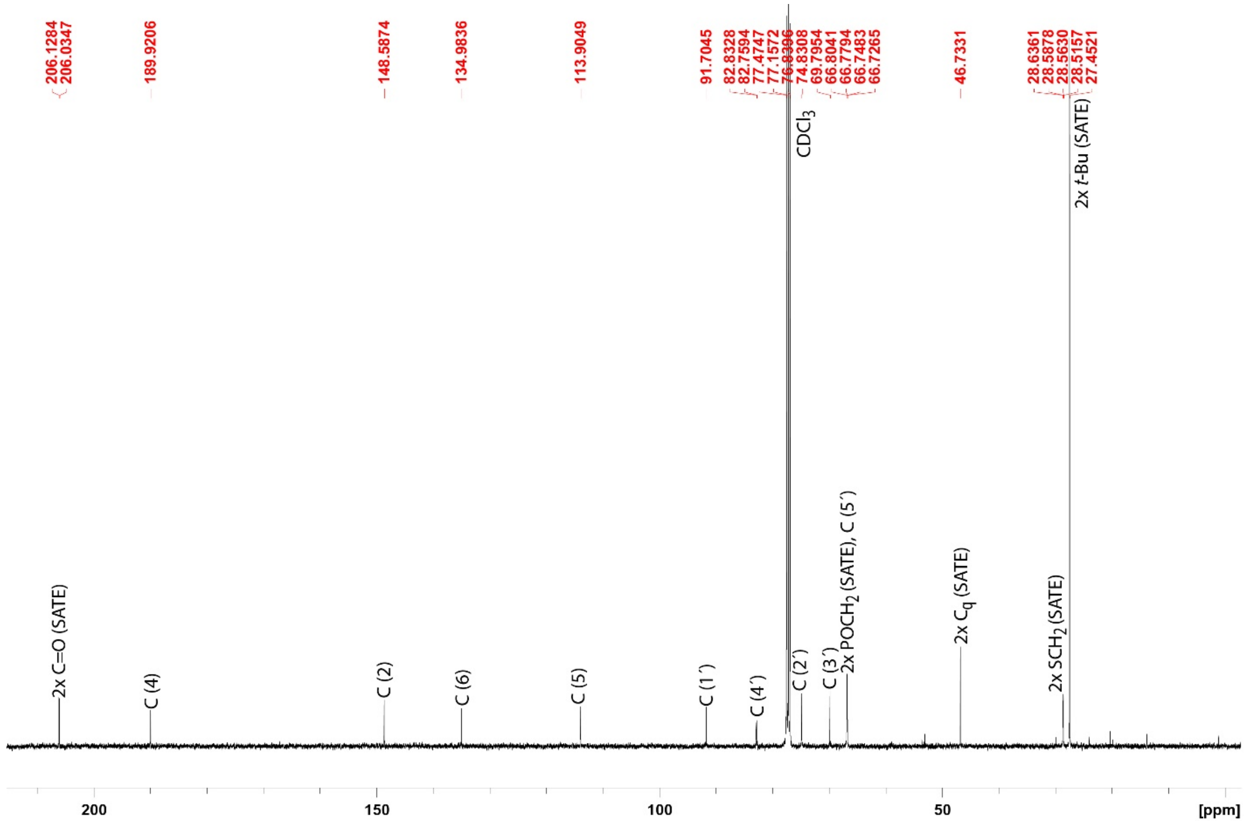

$^{31}P$-NMR (162 MHz, $CDCl_3$) of compound **8b**

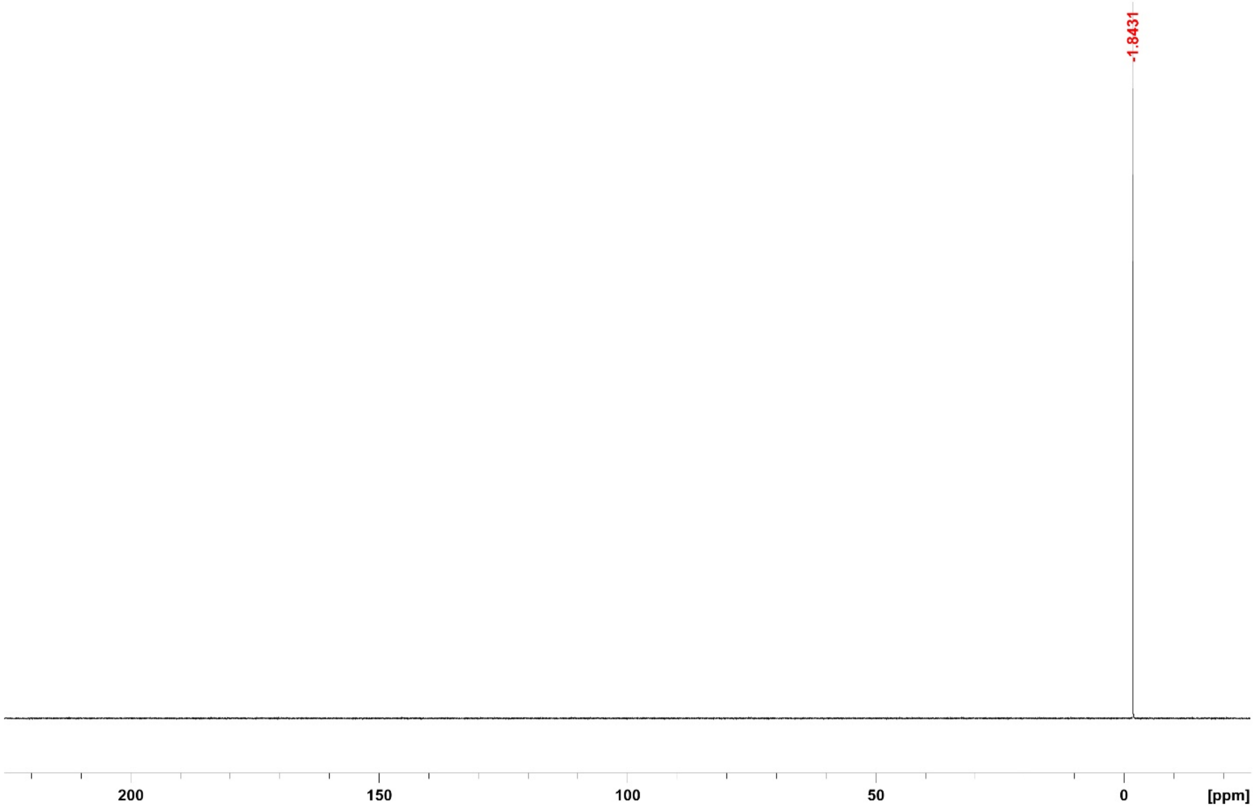

**3.9. Synthesis of (4-acetoxybenzyl)-5´-*O*-(4-thiouridinyl) phosphate triethylammonium salt (AB-4sU, 8c)** [7]

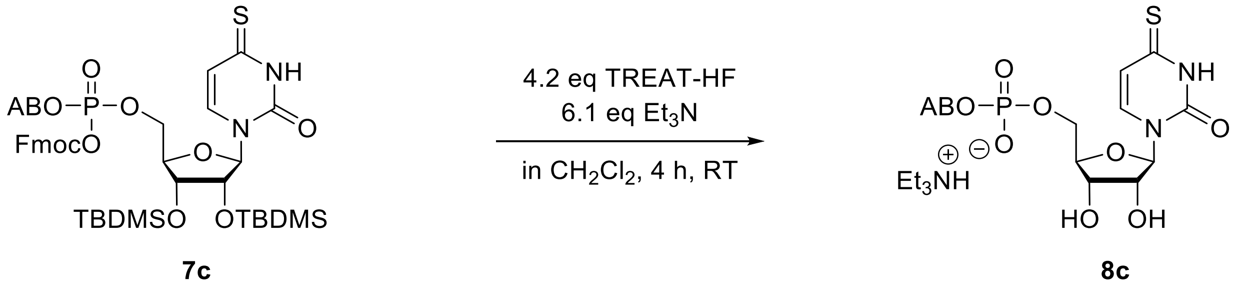


A solution of compound **7c** (74 mg, 0.083 mmol) in dichloromethane (1.0 ml) was treated with triethylamine trihydrofluoride (4.2 eq, 0.35 mmol, 57 µl) and triethylamine (6.1 eq, 0.50 mmol, 70 µl). Reaction was deemed complete after 4 h upon which solvent was evaporated. The yellow oil was dried under high vacuum overnight to remove the excess of triethylamine as good as possible. The oil was then dissolved in dichloromethane and treated with diethyl ether until a white precipitate appears. The suspension was transferred into Eppendorf tubes which were centrifuged for 5 min at 13 000 rpm. The clear supernatant was discarded and the yellow oil was redissolved in a minimal amount of (warm) dichloromethane. Diethyl ether was added and the white suspension was intensively vortexted. Centrifugation delivered again a clear supernatant, which was discarded. This process was repeated twice after which compound **8c** was obtained as its triethylammonium salt. Yield: 48 mg of **8c** as slightly yellow oil (99%). TLC (10% MeOH in $CH_2Cl_2$): 0.19. HR-ESI-MS (m/z): $[M]^-$ calculated for $[C_{18}H_{20}N_2O_{10}PS]^-$: 487.0571; found: 487.0584. $^1$H-NMR (CDCl$_3$, 400 MHz): δ 1.23 (9 H, t, $^3J_{HH}$ = 7.04 Hz, 3x $CH_3$ ($Et_3N$)); 2.25 (3 H, s, $CH_3$ (AB)); 3.00 (6 H, q, $^3J_{HH}$ = 6.72 Hz, 3x $CH_2$ ($Et_3N$)); 4.07 (2 H, m br., $CH_2$ (5´)); 4.16 (1 H, m br., CH (4´)); 4.24 (2 H, m br., CH (2´), CH (3´), 4.89 (2 H, m, $CH_2$ (AB)); 5.88 (1 H, d, $^3J_{HH}$ = 3.00 Hz, CH (1´)); 6.35 (1 H, d, $^3J_{HH}$ = 7.52 Hz, CH (5)); 6.99 (2 H, d, $^3J_{HH}$ =8.32 Hz, aromat. CH (AB)); 7.37 (2 H, d, $^3J_{HH}$ =8.32 Hz, aromat. CH (AB)); 7.68 (1 H, d, $^3J_{HH}$ = 7.49 Hz, CH (6)); 10.45 (2 H, s, 2x NH) ppm. $^{13}$C-NMR (CDCl$_3$, 101 MHz): δ 8.62 3x $CH_3$ $Et_3N$; 21.23 Acetyl (AB); 46.04 3x $CH_2$ $Et_3N$; 65.08 (m, C (5´)); 66.95 (d, $^2J_{CP}$ = 5.13 Hz, $CH_2$ (AB)); 70.59 C (3´); 74.97 C (2); 84.00 (d, $^3J_{CP}$ = 7.51 Hz, C (4´)); 89.61 C (1´); 113.47 C (5); 121.58, 128.77 aromat. C (AB); 135.92 C (6); 136.16 (d, $^3J_{CP}$ = 7.27 Hz, $C_q$ (AB)); 148.31 C (2); 150.15 $C_q$ (AB); 169.71 C=O (AB); 190.14 C (4) ppm. $^{31}$P-NMR (CDCl$_3$, 162 MHz): δ -0.95 ppm.

$^{1}$H-NMR (400 MHz, $CDCl_3$) of compound **8c**

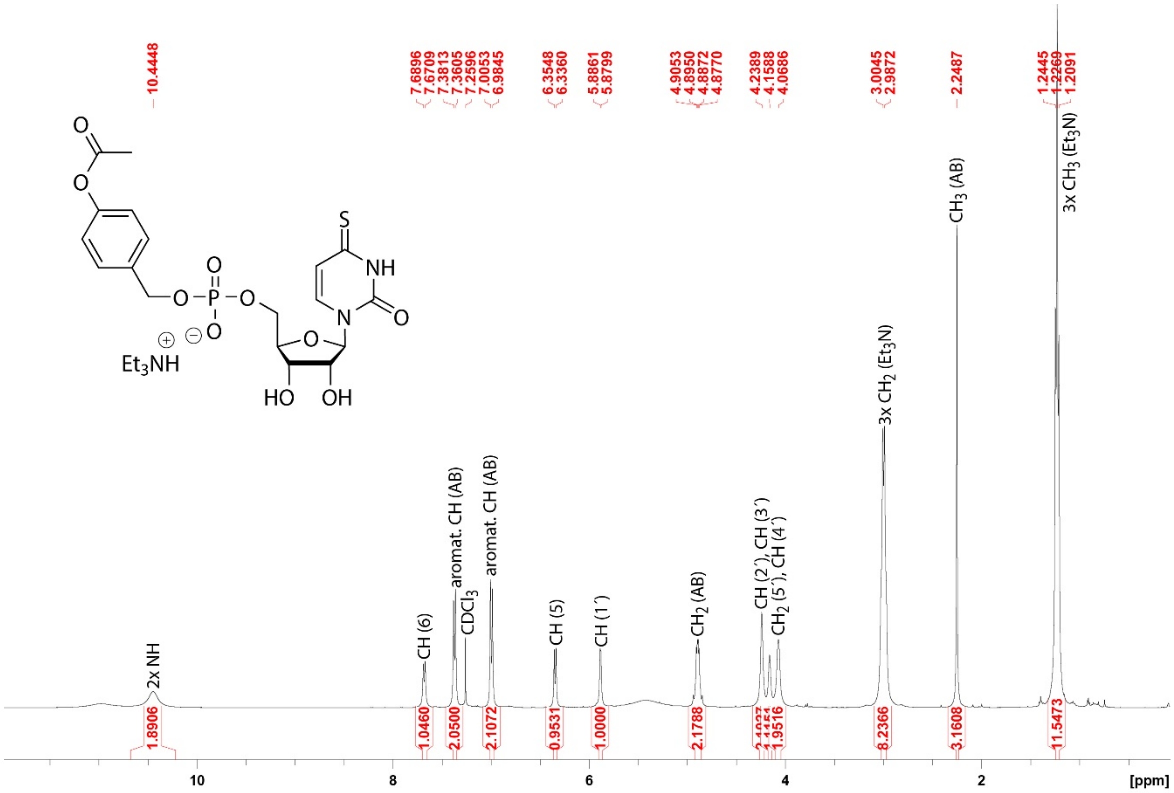


$^{13}$C-NMR (101 MHz, $CDCl_3$) of compound **8c**

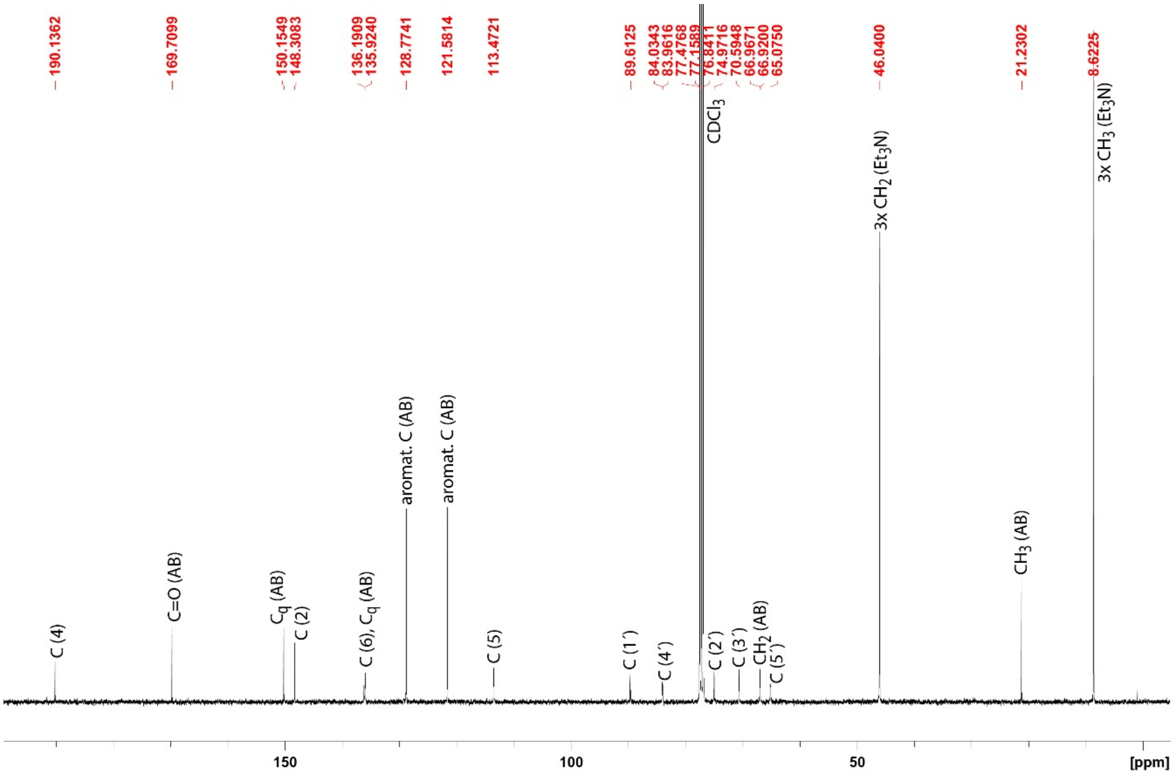

$^{31}$P-NMR (162 MHz, $CDCl_3$) of compound **8c**

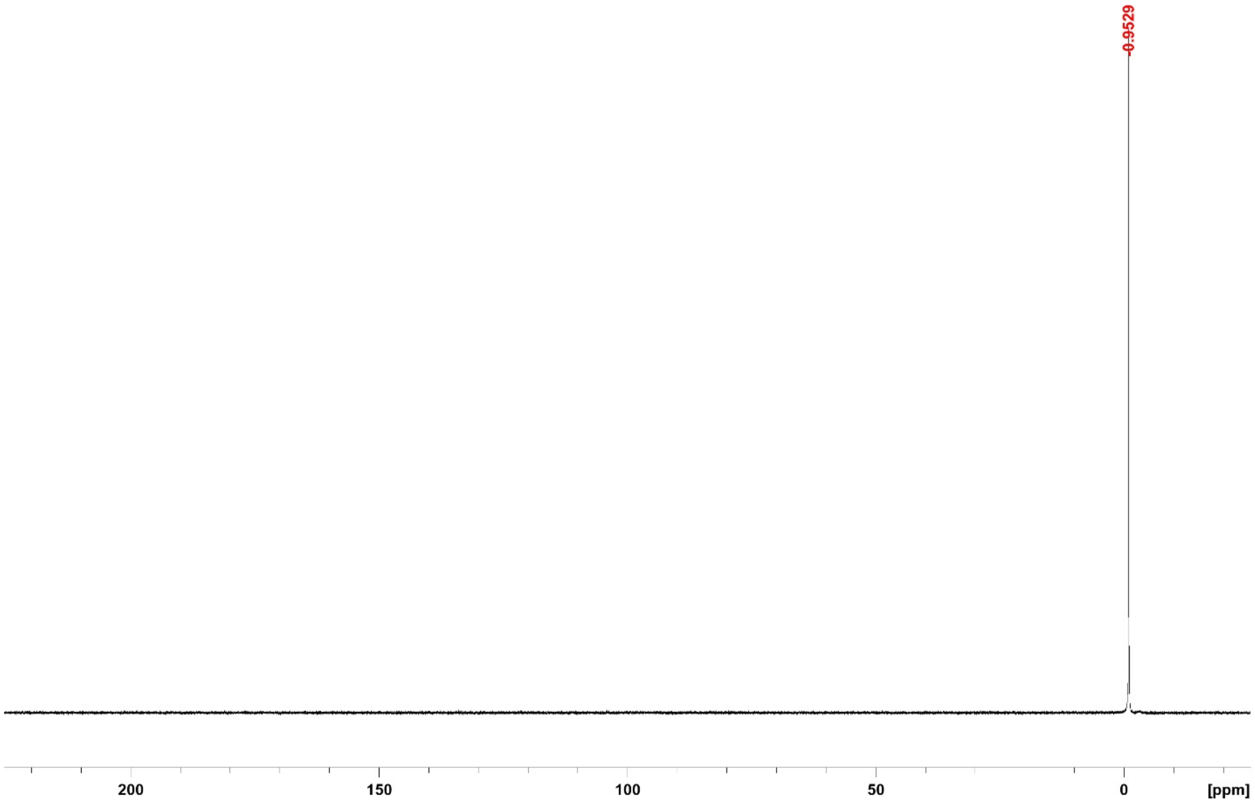

**4. Synthesis of CycloSal-4sU, Cl-CycloSal-4sU and ProTide-4sU (12a-c)**

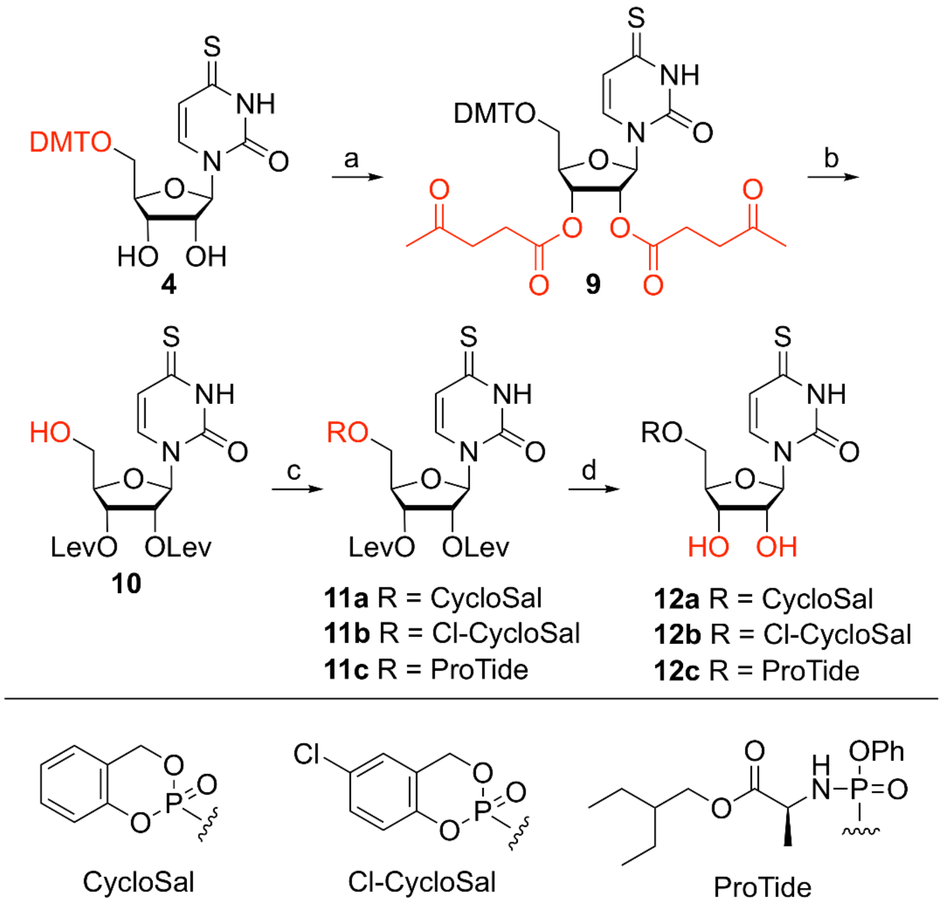


**Overview of the synthesis of CycloSal-4sU 12a, Cl-CycloSal-4sU 12b, and ProTide-4sU 12c**. Reaction conditions: **a)** i) 8.7 eq LevOH, 4.3 eq dicyclohexylcarbodiimide (DCC), $CH_2Cl_2$, 10 min, 0 °C; ii) 0.16 eq DMAP, 1.5 h, room temperature (RT), 97%. **b)** 1.5 eq benzenesulfonic acid, $CH_2Cl_2$/MeOH 9/1, 10 min, 0 °C to RT, 81%. **c)** for **11a**: i) 1.1 eq compound **IV**, 1.1 eq 5-benzylthio-1*H*-tetrazole (BTT), acetonitrile, 1 h, RT; ii) 1.1 eq *t*BuOOH, 10 min, 0 °C to RT, 72%; for **11b**: i) 0.9 eq compound **V**, 0.9 eq BTT, acetonitrile, 45 min, RT; ii) 1.0 eq *t*BuOOH, 10 min, 0 °C to RT, 54%; for **11c**: i) 1.0 eq compound **VI**, 1.0 eq $C_6H_5OP(O)Cl_2$, 2.0 eq $Et_3N$, $CH_2Cl_2$, 16 h, -78 °C to RT; ii) 5.0 eq phosphoryl chloride of i), 8.0 eq NMI, $CH_2Cl_2$, 20 h, 0 °C to RT, 69%. **d)** $H_2NNH_2$ in pyridine/HOAc, pyridine, 5 to 15 min, RT to 0 °C, **12a** (53%), **12b** (33%), **12c** (83%). Overall yield: **12a** (30%), **12b** (14%), **12c** (45%).

**4.1. Synthesis of 2´,3´-di-*O*-levulinoyl-5´-*O*-(4,4´-dimethoxytrityl)-4-thiouridine (9)** [8,9]

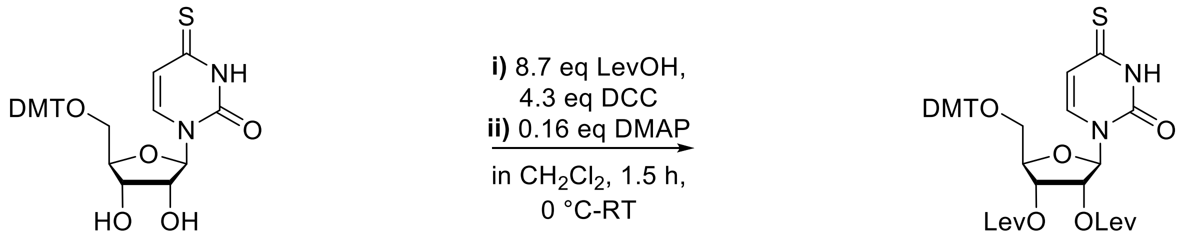


*N,N´*-Dicyclohexylcarbodiimid (4.3 eq, 5.827 g, 28.24 mmol) was dissolved in dichloromethane (45.0 ml) and cooled to 0 °C. Levulinic acid (8.7 eq, 57.57 mmol, 5.92 ml) was added and the reaction mixture was stirred for 10 min at ambient temperature and was then filtered into a flask containing compound **4** (3.705 g, 6.59 mmol) and 4-(dimethylamino)pyridine (0.16 eq, 1.08 mmol, 132 mg). The filter cake was washed with dichloromethane (15 ml) and the resulting solution was stirred 1.5 h at ambient temperature followed by extraction with saturated sodium bicarbonate solution and water. Drying over sodium sulfate and column chromatography (0-2% MeOH in $CH_2Cl_2$) delivered compound **9** as yellow foam. Yield: 4.840 g of **9** as yellow foam (97%). TLC (5% MeOH in $CH_2Cl_2$): 0.52. HR-ESI-MS (m/z): $[M+Na]^+$ calculated for $[C_{40}H_{42}N_2O_{11}SNa]^+$: 781.2402; found: 781.2383. $^1$H-NMR (CDCl3, 400 MHz): δ 2.162, 2.165 (6 H, s, 2x $CH_3$ (Lev)); 2.59-2.63, 2.72-2.78 (8 H, m, 4x $CH_2$ (Lev)); 3.43 (1 H, dd, $^2J_{HH}$ = 10.96 Hz, $^3J_{HH}$ = 2.25 Hz, $CH_a$ (5´)); 3.49 (1 H, dd, $^2J_{HH}$ = 11.03 Hz, $^3J_{HH}$ = 2.23 Hz, $CH_b$ (5´)); 3.77 (6 H, s, 2x $OCH_3$ (DMT)); 4.23 (1 H, dd, $^3J_{HH}$ = 4.42 Hz, $^3J_{HH}$ = 2.33 Hz, CH (4´)); 5.53-5.57 (2 H, m, CH (2´), CH (3´)); 5.93 (1 H, d, $^3J_{HH}$ = 7.67 Hz, CH (5)); 6.13 (1 H, d, $^3J_{HH}$ = 5.13 Hz, CH (1´)); 6.81-6.83, 7.19-7.35 (13 H, m, aromat. CH (DMT)); 7.49 (1 H, d, $^3J_{HH}$ = 7.71 Hz, CH (6)); 9.52 (1 H, m, NH) ppm. $^{13}$C-NMR (CDCl3, 101 MHz): δ 27.66, 27.77 2x $CH_2$ (Lev); 29.91, 29.93 2x $CH_3$ (Lev); 37.81 2x $CH_2$ (Lev); 55.40 2x $OCH_3$ (DMT); 62.62 C (5´); 71.26 C (3´); 73.39 C (2´); 82.50 C (4´); 86.19 C (1´); 87.69, 113.54 aromat. C (DMT); 113.67 C(5); 127.40, 128.26, 130.23, 130.30 aromat C (DMT); 134.41 C (6); 134.78, 135.00, 143.96 aromat. C (DMT); 147.61 C (2); 158.89, 158.92 aromat. C (DMT); 171.67, 171.89 2x C=O (Lev); 189.79 C (4); 206.23, 206.29 2x C=O (Lev) ppm.

## $^{1}$H-NMR (400 MHz, $CDCl_3$) of compound **9**

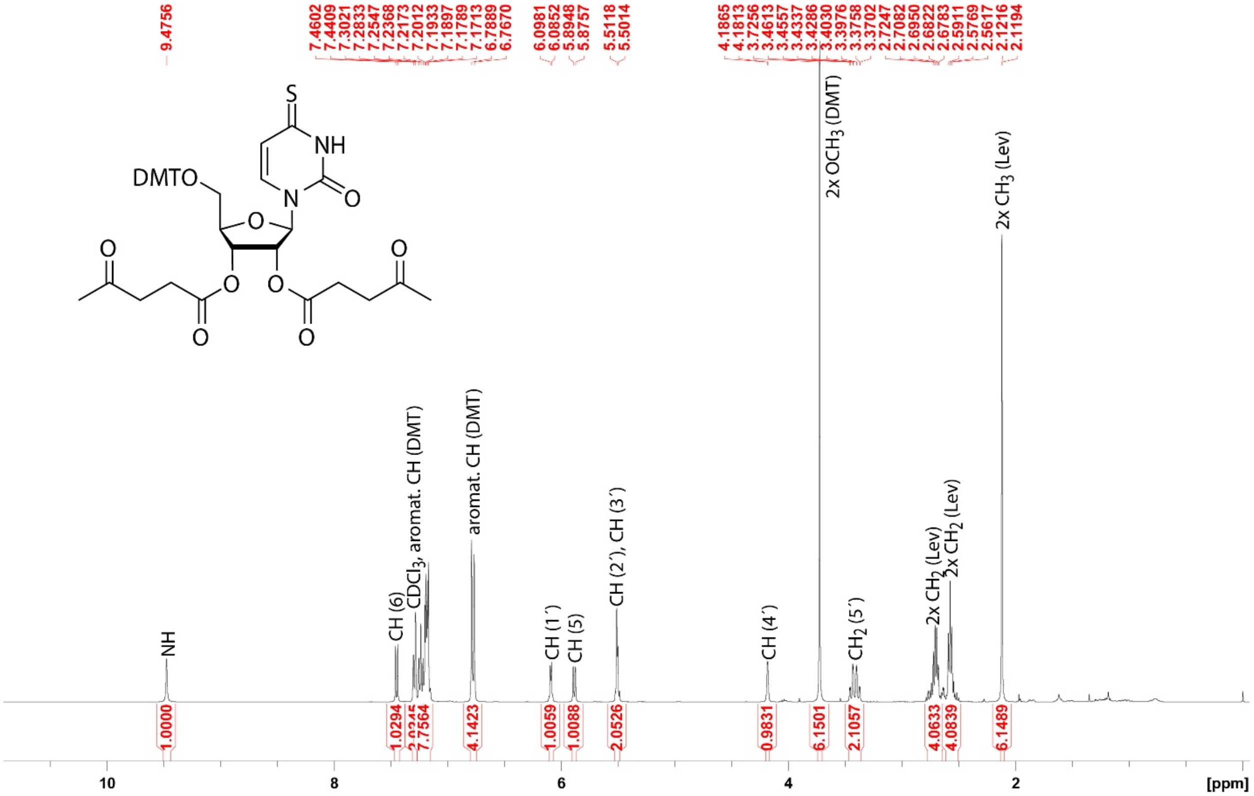


## $^{13}$C-NMR (101 MHz, $CDCl_3$) of compound **9**

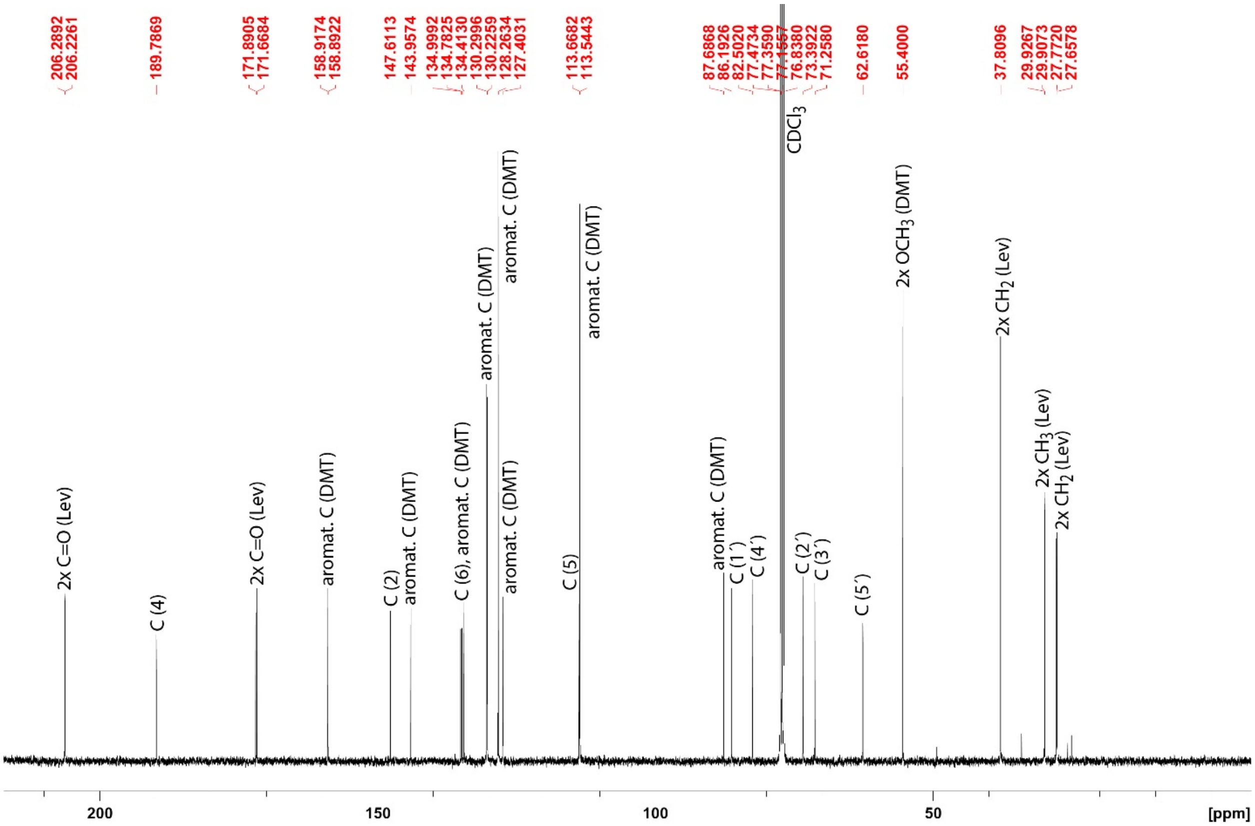

### 4.2. Synthesis of 2´,3´-di-*O*-levulinoyl-4-thiouridine (10)

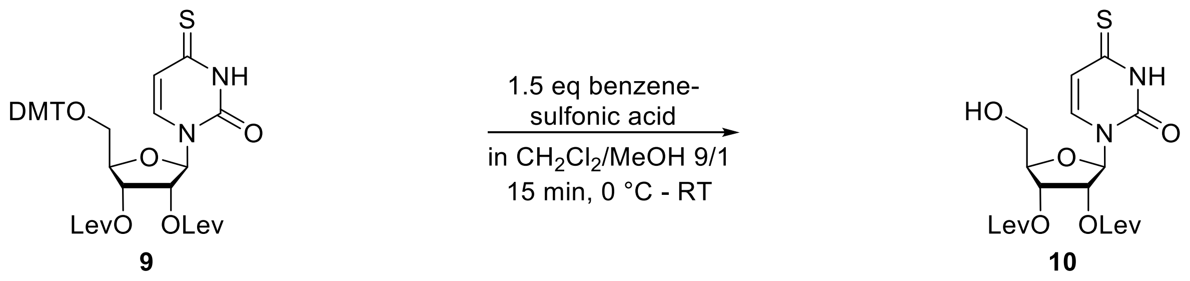


Compound **9** (588 mg, 0.77 mmol) was dissolved in a 9/1 mixture of dichloromethane and methanol (10.0 ml) and was cooled to 0 °C. A solution of benzenesulfonic acid (1.5 eq, 1.16 mmol, 184 mg) in the same solvent mixture (5.0 ml) was added and the bright orange solution was stirred for 10 min at 0 °C. Saturated sodium bicarbonate solution was added and the reaction mixture was stirred vigorously for 5 min. The organic phase was separated and the aqueous phase was washed two times with dichloromethane. The combined organic extracts were washed with saturated sodium bicarbonate solution and brine. Drying over sodium sulfate was followed by column chromatography (2-4% MeOH in $CH_2Cl_2$). Yield: 286 mg of **10** as yellow foam (81%). TLC (5% MeOH in $CH_2Cl_2$): 0.40. HR-ESI-MS (m/z): $[M+H]^+$ calculated for $[C_{19}H_{25}N_2O_9S]^+$: 457.1275; found: 457.1257. $^1$H-NMR (CDCl$_3$, 400 MHz): δ 2.17, 2.20 (2x 3 H, 2x s, 2x $CH_3$ (Lev)); 2.58-2.66, 2.73-2.81 (8 H, m, 4x $CH_2$ (Lev)); 3.85 (1 H, dd, $^2J_{HH}$ = 12.18 Hz, $^3J_{HH}$ = 1.66 Hz, $CH_a$ (5´)); 3.95 (1 H, dd, $^2J_{HH}$ = 12.26 Hz, $^3J_{HH}$ = 1.97 Hz, $CH_b$ (5´)); 4.24 (1 H, m, CH (4´)); 5.44-5.50 (2 H, m, CH (2´), CH (3´)); 5.93 (1 H, d, $^3J_{HH}$ = 5.38 Hz, CH (1´)); 6.61 (1 H, d, $^3J_{HH}$ = 7.64 Hz, CH (5)); 7.56 (1 H, d, $^3J_{HH}$ = 7.68 Hz, CH (6)); 9.67 (1 H, s, NH) ppm. $^{13}$C-NMR (CDCl$_3$, 101 MHz): δ 27.67, 27.80 2x $CH_2$ (Lev); 29.90, 29.94 2x $CH_3$ (Lev); 37.85, 37.88 2x $CH_2$ (Lev); 61.72 C (5´); 71.09 C (3´); 73.35 C (2´); 83.81 C (4´); 89.03 C (1´); 113.89 C(5); 135.63 C (6); 147.71 C (2); 171.86, 172.19 2x C=O (Lev); 189.91 C (4); 206.64, 206.72 2x C=O (Lev) ppm.

$^{1}$H-NMR (400 MHz, $CDCl_3$) of compound **10**

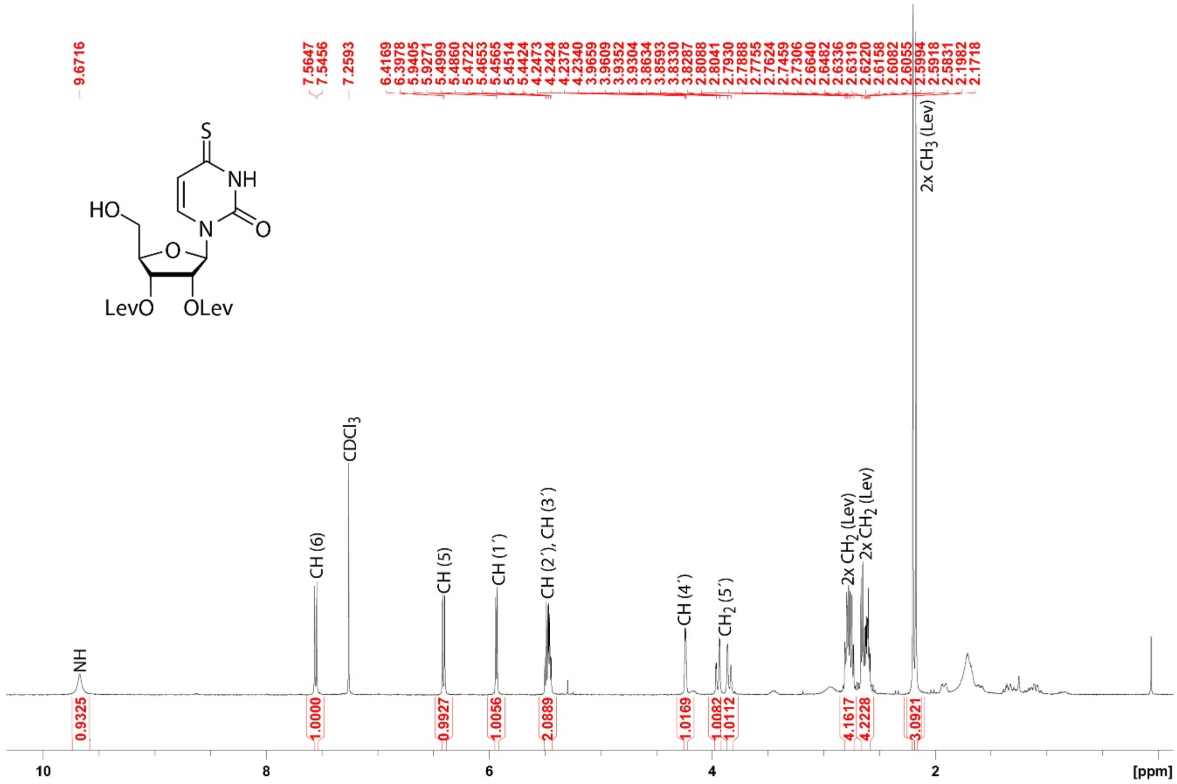

$^{13}$C-NMR (101 MHz, $CDCl_3$) of compound **10**

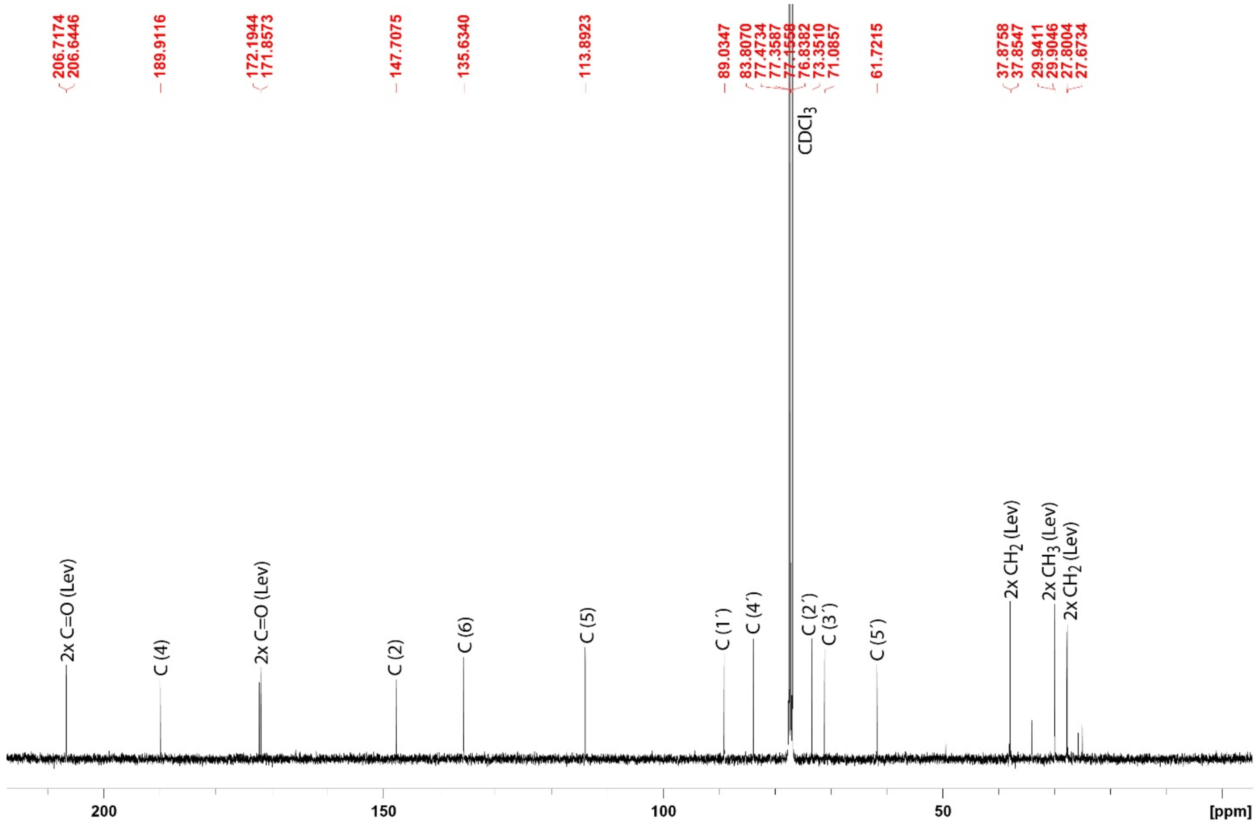

**4.3. Synthesis of cyclosaligenyl-5´-*O*-(2´,3´-di-*O*-levulinoyl-4-thiouridinyl) phosphate (11a)**

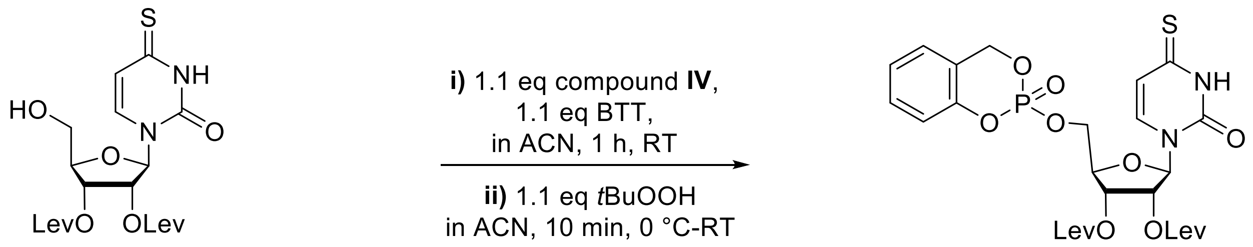


**10** **11a**

Compound **10** (71 mg, 0.15 mmol) was dissolved in a dry 5-(benzylthio)-1*H*-tetrazole solution (1.1 eq, 0.17 mmol, 0.57 ml 0.3 M in acetonitrile) and was subsequently treated with compound **IV** (1.1 eq, 0.17 mmol, 43 mg) in acetonitrile (0.6 ml). The reaction mixture was stirred 1 h at ambient temperature, was then cooled to 0 °C and t*ert*-butyl hydroperoxide in decane (1.1 eq, 0.17 mmol, 0.34 ml 0.5 M in toluene) was added dropwise. The yellow solution was allowed to stir 5 min at 0 °C and 5 min at room temperature, was then concentrated and purified by flash chromatography (0-3% MeOH in $CH_2Cl_2$). Yield: 70 mg of **11a** as yellow foam (72%). TLC (3% MeOH in $CH_2Cl_2$): 0.34. HR-ESI-MS (m/z): $[M+H]^+$ calculated for $[C_{26}H_{30}N_2O_{12}PS]^+$: 625.1252; found: 625.1247. $^1$H-NMR ($CDCl_3$, 400 MHz): δ 2.16, 2.17, 2.18 (2x 3 H, 2x s, 2x $CH_3$ (Lev)); 2.57-2.65, 2.71-2.80 (8 H, m, 4x $CH_2$ (Lev)); 4.33 (1 H, m, CH (4´)); 4.37-4.47, 4.49-4.53 (2 H, m, $CH_2$ (5´)); 5.23 (0.5 H, q, $^3J_{HH}$ = 5.21 Hz, $CH_a$ (2´)); 5.29 (1 H, m, $CH_b$ (2´), $CH_a$ (3´)); 5.33-5.41 (2.5 H, m, $CH_b$ (3´), $POCH_2$); 5.89 (0.5 H, d, $^3J_{HH}$ = 5.27 Hz, $CH_a$ (1´)); 5.92 (0.5 H, d, $^3J_{HH}$ = 5.27 Hz, $CH_b$ (1´)); 6.19 (0.5 H, d, $^3J_{HH}$ = 7.66 Hz, $CH_a$ (5)); 6.22 (0.5 H, d, $^3J_{HH}$ = 7.66 Hz, $CH_b$ (5)); 7.05-7.19 (4 H, m, $CH_{aryl}$ (3), $CH_{aryl}$ (6), $CH_{aryl}$ (5), CH (6)); 7.33 (1 H, m, $CH_{aryl}$ (4)); 9.76, 9.77 (1 H, s, $NH_{a,b}$) ppm. $^{13}$C-NMR ($CDCl_3$, 101 MHz): δ 27.61, 27.65 2x $CH_2$ (Lev); 29.85, 29.88 2x $CH_3$ (Lev); 37.80 2x $CH_2$ (Lev); 66.94 (d, $^2J_{CP}$ = 5.09 Hz, $C_a$ (5´)); 67.00 (d, $^2J_{CP}$ = 5.09 Hz, $C_b$ (5´)); 68.95-69.18 (2x d, $POCH_2$); 69.89, 70.04 C (3´); 72.90, 72.97 C (2´); 80.83 (d, $^3J_{CP}$ = 7.27 Hz, $C_a$ (4´)); 80.96 (d, $^3J_{CP}$ = 7.27 Hz, $C_b$ (4´)); 88.36, 88.58 C (1´); 113.87, 113.92 C (5); 118.68-118.95 (2x d, $C_{aryl}$ (3)); 120.50-120.80 (2x d, $C_{aryl}$ (1)); 124.86, 124.87 $C_{aryl}$ (5); 125.57, 125.68 $C_{aryl}$ (6); 130.24, 130.25 $C_{aryl}$ (4); 134.32 C (6); 147.37, 147.44 C (2); 149.86-150.01 (2x d, $C_{aryl}$ (2)); 171.74, 171.77, 171.79, 171.84 2x C=O (Lev); 189.78, 189.81 C (4); 206.40, 206.44, 206.45 2x C=O (Lev) ppm. $^{31}$P-NMR ($CDCl_3$, 162 MHz): δ -9.73, -9.37 ppm (2x diastereomers).

$^{1}$H-NMR (400 MHz, $CDCl_3$) of compound **11a**

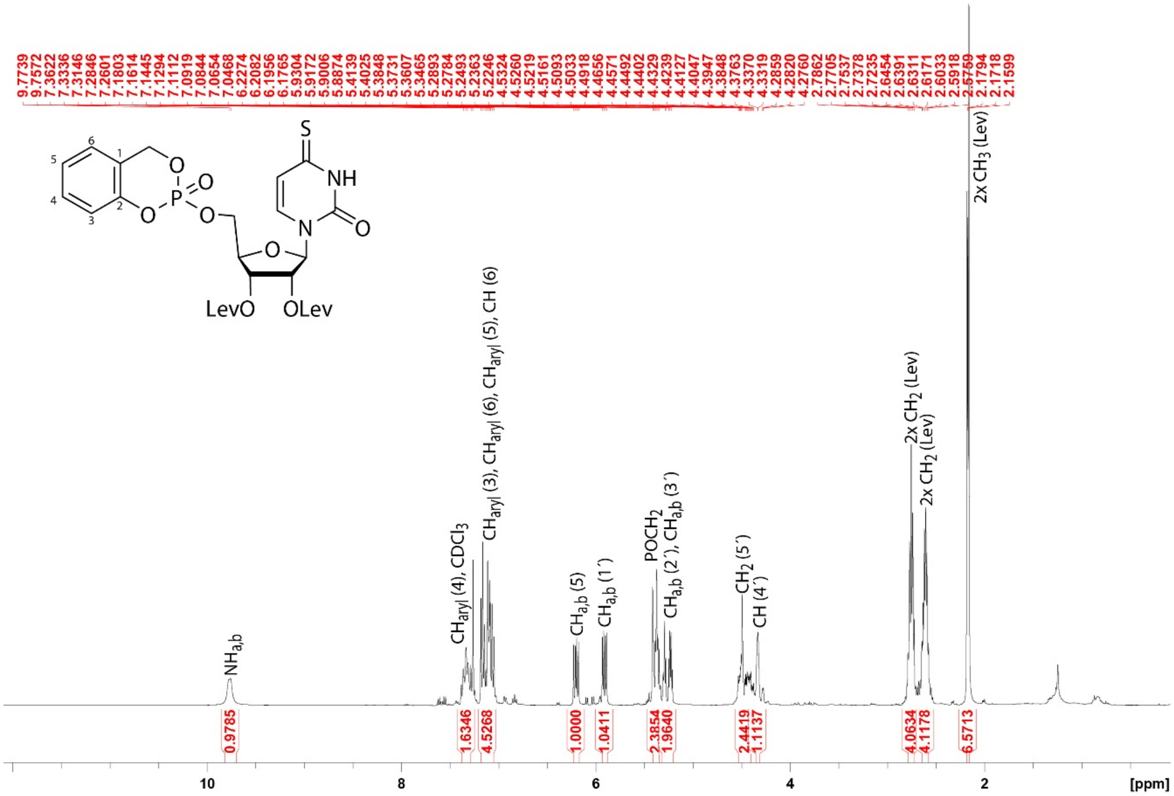

$^{13}$C-NMR (101 MHz, $CDCl_3$) of compound **11a**

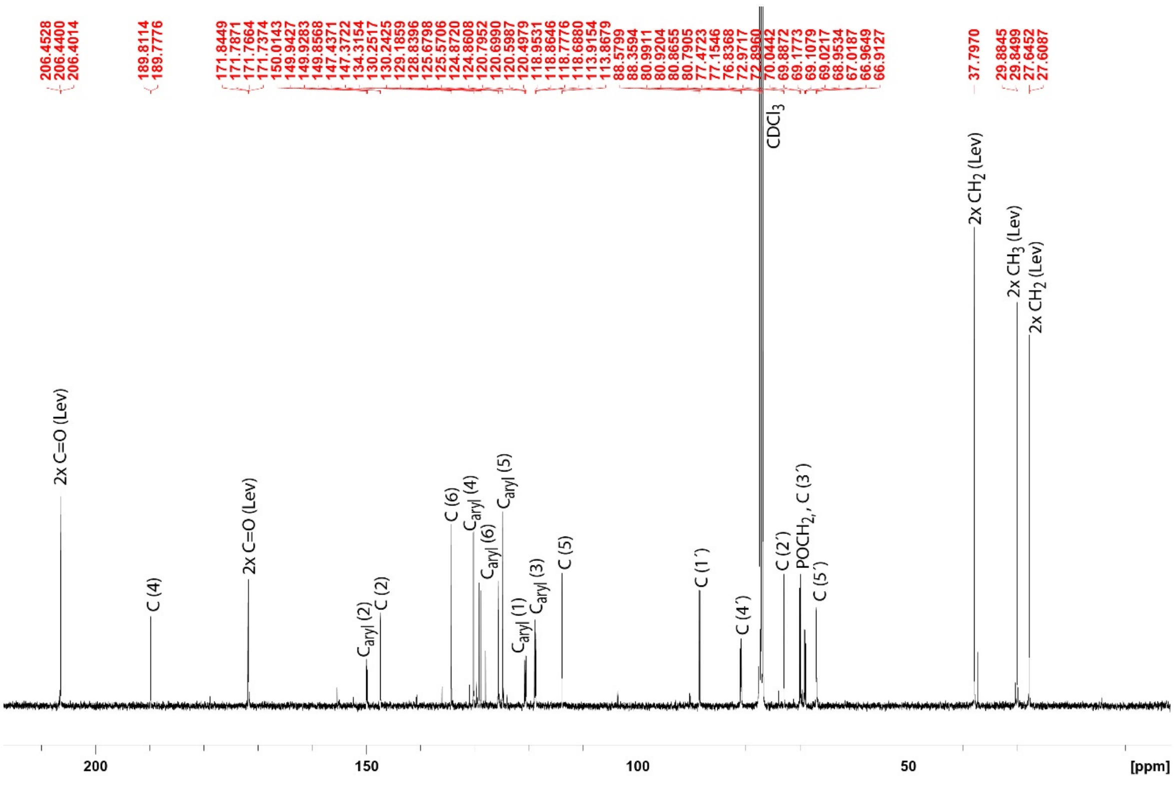

$^{31}$P-NMR (162 MHz, $CDCl_3$) of compound **11a**

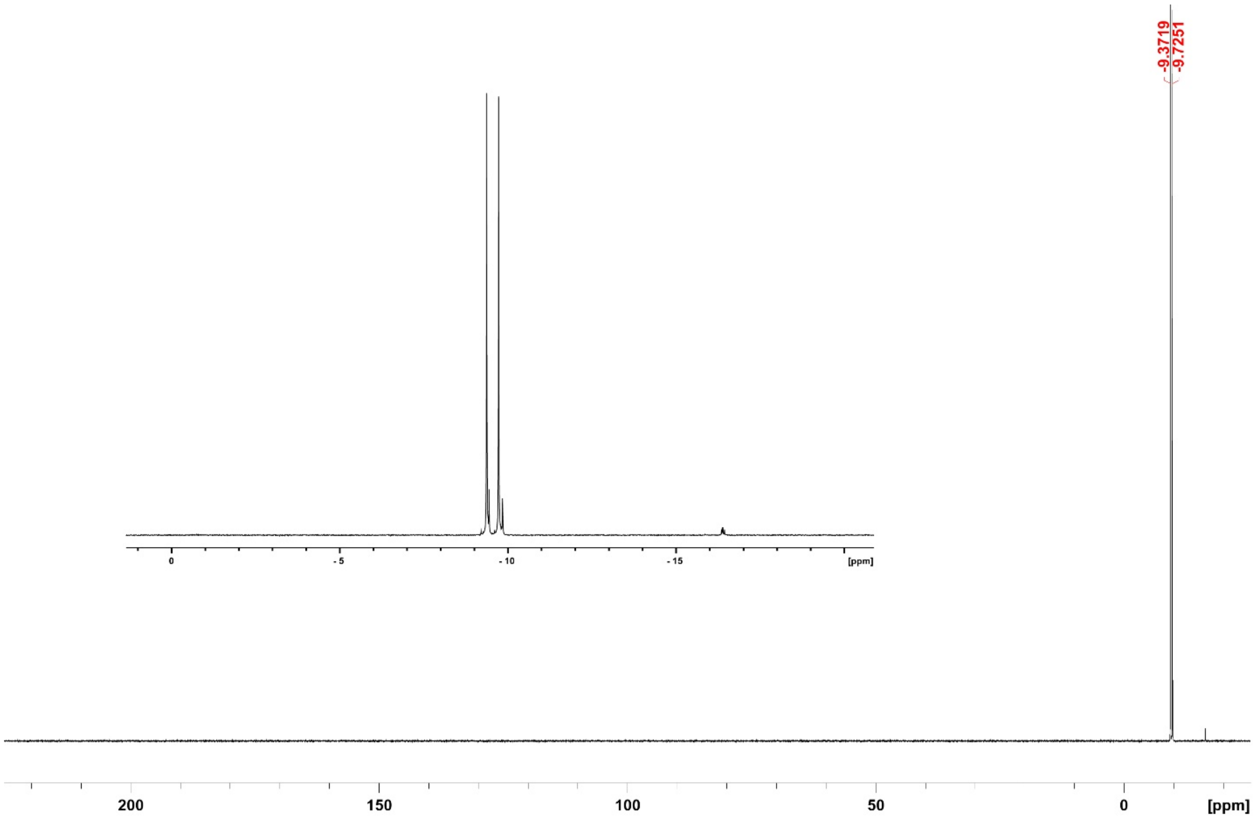

**4.4. Synthesis of cyclo(5-chlorosaligenyl)-5´-*O*-(2´,3´-di-*O*-levulinoyl-4-thiouridineyl) phosphate (11b)**

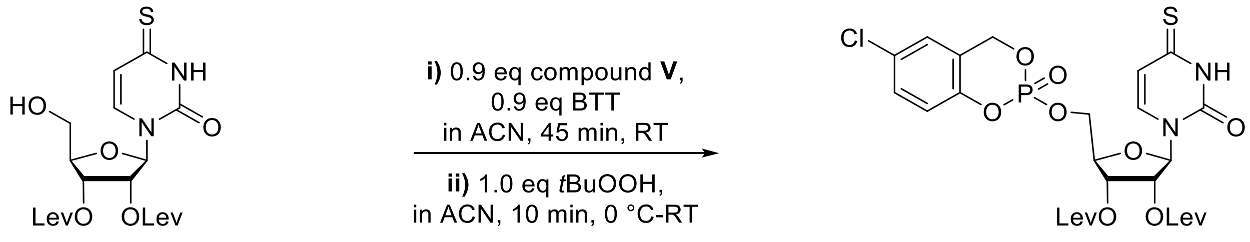


**10** **11b**

Compound **10** (204 mg, 0.45 mmol) was dissolved in 1.34 ml of 0.3 M 5-(benzylthio)-1*H*-tetrazole in acetonitrile (1.1 eq) and was subsequently treated dropwise with compound **V** (0.9 eq, 0.40 mmol, 116 mg) in acetonitrile (1.4 ml) over a period of 15 min. The reaction was stirred for another 30 min and was then cooled to 0 °C. *Tert*-butyl hydroperoxide in decane (1.0 eq, 0.45 mmol, 0.45 ml 1.0 M in toluene) was added dropwise and the yellow solution was allowed to stir 5 min at 0 °C and 5 min at room temperature. The reaction mixture was concentrated and purified by column chromatography (0-3% MeOH in $CH_2Cl_2$) yielding compound **11b** as a mixture of diastereomers. Yield: 144 mg of **11b** as yellow foam (54%). TLC (5% MeOH in $CH_2Cl_2$): 0.44. HR-ESI-MS (m/z): $[M+H]^+$ calculated for $[C_{26}H_{29}ClN_2O_{12}PS]^+$: 659.0862; found: 659.0844. $^1$H-NMR ($CDCl_3$, 400 MHz): δ 2.17, 2.176, 2.183, 2.19 (2x 3 H, 2x s, 2x $CH_3$ (Lev)); 2.58-2.66, 2.72-2.80 (8 H, m, 4x $CH_2$ (Lev)); 4.34 (1 H, m, CH (4´)); 4.38-4.46, 4.49-4.55 (2 H, 2x m, $CH_2$ (5´)); 5.24-5.38 (4 H, m, CH (2´), CH (3´), $POCH_2$); 5.86 (0.5 H, d, $^3J_{HH}$ = 5.05 Hz, $CH_a$ (1´)); 5.88 (0.5 H, d, $^3J_{HH}$ = 5.08 Hz, $CH_b$ (1´)); 6.29 (0.5 H, d, $^3J_{HH}$ = 7.70 Hz, $CH_a$ (5)); 6.31 (0.5 H, d, $^3J_{HH}$ = 7.73 Hz, $CH_b$ (5)); 7.00 (0.5 H, d, $^3J_{HH}$ = 3.48 Hz, $CH_{aryl,a}$ (3)); 7.02 (0.5 H, d, $^3J_{HH}$ = 3.49 Hz, $CH_{aryl,b}$ (3)); 7.09, 7.10 (1 H, 2x s, $CH_{aryl}$ (6)); 7.15 (0.5 H, d, $^3J_{HH}$ = 7.69 Hz, $CH_a$ (6)); 7.18 (0.5 H, d, $^3J_{HH}$ = 7.70 Hz, $CH_b$ (6)); 7.26-7.31 (1 H, m, $CH_{aryl}$ (4)); 9.81 (1 H, s, NH) ppm. $^{13}$C-NMR ($CDCl_3$, 101 MHz): δ 27.61, 27.64 2x $CH_2$ (Lev); 29.86, 29.89 2x $CH_3$ (Lev); 37.78, 37.82 2x $CH_2$ (Lev); 67.14-67.29 (2x d, C (5´)); 68.27-68.57 (2x d, $POCH_2$); 69.84, 69.96 C (3´); 72.91, 73.00 C (2´); 80.73-80.91 (2x d, C (4´)); 88.90, 89.15 C (1´); 113.90, 113.95 C (5); 120.09-120.42 (2x d, $C_{aryl}$ (3)); 122.02-122.25 (2x d, $C_{aryl}$ (1)); 125.57, 125.65 $C_{aryl}$ (6); 130.04, 130.19 $C_{aryl}$ (4) & $C_{aryl}$ (5)); 134.60 C (6); 147.40, 147.43 C (2); 148.45.148.61 (2x d, $C_{aryl}$ (2)); 171.82, 171.85, 171.88 2x C=O (Lev); 189.83, 189.87 C (4); 206.41, 206.46, 206.50 2x C=O (Lev) ppm. $^{31}$P-NMR ($CDCl_3$, 162 MHz): δ -10.13, -9.77 ppm (2 diastereomers).

$^{1}$H-NMR (400 MHz, $CDCl_3$) of compound **11b**

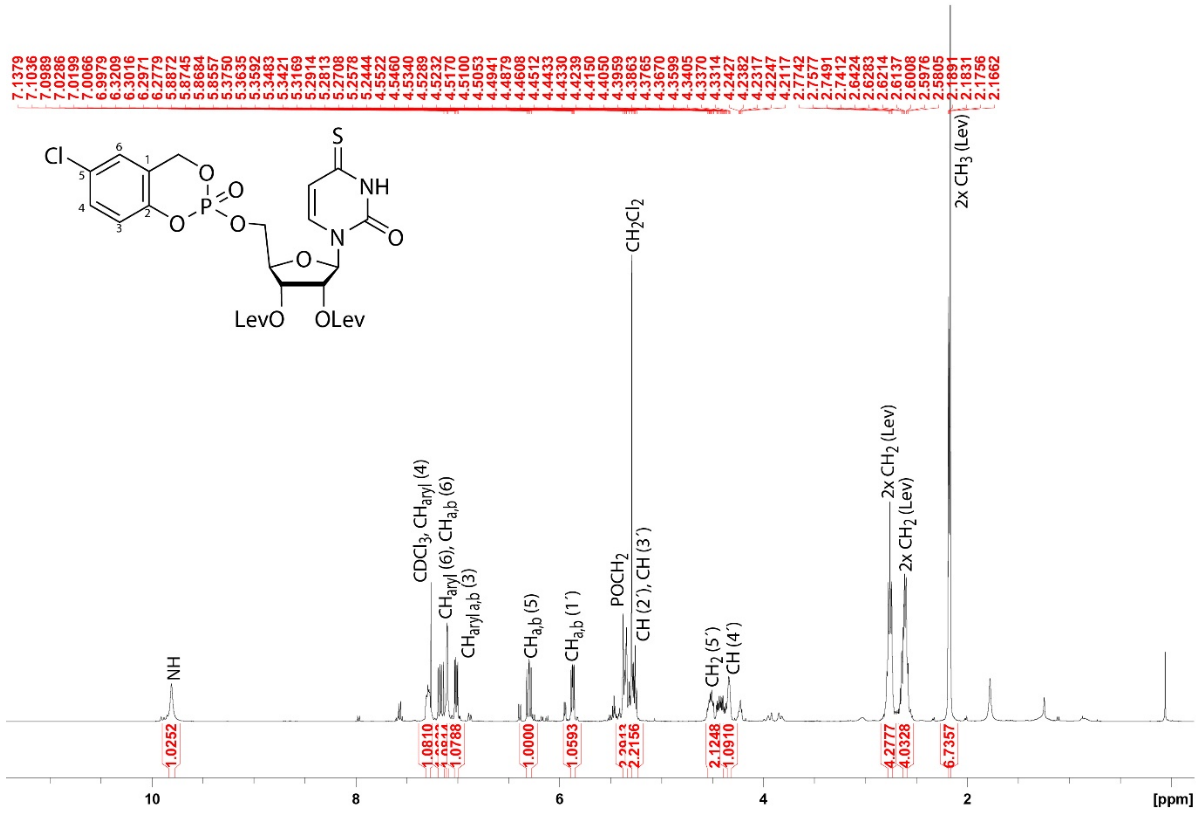

$^{13}$C-NMR (101 MHz, $CDCl_3$) of compound **11b**

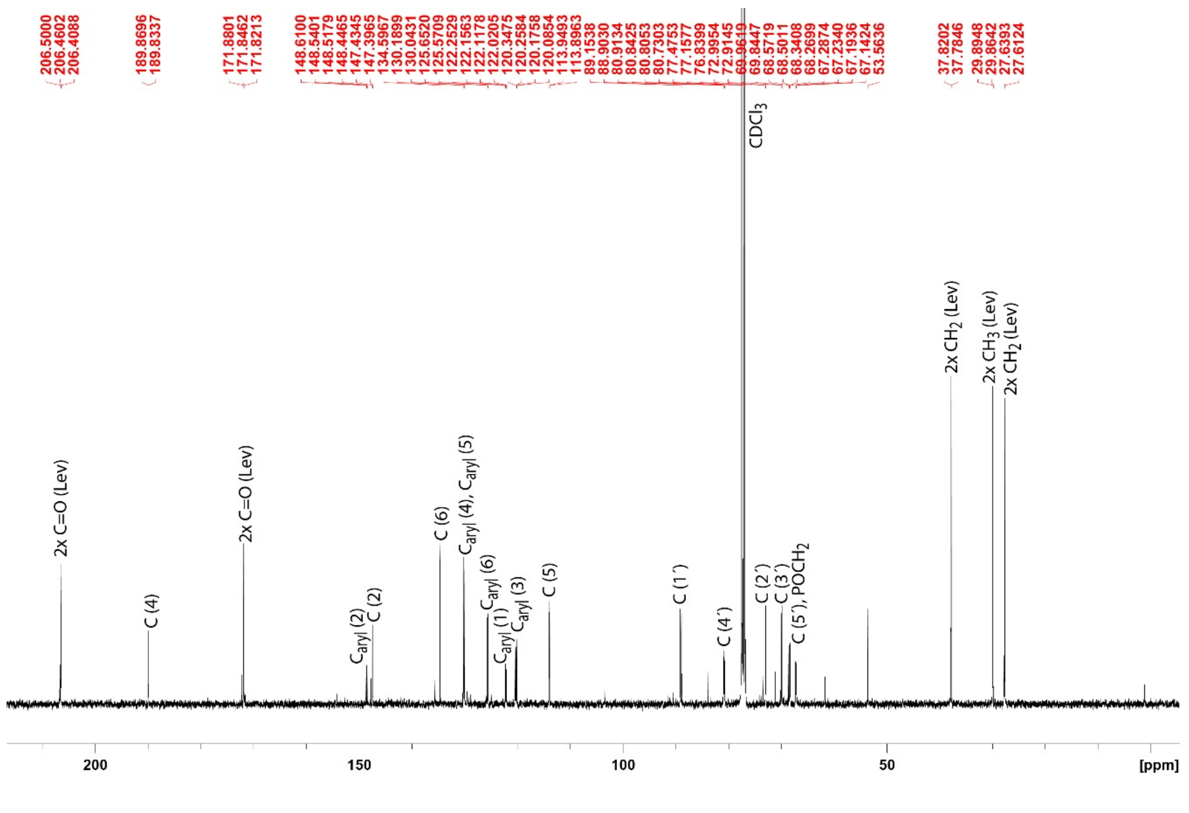

$^{31}$P-NMR (162 MHz, $CDCl_3$) of compound **11b**

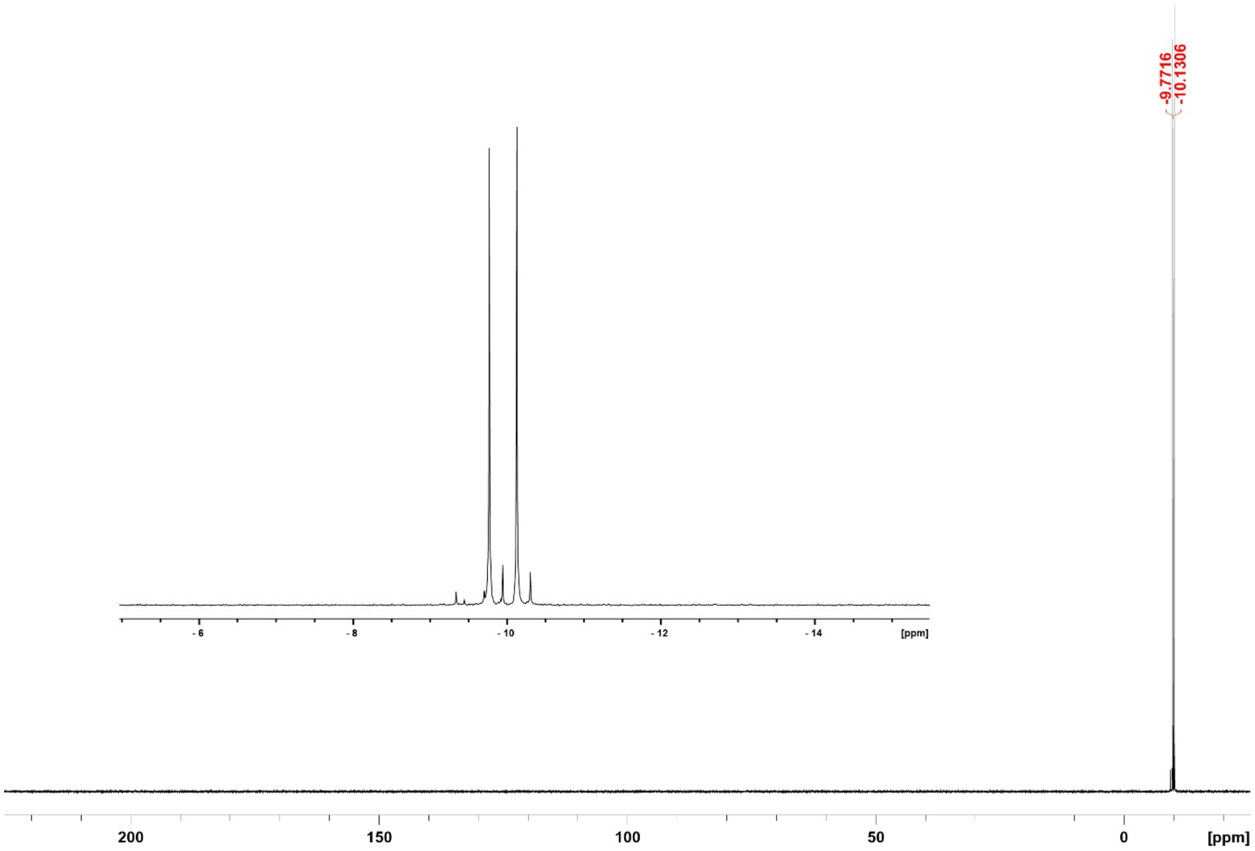

### 4.5. Synthesis of 2-ethylbutyl-2-(5´-*O*-(2´,3´-di-*O*-levulinoyl-thiouridinyl)(phenoxy)phosphoryl) alaninate (11c) [10]

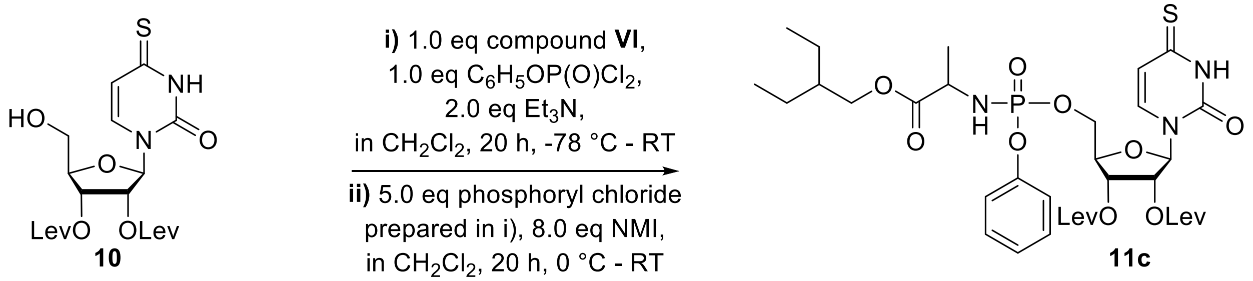


Compound **VI** (440 mg, 2.54 mmol) was dried at 50 °C under high vacuum overnight. It was then dissolved in dichloromethane (7.8 ml) and cooled to 0 °C. Phenyldichlorophosphate (1.0 eq. 2.54 mmol, 536 mg) was added and the clear solution was further cooled to -78 °C whereas triethylamine (2.0 eq, 5.08 mmol, 514 mg) was added dropwise over a period of 30 min. The reaction mixture was stirred for another 2 h at -78 °C and was then allowed to warm to ambient temperature overnight (20 h). Solvent was evaporated on a pre-dried rotavapor, which was flushed with argon after evaporation. The remaining residue was dissolved in diethyl ether (11.0 ml) and salts were removed by filtration using a Schlenk-frit. Evaporation of the solvent was performed as described above, yielding 873 mg of the chloro-intermediate which was used for the next step without further purification.

Compound **10** (51 mg, 0.11 mmol) was dissolved in dichloromethane (3.0 ml) and cooled to 0 °C. Freshly prepared 2-ethylbutyl (chloro(phenoxy)phosphoryl) alaninate (5.0 eq, 0.56 mmol, 194 mg) was added followed by the dropwise addition of *N*-methyl imidazole (8.0 eq, 0.89 mmol, 71 µl). The yellow solution was stirred for 20 h at ambient temperature. It was then diluted with dichloromethane and was washed with brine. The crude product was purified by column chromatography (0-3% MeOH in $CH_2Cl_2$). Yield: 59 mg of **11c** as yellow oil (69%). TLC (3% MeOH in $CH_2Cl_2$): 0.48. HR-ESI-MS (m/z): $[M+H]^+$ calculated for $[C_{34}H_{47}N_3O_{13}PS]^+$: 768.2562; found: 768.2535. $^1$H-NMR ($CDCl_3$, 400 MHz): δ 0.80 (3 H, t, $^3J_{HH}$ = 7.47 Hz, $CH_3$ (ester)); 0.81 (3 H, t, $^3J_{HH}$ = 7.41 Hz, $CH_3$ (ester)); 1.24-1.36 (4 H, m, 2x $CH_2$ (ester)); 1.34 (1.5 H, d, $^3J_{HH}$ = 7.04 Hz, $CH_{3,a}$ (Ala)); 1.39 (1.5 H, d, $^3J_{HH}$ = 6.99 Hz, $CH_{3,b}$ (Ala)); 1.50 (1 H, sept, $^3J_{HH}$ = 6.23 Hz, CH (ester)); 2.157, 2.164, 2.173 (2x 3 H, 3x s, 2x $CH_3$ (Lev)); 2.59-2.65, 2.71-2.79 (2x 4 H, 2x m, 4x $CH_2$ (Lev)); 3.90-4.10 (3 H, m, $OCH_2$ (ester), CH (Ala)); 4.28-4.45 (3 H, m, CH (4´), $CH_2$ (5´)); 5.12 (0.5 H, t, $^3J_{HH}$ = 5.96 Hz, $CH_a$ (2´)); 5.22 (0.5 H, t, $^3J_{HH}$ = 6.04 Hz, $CH_b$ (2´)); 5.39 (0.5 H, dd, $^3J_{HH}$ = 5.84 Hz, $^3J_{HH}$ =3.84 Hz, $CH_a$ (3´)); 5.42 (0.5 H, dd, $^3J_{HH}$ = 5.84 Hz, $^3J_{HH}$ =3.92 Hz, $CH_b$ (3´)); 6.01 (0.5 H, d, $^3J_{HH}$ = 6.04 Hz, $CH_a$ (1´)); 6.04 (0.5 H, d, $^3J_{HH}$ = 6.12 Hz, $CH_b$ (1´)); 6.15 (0.5 H, d, $^3J_{HH}$ = 6.64 Hz, $CH_a$ (5)); 6.37 (0.5 H, d, $^3J_{HH}$ = 6.68 Hz, $CH_b$ (5)); 7.09-7.23, 7.29-7.34 (6 H, m, CH (6), aromat. CH); 9.95, 10.07 (1 H, 2x s, $NH_{a,b}$) ppm. $^{13}$C-NMR ($CDCl_3$, 101 MHz): δ 11.03, 11.05, 11.08 2x $CH_3$ (ester); 21.10-21.27 (2x d, $CH_3$ (Ala)); 23.24, 23.27 2x $CH_2$ (ester); 27.61, 27.68, 27.69 2x $CH_2$ (Lev); 29.85, 29.88 2x $CH_3$ (Lev); 37.77, 37.81, 37.84 2x $CH_2$ (Lev); 40.28 CH (ester); 50.49-50.60 (2x d, CH (Ala)); 65.43 (d, $^2J_{CP}$ = 5.09 Hz, $C_a$ (5´)); 65.66 (d, $^2J_{CP}$ = 5.69 Hz, $C_b$ (5´)); 67.82, 67.85 $OCH_2$ (ester); 70.13, 70.36 C (3´); 72.89, 73.06 C (2´); 81.25-81.38 (2x d, C (4´)); 86.97, 87.13 C (1´); 114.07, 114.20 C (5); 120.18-120.37 (2x d, aromat. C); 125.29, 125.46, 129.93, 130.04

aromat. C; 134.02, 134.38 C (6); 147.60, 147.66 C (2); 150.42-150.57 (2x d, aromat. $C_q$); 171.64, 171.78, 171.83 2x C=O (Lev); 173.57 (d, $^3J_{CP}$ = 7.26 Hz, C=$O_a$ (Ala)); 173.88 (d, $^3J_{CP}$ = 7.26 Hz, C=$O_b$ (Ala)); 189.85, 190.03 C (4); 206.28, 206.30, 206.35, 206.41 2x C=O (Lev) ppm. $^{31}$P-NMR ($CDCl_3$, 162 MHz): δ 2.50, 2.75 ppm (2 diastereomers).

$^1$H-NMR (400 MHz, $CDCl_3$) of compound **11c**

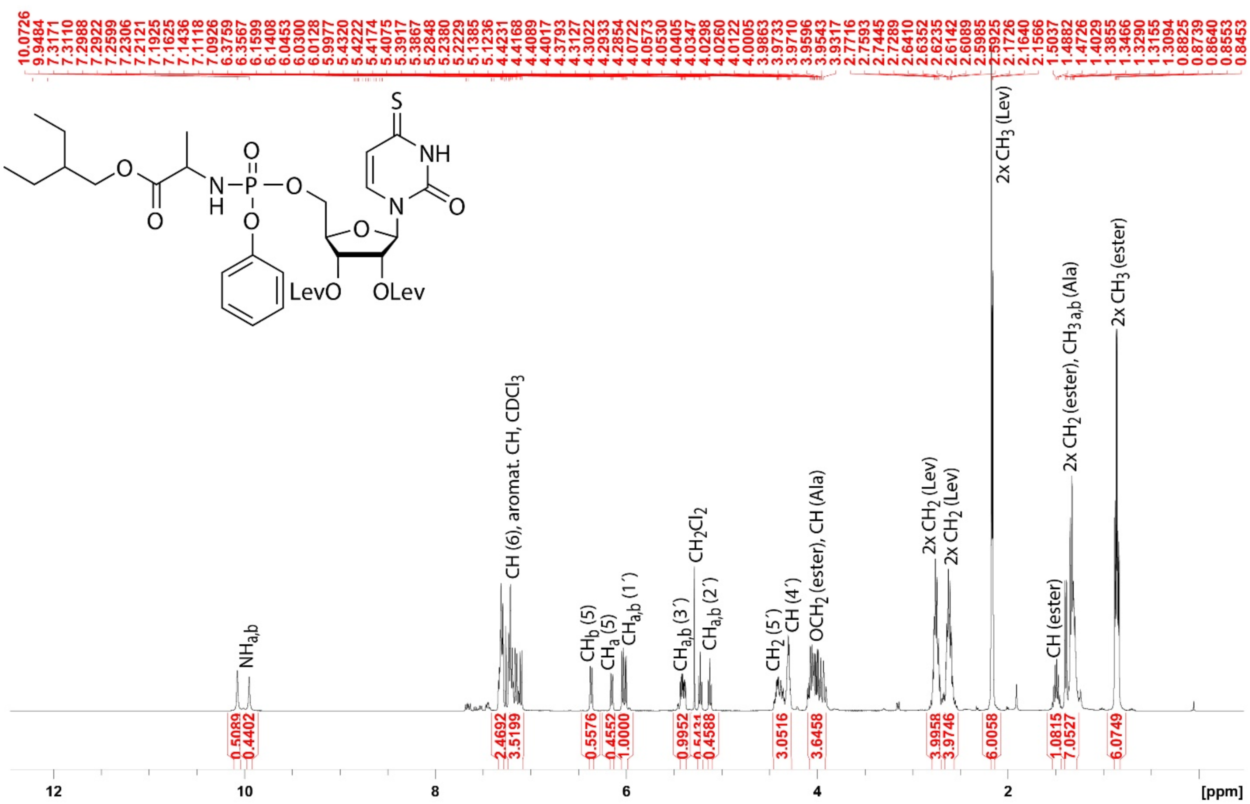

$^{13}$C-NMR (101 MHz, $CDCl_3$) of compound **11c**

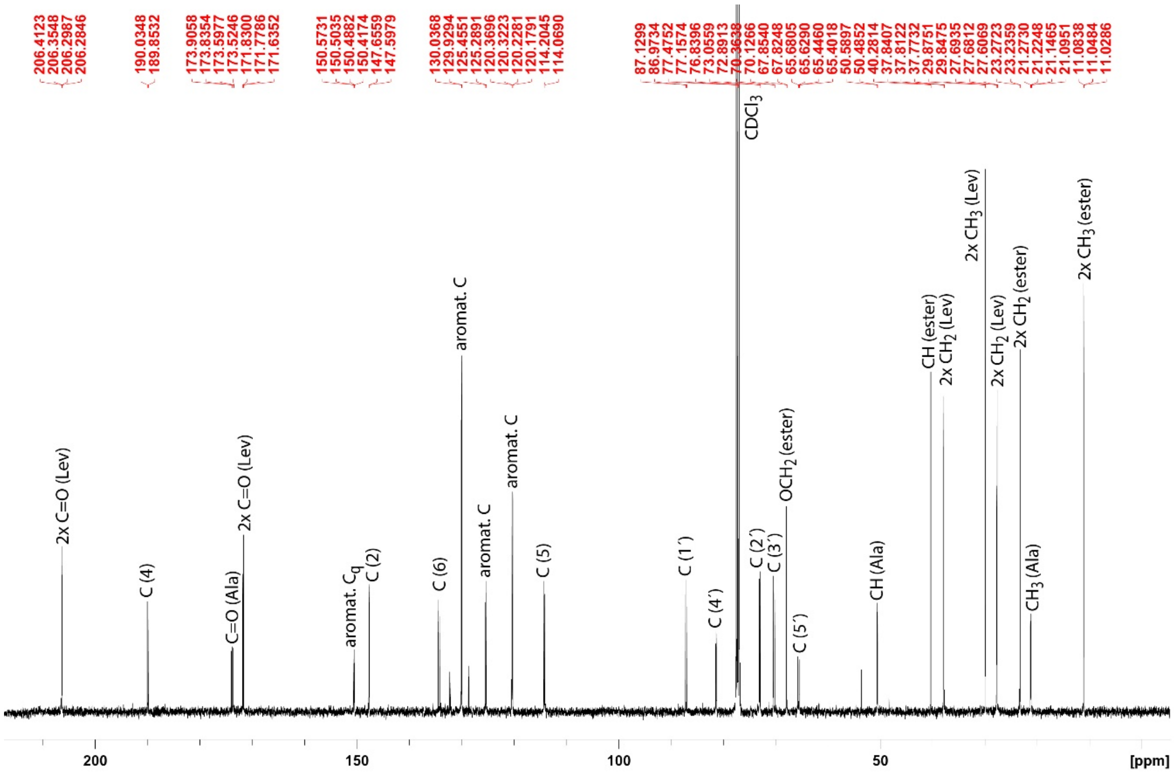


$^{31}$P-NMR (162 MHz, $CDCl_3$) of compound **11c**

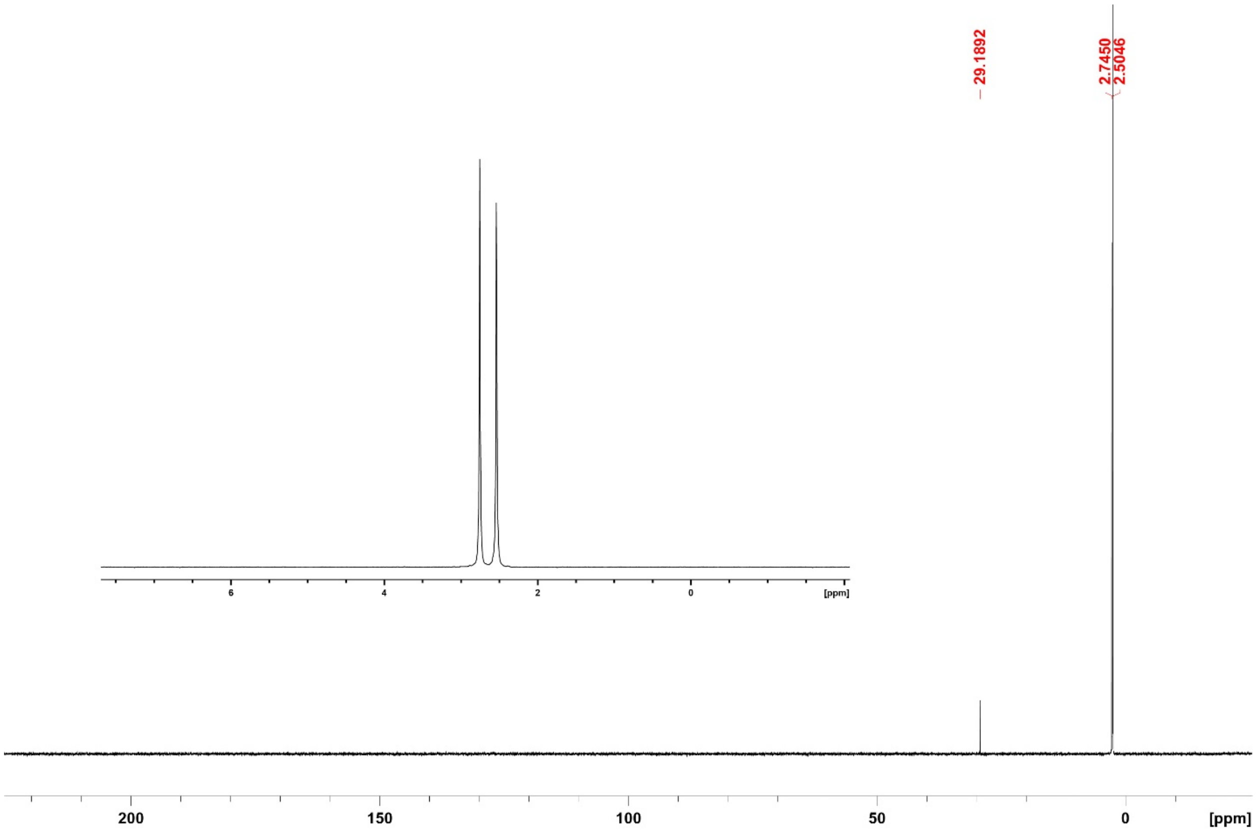

### 4.6. Synthesis of cyclosaligenyl-5´-*O*-(4-thiouridineyl) phosphate (CycloSal-4sU, 12a) [11]

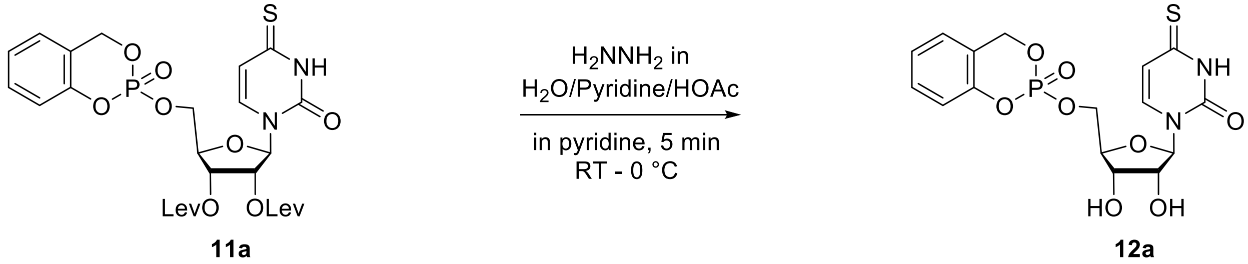


Compound **11a** (43 mg, 0.07 mmol) was dissolved in pyridine (2.5 ml) and treated with hydrazine in a pyridine/acetic acid-buffer (24-26% $H_2NNH_2$ in $H_2O$/pyridine/acetic acid 2/4/3, 3.3 ml) and was stirred for 5 min. The reaction mixture was then cooled to 0 °C and ethyl acetate (12.5 ml) and water (12.5 ml) were added and stirred vigorously for 5 min at 0 °C. The organic layer was washed with 5% sodium bicarbonate solution, dried over sodium sulfate and was purified by flash chromatography (0-4% MeOH in $CH_2Cl_2$). Yield: 16 mg of **12a** as a slightly yellow oil (53%). TLC (5% MeOH in $CH_2Cl_2$): 0.29. HR-ESI-MS (m/z): $[M+Na]^+$ calculated for $[C_{16}H_{17}N_2O_8PSNa]^+$: 451.0335; found: 451.0329. $^1$H-NMR ($d_6$-DMSO, 400 MHz): δ 3.93 (1 H, m, CH (3´)); 4.01-4.06 (2 H, m, CH (4´), CH (2´)); 4.30-4.44 (2 H, m, $CH_2$ (5´)); 5.35 (1 H, s br., OH (3´)); 5.42-5.57 (3 H, m, $POCH_2$, OH (2´)); 5.69-5.70 (1 H, 2x d, CH (1´)); 6.07 (0.5 H, dd, $^3J_{HH}$ = 7.58 Hz, $^4J_{HH}$ = 1.78 Hz, $CH_a$ (5´)); 6.16 (0.5 H, dd, $^3J_{HH}$ = 7.56 Hz, $^4J_{HH}$ = 1.83 Hz, $CH_b$ (5´)); 7.14-7.45 (5 H, m, aromat. CH, CH (6)); 12.74 (1 H, s br., NH) ppm. $^{13}$C-NMR ($d_6$-DMSO, 101 MHz): δ 67.29 (d, $^2J_{CP}$ = 5.06 Hz, $C_a$ (5´)); 67.33 (d, $^2J_{CP}$ = 4.36 Hz, $C_b$ (5´)); 68.47-68.58 (2x d, $POCH_2$); 69.13, 69.16 C (3´); 72.80, 72.87 C (2´); 81.69-81.79 (2x d, C (4´)); 89.33, 89.39 C (1´); 112.61, 122.65 C (5); 118.18-118.28 (2x d, $C_{aryl}$ (3)); 120.86-121.00 (2x d, $C_{aryl}$ (1)); 124.48, 124.52 $C_{aryl}$ (5); 126.13 $C_{aryl}$ (6); 129.80 $C_{aryl}$ (4); 135.56, 135.70 C (6); 147.76 C (2); 149.32-149.43 (2x d, $C_{aryl}$ (2)); 190.19, 190.21 C (4); ppm. $^{31}$P-NMR ($d_6$-DMSO, 162 MHz): δ -9.86, -9.78 ppm (2 diastereomers).

$^{1}H$-NMR (400 MHz, $d_6$-DMSO) of compound **12a**

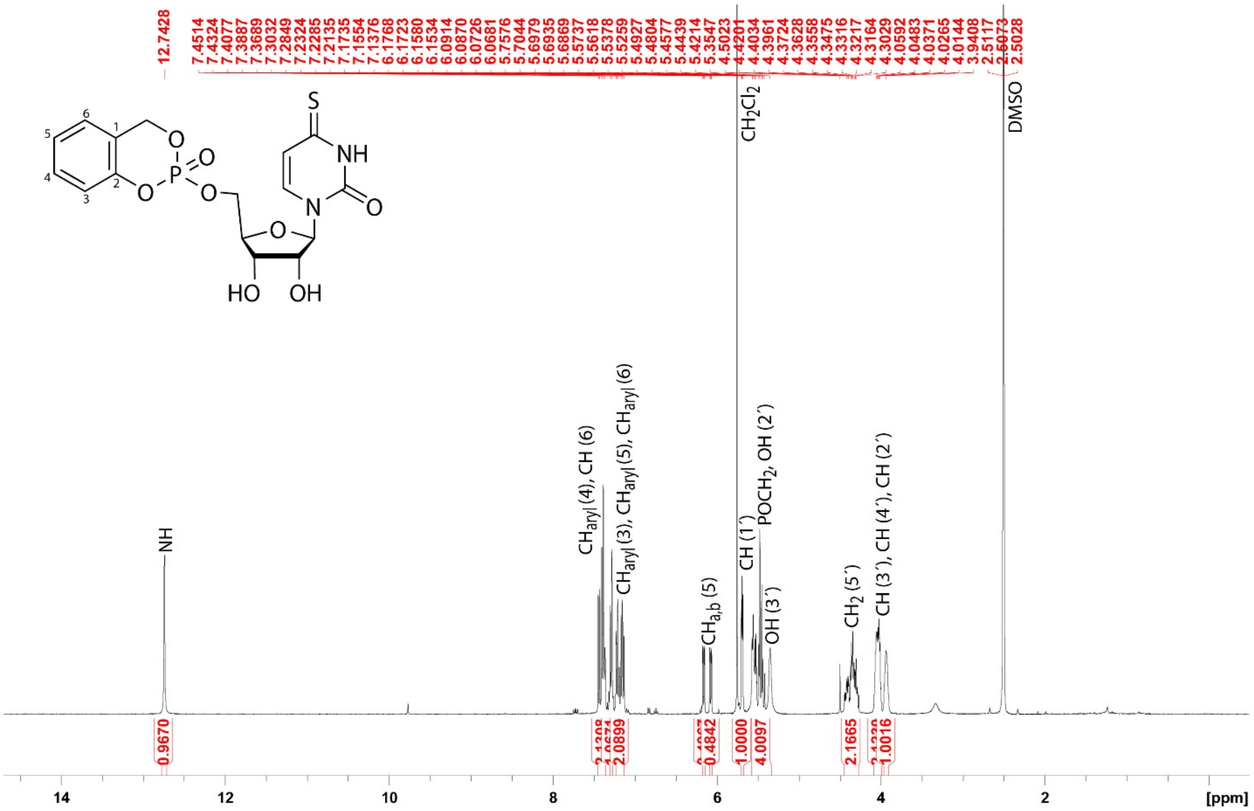


$^{13}C$-NMR (101 MHz, $d_6$-DMSO) of compound **12a**

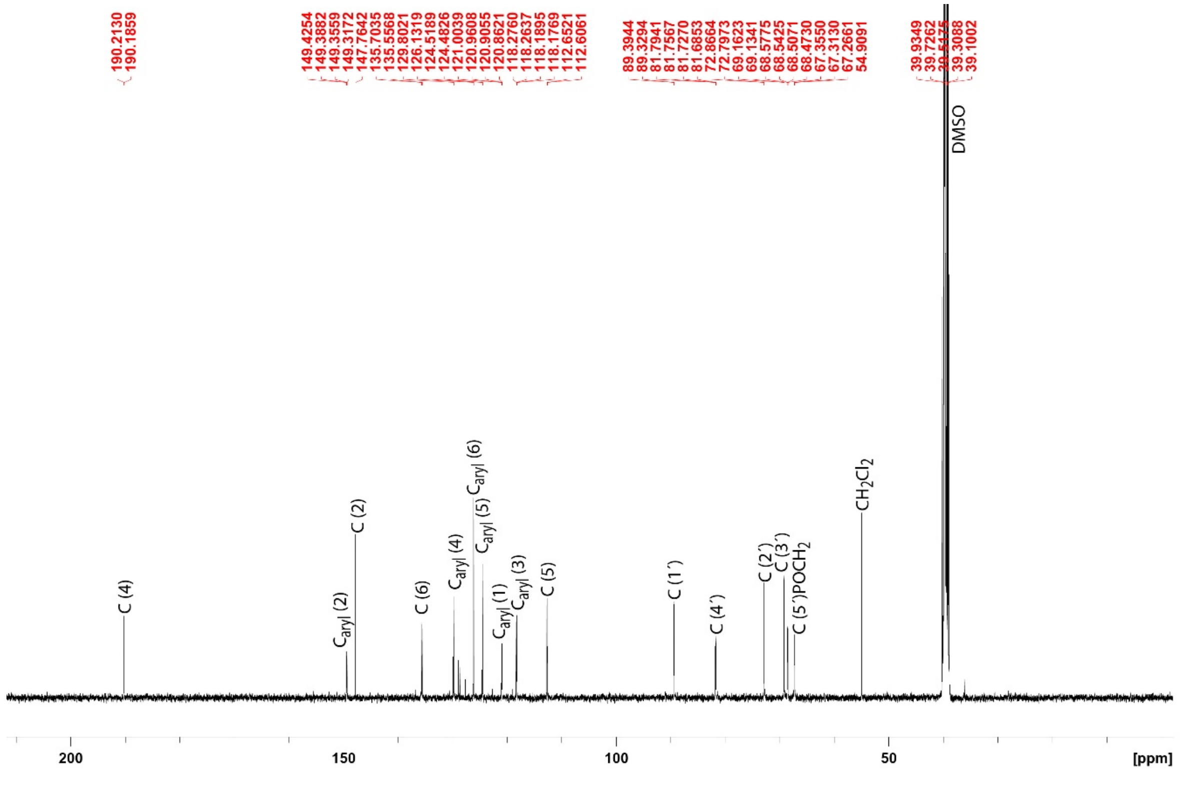

$^{31}$P-NMR (162 MHz, $d_6$-DMSO) of compound **12a**

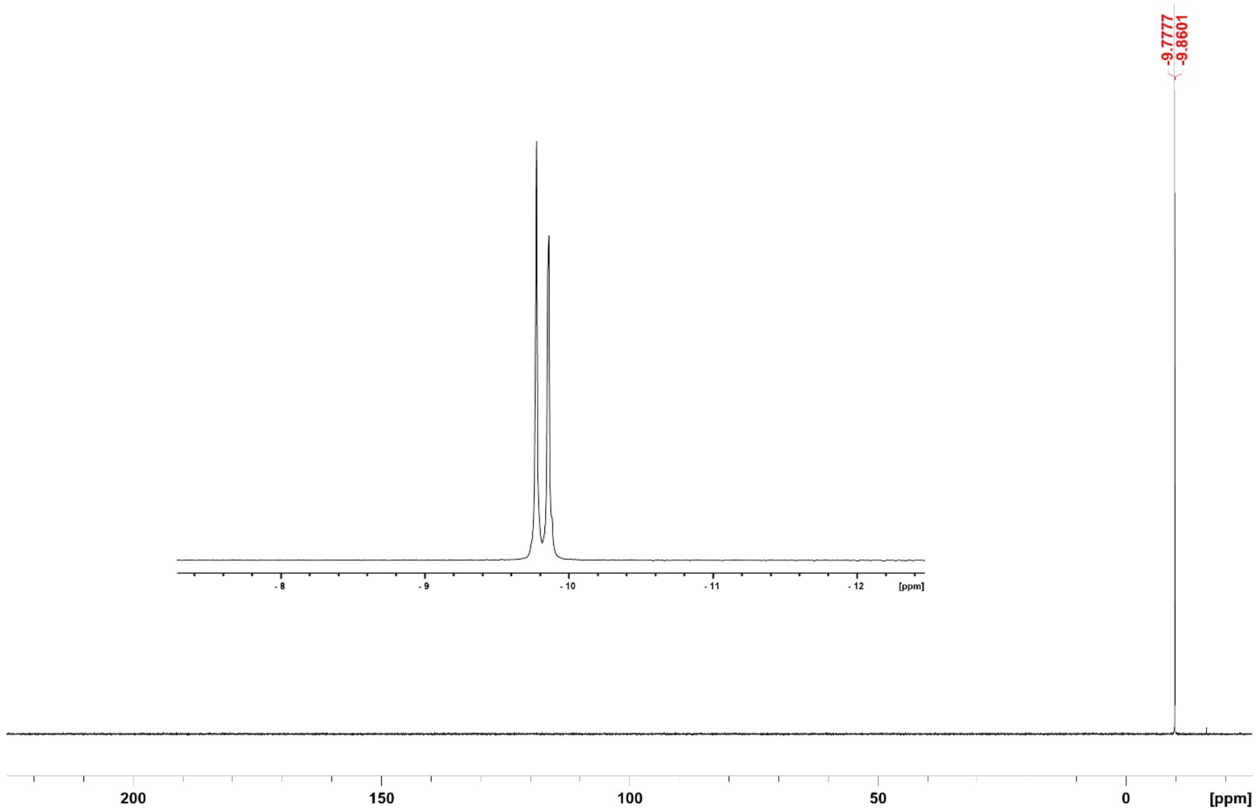

**4.7. Synthesis of cyclo(5-chlorosaligenyl)-5´-*O*-(4-thiouridineyl) phosphate (Cl-CycloSal-4sU, 12b)** [11]

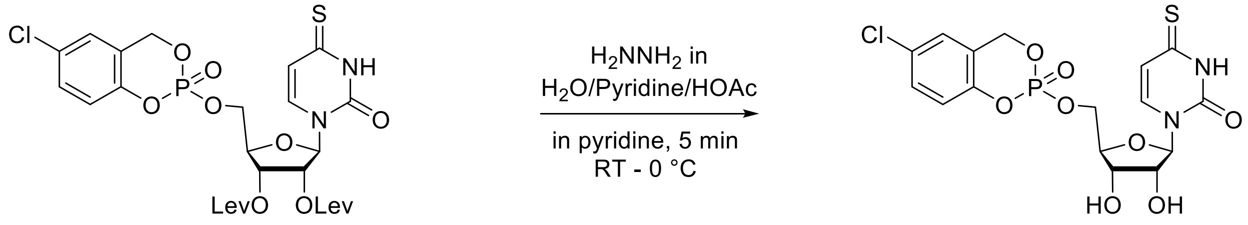


Compound **11b** (100 mg, 0.15 mmol) was dissolved in pyridine (5.4 ml) and treated with hydrazinium acetate (24-26% $H_2NNH_2$ in $H_2O$/pyridine/acetic acid 2/4/3, 7.2 ml) and was stirred for 5 min. The reaction mixture was then cooled to 0 °C and ethyl acetate (27.0 ml) and water (27.0 ml) were added and stirred vigorously. The organic layer was washed with 5% sodium bicarbonate solution, dried over sodium sulfate and was purified by flash chromatography (1-5% MeOH in $CH_2Cl_2$). Yield: 23 mg of **12b** as colorless oil (33%). TLC (5% MeOH in $CH_2Cl_2$): 0.25. HR-ESI-MS (m/z): $[M+H]^+$ calculated for $[C_{16}H_{17}N_2O_8PS]^+$: 463.0126; found: 463.0112. $^1$H-NMR ($CDCl_3$, 400 MHz): δ 4.24-4.28 (1 H, m, CH (2´)); 4.30 (1 H, m, CH (4´)); 4.38 (1 H, m, CH (3´)); 4.44 (1 H, m, $CH_a$ (5´)); 4.53 (1 H, m, $CH_b$ (5´)); 5.27-5.37 (2 H, m, $POCH_2$); 5.609 (0.5 H, d, $^3J_{HH}$ = 3.38 Hz, $CH_a$ (1´)); 5.614 (0.5 H, d, $^3J_{HH}$ = 3.47 Hz, $CH_b$ (1´)); 6.33 (0.5 H, d, $^3J_{HH}$ = 7.66 Hz, $CH_a$ (5)); 6.36 (0.5 H, d, $^3J_{HH}$ = 7.66 Hz, $CH_b$ (5)); 7.01 (1 H, d, $^3J_{HH}$ = 8.75 Hz, $CH_{aryl}$ (3)); 7.11 (1 H, m, $CH_{aryl}$ (6)); 7.32 (1 H, m, $CH_{aryl}$ (4)); 7.35 (0.5 H, d, $^3J_{HH}$ = 7.47 Hz, $CH_a$ (6)); 7.36 (0.5 H, d, $^3J_{HH}$ = 7.61 Hz, $CH_a$ (6)); ppm. $^{13}$C-NMR ($CDCl_3$, 101 MHz): δ 67.58-67.65 (2x d, C (5´)); 68.32-68.40 (2x d, $POCH_2$); 70.32, 70.41 C (3´); 75.34, 75.39 C (2´); 83.34-83.45 (2x d, C (4´)); 93.25, 93.40 C (1´); 113.51, 113.55 C (5); 120.16-120.23 (2x d, $C_{aryl}$ (3)); 121.99-122.05 (2x d, $C_{aryl}$ (1)); 125.63 $C_{aryl}$ (6); 130.29, 130.32, 130.40 $C_{aryl}$ (4) & $C_{aryl}$ (5)); 134.74, 134.83 C (6); 148.31 C (2) & $C_{aryl}$ (2); 189.41, 189.45 C (4) ppm. $^{31}$P-NMR ($d_6$-DMSO, 162 MHz): δ -9.40, -9.29 ppm (2 diastereomers).

$^1$H-NMR (700 MHz, d$_6$-DMSO) of compound **12b**

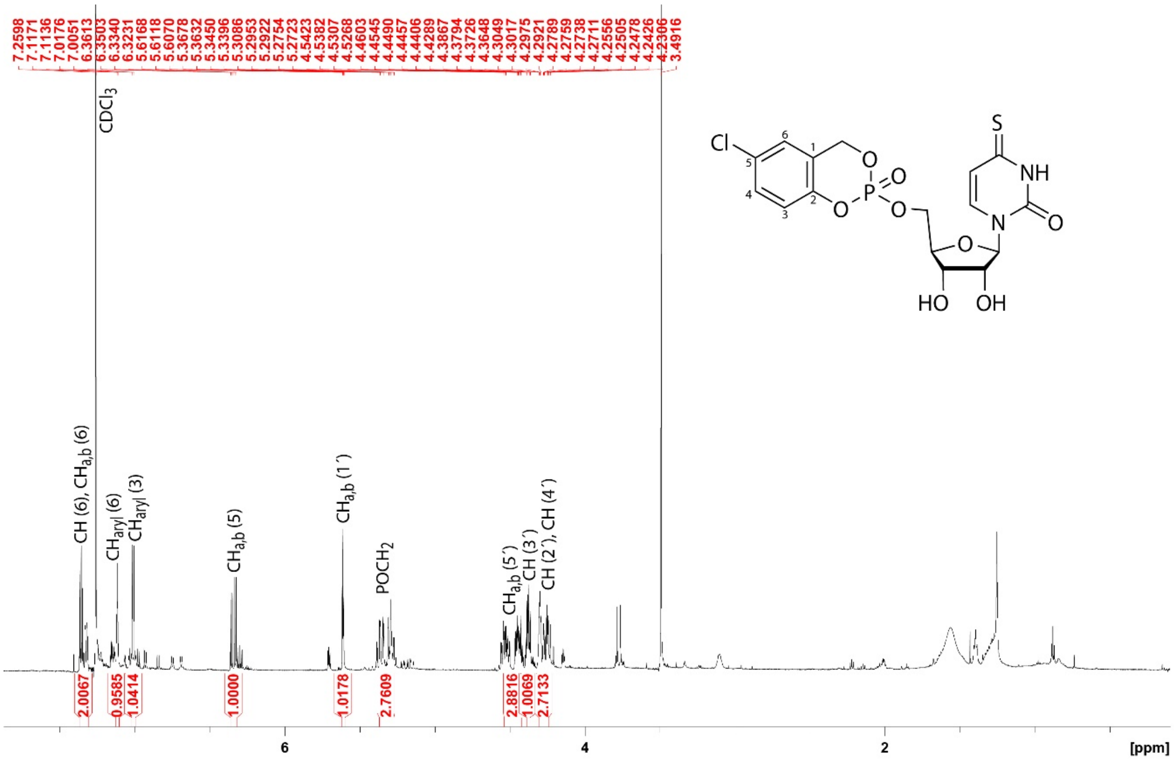

$^{13}$C-NMR (176 MHz, d$_6$-DMSO) of compound **12b**

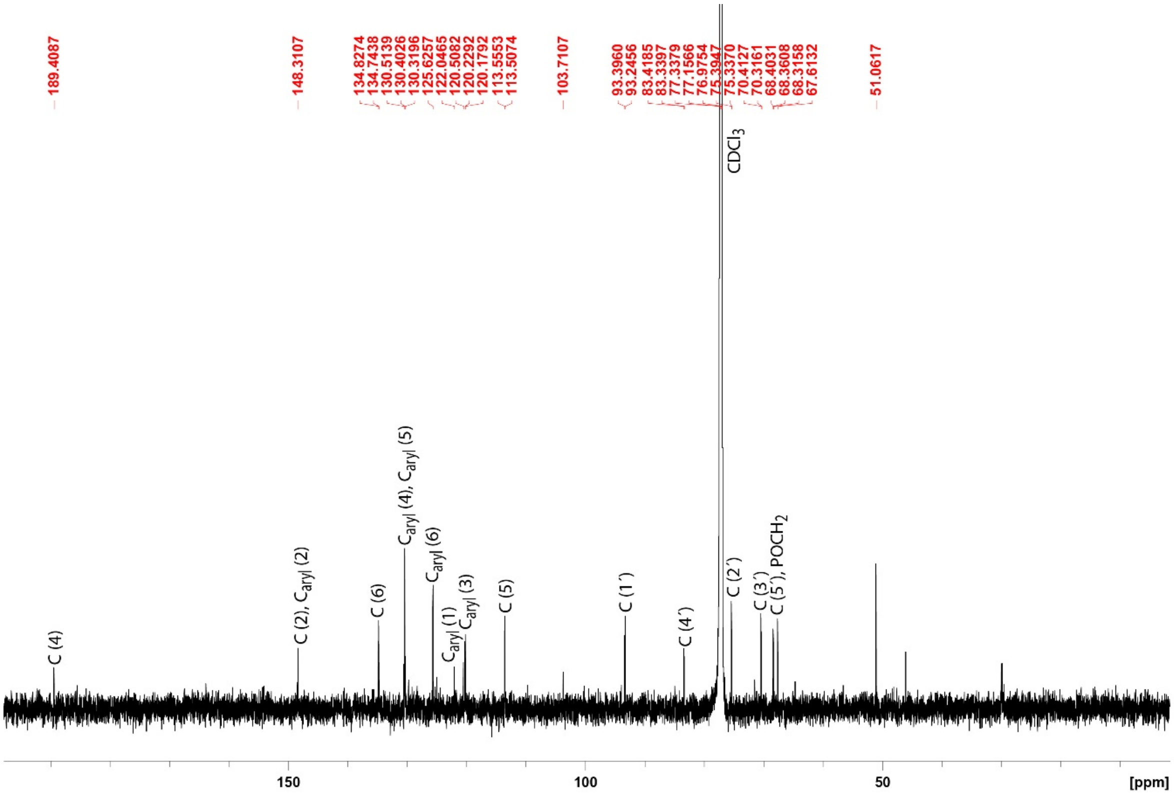

$^{31}$P-NMR (162 MHz, $d_6$-DMSO) of compound **12b**

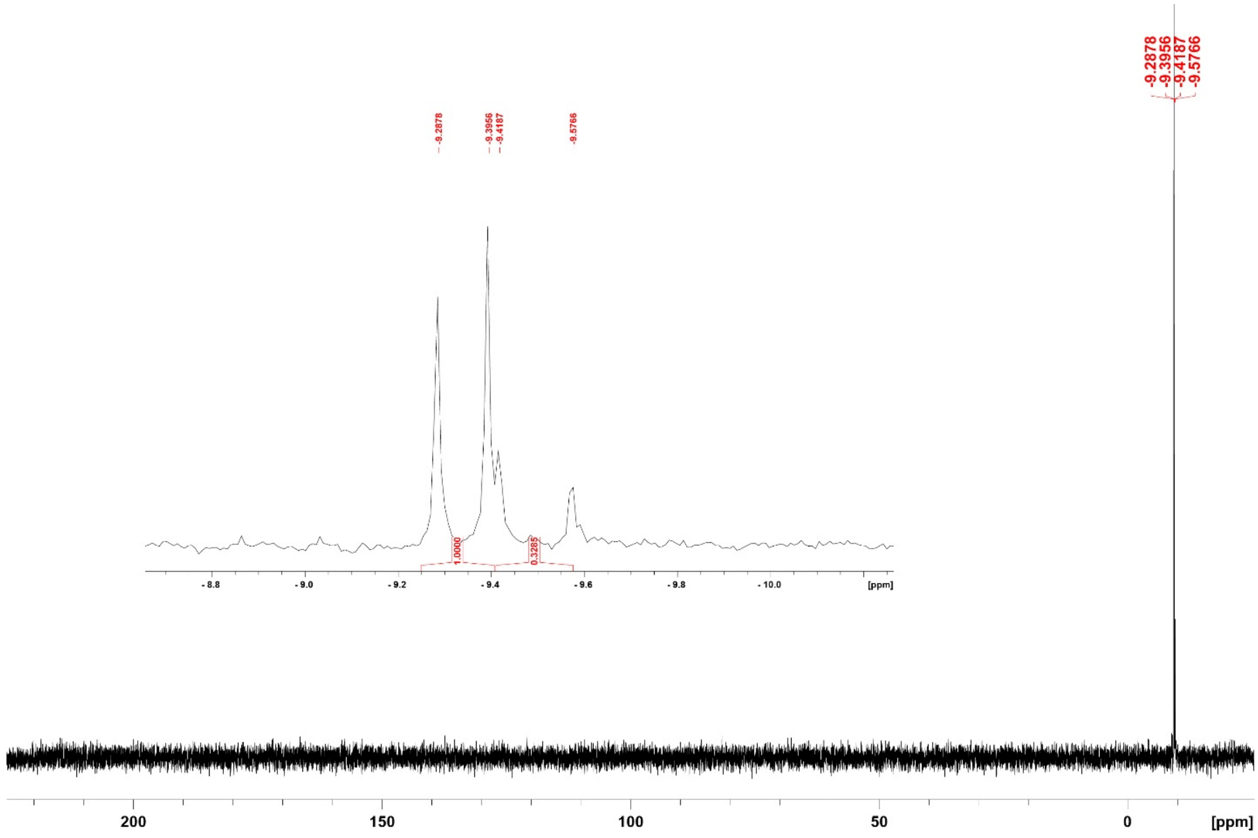

### 4.8. Synthesis of 2-ethylbutyl-2-(5´-*O*-(4-thiouridineyl)(phenoxy) phosphoryl)alaninate (ProTide-4sU, 12c) [11,12]

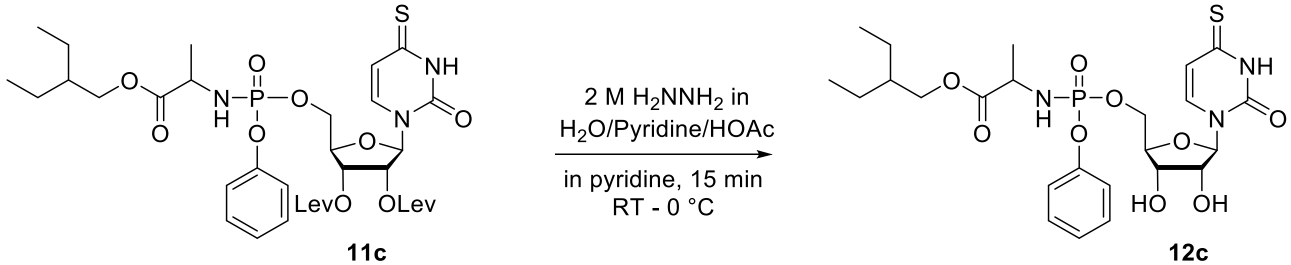


**11c** **12c**

Compound **11c** (41 mg, 0.053 mmol) was dissolved in pyridine (0.30 ml) and treated with 0.19 ml 2 M hydrazine buffer (hydrazine monohydrate/pyridine/acetic acid 1/5/4). After 10 minutes the solution was cooled to 0 °C and ethyl acetate (4.0 ml) as well as water (4.0 ml) was added and the reaction mixture was stirred vigorously for 5 minutes. The organic phase was separated and washed with 5% sodium bicarbonate solution. Drying over sodium sulfate was followed by evaporation of the solvent and column chromatography (0-4% MeOH in $CH_2Cl_2$). Yield: 25 mg of **12c** as slightly yellow oil (83%). TLC (5% MeOH in $CH_2Cl_2$): 0.35 (two diastereomers). HR-ESI-MS (m/z): $[M+H]^+$ calculated for $[C_{24}H_{35}N_3O_9PS]^+$: 572.1826; found: 572.1815. $^1$H-NMR ($CDCl_3$, 400 MHz): δ 0.85 (3 H, t, $^3J_{HH}$ = 7.50 Hz, $CH_3$ (ester)); 0.86 (3 H, t, $^3J_{HH}$ = 7.49 Hz, $CH_3$ (ester)); 1.25-1.36 (7 H, m, 2x $CH_2$ (ester), $CH_3$ (Ala)); 1.49 (1 H, sept, $^3J_{HH}$ = 6.16 Hz, CH (ester)); 3.96-4.19 (4 H, m, CH (Ala), $OCH_2$ (ester), CH (2´)); 4.22-4.41 (4 H, m, CH (4´), CH (3´), $CH_2$ (5´)); 5.54 (1 H, dd, $^2J_{HP}$ or $^3J_{HH}$ = 11.58 Hz, $^2J_{HP}$ or $^3J_{HH}$ = 9.91 Hz, NH (Ala)); 5.79 (0.5 H, d, $^3J_{HH}$ = 3.92 Hz, $CH_a$ (1´)); 5.81 (0.5 H, d, $^3J_{HH}$ = 4.08; $CH_b$ (1´)); 6.32 (0.5 H, d, $^3J_{HH}$ = 7.60 Hz, $CH_a$ (5)); 6.33 (0.5 H, d, $^3J_{HH}$ = 7.60 Hz, $CH_b$ (5)); 7.12-7.21 (3 H, m, aromat. CH); 7.28-7.33 (2.5 H, m, aromat. CH, $CH_a$ (6)); 7.35 (0.5 H, d, $^3J_{HH}$ = 7.68 Hz, $CH_b$ (6)); 10.99 (1 H, s, NH) ppm. $^{13}$C-NMR ($CDCl_3$, 101 MHz): δ 11.08, 11.12, 11.13 2x $CH_3$ (ester); 21.05-21.13 (2x d, $CH_3$ (Ala)); 23.26, 23.29 2x $CH_2$ (ester); 40.30 CH (Esther), 50.47 (d, $^2J_{CP}$ = 12.35, CH (Ala)); 65.83 (d, $^2J_{CP}$ = 5.09 Hz, $C_a$ (5´)); 66.06 (d, $^2J_{CP}$ = 5.47 Hz, $C_b$ (5´)); 67.97, 68.05 $OCH_2$ (ester); 69.83 C (3´); 74.81 C (2´); 82.80 (d, $^3J_{CP}$ = 7.27 Hz, $C_a$ (4´)); 82.94 (d, $^3J_{CP}$ = 7.26 Hz, $C_b$ (4´)); 90.36, 90.68 C (1´); 113.99 C (5); 120.06-120.19 (2x d, aromat. C), 125.38, 125.46, 130.01 aromat C; 134.68, 134.92 C (6); 148.69, 148.70 C (2); 150.47 (d, $^2J_{CP}$ = 5.90 Hz, aromat. $C_{q,a}$); 150.54 (d, $^2J_{CP}$ = 5.81 Hz, aromat. $C_{q,b}$); 173.89 (d, $^3J_{CP}$ = 7.27 Hz, C=$O_a$ (Ala)); 173.95 (d, $^3J_{CP}$ = 6.54 Hz, C=$O_b$ (Ala)); 189.91 C (4) ppm. $^{31}$P-NMR ($CDCl_3$, 162 MHz): δ 2.84, 3.05 ppm (2 diastereomers).

Note: ***C***H (Ala) appears as doublet while we expect 2xd due to the presence of 2 diastereomers along with coupling to the adjacent phosphorous. The observed signal was assigned as a $^2J_{CP}$ coupling because an overlay of diasteromeric signals is more likely than $^2J_{CP}$ coupling, which is not resolved.

$^{1}$H-NMR (400 MHz, $CDCl_3$) of compound **12c**

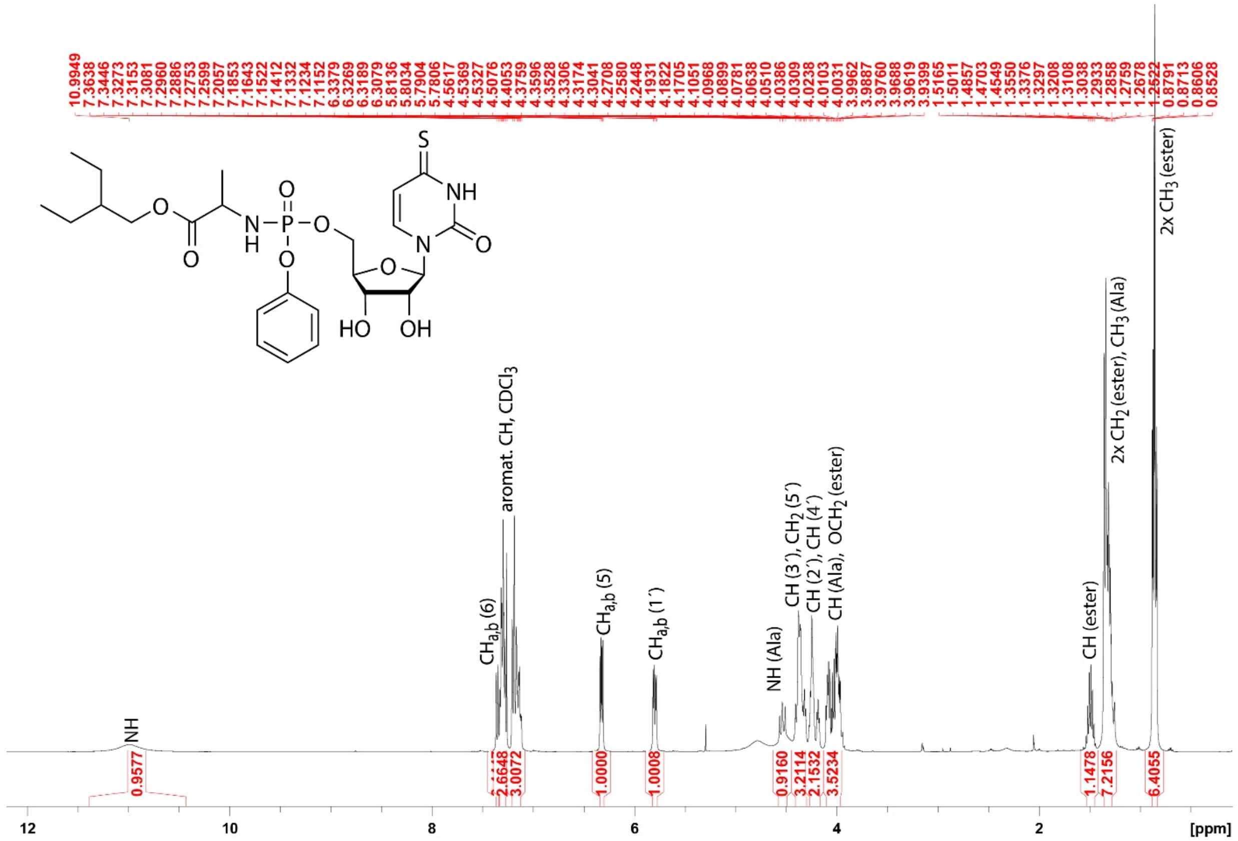


$^{13}$C-NMR (101 MHz, $CDCl_3$) of compound **12c**

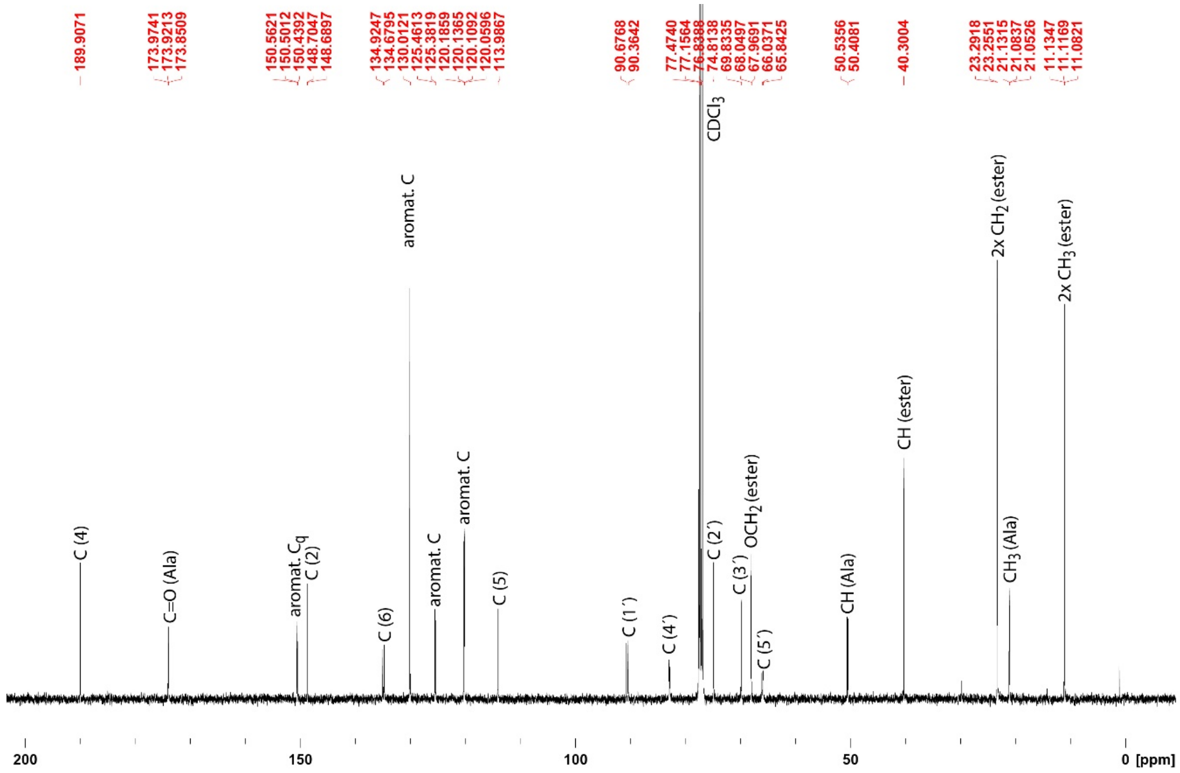

$^{31}$P-NMR (162 MHz, $CDCl_3$) of compound **12c**

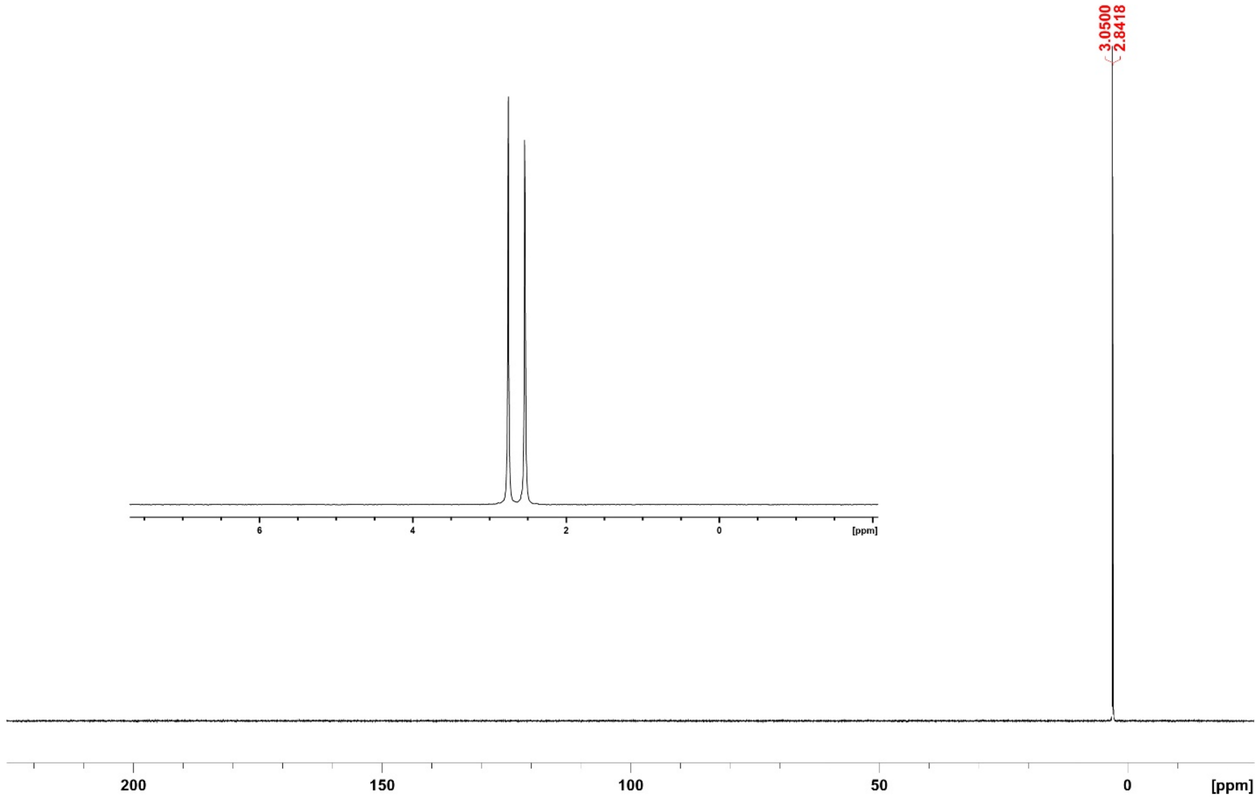

### 5. Synthesis of 4sU prodrugs with *S*[4]-biolabile protecting group (18a-c)

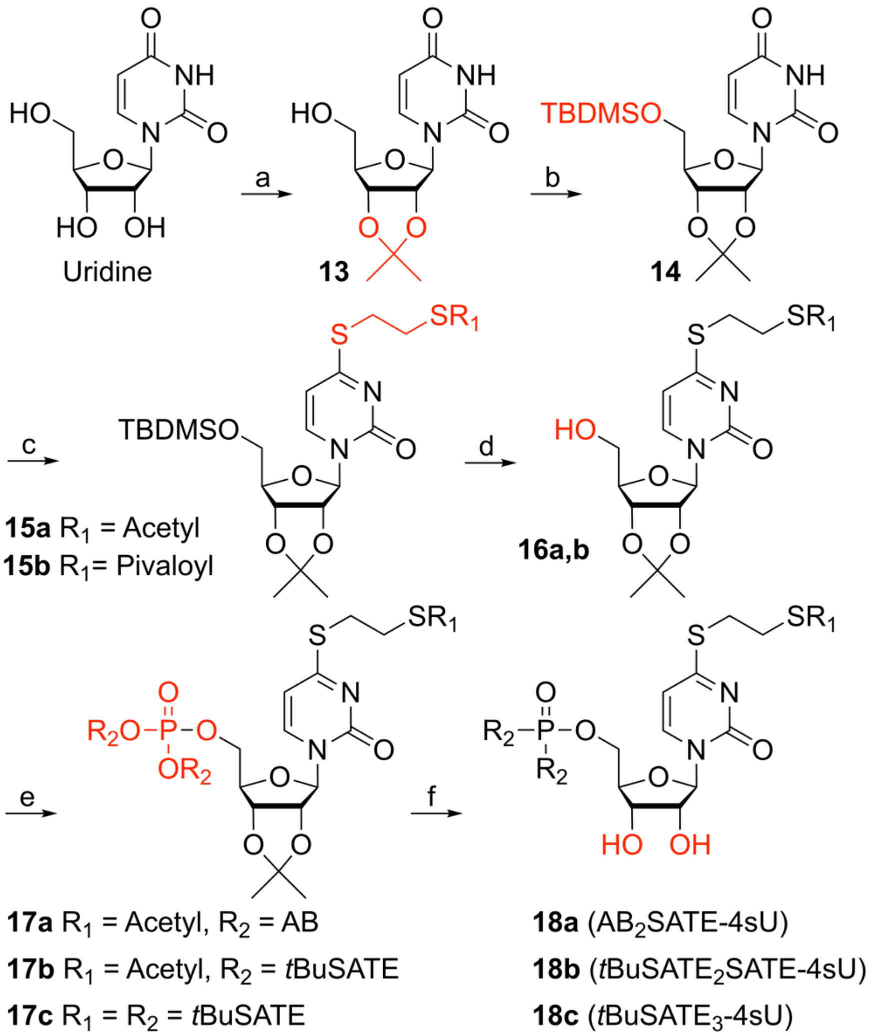


**Overview of the synthesis of compounds 18a-c.** Reaction conditions: **a)** 1.1 eq p-TsOH, acetone, 3 h, RT, 93%. **b)** 4.0 eq imidazole, 2.0 eq TBDMSCl, DMF, 16 h, room temperature (RT), 91%. **c)** i) 0.1 eq DMAP, 4.0 eq $Et_3N$, 1.3 eq TPSCl, $CH_2Cl_2$, 1 h, RT; ii) 10.0 eq *N*-methylpyrrolidine, iii) for **15a**: 5.0 eq ethanedithiol monoacetate, 16 h, 0 °C-RT, 67%. for **15b**: 5.0 eq ethanedithiol monopivaloate, 5 h, RT, 57%. **d)** 1 M TBAF, 0.5 M HOAc, 2 h, RT, **16a** (69%), **16b** (91%). **e)** for **17a**: i) 1.1 eq 5-(benzylthio)-1*H*-tetrazole (BTT), 1.1 eq compound **I**, acetonitrile, 1 h, RT ii) 1.1 eq mCPBA, 5 min, 0 °C, 65%. for **17b**: i) 1.4 eq BTT, 1.4 eq compound **II**, acetonitrile, 30 min, RT ii) 1.4 eq mCPBA, 5 min, 0 °C, 72%. for **17c**: i) 1.1 eq BTT, 1.1 eq compound **II**, acetonitrile, 1 h, RT ii) 1.1 eq mCPBA, 5 min, 0 °C, 62%. **f)** formic acid, 4 h, **18a** (23%), **18b** (23%), **18c** (34%). Overall yield: **18a** (6%), **18b** (7%), **18c** (9%).

### 5.1. Synthesis of 2´,3´-*O*-isopropylidene-uridine (13) [13]

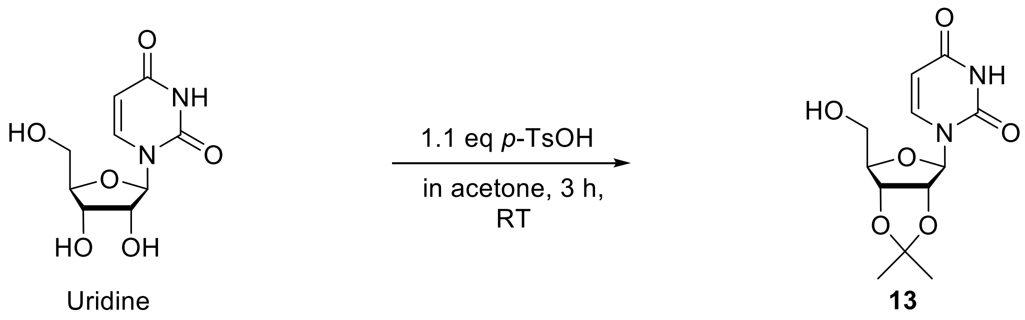

Uridine (1.5 g, 6.14 mmol) was suspended in acetone (30.0 ml) and *p*-toluenesulfonic acid (1.1 eq, 6.76 mmol, 1.285 g) was added. The reaction mixture was stirred 3 h at ambient temperature followed by neutralization with saturated sodium bicarbonate solution. Acetone was removed under reduced pressure and the remaining residue was dissolved in ethyl acetate and was washed three times with saturated sodium bicarbonate solution. The aqueous phase was subsequently extracted several times with ethyl acetate. The combined organic phases were dried over sodium sulfate. Column chromatography (4-6% MeOH in $CH_2Cl_2$) yields compound **13**. Yield: 1.621 g of **13** as white foam (93%). TLC (10% MeOH in $CH_2Cl_2$): 0.57. $^1$H-NMR (d$_6$-DMSO, 400 MHz): δ 1.29, 1.48 (6 H 2x 3 H, 2x s, 2x $CH_3$ (isopropyliden)); 3.57 (2 H, m, $CH_2$ (5´)); 4.06 (1 H, dd, $^3J_{HH}$ = 8.21 Hz, $^3J_{HH}$ = 4.38 Hz, CH (4´)); 4.74 (1 H, dd, $^3J_{HH}$ = 6.32 Hz, $^3J_{HH}$ = 3.56 Hz, CH (3´)); 4.89 (1 H, dd, $^3J_{HH}$ = 6.34 Hz, $^3J_{HH}$ = 2.65 Hz, CH (2´)); 5.07 (1 H, t, $^3J_{HH}$ = 5.29 Hz, OH (5´)); 5.63 (1 H, dd, $^3J_{HH}$ = 8.00 Hz, $^4J_{HH}$ = 1.13 Hz, CH (5)); 5.83 (1 H, d, $^3J_{HH}$ = 2.64 Hz, CH (1´)); 7.79 (1 H, d, $^3J_{HH}$ = 8.07 Hz, CH (6)); 11.37 (1 H, s, NH)) ppm.

<sup></sup>

$^{1}$H-NMR (400 MHz, $d_6$-DMSO) of compound **13**

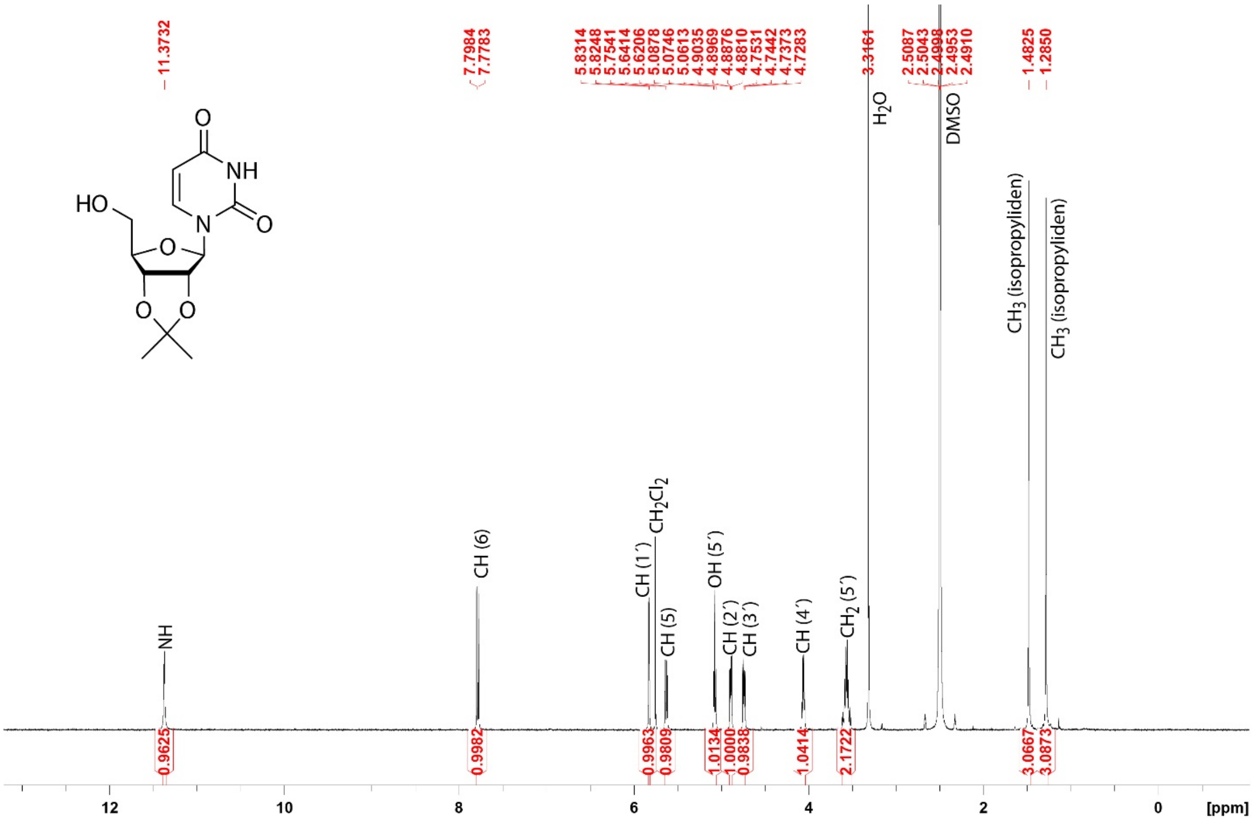

### 5.2. Synthesis of 5´-*O*-(*tert*-butyldimethylsilyl)-2´,3´-*O*-isopropylidene-uridine (14)

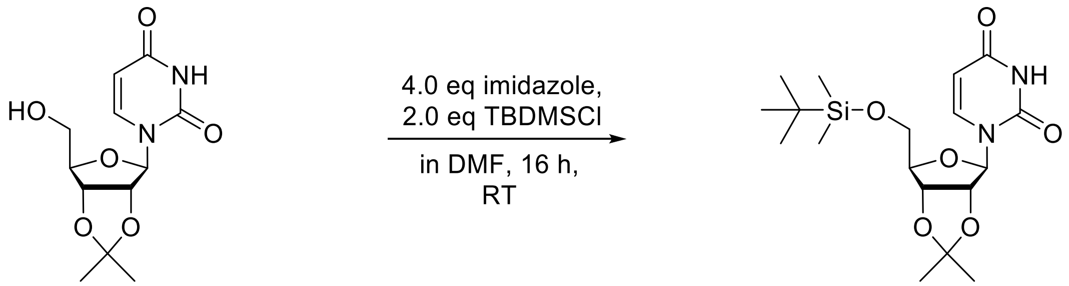


**13** **14**

Compound **13** (1.621 g, 5.70 mmol) was dissolved in *N,N*-dimethylformamide (24.9 ml) and imidazole (4.0 eq, 22.81 mmol, 1.553 g) as well as *tert*-butyldimethylsilyl chloride (2.0 eq, 11.41 mmol, 1.719 g) were introduced to the reaction mixture. The solution was stirred at ambient temperature for 16 h followed by the removal of the solvent under high vacuum. The oily residue was dissolved in dichloromethane and was washed three times with brine. The organic phase was dried over sodium sulfate and was purified by column chromatography (0-4% MeOH in $CH_2Cl_2$). Yield: 2.063 g of **14** as white foam (91%). TLC (3% MeOH in $CH_2Cl_2$): 0.41. HR-ESI-MS (m/z): $[M+H]^+$ calculated for $[C_{18}H_{31}N_2O_6Si]^+$: 399.1946; found: 399.1918. $^1$H-NMR ($CDCl_3$, 400 MHz): δ -0.086, 0.092 (6 H, 2x s, 2x $CH_3$ (TBDMS)); 0.90 (9 H, s, *t*-Bu (TBDMS)); 1.36, 1.59 (6 H, 2x s, 2x $CH_3$ (isopropyliden)); 3.80 (1 H, dd, $^2J_{HH}$ = 11.52 Hz, $^3J_{HH}$ = 2.95 Hz, $CH_a$ (5´)); 3.92 (1 H, dd, $^2J_{HH}$ = 11.52 Hz, $^3J_{HH}$ = 2.38 Hz, $CH_b$ (5´)); 4.32 (1 H, dd, $^3J_{HH}$ = 5.43 Hz, $^3J_{HH}$ = 2.74 Hz, CH (4´)); 4.68 (1 H, dd, $^3J_{HH}$ = 6.14 Hz, $^3J_{HH}$ = 2.78 Hz, CH (2´)); 4.76 (1 H, dd, $^3J_{HH}$ = 6.15 Hz, $^3J_{HH}$ = 2.98 Hz, CH (3´)); 5.68 (1 H, dd, $^3J_{HH}$ = 8.12 Hz, $^4J_{HH}$ = 2.11 Hz, CH (5)); 5.98 (1 H, d, $^3J_{HH}$ = 2.77 Hz, CH (1´)); 7.69 (1 H, d, $^3J_{HH}$ = 8.14 Hz, CH (6)); 8.72 (1 H, s, NH)) ppm. $^{13}$C-NMR ($CDCl_3$, 101 MHz): -5.41, -5.31 2x $CH_3$ (TBDMS); 18.47 $C_q$ (TBDMS); 25.49 $CH_3$ (isopropyliden); 25.98 *t*-Bu (TBDMS); 27.41 $CH_3$ (isopropyliden); 63.49 C (5´); 80.41 C (3´); 85.52 C (2´); 86.79 C (4´); 92.05 C (1´); 102.32 C (5); 114.28 $C_q$ (isopropyliden); 140.66 C (6); 150.14 C (2); 163.12 C (4) ppm.

$^1$H-NMR (400 MHz, $CDCl_3$) of compound **14**

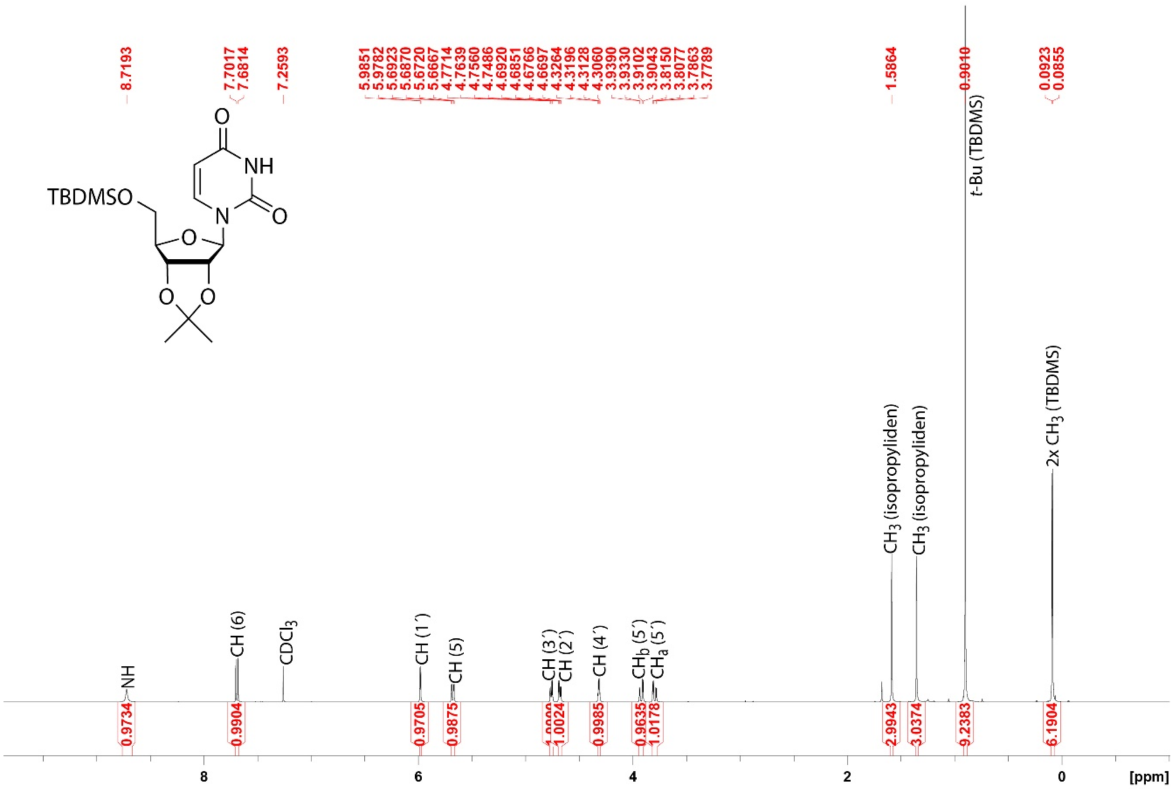


$^{13}$C-NMR (101 MHz, $CDCl_3$) of compound **14**

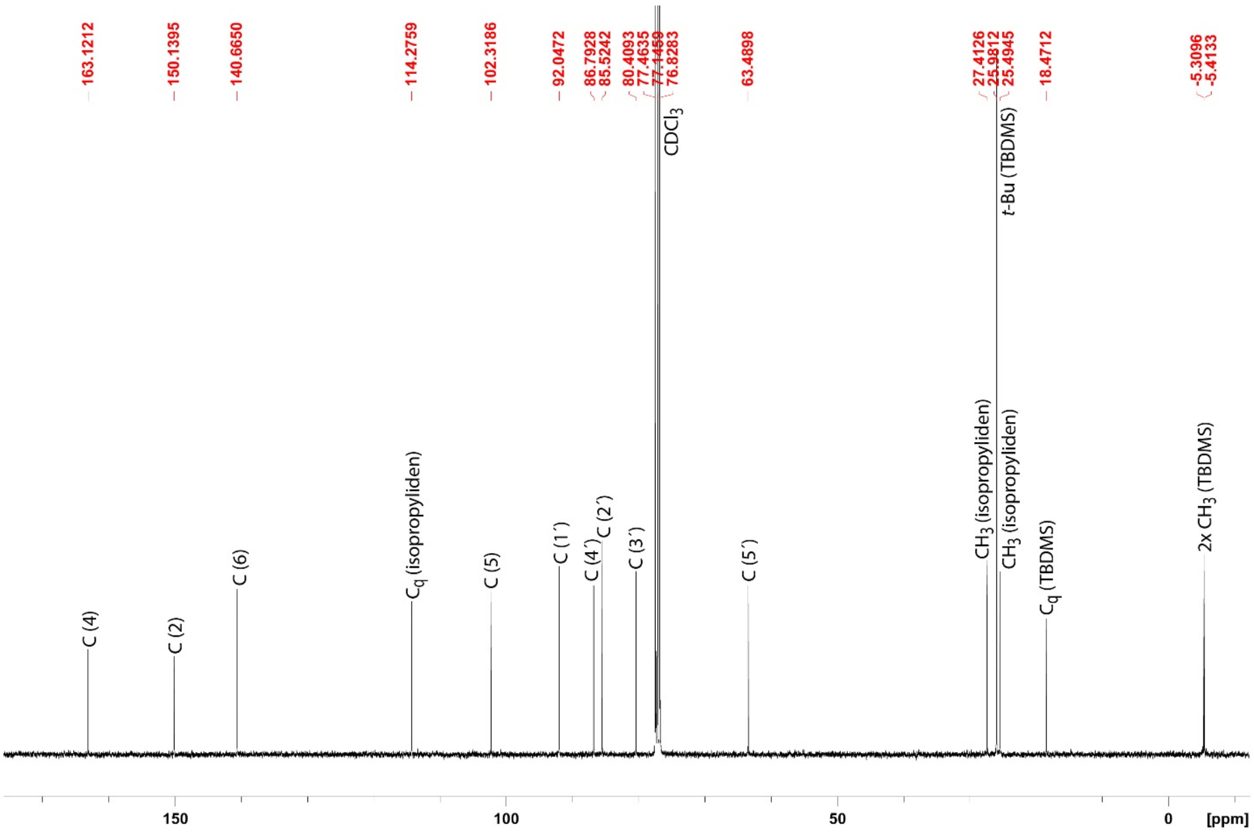

**5.3. Synthesis of 4-(*S*-(β-acetylmercapto)ethyl)-5´-*O*-(*tert*-butyldimethylsilyl)-2´,3´-*O*-isopropylidene-uridine (15a)** [14]

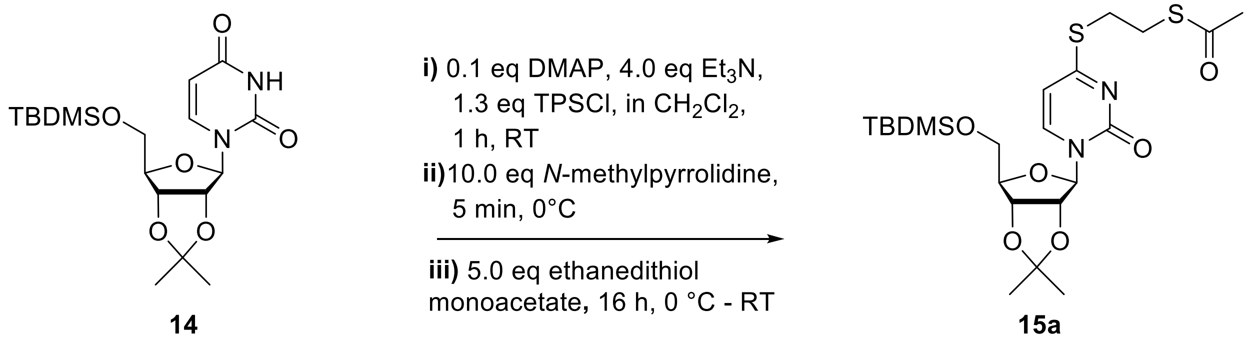


Compound **14** (1.113 g, 2.79 mmol) was dissolved in dichloromethane (7.40 ml) and treated consecutively with 4-(dimethylamino)-pyridine (0.1 eq, 0.28 mmol, 63 mg), triethylamine (4.0 eq, 11.17 mmol, 1.56 ml) and 2,4,6-triisopropylbenzesulfonyl chloride (1.3 eq, 3.63 mmol, 1.099 g). The resulting solution was stirred at ambient temperature until TLC (3% MeOH in $CH_2Cl_2$, $R_f$ = 0.81) showed a complete turnover (1 h). Activated compound **14** was then cooled to 0 °C and treated with *N*-methylpyrrolidine (10.0 eq, 27.9 mmol, 2.90 ml) and stirred at 0 °C for 5 min. Ethanedithiol monoacetate [15] (5.0 eq, 13.9 mmol, 1.902 g) was introduced to the reaction mixture which was stirred for another 30 min at 0 °C. Stirring was continued at ambient temperature for 16 h followed by dilution with dichloromethane and extraction with saturated sodium bicarbonate solution. The crude product was purified by column chromatography (0-2% MeOH in $CH_2Cl_2$). Yield: 969 mg of **15a** as colorless oil (67%). TLC (ethyl acetate in cyclohexane 1:1): 0.63. HR-ESI-MS (m/z): $[M+H]^+$ calculated for $[C_{22}H_{37}N_2O_6S_2Si]^+$: 517.1857; found: 517.1828. $^1$H-NMR ($CDCl_3$, 400 MHz): δ -0.064 (6 H, s, 2x $CH_3$ (TBDMS)); 0.87 (9 H, s, *t*-Bu (TBDMS)); 1.35, 1.59 (6 H, 2x s, 2x $CH_3$ (isopropyliden)); 2.34 (3 H, s, $SOCH_3$); 3.21 (2 H, m, $CH_2SCOCH_3$); 3.28-3.45 (2 H, m, $SCH_2$); 3.80 (1 H, dd, $^2J_{HH}$ = 11.60 Hz, $^3J_{HH}$ = 3.39 Hz, $CH_a$ (5´)); 3.95 (1 H, dd, $^2J_{HH}$ = 11.59 Hz, $^3J_{HH}$ = 2.51 Hz, $CH_b$ (5´)); 4.35 (1 H, m, CH (4´)); 4.74 (2 H, m, CH (2´), CH (3´)); 5.94 (1 H, s, CH (1´)); 6.14 (1 H, d, $^3J_{HH}$ = 7.11 Hz, CH (5)); 7.84 (1 H, d, $^3J_{HH}$ = 7.12 Hz, CH (6)) ppm. $^{13}$C-NMR ($CDCl_3$, 101 MHz): -5.39, -5.28 2x $CH_3$ (TBDMS); 18.46 $C_q$ (TBDMS); 25.46 $CH_3$ (isopropyliden); 25.99 *t*-Bu (TBDMS); 27.38 $CH_3$ (isopropyliden);28.78 $CH_2SCOCH_3$; 29.50 $SCH_2$; 30.74 $SCOCH_3$; 63.32 C (5´); 80.01 C (3´); 86.28 C (2´); 88.14 C (4´); 93.81 C (1´); 103.23 C (5); 113.97 $C_q$ (isopropyliden); 140.74 C (6); 153.70 C (2); 176.84 C (4), 195.57 C=O ppm.

$^{1}$H-NMR (400 MHz, CDCl3) of compound **15a**

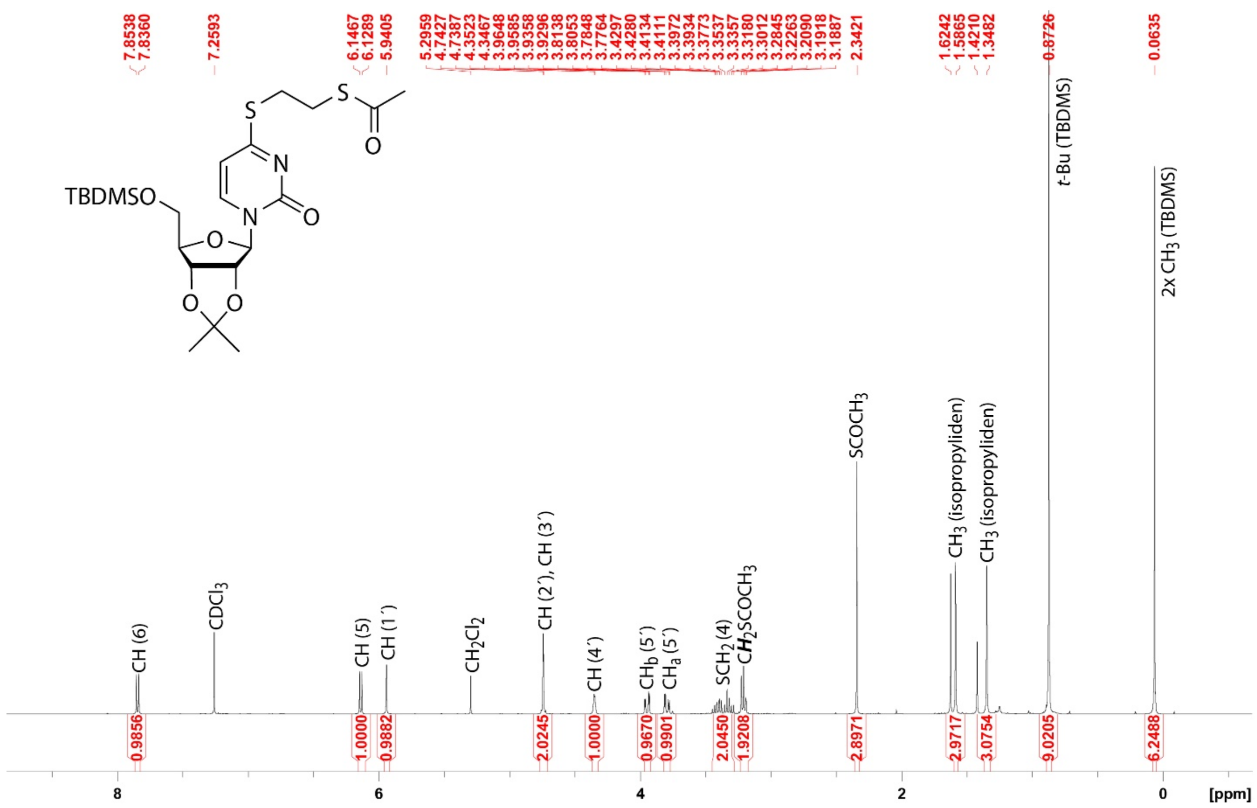

$^{13}$C-NMR (101 MHz, CDCl3) of compound **15a**

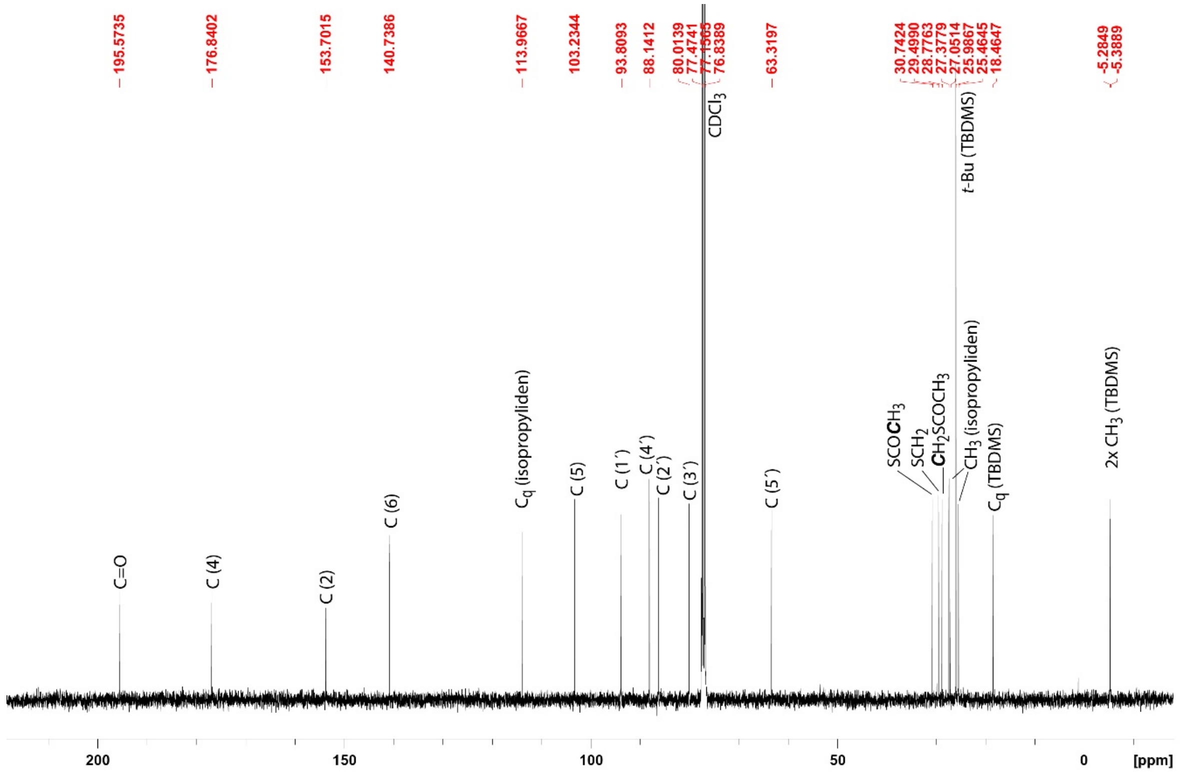

### 5.4. Synthesis of 5´-*O*-(*tert*-butyldimethylsilyl)-2´,3´-*O*-isopropylidene-4-(*S*-(β-pivaloylmercapto)ethyl)-uridine (15b) [14]

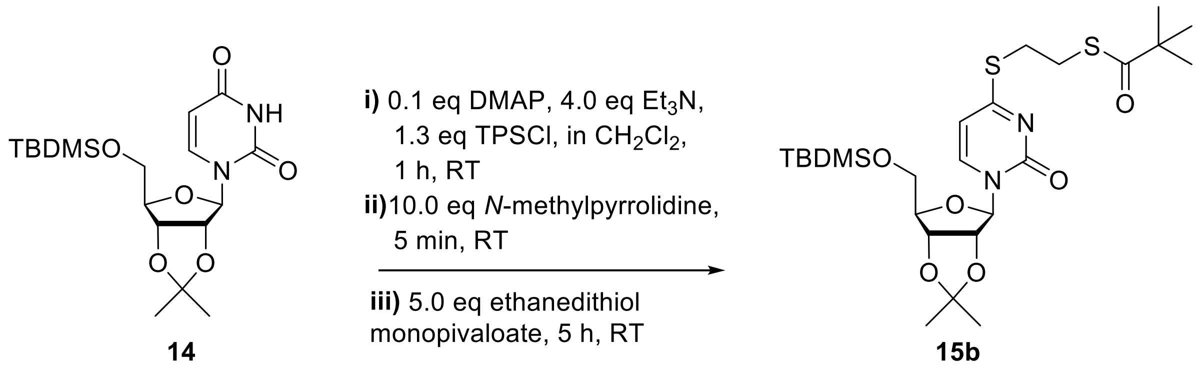


Compound **14** (405 mg, 1.02 mmol) was dissolved in dichloromethane (3.4 ml) and treated consecutively with 4-(dimethylamino)-pyridine (0.1 eq, 0.10 mmol, 12 mg), triethylamine (4.0 eq, 4.06 mmol, 0.57 ml) and 2,4,6-triisopropylbenzesulfonyl chloride (1.3 eq, 1.32 mmol, 400 mg). The resulting solution was stirred at ambient temperature until TLC (3% MeOH in $CH_2Cl_2$, $R_f$ = 0.81) showed a complete turnover (1 h). Activated compound **14** was then treated with *N*-methylpyrrolidine (10.0 eq, 10.16 mmol, 1.06 ml) and stirred for 5 min. Ethanedithiol monopivaloate [16] (5.0 eq, 5.08 mmol, 906 mg) was introduced to the reaction mixture which was stirred for another 5 h at ambient temperature. The solution was diluted with dichloromethane, was washed with saturated sodium bicarbonate solution and dried over sodium sulfate. The crude product was purified by column chromatography (0-3% MeOH in $CH_2Cl_2$). Yield: 334 mg of **15b** as colorless oil (57%). TLC (3% MeOH in $CH_2Cl_2$): 0.50. HR-ESI-MS (m/z): $[M+H]^+$ calculated for $[C_{25}H_{43}N_2O_6S_2Si]^+$: 559.2326; found: 559.2296. $^1$H-NMR ($CDCl_3$, 400 MHz): δ -0.063 (6 H, s, 2x $CH_3$ (TBDMS)); 0.87 (9 H, s, *t*-Bu (TBDMS)); 1.23 (9 H, s, $CH_2SCOC(CH_3)_3$); 1.35, 1.58 (6 H, 2x s, 2x $CH_3$ (isopropyliden)); 3.16 (2 H, m, $CH_2SCOC(CH_3)_3$); 3.26-3.43 (2 H, m, $SCH_2$); 3.79 (1 H, dd, $^2J_{HH}$ = 11.58 Hz, $^3J_{HH}$ = 3.41 Hz, $CH_a$ (5´)); 3.95 (1 H, dd, $^2J_{HH}$ = 11.58 Hz, $^3J_{HH}$ = 2.54 Hz, $CH_b$ (5´)); 4.34 (1 H, dd, $^3J_{HH}$ = 5.15 Hz, $^3J_{HH}$ = 2.64 Hz, CH (4´)); 4.74 (2 H, m, CH (2´), CH (3´)); 5.94 (1 H, d, $^3J_{HH}$ = 0.79 Hz, CH (1´)); 6.14 (1 H, d, $^3J_{HH}$ = 7.11 Hz, CH (5)); 7.84 (1 H, d, $^3J_{HH}$ = 7.13 Hz, CH (6)) ppm. $^{13}$C-NMR ($CDCl_3$, 101 MHz): -5.39, -5.29 2x $CH_3$ (TBDMS); 18.45 $C_q$ (TBDMS); 25.47 $CH_3$ (isopropyliden); 25.98 *t*-Bu (TBDMS); 27.38 $CH_3$ (isopropyliden); 27.49 $CH_2SCOC(CH_3)_3$; 28.13 $SCH_2$; 29.57 $CH_2SCOC(CH_3)_3$; 46.62 $CH_2SCOC(CH_3)_3$; 63.31 C (5´); 79.99 C (3´); 86.27 C (2´); 88.14 C (4´); 93.78 C (1´); 103.22 C (5); 113.95 $C_q$ (isopropyliden); 140.68 C (6); 153.70 C (2); 177.01 C (4); 206.53 C=O ppm.

<sup></sup>

$^{1}$H-NMR (400 MHz, $CDCl_3$) of compound **15b**

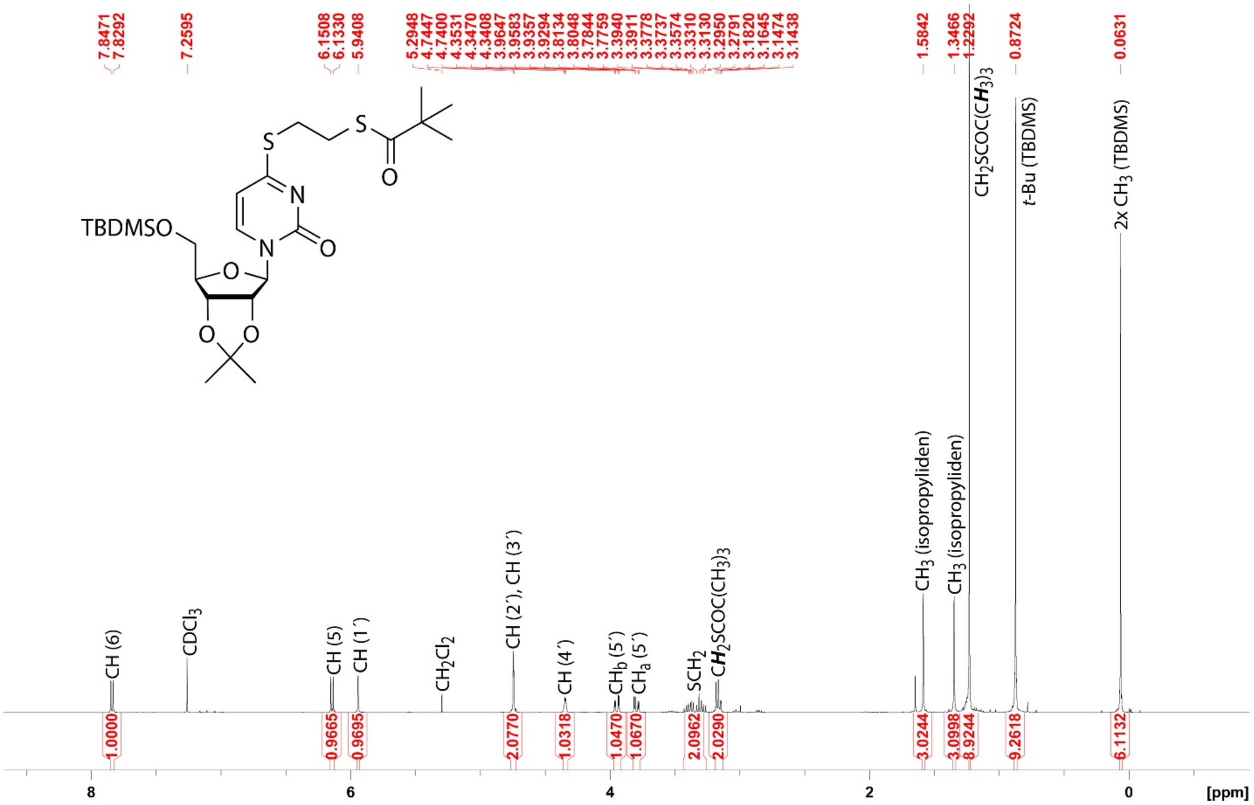


$^{13}$C-NMR (101 MHz, $CDCl_3$) of compound **15b**

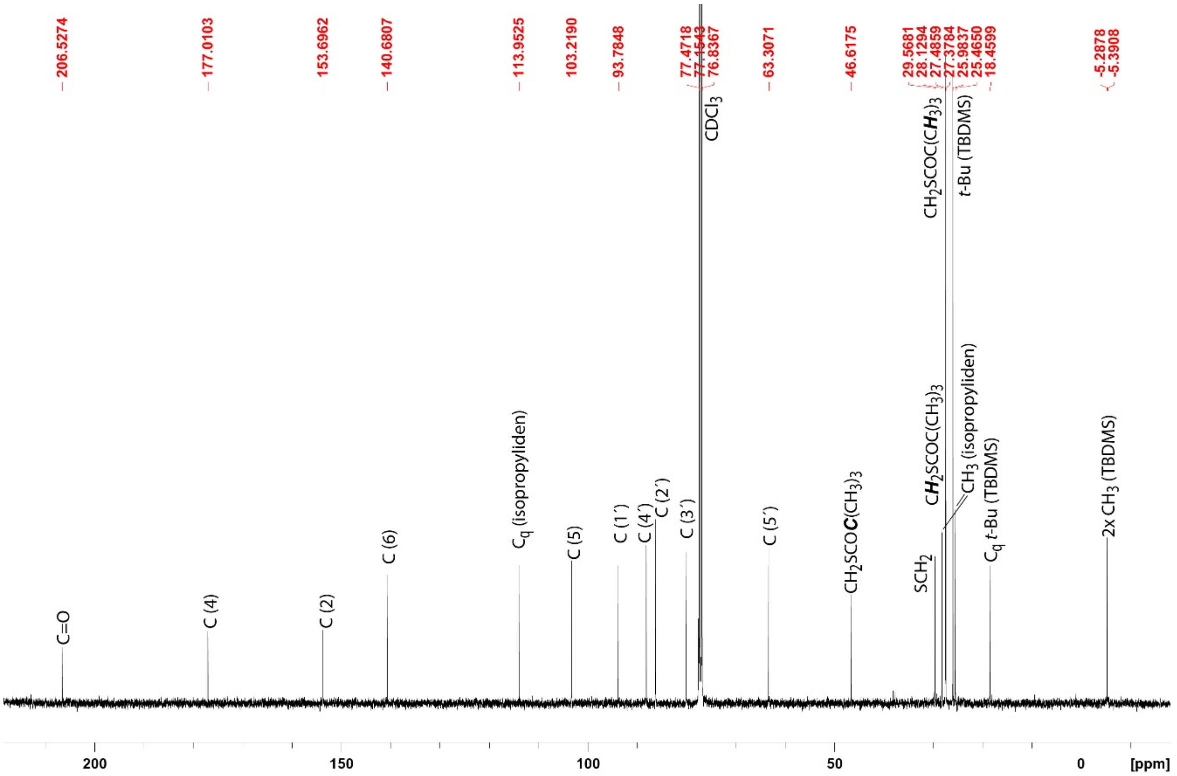

**5.5. Synthesis of 4-(*S*-(β-acetylmercapto)ethyl)-2´,3´-*O*-isopropylidene-uridine (16a)**

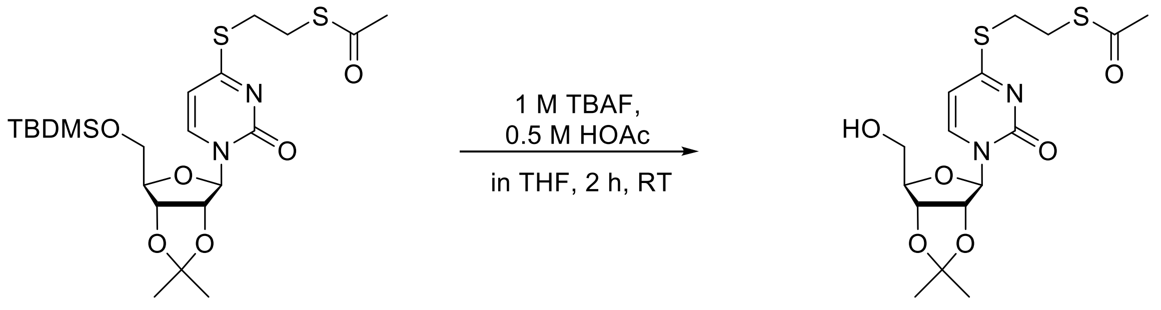


**15a** **16a**

Compound **15a** (260 mg, 0.94 mmol) was dissolved in tetrahydrofuran (3.0 ml) containing 1 M tetrabutylammonium fluoride trihydrate and 0.5 M acetic acid. Solvents were evaporated after 2 h and the crude product was purified via column chromatography (0-3% MeOH in $CH_2Cl_2$). Yield: 139 mg of **16a** as white powder (69%). TLC (3% MeOH in $CH_2Cl_2$): 0.22. HR-ESI-MS (m/z): $[M+H]^+$ calculated for $[C_{16}H_{23}N_2O_6S_2]^+$: 403.0992; found: 403.0977. $^1$H-NMR ($d_6$-DMSO, 400 MHz): 1.28, 1.49 (6 H, 2x s, 2x $CH_3$ (isopropyliden)); 2.35 $SCOCH_3$; 3.15 (2 H, m, $C\boldsymbol{H_2}SCOCH_3$); 3.28 (2 H, m, $SCH_2$(4)); 3.52-3.58 (1 H, m, $CH_a$ (5´)); 3.61-3.66 (1 H, m, $CH_b$ (5´)); 4.18 (1 H, dd, $^3J_{HH}$ = 7.96 Hz, $^3J_{HH}$ = 4.20 Hz, CH (4´)); 4.74 (1 H, dd, $^3J_{HH}$ = 6.15 Hz, $^3J_{HH}$ = 3.32 Hz, CH (3´)); 4.87 (1 H, dd, $^3J_{HH}$ = 6.22 Hz, $^3J_{HH}$ = 1.74 Hz, CH (2´)); 5.09 (1 H, t, $^3J_{HH}$ = 5.05 Hz, OH (5´)); 5.81 (1 H, d, $^3J_{HH}$ = 1.68 Hz, CH (1´)); 6.47 (1 H, d, $^3J_{HH}$ = 7.09 Hz, CH (5)); 8.05 (1 H, d, $^3J_{HH}$ = 7.12 Hz, CH (6)) ppm. $^{13}$C-NMR ($d_6$-DMSO, 101 MHz): 25.08, 26.95 2x $CH_3$ (isopropyliden); 28.00 *C*$H_2SCOCH_3$; 28.60 $SCH_2$; 30.52 SCO*C*$H_3$; 61.15 C (5´); 80.40 C (3´); 84.77 C (2´); 87.80 C (4´); 93.43 C (1´); 102.57 C (5); 112.56 $C_q$ (isopropyliden); 142.88 C (6); 152.55 C (2); 176.01 C (4); 194.84 C=O ppm.

$^{1}$H-NMR (400 MHz, $d_6$-DMSO) of compound **16a**

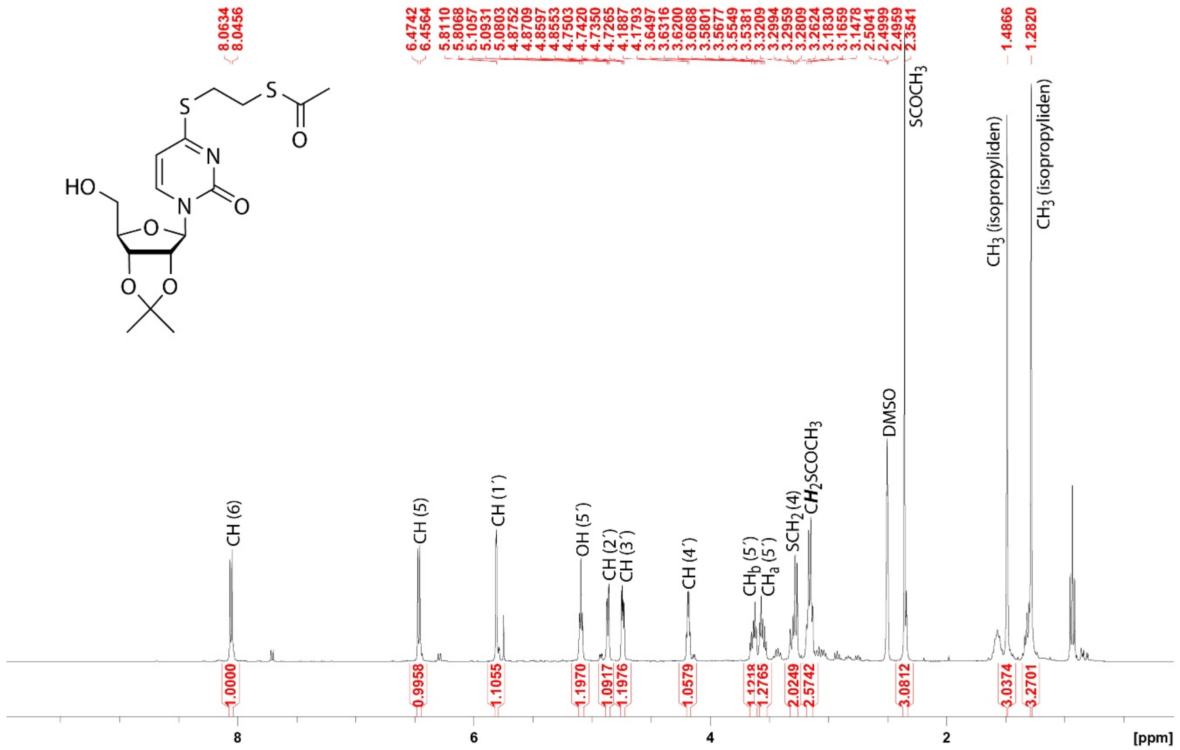


$^{13}$C-NMR (101 MHz, $d_6$-DMSO) of compound **16a**

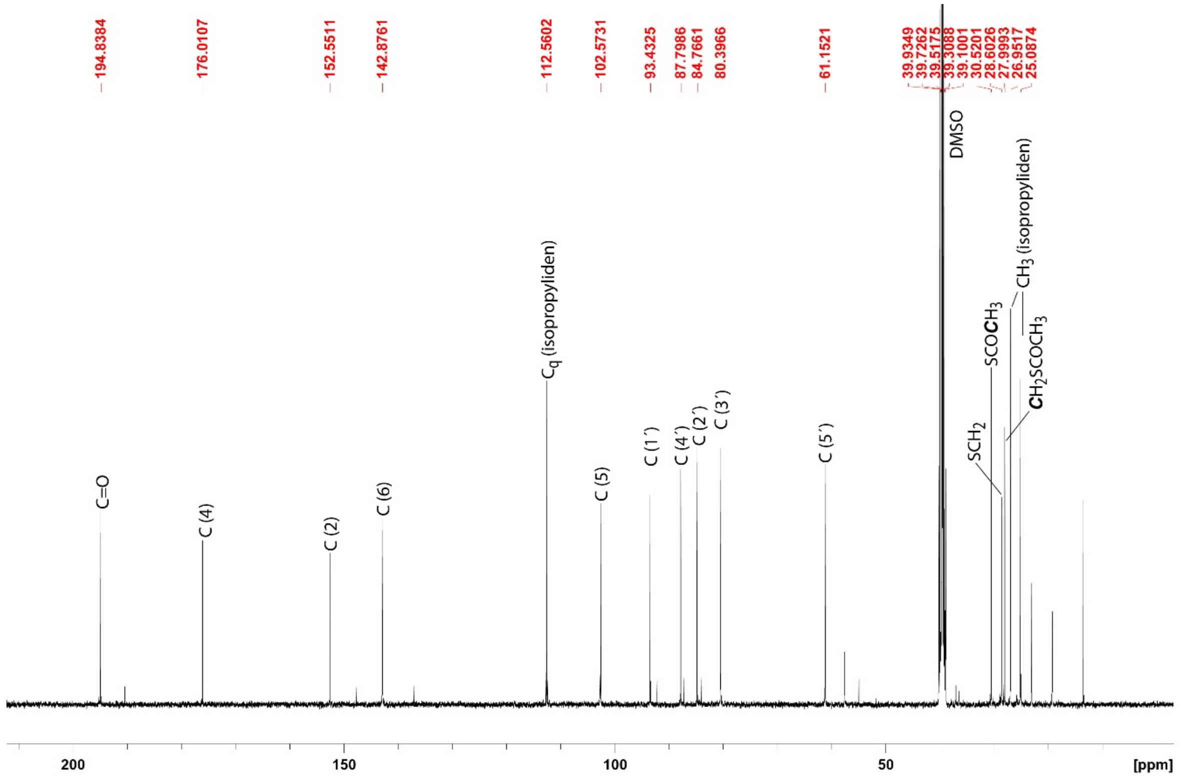

**5.6. Synthesis of 2´,3´-*O*-isopropylidene-4-(*S*-(β-pivaloylmercapto)ethyl)-uridine (16b)**

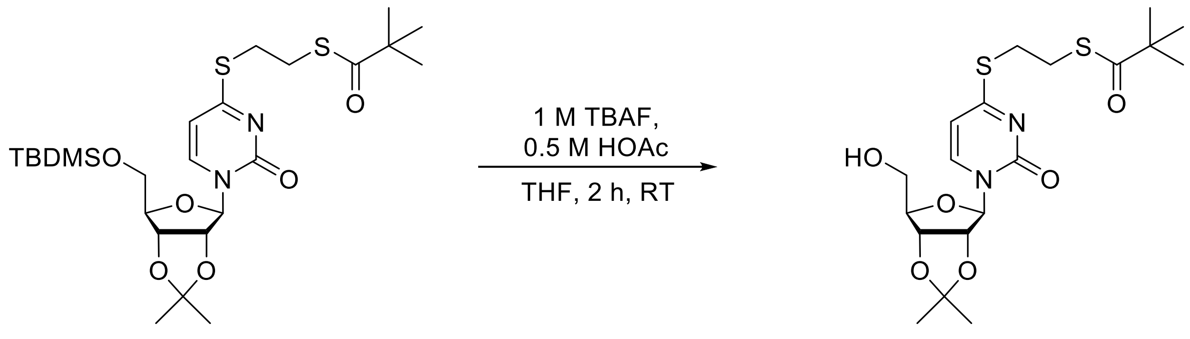


Compound **15b** (334 mg, 0.60 mmol) was dissolved in tetrahydrofuran (1.2 ml) containing 1 M tetrabutylammonium fluoride trihydrate and 0.5 M acetic acid. Solvents were evaporated after 2 h and the crude product was purified via column chromatography (2-4% MeOH in $CH_2Cl_2$). Yield: 243 mg of **16b** as white powder (91%). TLC (5% MeOH in $CH_2Cl_2$): 0.42. HR-ESI-MS (m/z): $[M+H]^+$ calculated for $[C_{19}H_{29}N_2O_6S_2]^+$: 445.1462; found: 445.1439. $^1$H-NMR ($CDCl_3$, 400 MHz): δ 1.23 (9 H, s, $CH_2SCOC(C\mathit{H}_3)_3$); 1.36, 1.56 (2x 3 H, 2x s, 2x $CH_3$ (isopropyliden)); 3.170 (2 H, t, $^3J_{HH}$ = 7.15 Hz, $C\mathit{H}_2SCOC(CH_3)_3$); 3.27-3.38 (2 H, m, $SCH_2$); 3.41 (0.5 H, d, $^3J_{HH}$ = 2.97 Hz, $OH_a$ (5´)); 3.43 (0.5 H, d, $^3J_{HH}$ = 2.92 Hz, $OH_b$ (5´)); 3.79-3.85 (1 H, m, $CH_a$ (5´)); 3.94 (1 H, txd, $^2J_{HH}$ = 12.29 Hz, $^3J_{HH}$ = 2.64 Hz, $CH_b$ (5´)); 4.34 (1 H, dd, $^3J_{HH}$ = 5.71 Hz, $^3J_{HH}$ = 3.28 Hz, CH (4´)); 5.10 (1 H, dd, $^3J_{HH}$ = 6.44 Hz, $^3J_{HH}$ = 3.45 Hz, CH (3´)); 5.25 (1 H, dd, $^3J_{HH}$ = 6.45 Hz, $^3J_{HH}$ = 2.80 Hz, CH (2´)); 5.40 (1 H, d, $^3J_{HH}$ = 2.79 Hz, CH (1´)); 6.23 (1 H, d, $^3J_{HH}$ = 7.03 Hz, CH (5)); 7.38 (1 H, d, $^3J_{HH}$ = 7.11 Hz, CH (6)) ppm. $^{13}$C-NMR ($CDCl_3$, 101 MHz): 25.35 $CH_3$ (isopropyliden); 27.44 $C\mathit{H}_2SCOC(CH_3)_3$; 27.48 $CH_2SCOC(\mathit{C}H_3)_3$; 27.95 $CH_3$ (isopropyliden); 29.85 $SCH_2$; 46.66 $CH_2SCO\mathit{C}(CH_3)_3$; 63.09 C (5´); 80.61 C (3´); 83.30 C (2´); 88.19 C (4´); 99.89 C (1´); 104.21 C (5); 114.29 $C_q$ (isopropyliden); 143.57 C (6); 153.74 C (2); 178.63 C (4); 206.46 C=O ppm.

$^{1}$H-NMR (400 MHz, $CDCl_3$) of compound **16b**

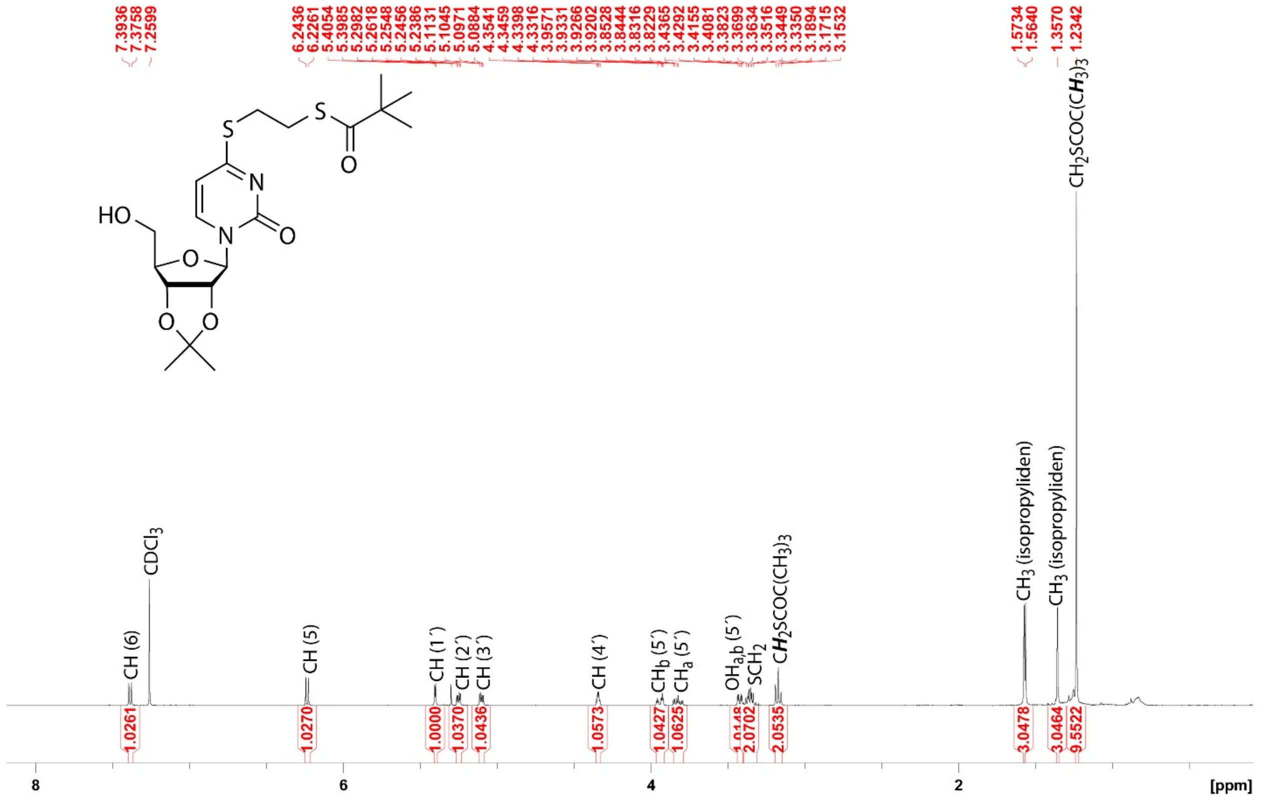


$^{13}$C-NMR (101 MHz, $CDCl_3$) of compound **16b**

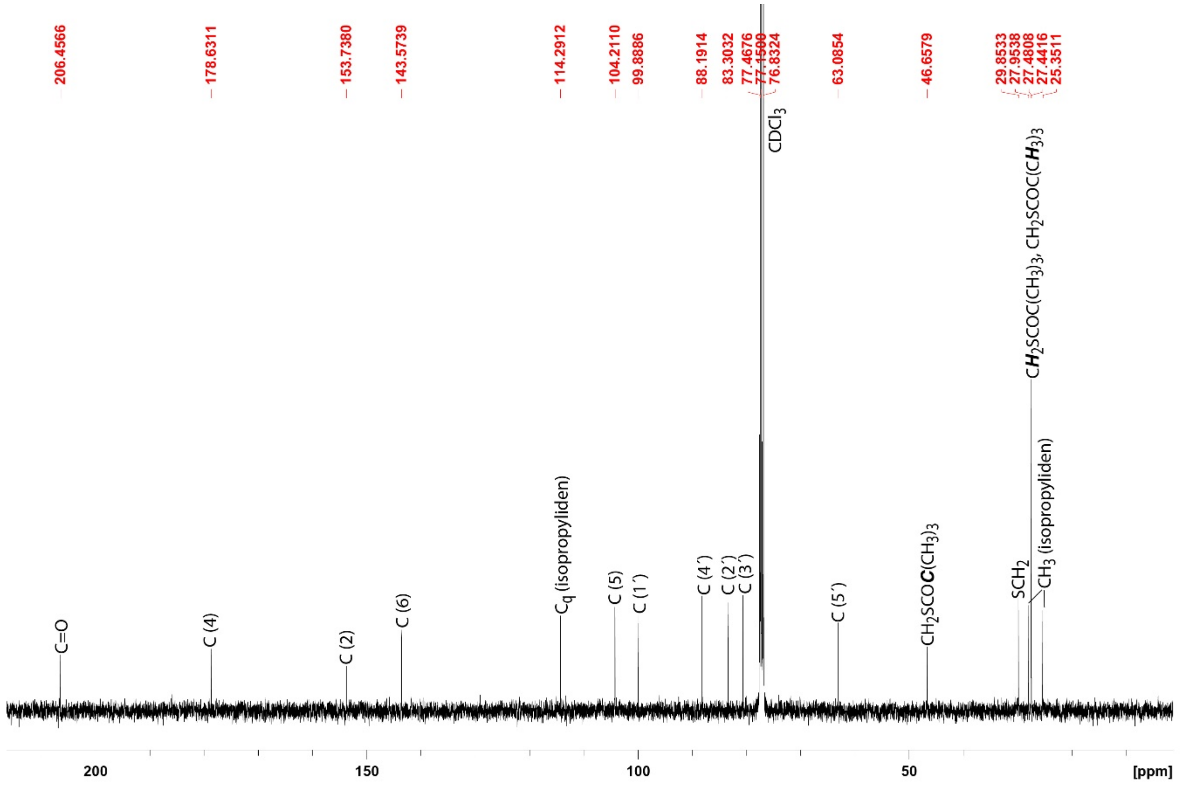

**5.7. Synthesis of bis(4-acetoxybenzyl)-5´-*O*-(4-(*S*-(β-acetylmercapto)ethyl)-2´,3´-*O*-isopropylidene-uridinyl) phosphate (17a)** [5,6]

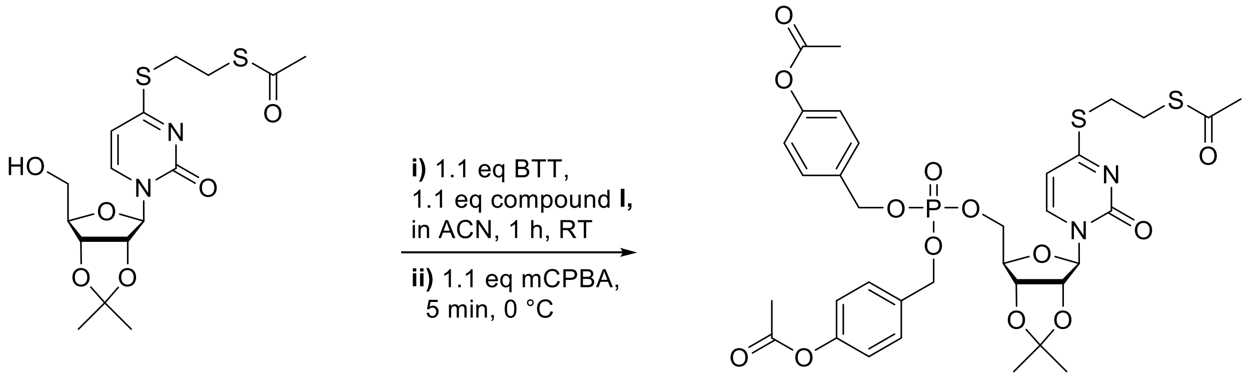


**16a** **17a**

Compound **16a** (87 mg, 0.22 mmol) and compound **I** (1.1 eq, 0.22 mmol, 10 mg) were coevaporated trice with acetonitrile and were subsequently dried under high vacuum for a few min. The oily residue was dissolved in a 0.3 M BTT-solution (1.1 eq, 0.79 ml 0.3 M in acetonitrile; dried over molecular sieves) and was stirred at room temperature for 60 min The reaction mixture was then cooled to 0 °C and meta-chloroperbenzoic acid (1.1 eq, 0.22 mmol, 41 mg) was added carefully. Solvent was evaporated after 5 min and the residue was subjected to column chromatography (0-3% MeOH in dichloromethane). Yield: 109 mg of **17a** as white foam (65%). TLC (4% MeOH in $CH_2Cl_2$): 0.44. HR-ESI-MS (m/z): $[M+H]^+$ calculated for $[C_{34}H_{40}N_2O_{13}PS_2]^+$: 779.1704; found: 779.1656. $^1$H-NMR ($CDCl_3$, 400 MHz): δ 1.28, 1.51 (2x 3 H, 2x s, 2x $CH_3$ (isopropyliden)); 2.26 (6 H, s, 2x Acetyl (AB)); 2.30 (3 H, s, $SCOCH_3$); 3.16 (2 H, t, $^3J_{HH}$ = 7.18 Hz, C*H*2SCOCH3); 3.24-3.35 (2 H, m, $SCH_2$); 4.20-4.29 (2 H, m, $CH_2$ (5´)); 4.32 (1 H, dd, $^3J_{HH}$ =8.90 Hz, $^3J_{HH}$ = 4.45 Hz, CH (4´)); 4.76 (1 H, dd, $^3J_{HH}$ = 6.26 Hz, $^3J_{HH}$ = 4.02 Hz, CH (3´)); 4.90 (1 H, dd, $^3J_{HH}$ = 6.37 Hz, $^3J_{HH}$ = 1.29 Hz, CH (2´)); 4.97 (4 H, d, $^3J_{HP}$ = 8.52 Hz, 2x $CH_2$ (AB)); 5.63 (1 H, d, $^3J_{HH}$ = 1.38 Hz, CH (1´)); 6.07 (1 H, d, $^3J_{HH}$ = 7.06 Hz, CH (5)); 7.02-7-05, 7.30 (8 H, m, aromat. CH (AB)); 7.40 (1 H, d, $^3J_{HH}$ = 7.11 Hz, CH (6)) ppm. $^{13}$C-NMR ($CDCl_3$, 101 MHz): δ 21.12 2x Acetyl (AB); 25.19, 27.08 2x $CH_3$ (isopropyliden); 28.48 *C*H2SCOCH3; 29.47 $SCH_2$; 30.59 SCO*C*H3; 67.32 (d, $^2J_{CP}$ = 5.81 Hz, C (5´)); 68.83-68.90 (2x d, 2x $CH_2$ (AB)); 80.87 C (3´); 84.99 C (2´); 86.80 (d, $^3J_{CP}$ = 7.26 Hz, C (4´)); 96.33 C (1´); 103.66 C (5); 114.22 $C_q$ (isopropyliden); 121.86, 129.23 aromat. C (AB), 133.16 (d, $^3J_{CP}$ = 7.23 Hz, $C_q$ (AB)); 142.09 C (6); 150.86 $C_q$ (AB); 153.26 C (2); 169.24 2x C=O (AB); 177.72 C (4); 195.28 S*C*OCH3 ppm. $^{31}$P-NMR ($CDCl_3$, 162 MHz): δ -1.09 ppm.

$^{1}$H-NMR (400 MHz, $CDCl_3$) of compound **17a**

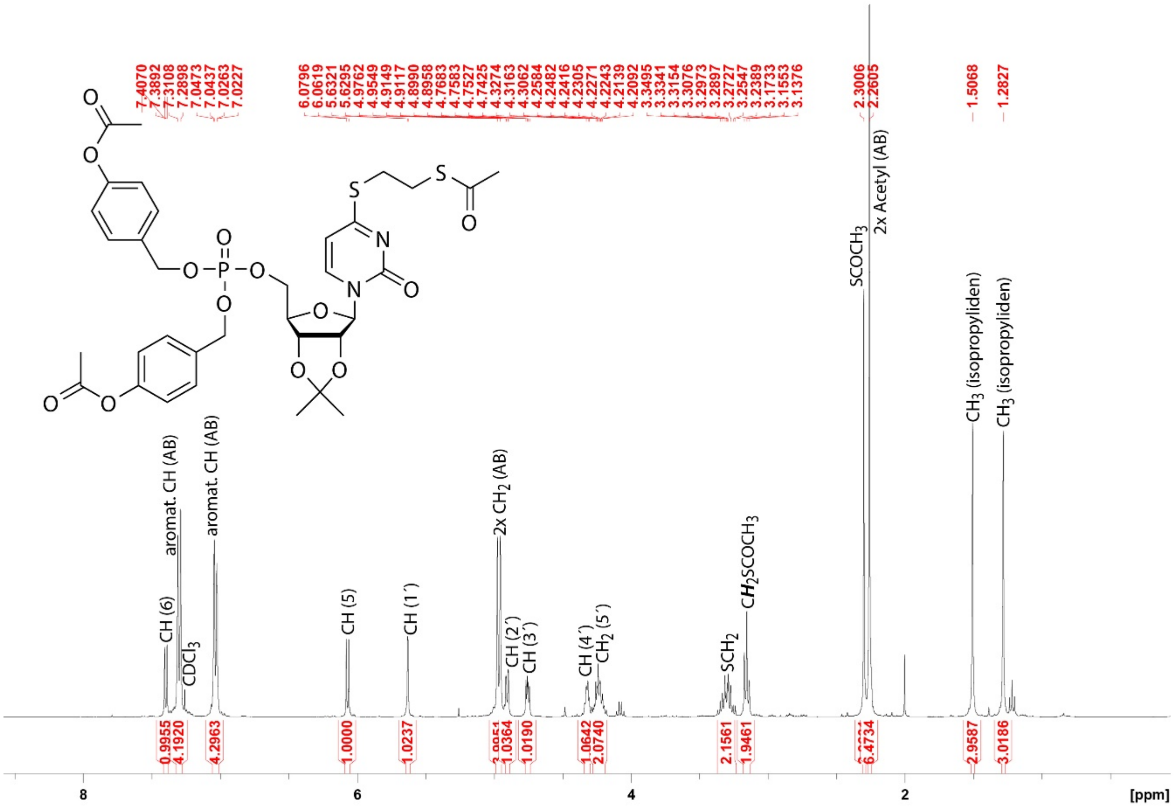


$^{13}$C-NMR (101 MHz, $CDCl_3$) of compound **17a**

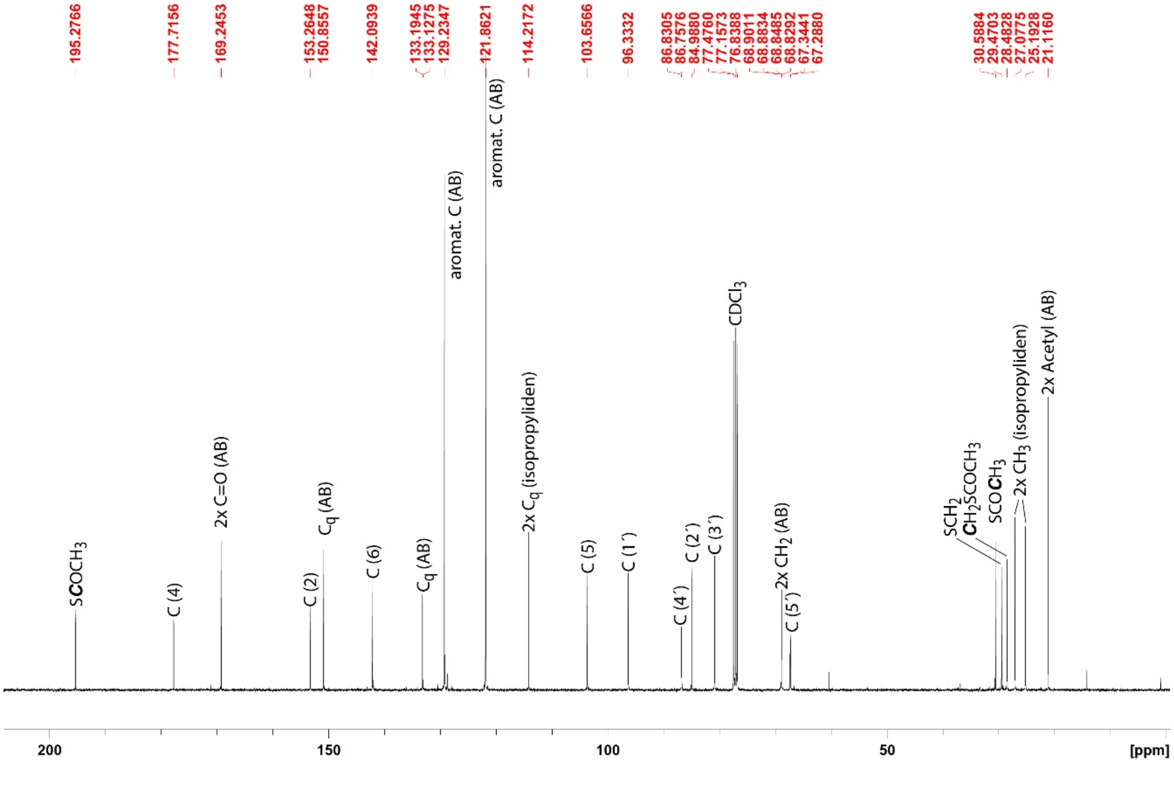

$^{31}$P-NMR (162 MHz, $CDCl_3$) of compound **17a**

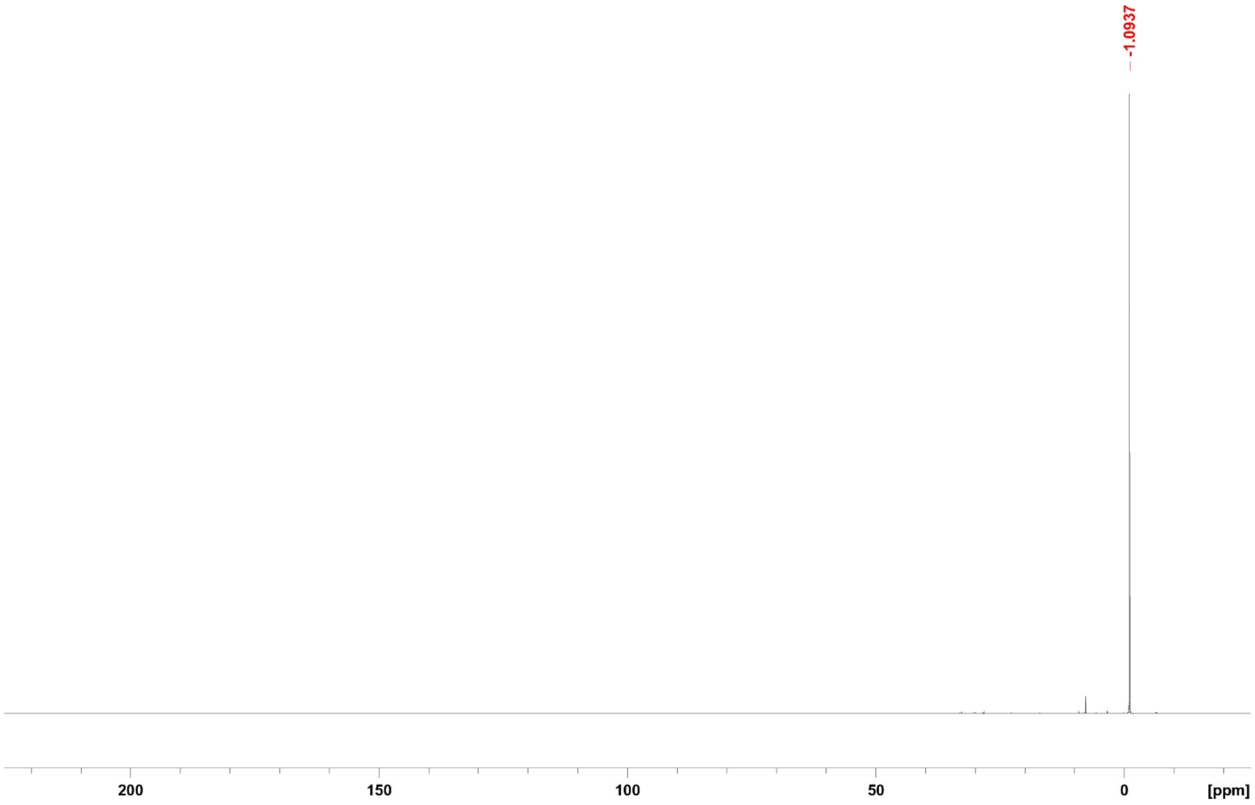

### 5.8. Synthesis of bis(*S*-pivaloyl-2-thioethyl)-5´-*O*-(4-(*S*-(β-acetylmercapto)ethyl)-2´,3´-*O*-isopropylidene-uridinyl) phosphate (17b) [5,6]

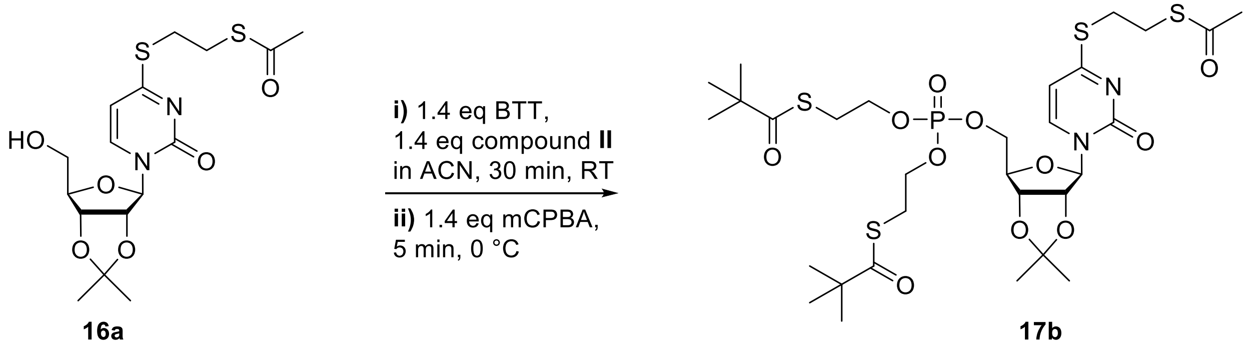


**16a** **17b**

Compound **16a** (90 mg, 0.22 mmol) and compound **II** (1.4 eq, 0.31 mmol, 142 mg) were coevaporated trice with acetonitrile and were subsequently dried under high vacuum . The oily residue was dissolved in acetonitrile containing 0.3 M 5-(benzylthio)-1*H*-tetrazole (1.1 eq, 1.04 ml, dried over molecular sieves) and was stirred at room temperature until TLC showed full conversion (30 min). The reaction mixture was then cooled to 0 °C and meta-chloroperbenzoic acid (1.4 eq, 0.31 mmol, 54 mg) was added carefully. Solvent was evaporated after 5 min and the residue was subjected to column chromatography (0-3% MeOH in dichloromethane). Yield: 124 mg of **17b** as colorless oil (72%). TLC (4% MeOH in $CH_2Cl_2$): 0.48. HR-ESI-MS (m/z): $[M+H]^+$ calculated for $[C_{30}H_{48}N_2O_{11}PS_4]^+$: 771.1873; found: 771.1859. $^1$H-NMR ($CDCl_3$, 400 MHz): 1.191, 1.194 (2x 9 H, 2x s, 2x *t*-Bu (SATE)); 1.30, 1.53 (2x 3 H, 2x s, 2x $CH_3$ (Isopropyliden)); 2.31 (3 H, s, $CH_2SCOCH_3$); 3.08 (4 H, t, $^3J_{HH}$ = 6.68 Hz , 2x $SCH_2$ (SATE)); 3.16 (2 H, t, $^3J_{HH}$ = 7.23 Hz, $CH_2SCOCH_3$); 3.25-3.38 (2 H, m, $SCH_2$); 4.06 (4 H, dd, $^3J_{HP}$ = 14.68, $^3J_{HH}$ = 6.94 Hz, 2x $POCH_2$ (SATE)); 4.25-4.34 (2 H, m, $CH_2$ (5´)); 4.35-4.39 (1 H, m, CH (4´)); 4.86 (1 H, dd, $^3J_{HH}$ = 6.30 Hz, $^3J_{HH}$ = 3.98 Hz, CH (3´)); 4.96 (1 H, dd, $^3J_{HH}$ = 6.38 Hz, $^3J_{HH}$ = 1.38 Hz, CH (2´)); 5.68 (1 H, d, $^3J_{HH}$ = 1.43 Hz, CH (1´)); 6.20 (1 H, d, $^3J_{HH}$ = 7.07 Hz, CH (5)); 7.49 (1 H, d, $^3J_{HH}$ = 7.12 Hz, CH (6)) ppm. $^{13}$C-NMR ($CDCl_3$, 101 MHz): δ 25.29, 27.16 2x $CH_3$ (isopropyliden); 27.37 2x *t*-Bu (SATE); 28.51-28.59 (1x d, 1x s, 2x $SCH_2$ (SATE), $CH_2SCOCH_3$); 29.58 $SCH_2$; 30.67 $SCOCH_3$; 46.59 2x $C_q$-*t*-Bu (SATE); 66.42 (d, $^2J_{CP}$ = 5.81 Hz, 2x $POCH_2$ (SATE); 67.51 (d, $^2J_{CP}$ = 5.82 Hz, C (5´)); 80.92 C (3´); 85.15 C (2´); 86.86 (d, $^3J_{CP}$ = 7.27 Hz, C (4´)); 96.33 C (1´); 103.87 C (5); 114.41 $C_q$ (isopropyliden); 142.03 C (6); 153.44 C (2); 177.91 C (4); 195.39 $SCOCH_3$; 205.64 2x C=O (SATE) ppm. $^{31}$P-NMR ($CDCl_3$, 162 MHz): δ -1.92 ppm.

$^{1}$H-NMR (400 MHz, $CDCl_3$) of compound **17b**

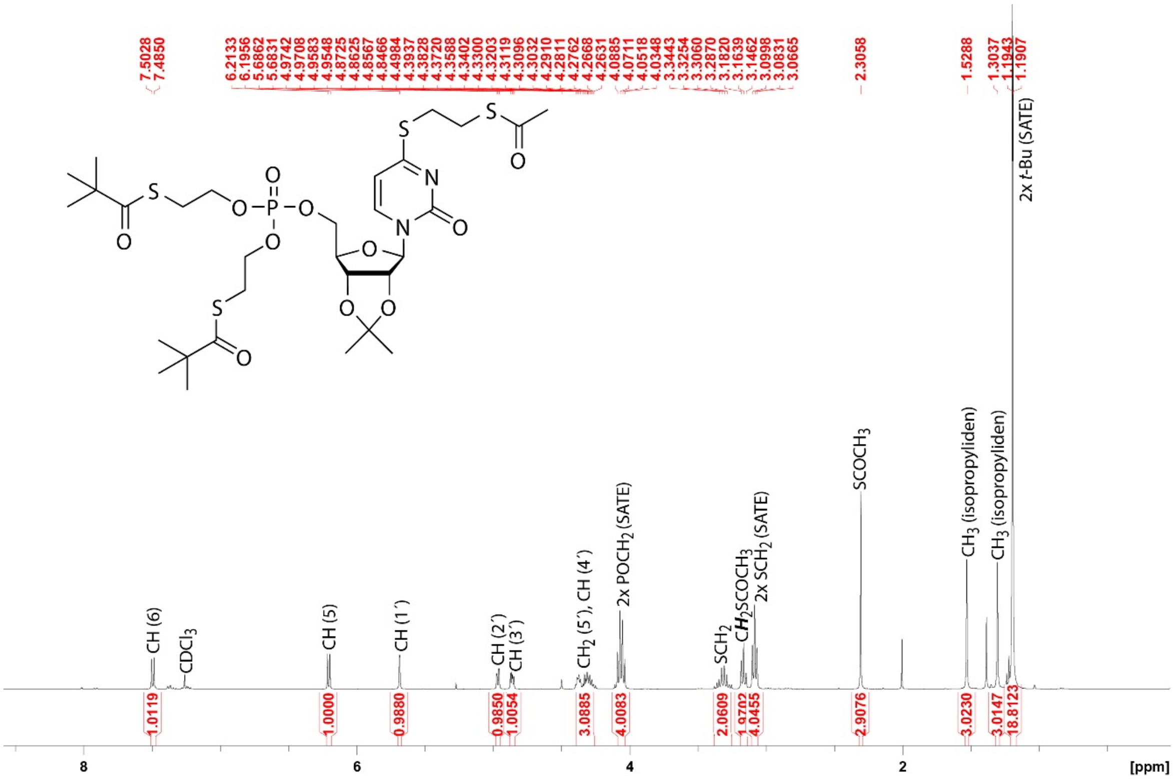

$^{13}$C-NMR (101 MHz, $CDCl_3$) of compound **17b**

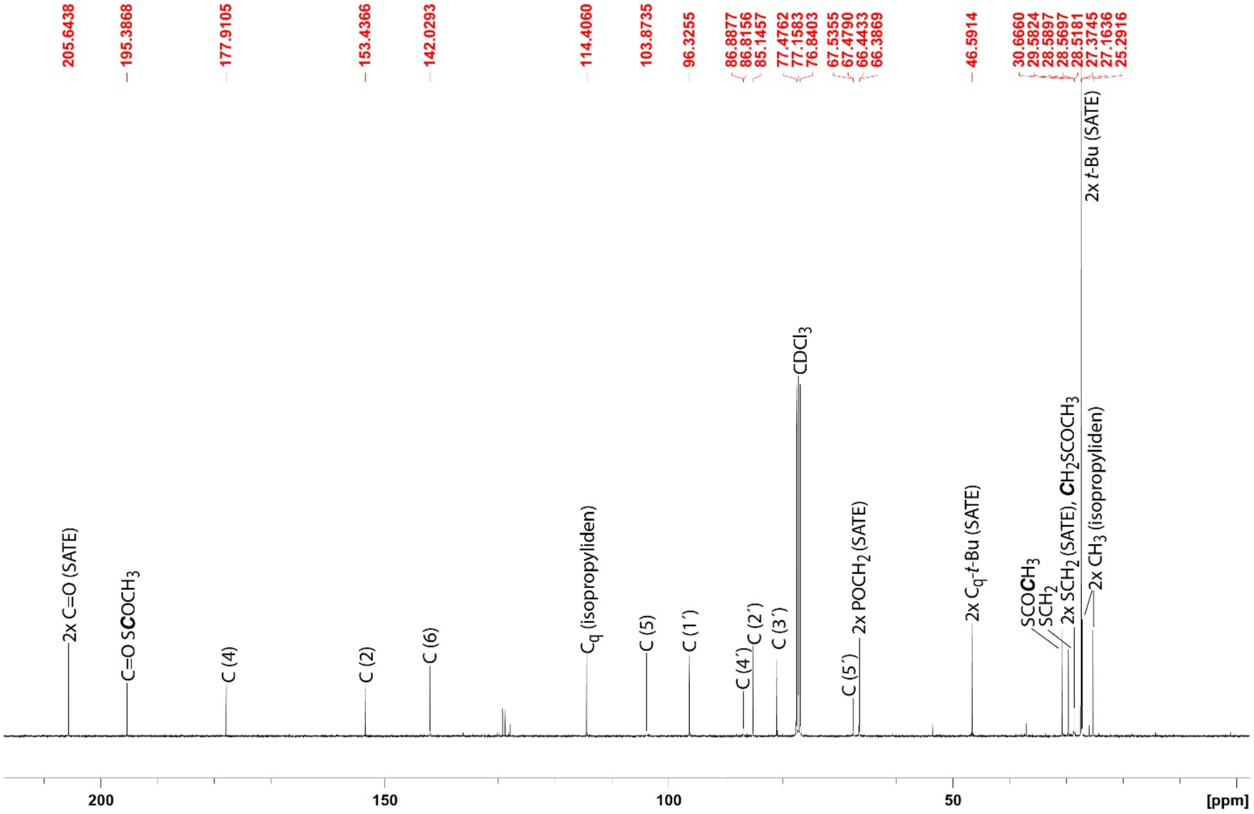

$^{31}$P-NMR (162 MHz, $CDCl_3$) of compound **17b**

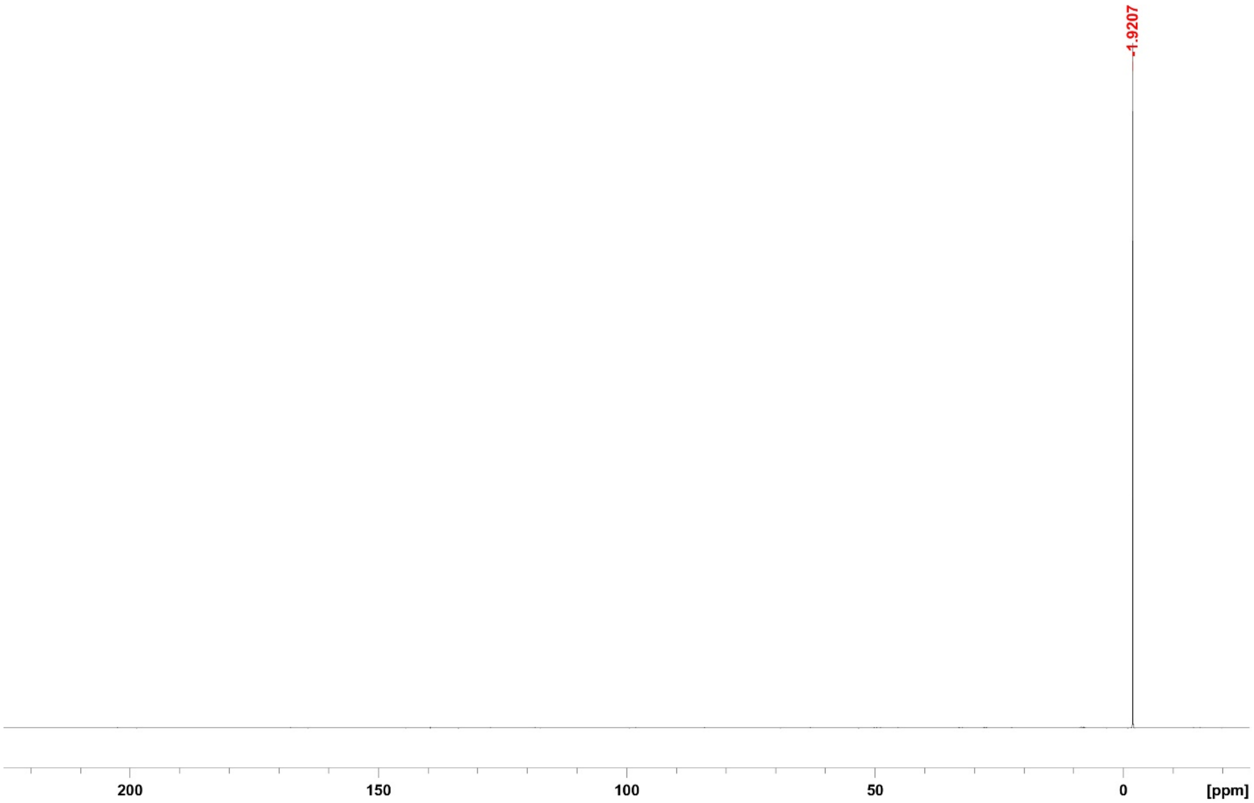

### 5.9. Synthesis of bis(*S*-pivaloyl-2-thioethyl)-5´-*O*-(2´,3´-*O*-isopropylidene-4-(*S*-(β-pivaloylmercapto)ethyl)-uridinyl) phosphate (17c) [5,6]

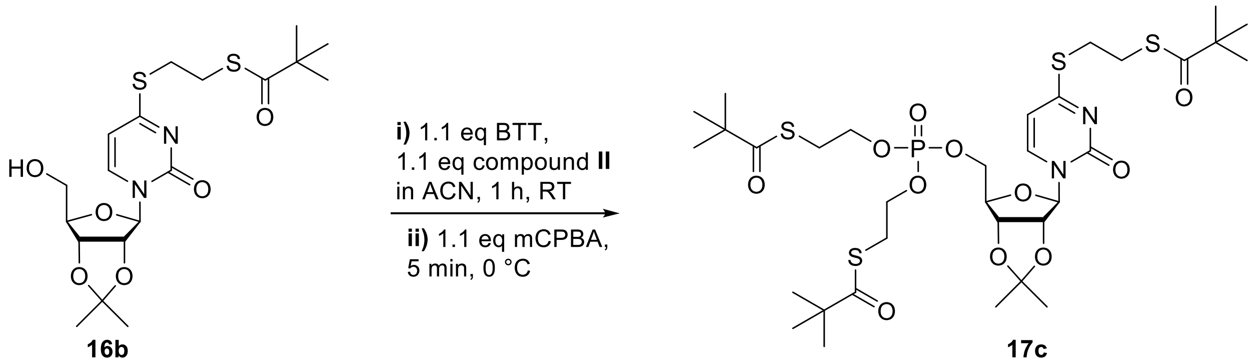


**16b** **17c**

Compound **16b** (115 mg, 0.26 mmol) and compound **II** (1.1 eq, 0.29 mmol, 130 mg) were coevaporated trice with acetonitrile and were subsequently dried under high vacuum for 5 min. The oily residue was dissolved in acetonitrile containing 0.3 M 5-(benzylthio)-1*H*-tetrazole (1.1 eq, 0.95 ml, dried over molecular sieves). The reaction mixture was stirred at ambient temperature for 1 h followed by cooling to 0 °C and the slow addition of meta-chloroperbenzoic acid (1.1 eq, 0.29 mmol, 49 mg). After 5 min at 0 °C the reaction mixture was concentrated and subjected to column chromatography (0-3% MeOH in $CH_2Cl_2$). Yield: 130 mg of **17c** as colorless oil (62%). TLC (5% MeOH in $CH_2Cl_2$): 0.52. HR-ESI-MS (m/z): $[M+H]^+$ calculated for $[C_{33}H_{54}N_2O_{11}PS_4]^+$: 813.2343; found: 813.2296. $^1$H-NMR ($CDCl_3$, 400 MHz): δ 1.200, 1.201 (3x 9 H, 3x s, $CH_2SCOC(CH_3)_3$, 2x *t*-Bu (SATE)); 1.31, 1.54 (6 H, 2x s, 2x $CH_3$ (isopropyliden)); 3.09 (4 H, t, $^3J_{HH}$ = 6.70 Hz, 2x $SCH_2$ (SATE)); 3.13 (2 H, t, $^3J_{HH}$ = 7.28 Hz, $CH_2SCOC(CH_3)_3$); 3.24-3.37 (2 H, m, $SCH_2$); 4.07 (4 H, dd, $^3J_{HP}$ = 14.76, $^3J_{HH}$ = 6.80 Hz, $POCH_2$ (SATE)); 4.25-4.35 (2 H, m, $CH_2$ (5´); 4.36-4.40 (1 H, m, CH (4´)); 4.87 (1 H, dd, $^3J_{HH}$ = 6.34 Hz, $^3J_{HH}$ = 3.97 Hz, CH (3´)); 4.97 (1 H, dd, $^3J_{HH}$ = 6.37 Hz, $^3J_{HH}$ = 1.49 Hz, CH (2´)); 5.68 (1 H, d, $^3J_{HH}$ = 1.40 Hz, CH (1´)); 6.20 (1 H, d, $^3J_{HH}$ = 7.07 Hz, CH (5)); 7.48 (1 H, d, $^3J_{HH}$ = 7.15 Hz, CH (6)) ppm. $^{13}$C-NMR ($CDCl_3$, 101 MHz): δ 25.30, 27.17 2x $CH_3$ (isopropyliden); 27.38, 27.41 2x *t*-Bu (SATE), $CH_2SCOC(CH_3)_3$; 27.93 $CH_2SCOC(CH_3)_3$, 28.56 (d, $^3J_{CP}$ = 7.27 Hz, 2x $SCH_2$ (SATE)); 29.67 $SCH_2$; 46.55, 46.59 2x $C_q$ *t*-Bu (SATE), $CH_2SCOC(CH_3)_3$; 66.40 (d, $^2J_{CP}$ = 5.81 Hz, 2x $POCH_2$ (SATE); 67.51 (d, $^2J_{CP}$ = 5.81 Hz, C (5´)); 80.97 C (3´); 85.14 C (2´); 86.90 (d, $^3J_{CP}$ = 7.27 Hz, C (4´)); 96.39 C (1´); 103.84 C (5); 114.38 $C_q$ (isopropyliden); 142.00 C (6); 153.40 C (2); 178.04 C (4); 205.64 2x C=O (SATE); 206.36 C=O $CH_2SCOC(CH_3)_3$ ppm. $^{31}$P-NMR ($CDCl_3$, 162 MHz): δ -1.90 ppm.

$^{1}$H-NMR (400 MHz, $CDCl_3$) of compound **17c**

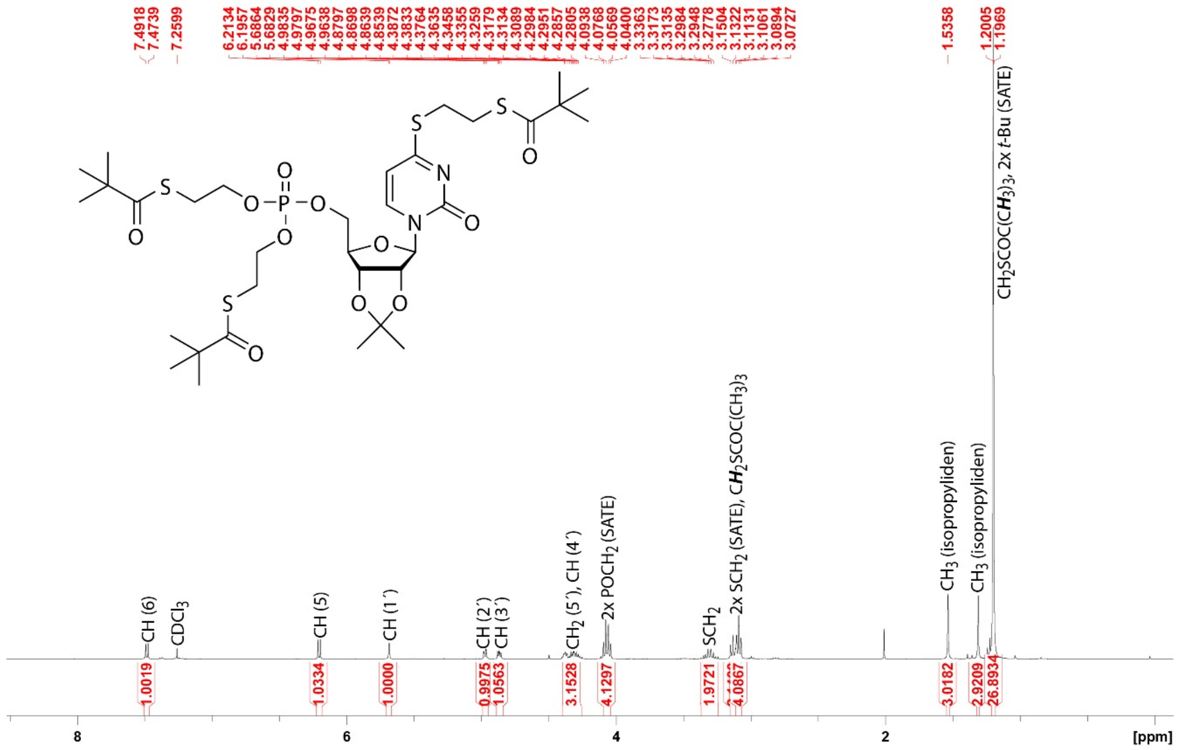


$^{13}$C-NMR (101 MHz, $CDCl_3$) of compound **17c**

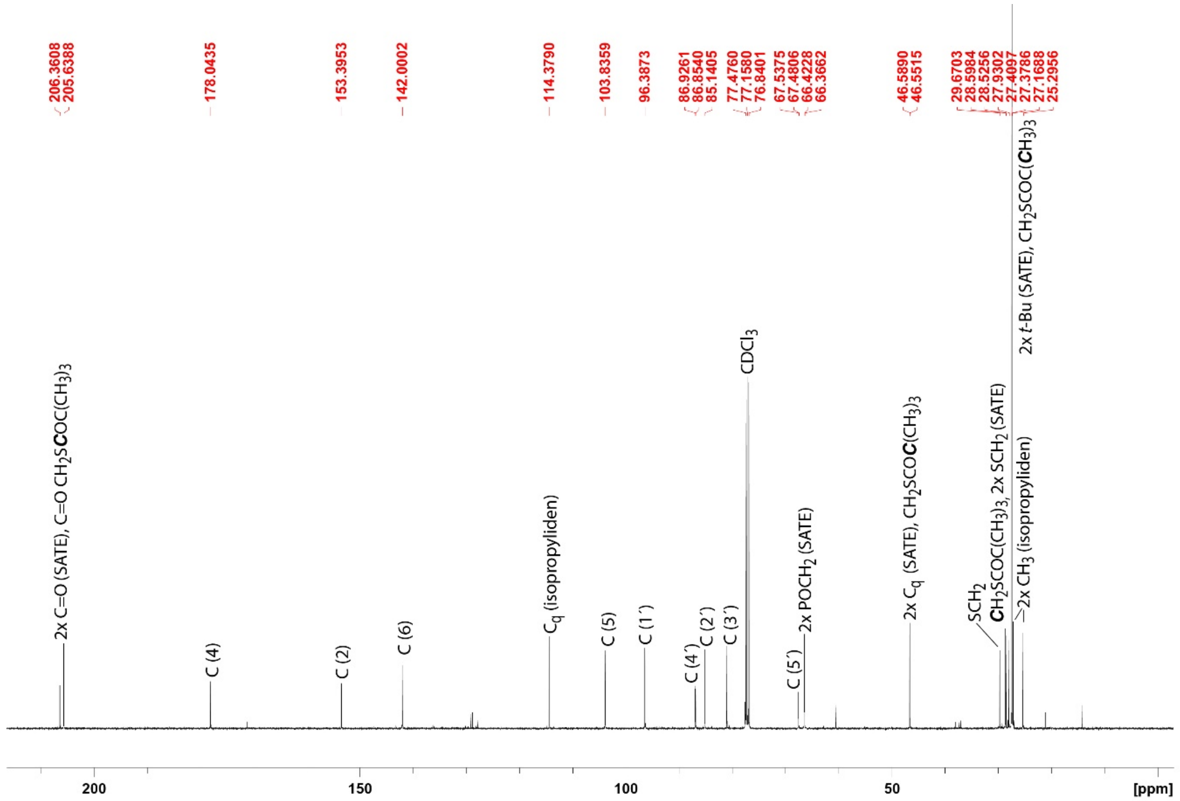

$^{31}$P-NMR (162 MHz, $CDCl_3$) of compound **17c**

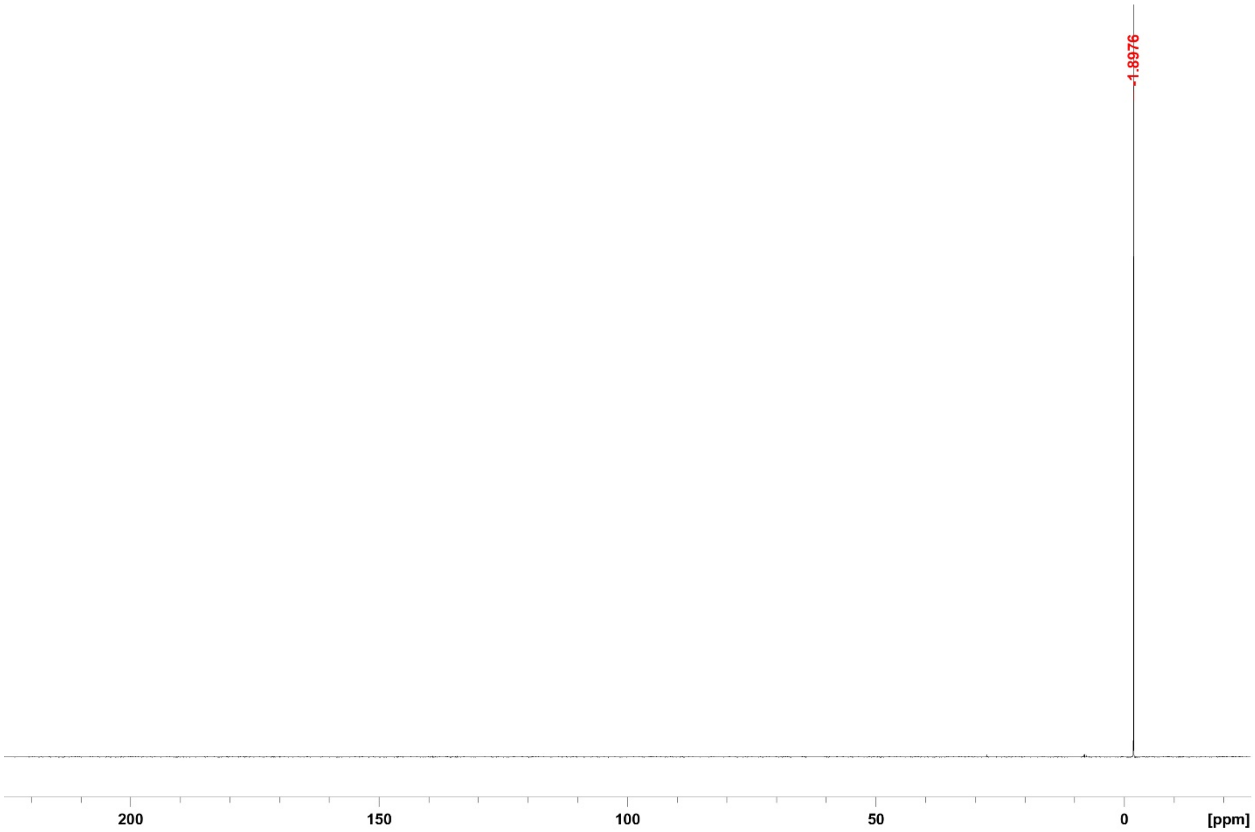

**5.10. Synthesis of bis(4-acetoxybenzyl)-5´-*O*-(4-(*S*-(β-acetylmercapto)ethyl)-uridinyl) phosphate (18a)** [17]

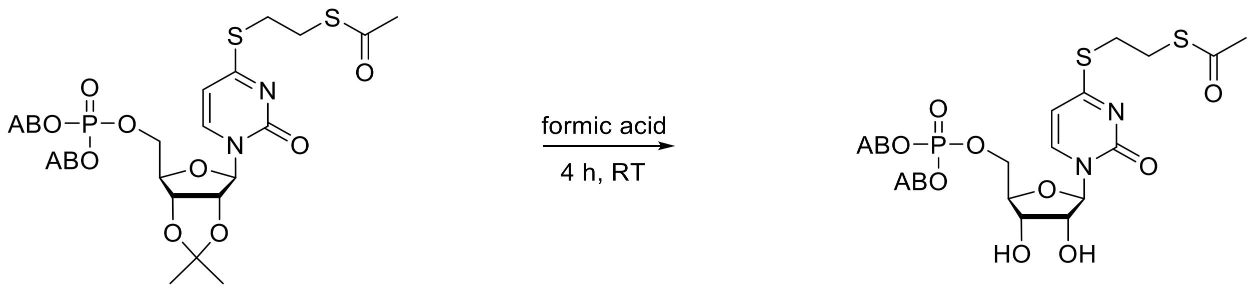


**17a** **18a**

Compound **17a** (89 mg, 0.11 mmol) was dissolved in formic acid (0.8 ml) was stirred at ambient temperature for 4 h. The solvent was removed under reduced pressure and the oily residue was subjected to column chromatography (0-4% MeOH in dichloromethane). Yield: 19 mg of **18a** as colorless oil (23%). TLC (4% MeOH in $CH_2Cl_2$): 0.28. HR-ESI-MS (m/z): $[M+H]^+$ calculated for $[C_{31}H_{36}N_2O_{13}PS_2]^+$: 739.1391; found: 739.1347. $^1$H-NMR ($CDCl_3$, 400 MHz): δ 2.301, 2.303 (6 H, 2x s, Acetyl (AB)); 2.34 $SCOCH_3$; 3.20 (2 H, t, $^3J_{HH}$ = 7.13 Hz, $CH_2SCOCH_3$); 3.29-3.39 (2 H, m, $SCH_2$); 3.94-3.98 (2 H, m, CH (3´), CH (2´)); 4.04 (1 H, ddd, $^2J_{HH}$ = 11.48 Hz, $^3J_{HH}$ or $^3J_{HP}$ = 5.50 Hz, $^3J_{HH}$ or $^3J_{HP}$ = 3.22 Hz, $CH_a$ (5´)); 4.21 (1 H, ddd, $^2J_{HH}$ = 11.51 Hz, $^3J_{HH}$ or $^3J_{HP}$ = 5.74 Hz, $^3J_{HH}$ or $^3J_{HP}$ = 2.62 Hz, $CH_b$ (5´)); 4.29 (1 H, m, CH (4´)); 4.92-5.07 (4 H, m, 2x $CH_2$ (AB)); 5.73 (1 H, d, $^3J_{HH}$ = 3.78 Hz, CH (1´)); 6.11 (1 H, d, $^3J_{HH}$ = 7.15 Hz, CH (5)); 7.07-7.10, 7.32-7.36 (8 H, m, aromat. CH (AB)); 7.78 (1 H, d, $^3J_{HH}$ = 7.15 Hz, CH (6)) ppm. $^{13}$C-NMR ($CDCl_3$, 101 MHz): δ 21.25 2x Acetyl (AB); 28.66 $CH_2SCOCH_3$; 29.61 $SCH_2$; 30.73 $SCOCH_3$; 66.64 (d, $^2J_{CP}$ = 5.81 Hz, C (5´)); 69.26-69.34 (2x d, 2x $CH_2$ (AB)); 71.08 C (3´); 76.49 C (2´); 84.32 (d, $^3J_{CP}$ = 7.99 Hz, C (4´)); 92.96 C (1´); 104.21 C (5); 122.18, 129.54, 129.60 aromat. C (AB), 133.04 (d, $^3J_{CP}$ = 5.09 $C_{q,a}$ (AB)); 133.09 (d, $^3J_{CP}$ = 5.09 $C_{q,b}$ (AB)); 139.60 C (6); 151.16, 151.18 $C_q$ (AB); 155.27 C (2); 169.52, 169.53 2x C=O (AB); 177.50 C (4); 195.45 C=O $SCOCH_3$ ppm. $^{31}$P-NMR ($CDCl_3$, 162 MHz): δ -0.63 ppm.

$^{1}$H-NMR (400 MHz, $CDCl_3$) of compound **18a**

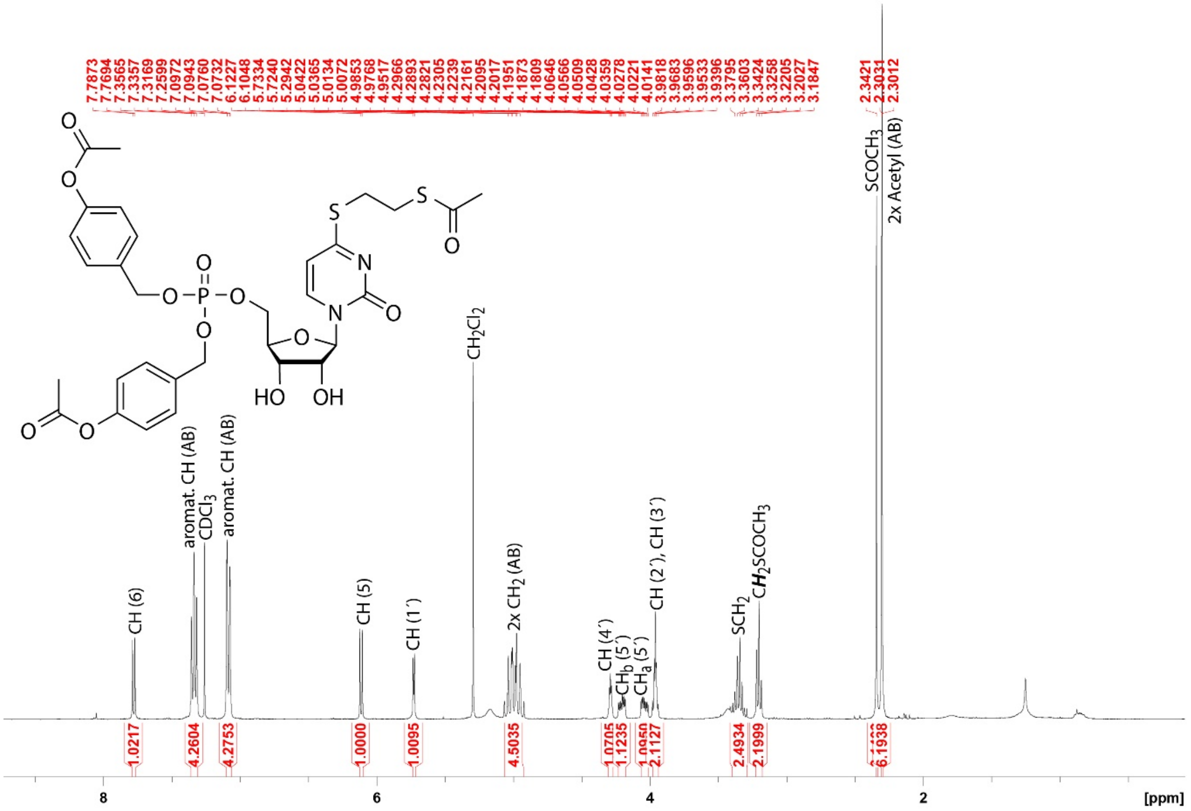

$^{13}$C-NMR (101 MHz, $CDCl_3$) of compound **18a**

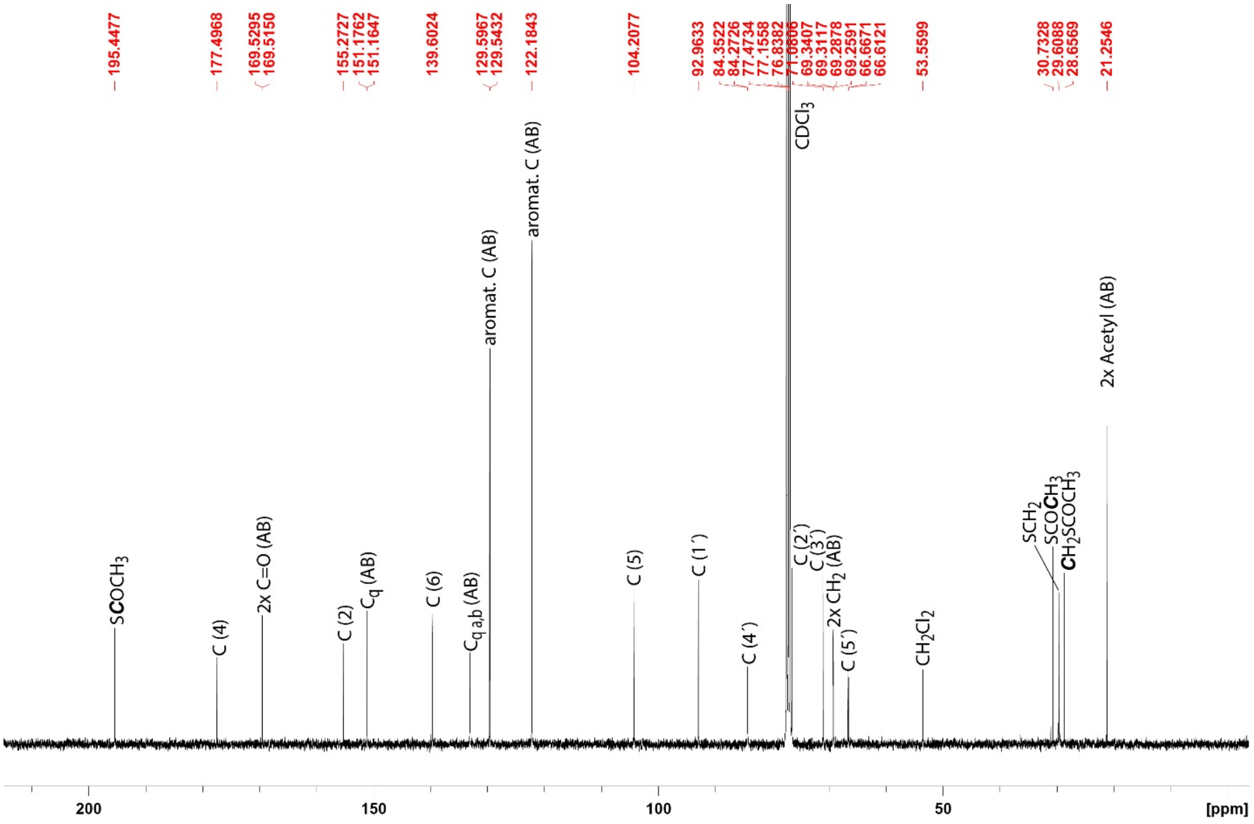

$^{31}$P-NMR (162 MHz, $CDCl_3$) of compound **18a**

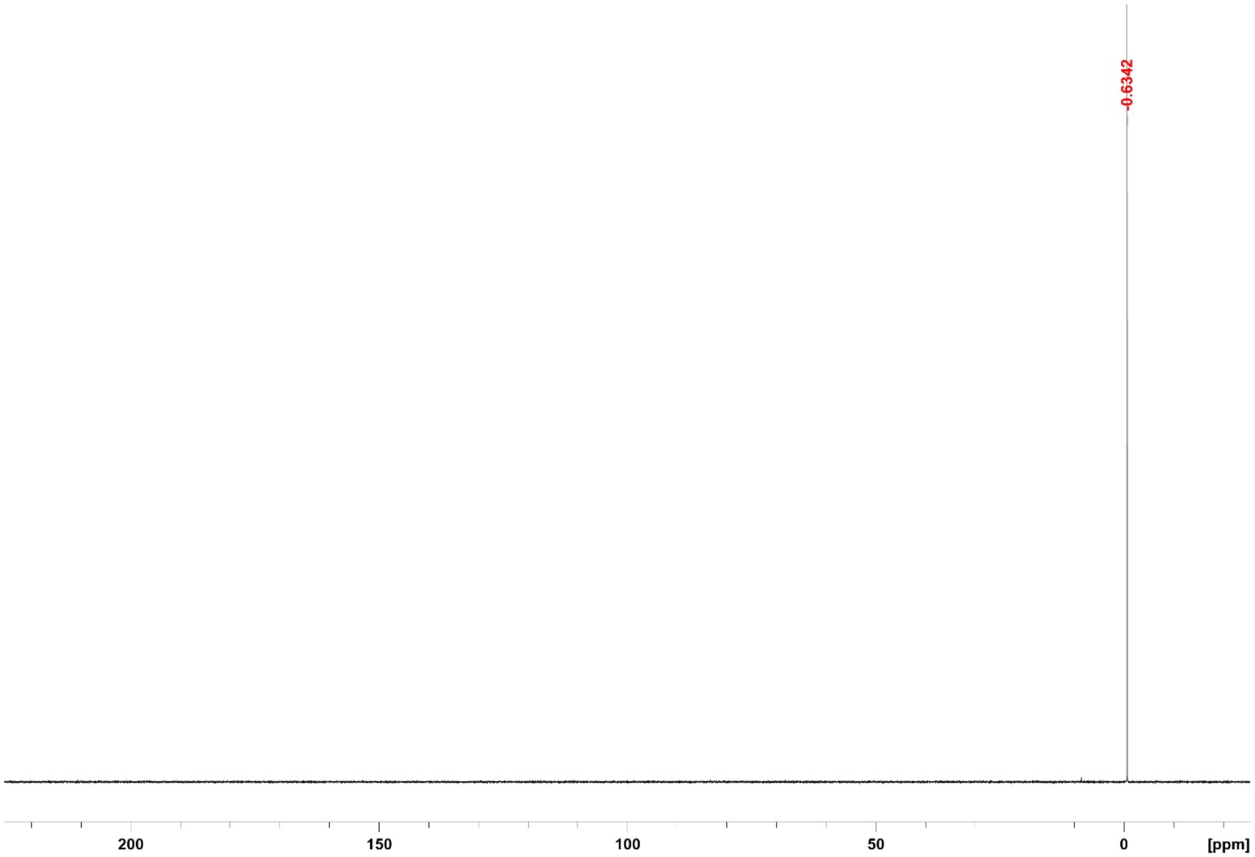

**5.11. Synthesis of bis(*S*-pivaloyl-2-thioethyl)-5´-*O*-(4-(*S*-(β-acetylmercapto)ethyl)-uridinyl) phosphate (18b)** [17]

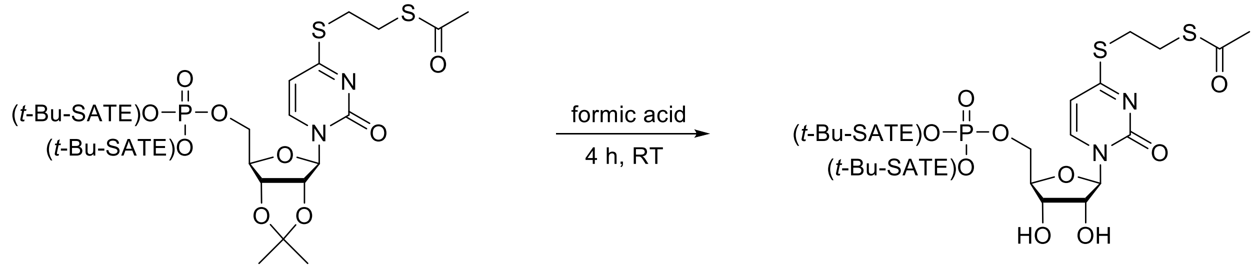


**17b** **18b**

Compound **17b** (124 mg, 0.17 mmol) was dissolved in formic acid (1.5 ml) was stirred at ambient temperature for 4 h. The solvent was removed under reduced pressure and the oily residue was subjected to column chromatography (2-5% MeOH in dichloromethane). Yield: 27 mg of **18b** colorless oil/white foam (23%). TLC (6% MeOH in $CH_2Cl_2$): 0.41. HR-ESI-MS (m/z): $[M+H]^+$ calculated for $[C_{27}H_{44}N_2O_{11}PS_4]^+$: 731.1560; found: 731.1528. $^1$H-NMR ($CDCl_3$, 400 MHz): 1.21, 1.22 (2x 9 H, 2x s, 2x *t*-Bu (SATE)); 2.33 (3 H, s, $SCOCH_3$); 3.08-3.13 (4 H, m, 2x $SCH_2$ (SATE)); 3.20 (2 H, t, $^3J_{HH}$ = 7.23 Hz, $CH_2SCOCH_3$); 3.29-3.41 (2 H, m, $SCH_2$); 4.05-4.13 (4 H, m, 2x $POCH_2$ (SATE)); 4.18-4.24 (2 H, m, CH (2´), CH (3´)); 4.26-4.30 (1 H, m, $CH_a$ (5´)); 4.36-4.39 (1 H, m, CH (4´)); 4.39-4.43 (1 H, m, $CH_b$ (5´)); 5.81 (1 H, d, $^3J_{HH}$ = 3.62 Hz, CH (1´)); 6.37 (1 H, d, $^3J_{HH}$ = 7.16 Hz, CH (5)); 7.93 (1 H, d, $^3J_{HH}$ = 7.16 Hz, CH (6)) ppm. $^{13}$C-NMR ($CDCl_3$, 101 MHz): δ 27.41 2x *t*-Bu (SATE); 28.56 $CH_2SCOCH_3$; 28.63 2x $SCH_2$ (SATE); 29.61 $SCH_2$; 30.72 $SCOCH_3$; 46.69 2x $C_q$ *t*-Bu (SATE); 66.58-66.72 (m, 2x $POCH_2$ (SATE), C (5´)); 70.51 C (3´); 76.19 C (2´); 83.97 (d, $^3J_{CP}$ = 7.99 Hz, C (4´)); 92.96 C (1´); 104.43 C (5); 139.71 C (6); 155.19 C (2); 177.64 C (4); 195.46 $SCOCH_3$; 205.73, 205.75 2x C=O (SATE) ppm. $^{31}$P-NMR ($CDCl_3$, 162 MHz): δ -1.65 ppm.

<sup></sup>

$^{1}$H-NMR (400 MHz, $CDCl_3$) of compound **18b**

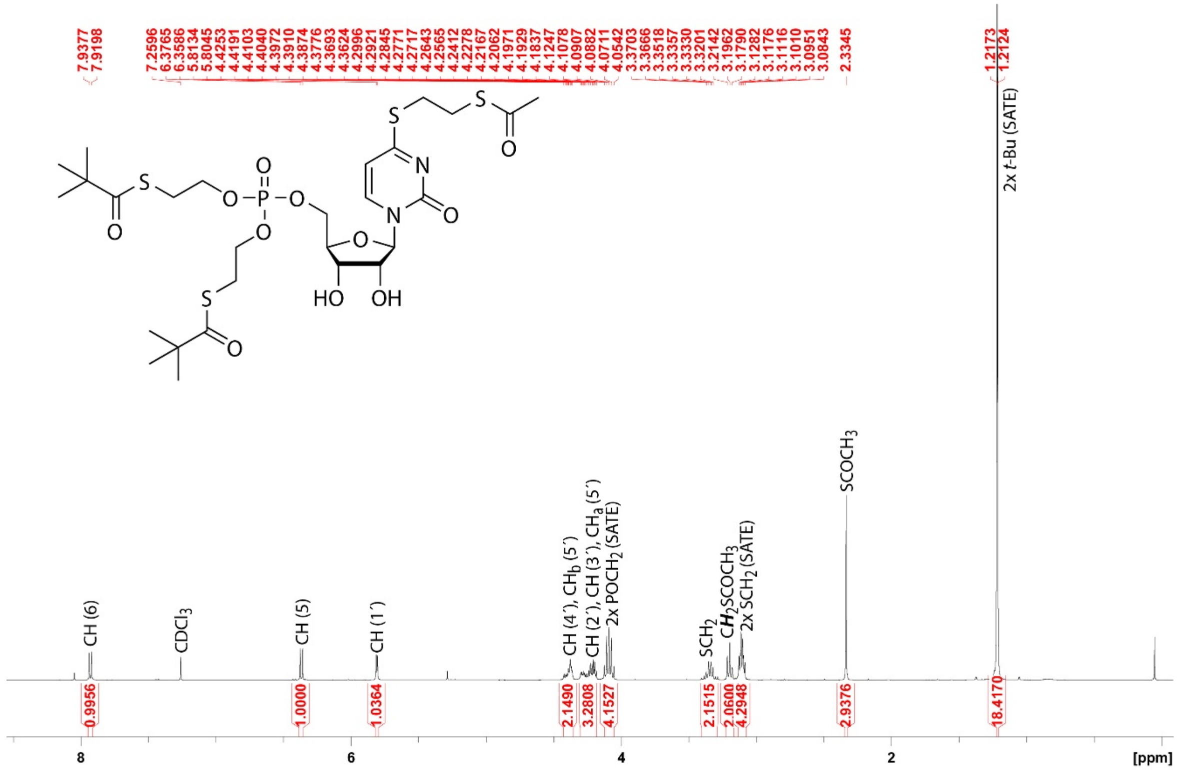


$^{13}$C-NMR (101 MHz, $CDCl_3$) of compound **18b**

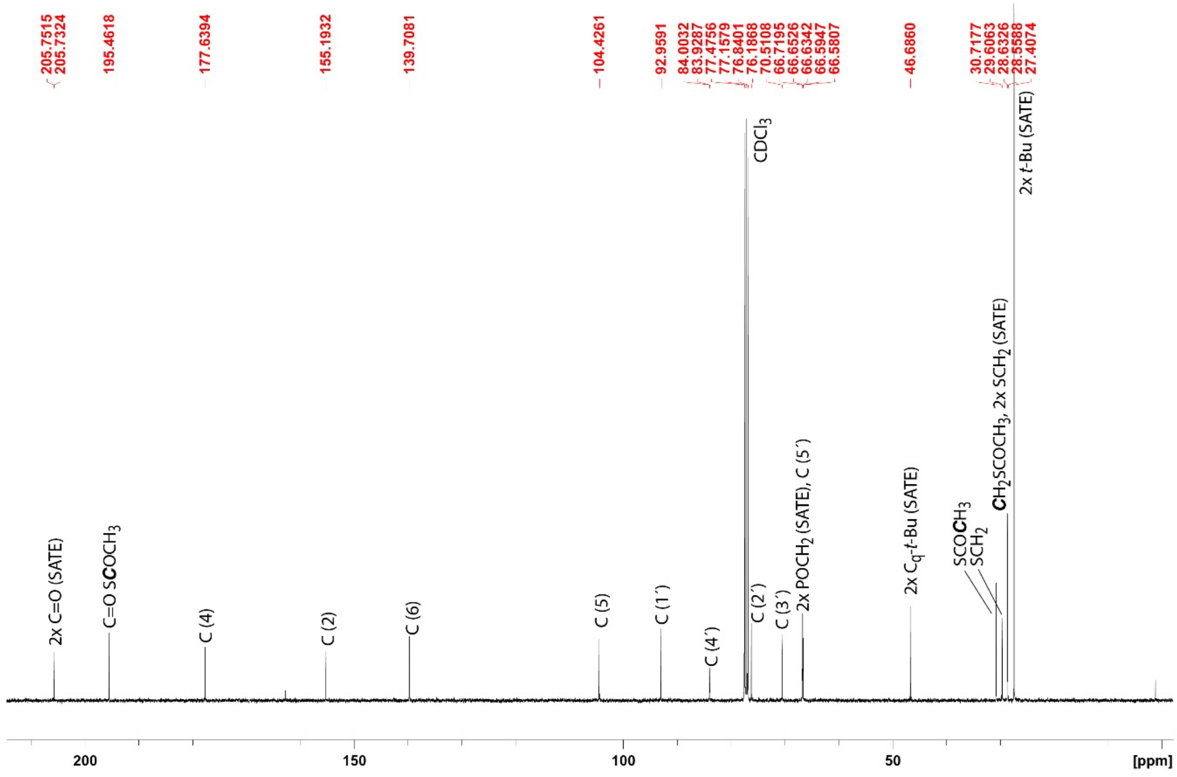

$^{31}$P-NMR (162 MHz, $CDCl_3$) of compound **18b**

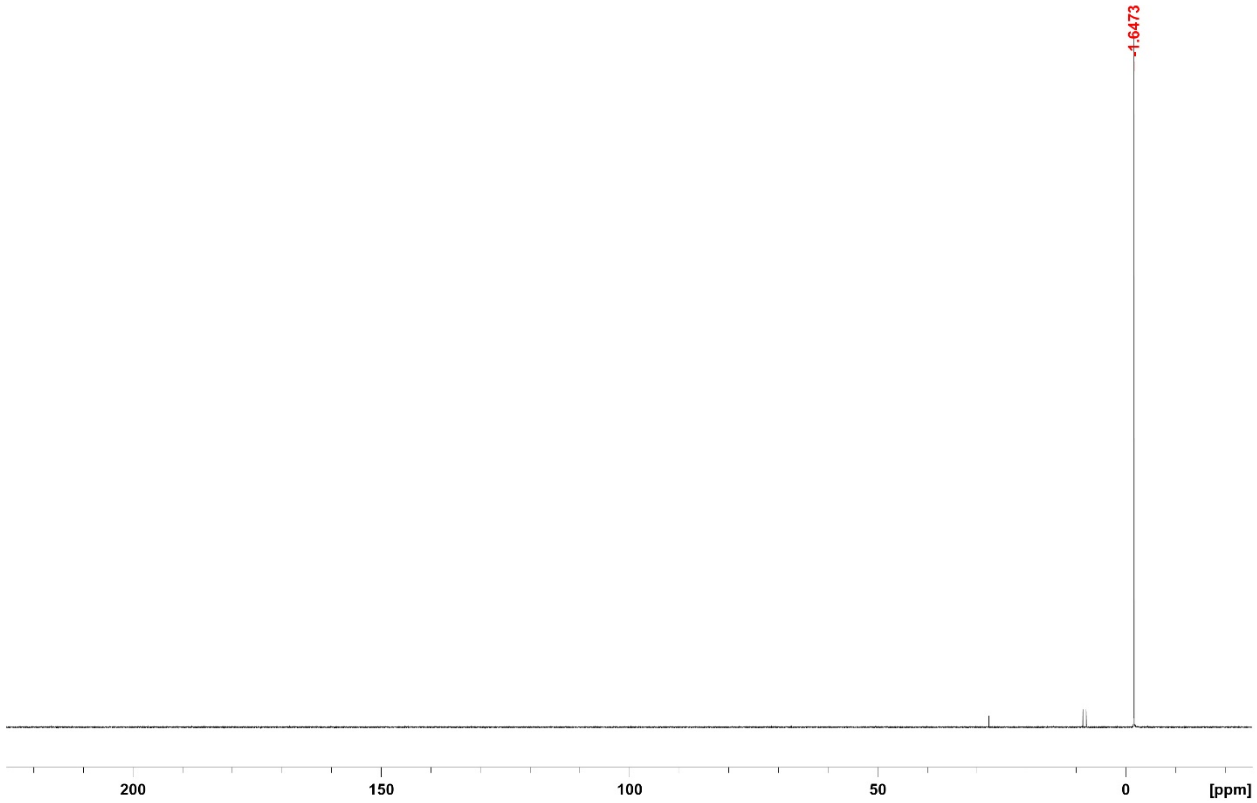

**5.12. Synthesis of bis(*S*-pivaloyl-2-thioethyl)-5´-*O*-(4-(*S*-(β-pivaloylmercapto)ethyl)-uridinyl) phosphate (18c)** [17]

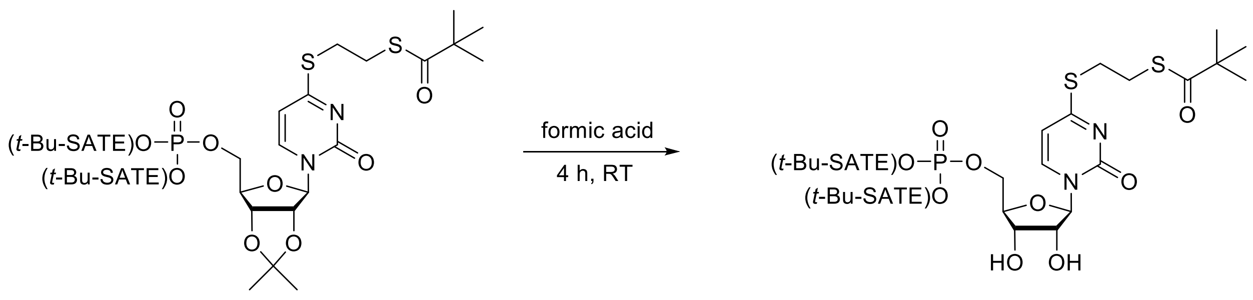


**17c** **18c**

Compound **17c** (336 mg, 0.41 mmol) was dissolved in formic acid (3.6 ml) was stirred at ambient temperature for 4 h. The solvent was removed under reduced pressure and the oily residue was subjected to column chromatography (2-5% MeOH in dichloromethane). Yield: 109 mg of **18c** colorless oil (34%). TLC (6% MeOH in $CH_2Cl_2$): 0.46. HR-ESI-MS (m/z): $[M+H]^+$ calculated for $[C_{30}H_{50}N_2O_{11}PS_4]^+$: 773.2030; found: 773.1984. $^1$H-NMR ($CDCl_3$, 400 MHz): δ 1.223, 1.227, 1.230 (3x 9 H, 3x s, $CH_2SCOC(C\mathit{H}_3)_3$, 2x *t*-Bu (SATE)); 3.11 (4 H, dd, $^2J_{HH}$ = 13.18 Hz, $^3J_{HH}$ = 6.57 Hz, 2x $SCH_2$ (SATE)); 3.17 (2 H, t, $^3J_{HH}$ = 7.28 Hz, $C\mathit{H}_2SCOCH_3$); 3.28-3.41 (2 H, m, $SCH_2$); 4.06-4.13 (4 H, m, $POCH_2$ (SATE)); 4.19 (1 H, dd, $^3J_{HH}$ = 5.24 Hz, $^3J_{HH}$ = 4.20 Hz, CH (2´)); 4.25 (1 H, dd, $^3J_{HH}$ = 5.50 Hz, $^3J_{HH}$ = 4.25 Hz, CH (3´); 4.26--4.30 (1 H, m, $CH_a$ (5´)); 4.37-4.41 (2 H, m, $CH_b$ (5´), CH (4´)); 5.80 (1 H, d, $^3J_{HH}$ = 4.12 Hz, CH (1´)); 6.39 (1 H, d, $^3J_{HH}$ = 7.16 Hz, CH (5)); 7.93 (1 H, d, $^3J_{HH}$ = 7.16 Hz, CH (6)) ppm. $^{13}$C-NMR ($CDCl_3$, 101 MHz): δ 27.43, 27.47 2x *t*-Bu (SATE), $CH_2SCOC(\mathit{C}H_3)_3$; 28.00 $\mathit{C}H_2SCOC(CH_3)_3$; 28.61 (d, $^3J_{CP}$ = 7.26 Hz, 2x $SCH_2$ (SATE)); 29.77 $SCH_2$; 46.64, 46.72 $CH_2SCO\mathit{C}(CH_3)_3$, 2x $C_q$ *t*-Bu (SATE); 66.63-66.70 (2x d, 2x $POCH_2$ (SATE)); 66.80 (d, $^2J_{CP}$ = 5.81 Hz, C (5´)); 70.89 C (3´); 76.46 C (2´); 84.30 (d, $^3J_{CP}$ = 8.00 Hz, C (4´)); 93.10 C (1´); 104.55 C (5); 139.54 C (6); 155.41 C (2); 177.94 C (4); 205.77, 205.80 2x C=O (SATE); 206.45 C=O $CH_2S\mathit{C}OC(CH_3)_3$ ppm. $^{31}$P-NMR ($CDCl_3$, 162 MHz): δ -1.66 ppm.

$^{1}$H-NMR (400 MHz, $CDCl_3$) of compound **18c**

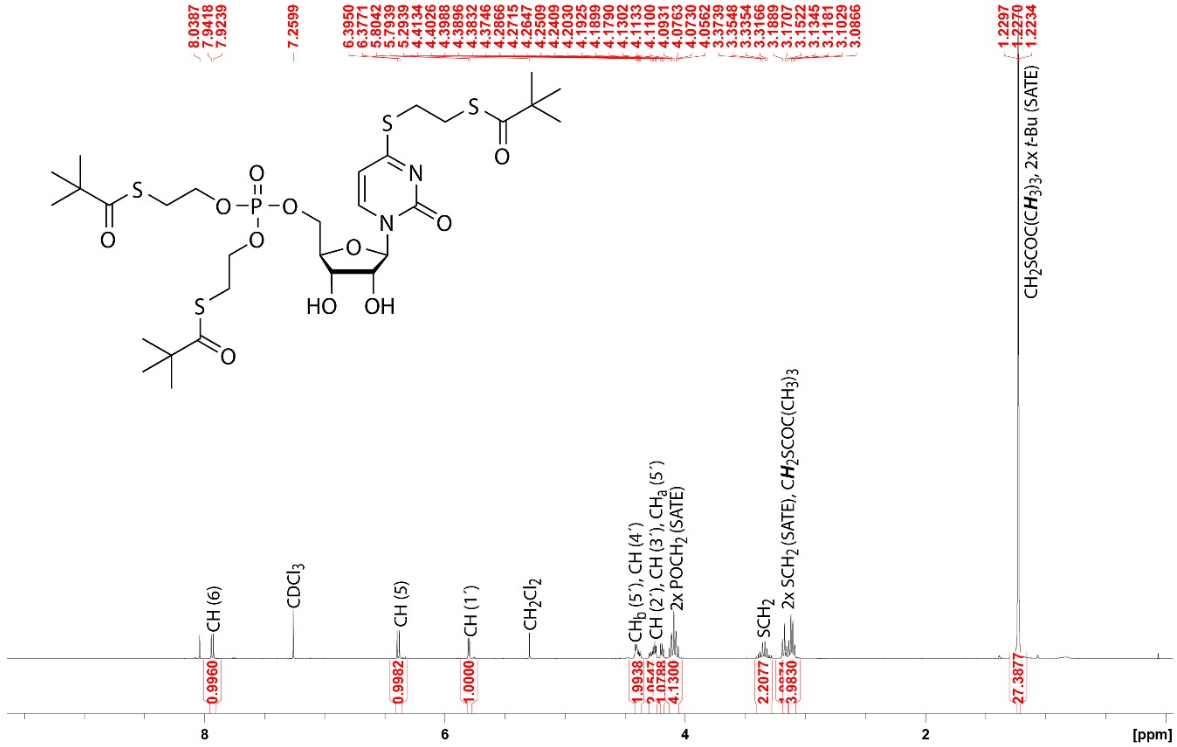


$^{13}$C-NMR (101 MHz, $CDCl_3$) of compound **18c**

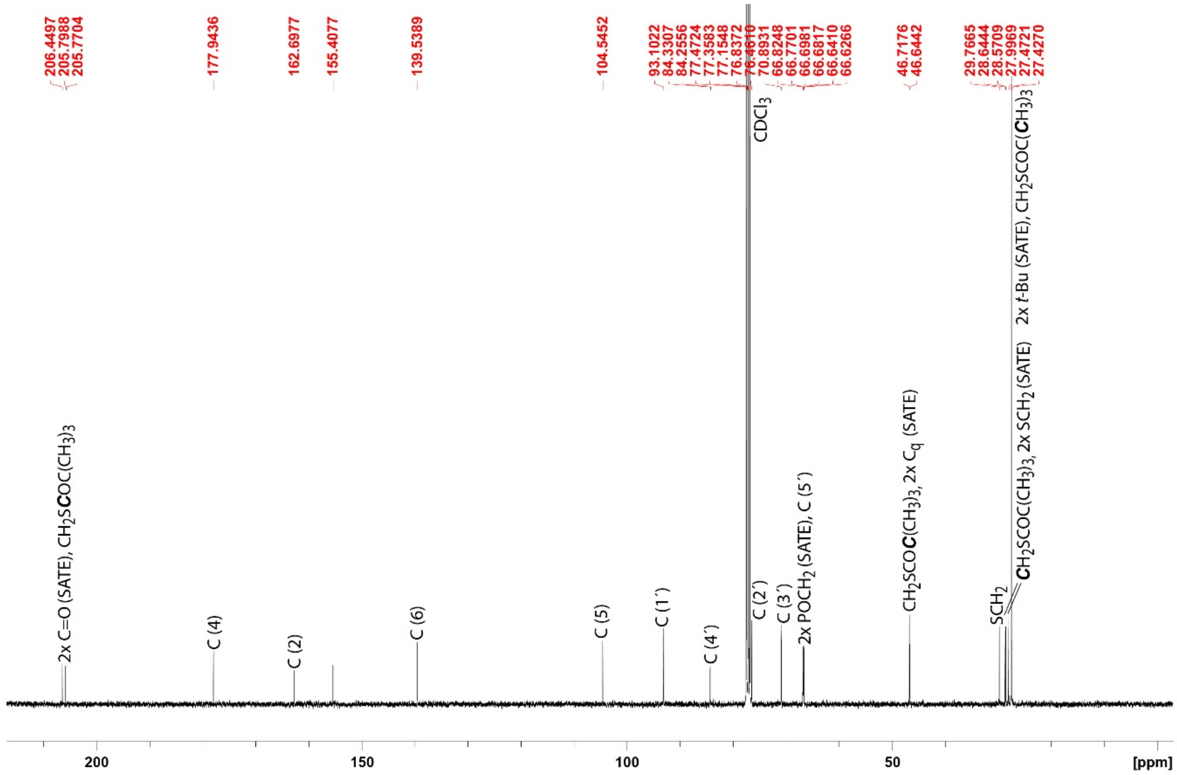

$^{31}$P-NMR (162 MHz, $CDCl_3$) of compound **18c**

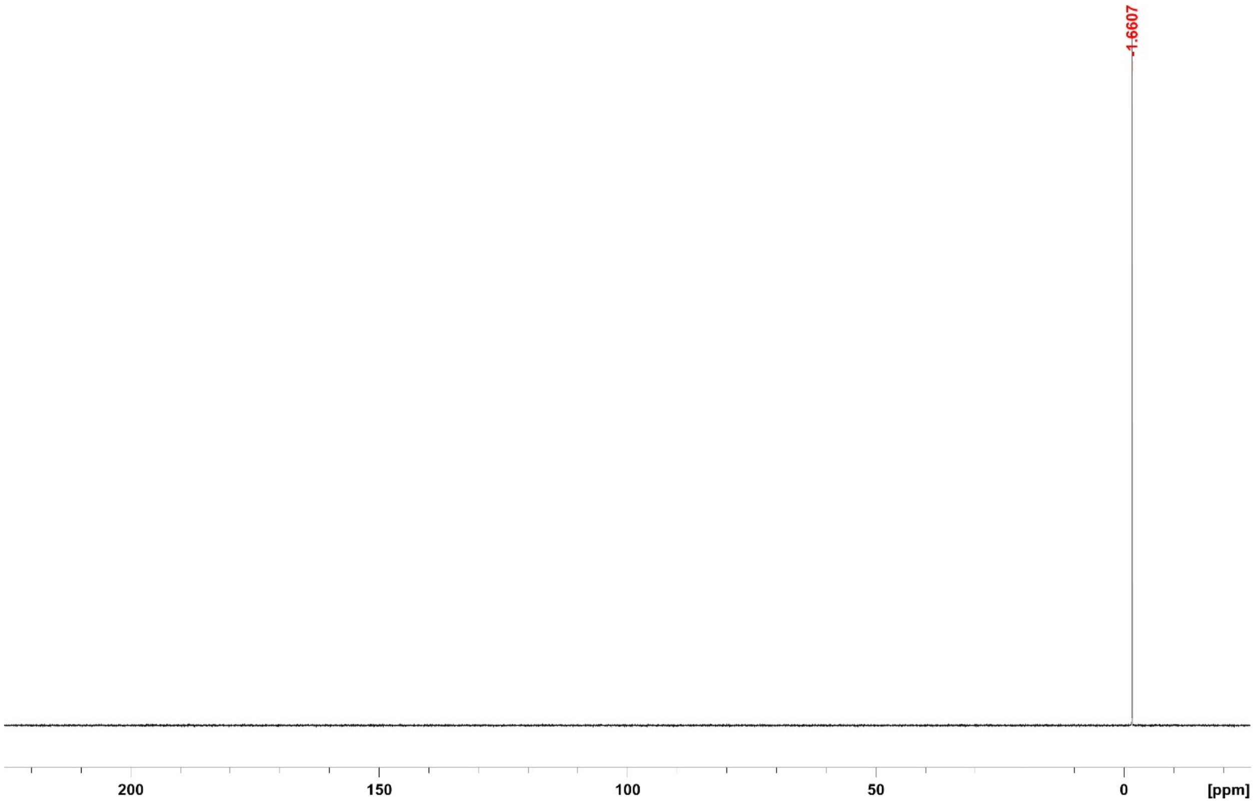

## 6. Reagent synthesis

### 6.1. Synthesis of bis-(4-acetoxybenzyl) *N,N*-diisopropylphosphoramidite (I)

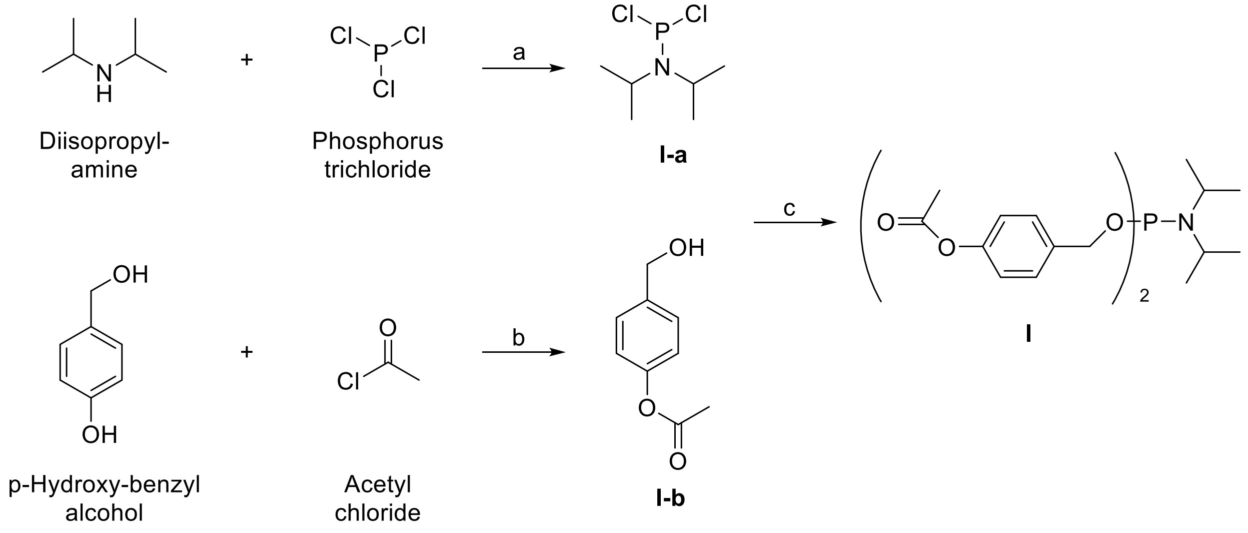


**Overview of the synthesis of bis-(4-acetoxybenzyl) *N,N*-diisopropylphosphoramidite**. Reaction conditions: **a)** $PCl_3$, 1.9 eq (*i*Pr)$_2$NH, hexane, 1 h, 0 °C to room temperature (RT), 91%. **b)** *p*-hydroxy benzylalcohol, 1.0 eq $Et_3N$, 1.1 eq $CH_3COCl$, THF, 140 min, 0 °C to RT, 63%. **c)** 2.3 eq $Et_3N$, 2.2 eq compound **I-b**, THF, 16 h, -78 °C to RT, 57%. Overall yield: 33%.

#### 6.1.1. Synthesis of *N,N*-diisopropylphosphoramidodichloride (I-a) [18]

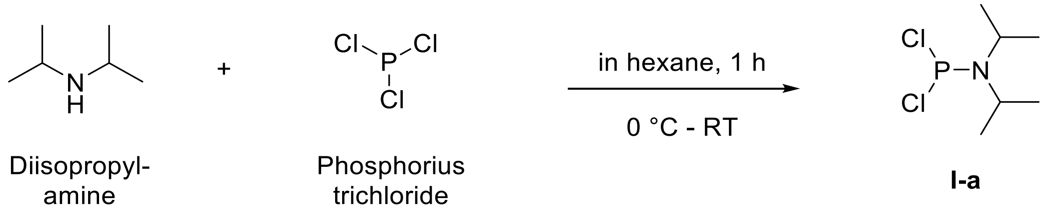


Phosphorous trichloride (0.33 ml, 3.802 mmol) and hexane (8.9 ml) were placed in an oven-dried flask under argon atmosphere and were cooled to 0 °C. Diisopropylamine (1.9 eq, 7.23 mmol, 1.01 ml) was added dropwise and the resulting suspension was stirred for 5 min at 0 °C and another 60 min at ambient temperature. Salts were removed by filtration and subsequently the frit was washed with dry hexane. Solvents were removed on a pre-dried rotavapor, which was flushed with argon afterwards. Compound **I-a** was immediately used for the next step without further purification. An analytical sample was subjected to NMR spectroscopy to evaluate identity and high purity. Yield: 696 mg as a colorless oil (91%). $^1$H-NMR ($CDCl_3$, 400 MHz): δ 1.28 (12 H, d, $^3J_{HH}$ = 6.88 Hz, 2x $(CH_3)_2$ (*i*Pr)); 3.93 (2 H, sept, $^3J_{HH}$ = 6.63 Hz, 2x CH (*i*Pr)) ppm. $^{31}$P-NMR ($CDCl_3$, 162 MHz): δ 169.60 ppm.

$^{1}$H-NMR (400 MHz, $CDCl_3$) of compound **I-a**

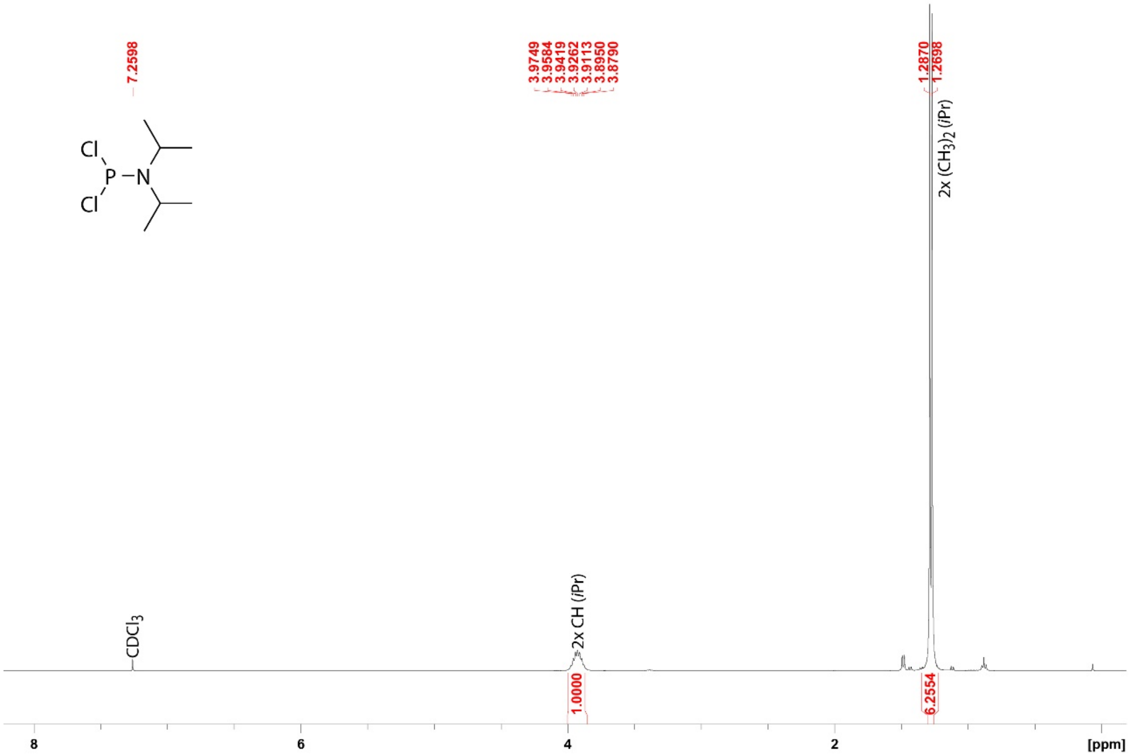


$^{31}$P-NMR (162 MHz, $CDCl_3$) of compound **I-a**

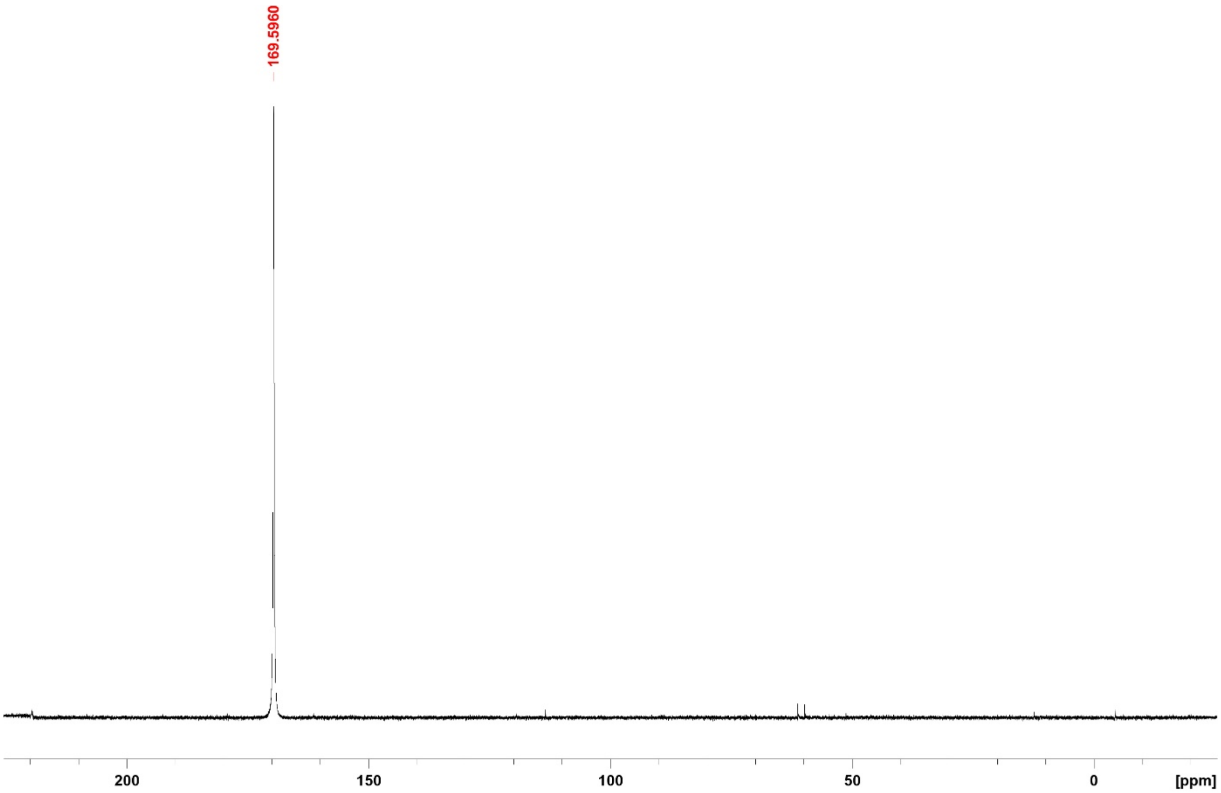

### 6.1.2. Synthesis of 4-acetoxy-benzylalcohol (I-b) [19]

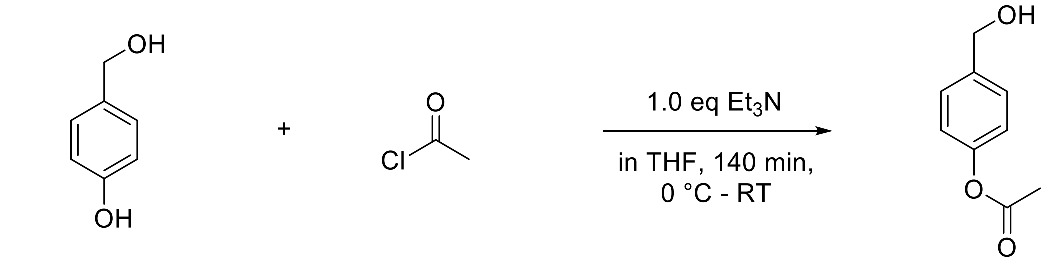


p-Hydroxy-benzyl alcohol | Acetyl chloride | **I-b**

*p*-Hydroxybenzyl alcohol (6.00 g, 48.33 mmol) and triethylamine (1.0 eq, 48.33 mol, 6.74 ml) were dissolved in tetrahydrofuran (60.0 ml) and cooled to 0 °C. Acetyl chloride (1.1 eq, 53.17 mmol, 3.79 ml) was added dropwise over a period of 20 min. The reaction mixture was stirred two more hours at 0 °C followed by filtration and evaporation of the solvent. The oily residue was diluted with dichloromethane and was washed with saturated sodium bicarbonate solution and water. Drying over sodium sulfate and column chromatography (20-50% ethyl acetate in c-hexane) delivers compound **I-b** as a colorless oil, which crystallized upon freezing. Yield: 5.07 g of **I-b** as a colorless oil/white solid (63%). TLC (ethyl acetate/c-hexane 1/1): 0.45. $^1$H-NMR ($d_6$-DMSO, 400 MHz): δ 2.25 (3 H, s, $CH_3$ Acetyl); 4.49 (2 H, d, $^3J_{HH}$ = 5.72 Hz, $CH_2$); 5.20 (1 H, t, $^3J_{HH}$ = 5.76 Hz, OH); 7.06 (2 H, d, $^3J_{HH}$ = 8.49 Hz, aromat. CH); 7.34 (2 H, d, $^3J_{HH}$ = 8.55 Hz, aromat. CH) ppm.

$^1$H-NMR (400 MHz, $d_6$-DMSO) of compound **I-b**

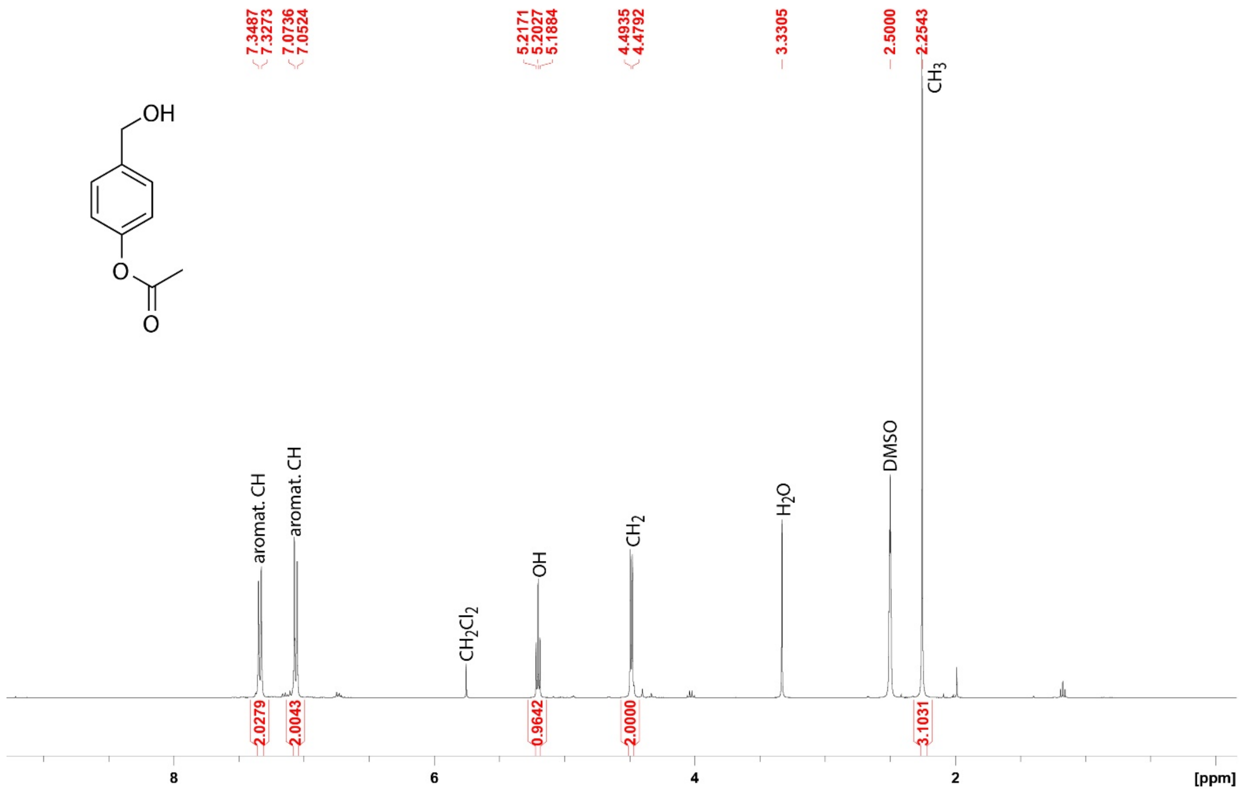

### 6.1.3. Synthesis of bis-(4-acetoxybenzyl) *N,N*-diisopropylphosphoramidite (I) [19]

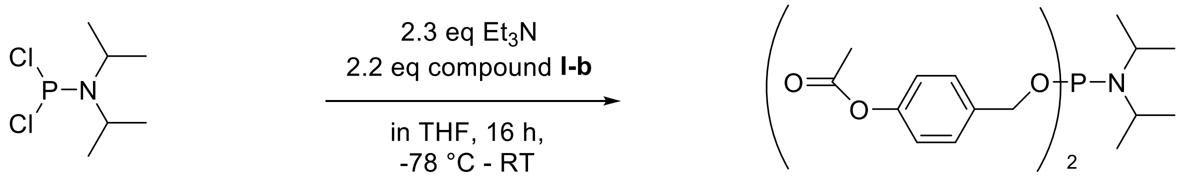


**I-a** **I**

Freshly prepared compound **I-a** (1.439 g, 7.12 mmol) was dissolved in tetrahydrofuran (7.0 ml) and cooled to -78 °C. Subsequently, a mixture of compound **I-b** (2.2 eq, 15.67 mmol, 2.603 g) and triethylamine (2.3 eq, 16.38 mmol, 2.28 ml) in tetrahydrofuran (7.0 ml) was added dropwise over a period of 60 min. The white slurry was allowed to warm to room temperature and was stirred for 16 h. Salts were removed by filtration and the product was concentrated under reduced pressure. Column chromatography (0-20% ethyl acetate in c-hexane) delivered the final product as a colorless oil that crystallized upon freezing. Yield: 1.860 g of **I** as white solid (57%). TLC (ethyl acetate/c-hexane 20/80): 0.43. $^1$H-NMR ($CDCl_3$, 400 MHz): δ 1.30 (12 H, d, $^3J_{HH}$ = 6.81 Hz, 2x $(CH_3)_2$ (*i*Pr)); 2.29 (6 H, s, 2x $CH_3$ (AB)); 3.69 (2 H, m, 2x CH (*i*Pr)); 4.67 (1 H, dd, $^2J_{HH}$ = 12.68 Hz, $^3J_{HP}$ = 8.54 Hz, $CH_a$ (AB); 4.75 (1 H, dd, $^2J_{HH}$ = 12.66 Hz, $^3J_{HP}$ = 8.10 Hz, $CH_b$ (AB); 7.04 (4 H, d, $^3J_{HH}$ = 8.59 Hz, aromat. CH); 7.35 (4 H, d, $^3J_{HH}$ = 8.66 Hz, aromat. CH) ppm. $^{31}$P-NMR ($CDCl_3$, 162 MHz): δ 147.96 ppm.

$^1$H-NMR (400 MHz, $CDCl_3$) of compound **I**

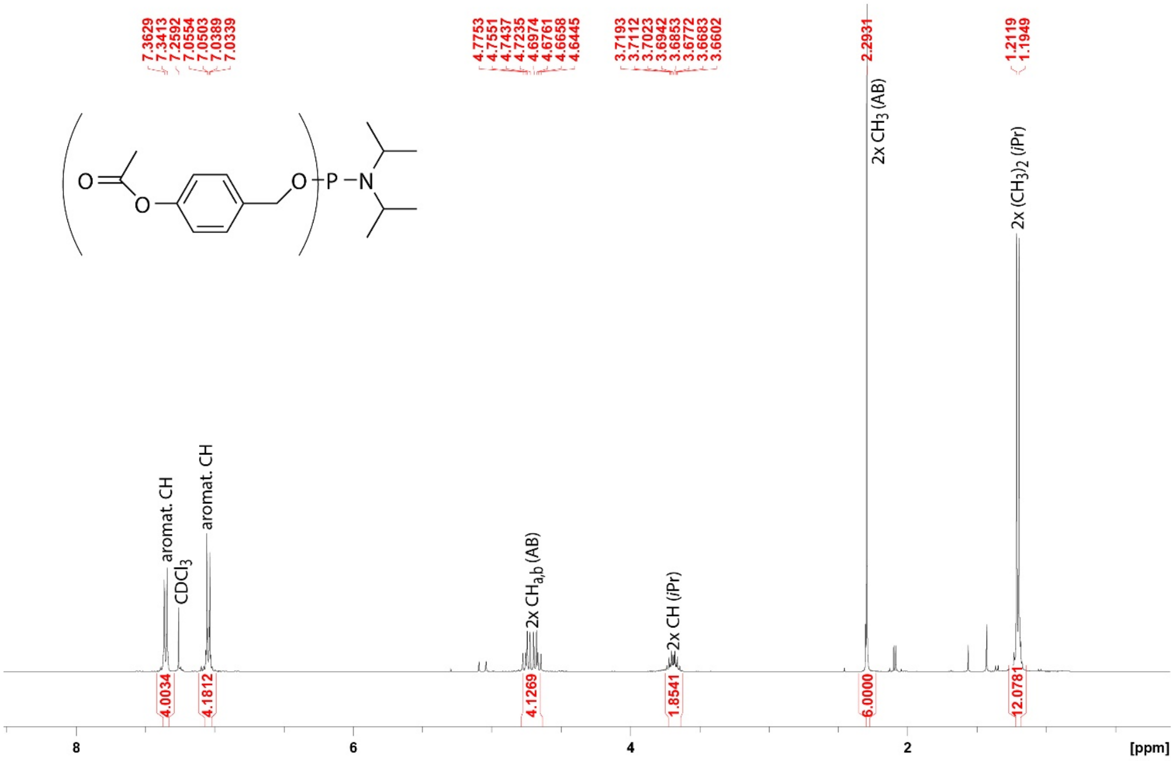

$^{31}$P-NMR (162 MHz, $CDCl_3$) of compound **I**

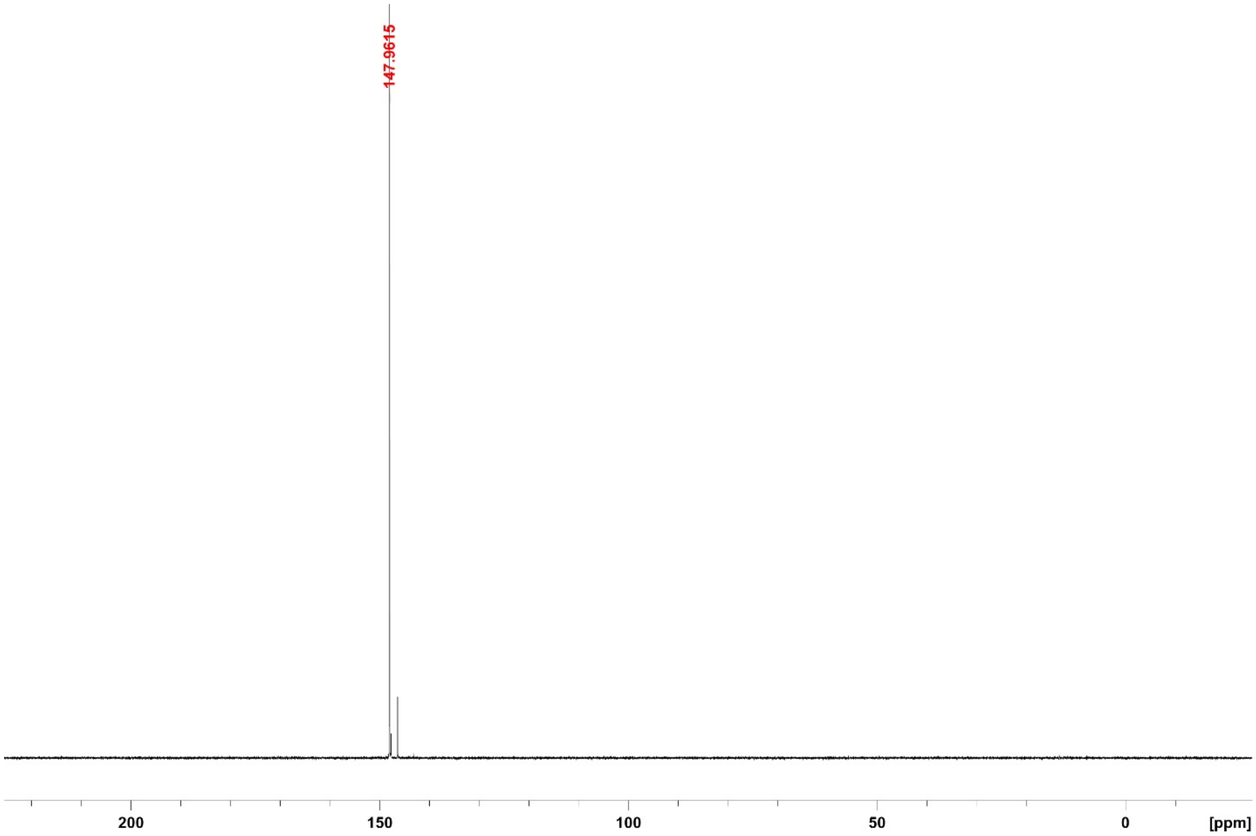

### 6.2. Synthesis of bis-(*S*-pivaloyl-2-thioethyl) *N,N*-diisopropylphosphoramidite (II)

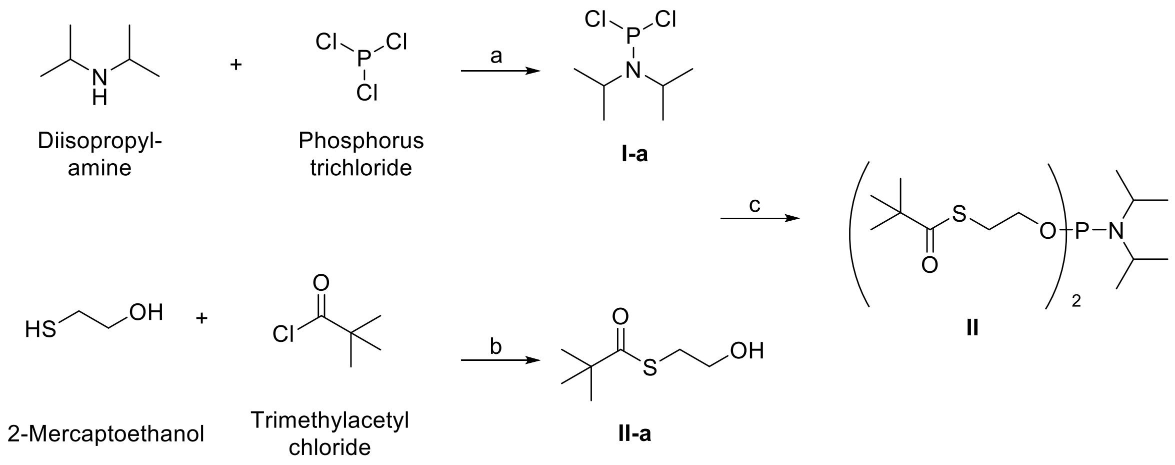


**Overview of the synthesis of bis-(*S*-pivaloyl-2-thioethyl) *N,N*-diisopropylphosphoramidite.** Reaction conditions: **a)** $PCl_3$, 1.9 eq ($i$Pr)$_2$NH, hexane, 1 h, 0 °C to RT, 91%. **b)** 2-mercaptoethanol, 1.3 eq $Et_3N$, 1.0 eq PivCl, $CH_2Cl_2$, 120 min, -78 °C to RT, 99%. **c)** 4.3 eq $Et_3N$, 2.0 eq compound **II-a**, THF, 6.5 h, -78 °C to RT, 60%. Overall yield: 54%

### 6.2.1. Synthesis of *S*-(2-hydroxyethyl) thiopivaloate (II-a) [20]

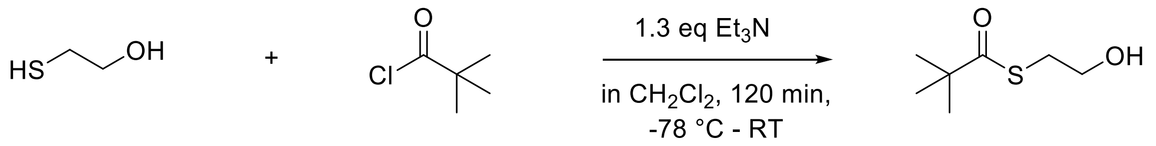


**2-Mercaptoethanol [$C_2H_6OS$]**  **Trimethylacetyl chloride**  **II-a**

2-Mercaptoethanol (1.50 ml, 21.39 mmol) and triethylamine (1.4 eq, 28.70 mmol, 4.0 ml) were dissolved in dichloromethane (55.0 ml) and were cooled to -78 °C. Trimethylacetyl chloride (1.0 eq, 21.43 mmol, 2.64 ml) was added dropwise over a period of 1 h and the reaction mixture was allowed to stir at ambient temperature for another hour. Water (30.0 ml) was added and the reaction mixture was stirred vigorously for 30 min followed by extraction with ethyl acetate and drying over sodium sulfate. Yield: 3.431 g of **II-a** as colorless oil (99%). TLC (10% MeOH in $CH_2Cl_2$): 0.72. $^1$H-NMR ($CDCl_3$, 400 MHz): δ 1.24 (9 H, s, *t*-Bu); 3.06 (2 H, t, $^3J_{HH}$ = 6.05 Hz, $SCH_2$); 3.74 (2 H, t, $^3J_{HH}$ = 6.04 Hz, $CH_2O$) ppm.

$^1$H-NMR (400 MHz, $CDCl_3$) of compound **II-a**

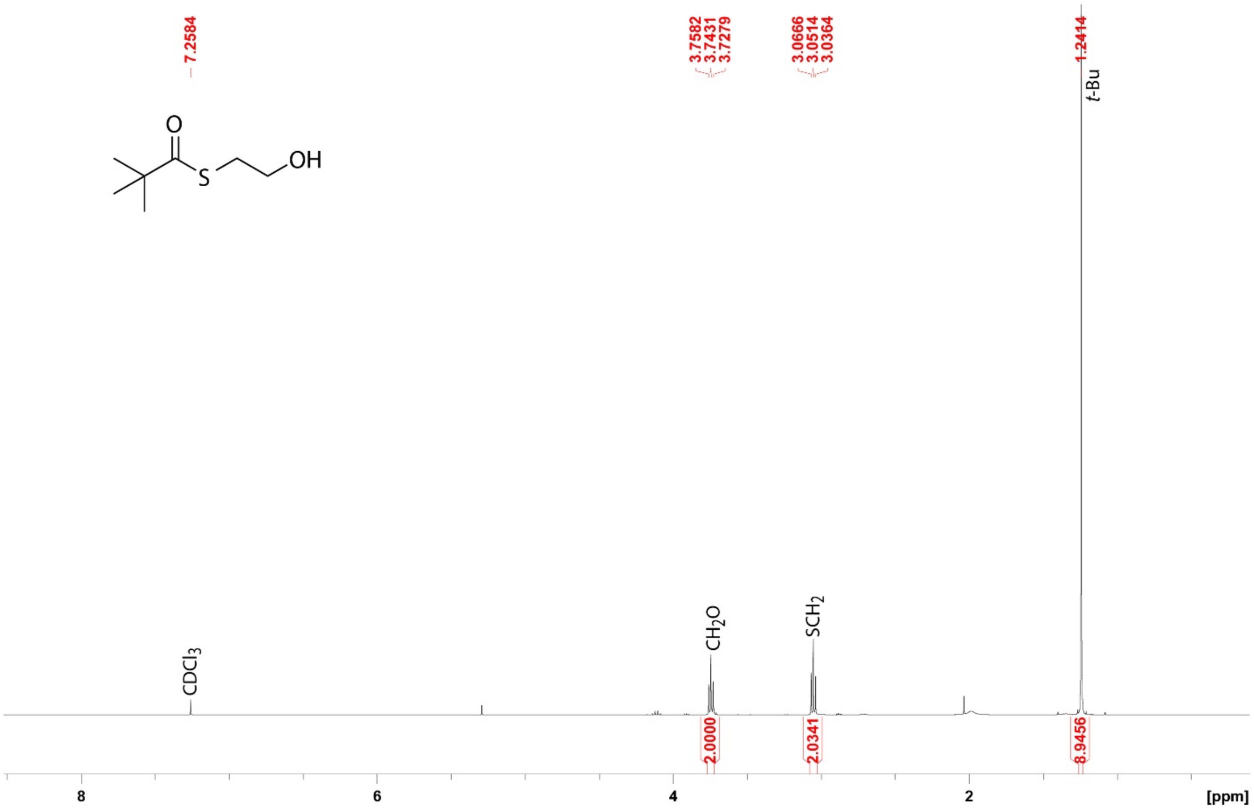

### 6.2.2. Synthesis of bis-(*S*-pivaloyl-2-thioethyl) *N,N*-diisopropylphosphoramidite (II) [20,21]

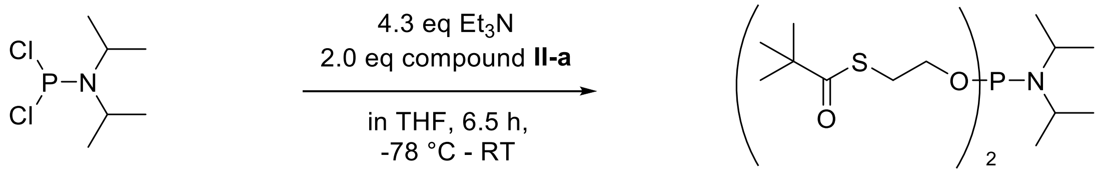


**I-a** **II**

Compound **II-a** (2.0 eq, 14.96 mmol, 2.527 g) and triethylamine (4.3 eq, 32.16 mmol, 4.48 ml) in tetrahydrofuran (20.0 ml) were added dropwise over a period of 1 h to a solution of freshly prepared compound **I-a** (1.541 g, 7.48 mmol) in tetrahydrofuran (20.0 ml) at -78 °C. The cloudy solution was left stirring for two more hours whereby it was allowed to warm to -50 °C. The cooling system was removed and stirring was continued for 3.5 h at ambient temperature. Salts were collected by filtration and the filtrate was concentrated to a thick oil which was purified by column chromatography (ethyl acetate in c-hexane 4/96 + 1% $Et_3N$). Yield: 2.080 g of **II** as colorless oil (60%). TLC (4% ethyl acetate in c-hexanes + 1% $Et_3N$): 0.49. $^1$H-NMR ($d_6$-DMSO, 400 MHz): δ 1.12 (12 H, d, $^3J_{HH}$ = 7.00 Hz, 2x $(CH_3)_2$ (*i*Pr)); 1.18 (18 H, s, 2x *t*-Bu); 3.02 (4 H, t, $^3J_{HH}$ = 6.33 Hz, 2x $SCH_2$); 3.49-3.71 (6 H, m, 2x $CH_2O$, 2x CH (*i*Pr)) ppm. $^{31}$P-NMR ($d_6$-DMSO, 162 MHz): δ 146.84 ppm.

$^1$H-NMR (400 MHz, $d_6$-DMSO) of compound **II**

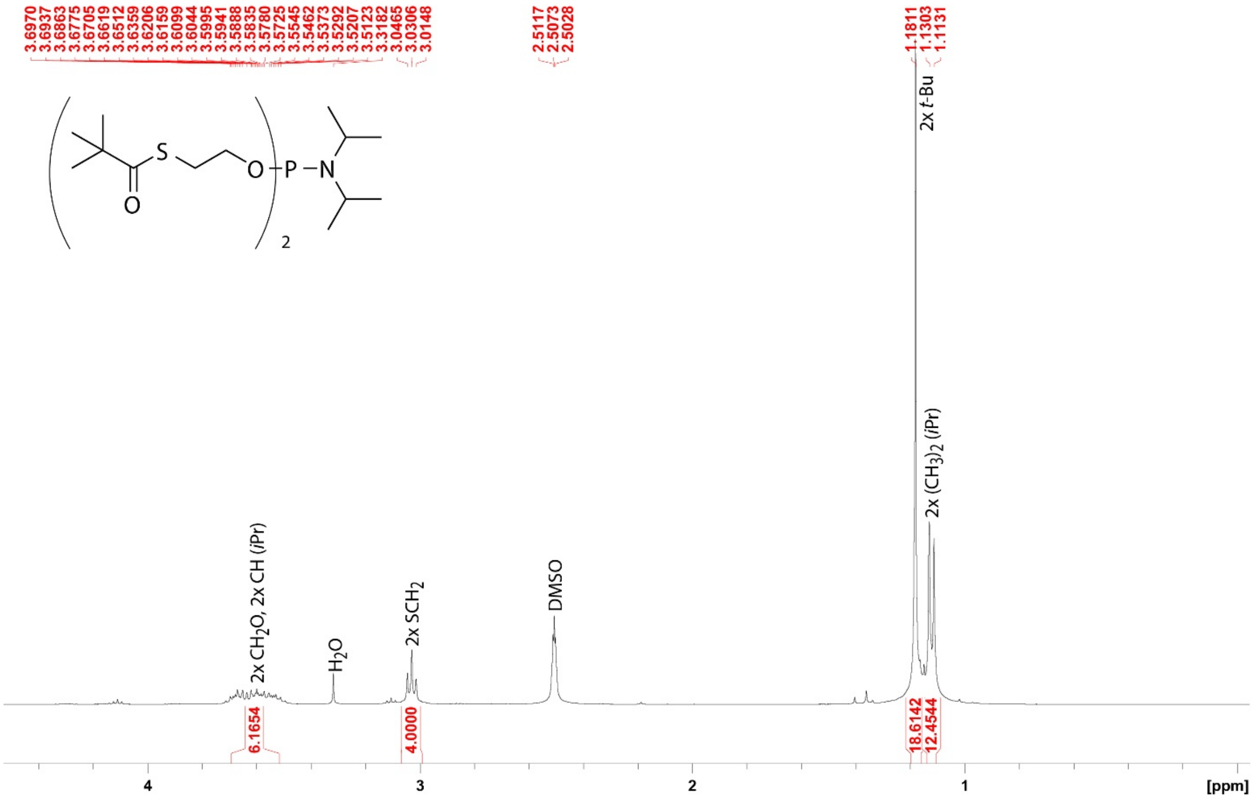

$^{31}$P-NMR (162 MHz, $d_6$-DMSO) of compound **II**

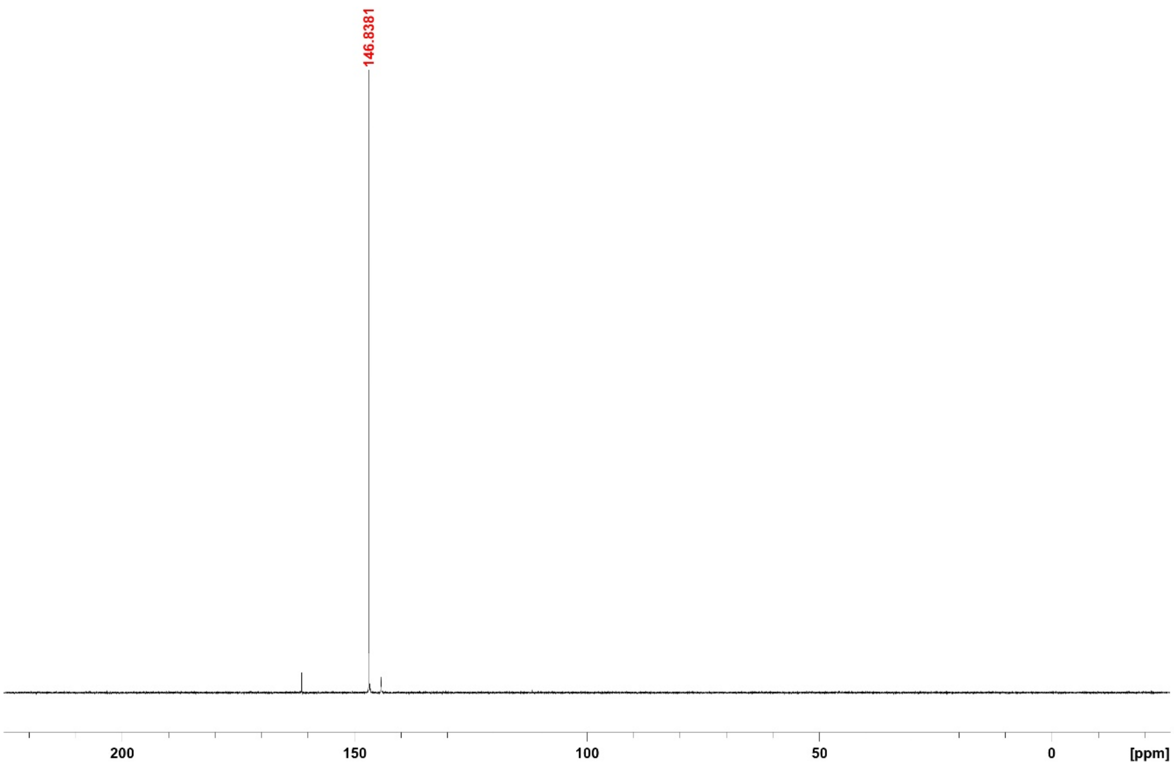

### 6.3. Synthesis of (4-acetoxybenzyl)-((9*H*-fluoren-9-yl)methyl)-*N,N*-diisopropyl phosphoramidite (III)

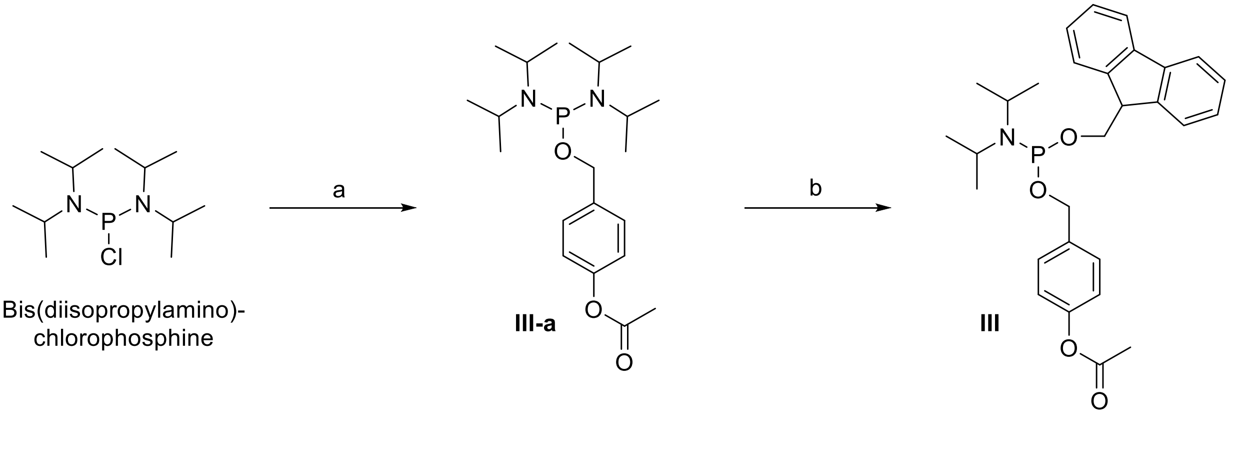


**Overview of the synthesis of (4-acetoxybenzyl)-((9*H*-fluoren-9-yl)methoxy)-*N,N*-diisopropyl-phosphoramidite**. Reaction conditions: **a)** 1.1 eq DIPEA, 1.0 eq compound **I-b**, $CH_2Cl_2$, 6 h, -78°C to room temperature (RT), 46%. **b)** 1.0 eq FmocOH, 1.0 eq 5-(benzylthio)-1*H*-tetrazole, THF, 45 min, RT, 72%. Overall yield: 33%.

#### 6.3.1. Synthesis of 4-acetoxybenzyl bis-(*N,N*-diisopropyl)phosphoramidite (III-a) [22]

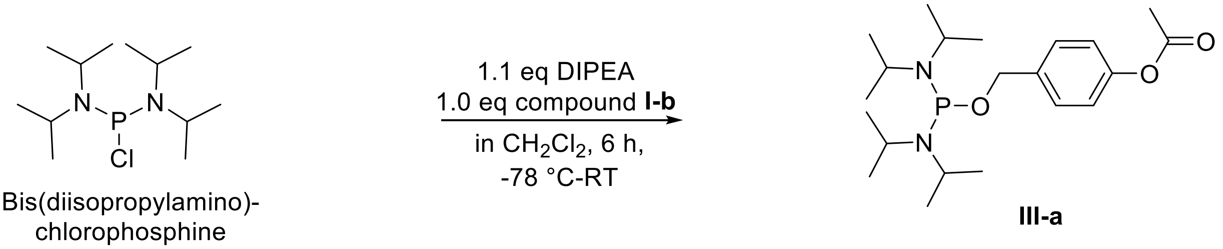


Bis(diisopropylamino)chlorophosphine (1.33 g, 4.99 mmol) was dissolved in dichloromethane (10.0 ml) and cooled to -78 °C. A solution of diisopropylethylamine (1.1 eq, 5.48 mmol, 0.96 ml) and compound **I-b** (1.03 eq, 5.11 mmol, 0.85 g) in dichloromethane (5.00 ml) was added dropwise over 1 h. The solution was allowed to warm to ambient temperature over the course of 4 h and was then stirred for another hour. Solvent was removed and the crude product was purified by column chromatography (20% ethyl acetate in c-hexane + 1% $Et_3N$). Yield: 905 mg of **III-a** as colorless oil (46%). TLC (20% ethyl acetate in c-hexane + 1% $Et_3N$): 0.79. $^1$H-NMR ($CDCl_3$, 400 MHz): δ 1.175 (12 H, d, $^3J_{HH}$ = 6.80 Hz, 2x $(CH_3)_2$ (*i*Pr)); 1.182 (12 H, d, $^3J_{HH}$ = 6.70 Hz, 2x $(CH_3)_2$ (*i*Pr)); 2.29 (3 H, s, $CH_3$ (AB)); 3.52-3.62 (4 H, m, 4x CH (*i*Pr)); 4.63 (1 H, d, $^3J_{HP}$ = 7.24 Hz, $CH_2$ (AB)); 7.04 (1 H, d, $^3J_{HH}$ = 8.52 Hz, aromat. CH); 7.37 (1 H, d, $^3J_{HH}$ = 8.56 Hz, aromat. CH) ppm. $^{31}$P-NMR ($CDCl_3$, 162 MHz): δ 123.43 ppm.

$^{1}H$-NMR (400 MHz, $CDCl_3$) of compound **III-a**

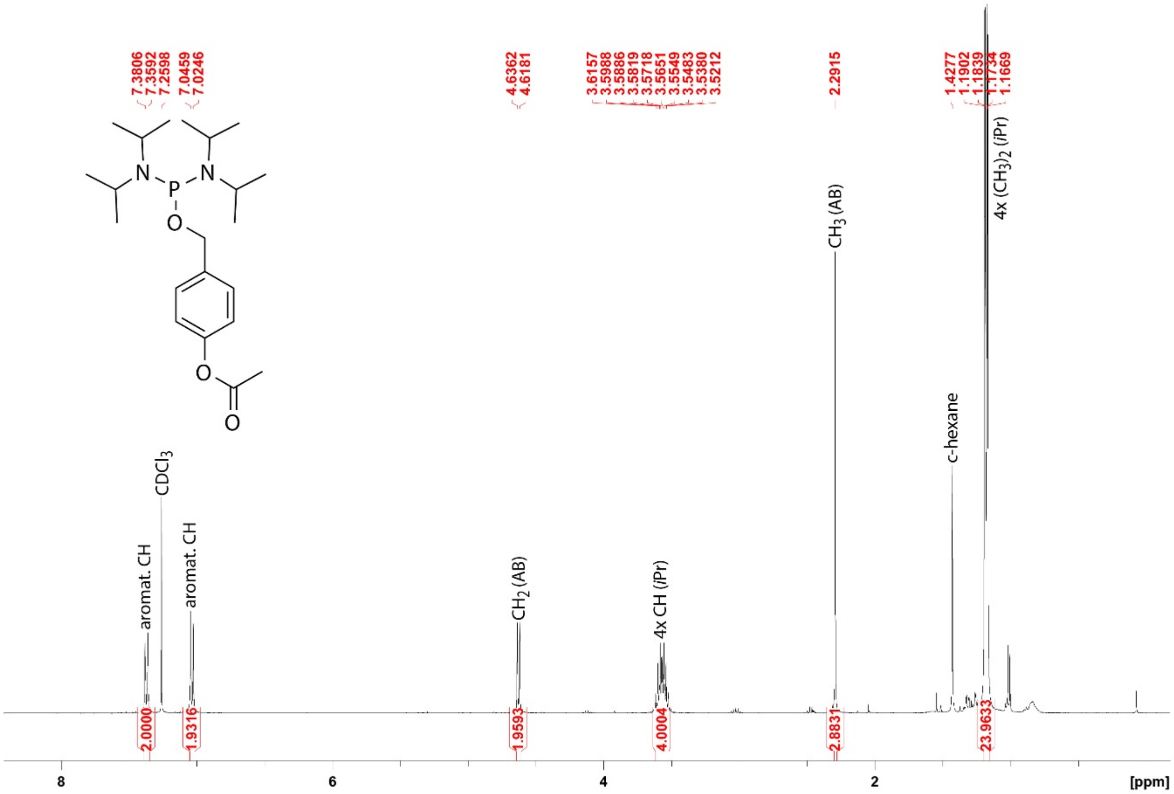


$^{31}P$-NMR (162 MHz, $CDCl_3$) of compound **III-a**

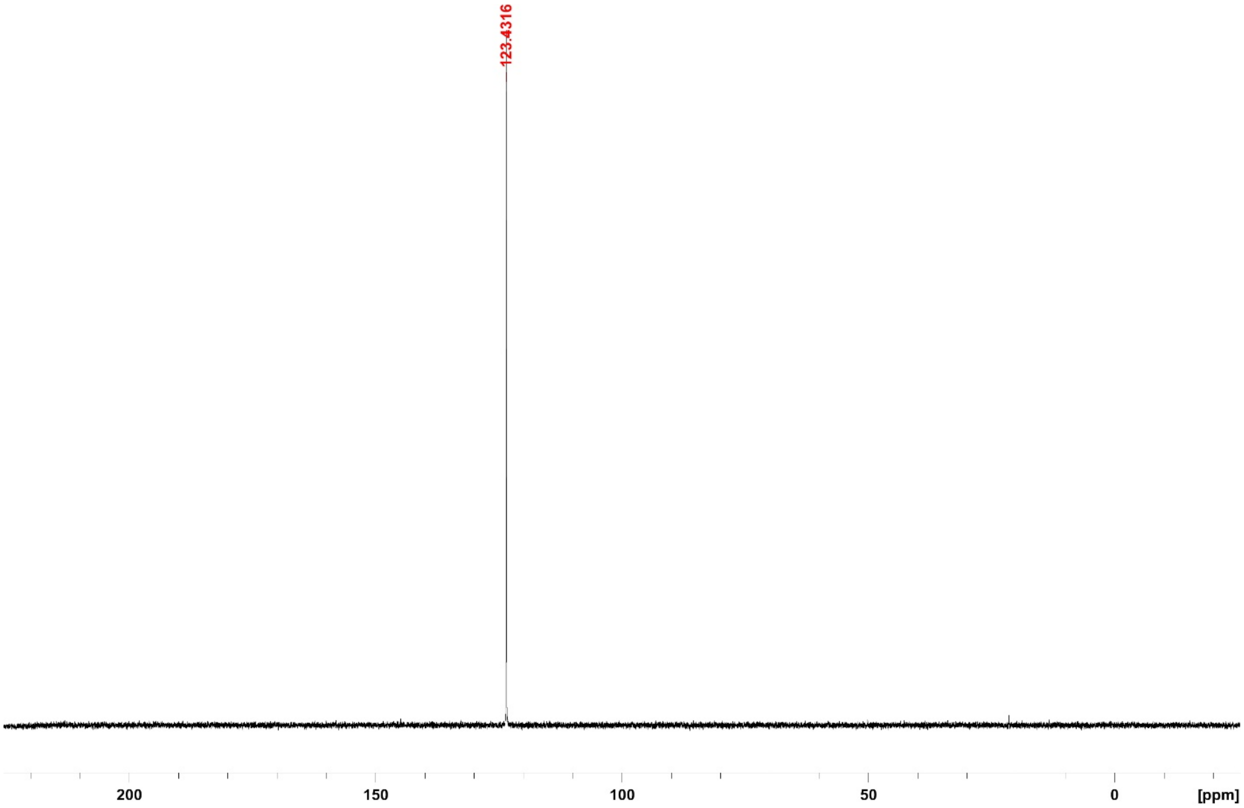

### 6.3.2 Synthesis of (4-acetoxybenzyl)-((9*H*-fluoren-9-yl)methyl)-*N,N*-diisopropylphosphoramidite (III) [5]

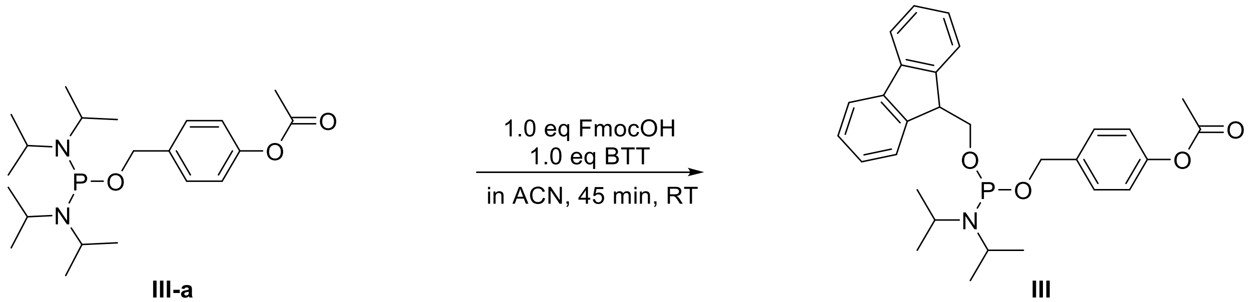


**III-a** **III**

Compound **III-a** (905 mg, 2.28 mmol) and 9-fluorenylmethanol (1.0 eq, 2.28 mmol, 448 mg) were coevaporated trice with acetonitrile and were dried under high vacuum for 5 min. They were then dissolved in acetonitrile containing 0.3 M 5-(benzylthio)-1*H*-tetrazole (7.61 ml) and stirred for 45 min at ambient temperature. The reaction mixture was concentrated and purified by flash column chromatography (30% ethyl acetate in c-hexane + 1% $Et_3N$). Yield: 812 mg of **III** as colorless oil (72%). TLC (30% ethyl acetate in c-hexane + 1% $Et_3N$): 0.76. $^1$H-NMR ($CDCl_3$, 400 MHz): δ 1.18 (6 H, d, $^3J_{HH}$ = 6.79 Hz, $(CH_3)_2$ (*i*Pr)); 1.21 (6 H, d, $^3J_{HH}$ = 6.80 Hz, $(CH_3)_2$ (*i*Pr)); 2.31 (3 H, s, $CH_3$ (AB)); 3.64-3.74 (2 H, m, 2x CH (*i*Pr)); 3.83-3.89 (1 H, m, $CH_a$ (Fmoc); 4.03-4.09 (1 H, m, $CH_b$ (Fmoc); 4.22 (1 H, t, $^3J_{HH}$ = 6.96 Hz, CH (Fmoc)); 4.65 (1 H, dd, $^2J_{HH}$ = 12.67 Hz, $^3J_{HP}$ = 8.38 Hz, $CH_a$ (AB); 4.73 (1 H, dd, $^2J_{HH}$ = 12.70 Hz, $^3J_{HP}$ = 8.24 Hz, $CH_b$ (AB); 7.07 (1 H, d, $^3J_{HH}$ = 8.52 Hz, aromat. CH (AB)); 7.26-7.32, 7.36-7.41 (6 H, m, aromat. CH (AB+Fmoc); 7.63-7.69, 7.75-7.77 (4 H, m, aromat. CH (Fmoc) ppm. $^{31}$P-NMR ($CDCl_3$, 162 MHz): δ 147.20 ppm.

$^{1}$H-NMR (400 MHz, $CDCl_3$) of compound **III**

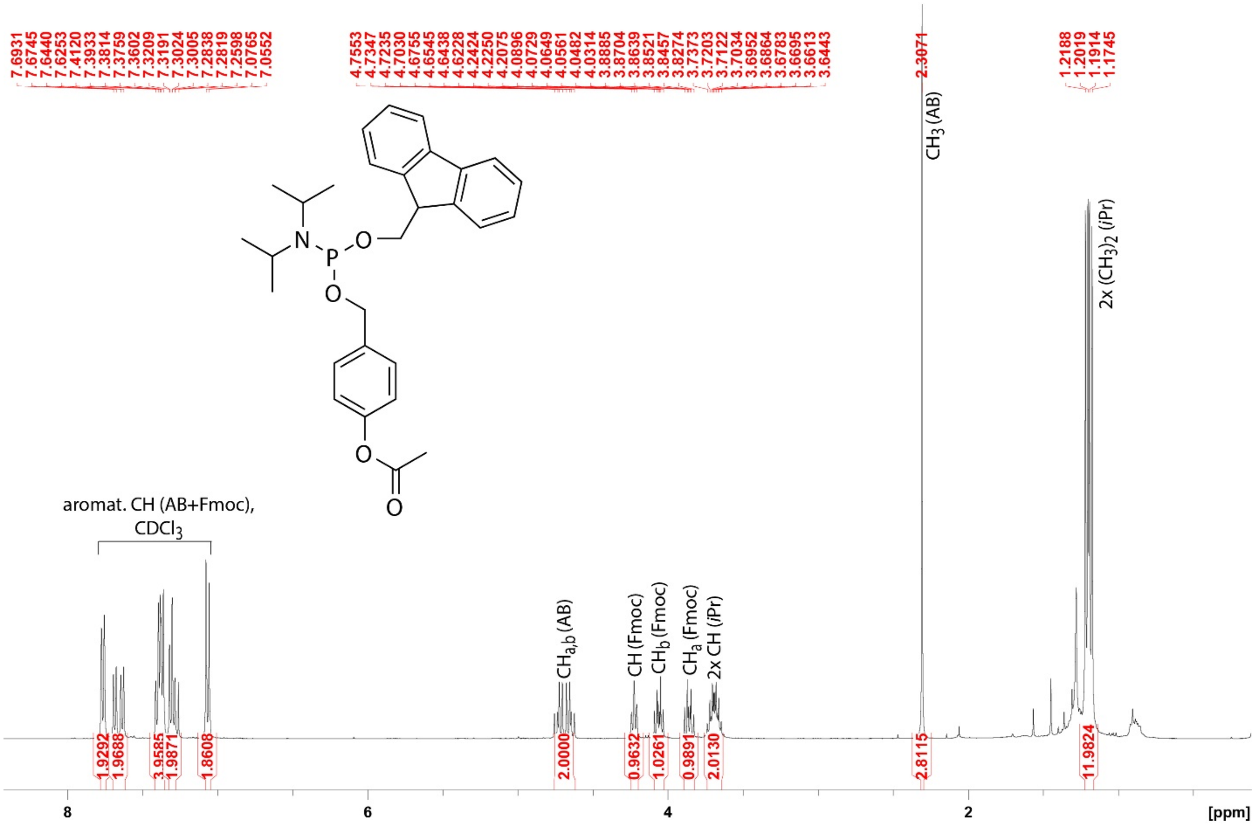


$^{31}$P-NMR (162 MHz, $CDCl_3$) of compound **III**

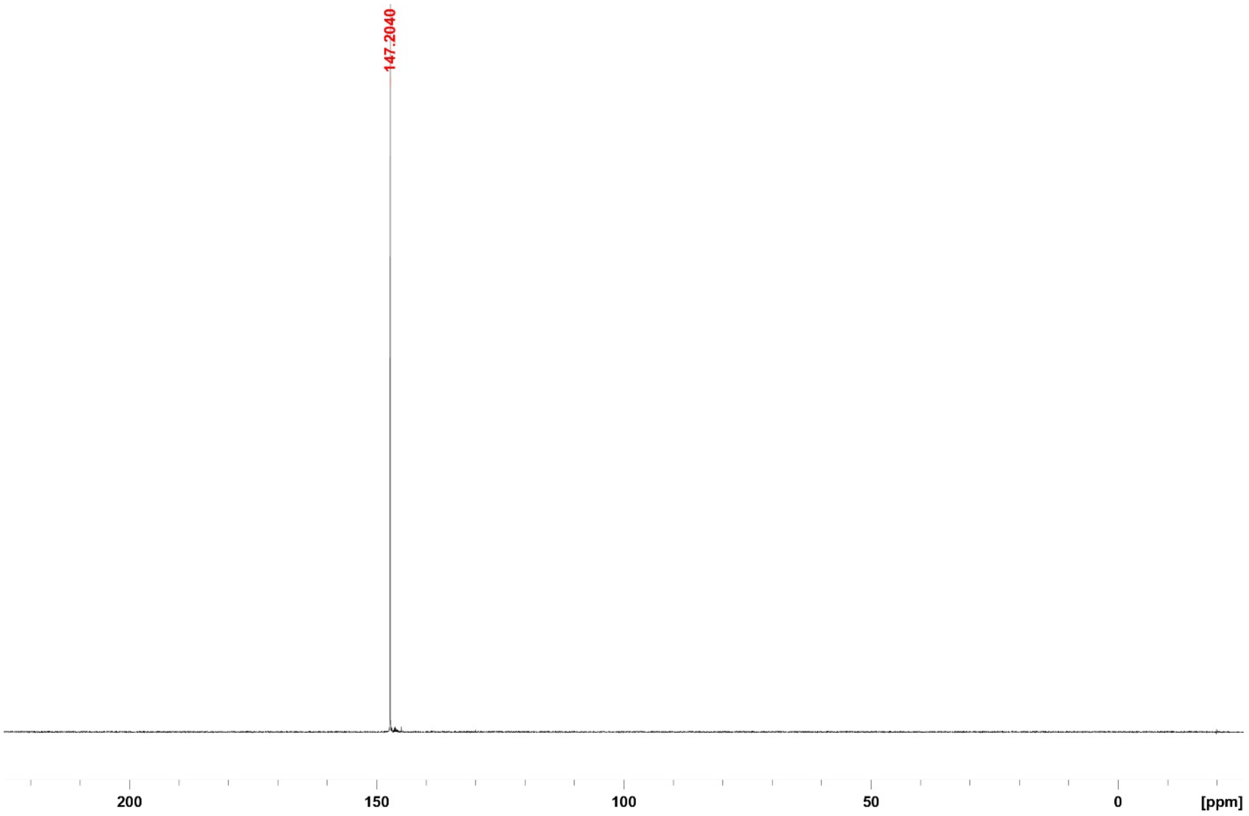

### 6.4. Synthesis of saligenyl-*N,N*-diisopropylphosphoramidite (IV) [23-25]

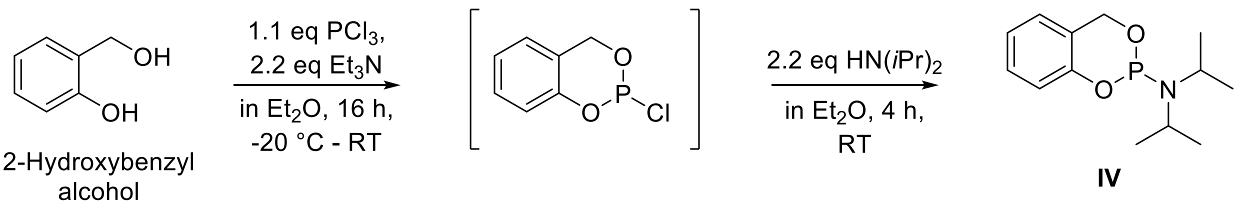


2-Hydroxybenzyl alcohol

**IV**

A solution of 2-hydroxybenzyl alcohol* (1.227 g, 9.88 mmol) in diethyl ether (22.0 ml) was treated with phosphorus trichloride (1.1 eq, 10.87 mmol, 0.95 ml) at -20 °C. After 15 min a solution of diisopropylamine (2.2 eq, 21.74 mmol, 3.03 ml) in diethyl ether (12.0 ml) was added dropwise over a period of 1 h. The resulting slurry was allowed to warm to ambient temperature and stirred for another 3 h. The flask was sealed** and stored at 0 °C overnight (16 h) for the best possible precipitation of triethylammonium chloride. Filtration under argon*** and evaporation of the solvent delivers salicylchlorophosphane (1.195 g, 6.34 mmol) as colorless oil.

The latter was dissolved in diethyl ether (28.0 ml) and diisopropylamine (2.2 eq, 13.94 mmol, 1.94 ml) was introduced dropwise over 30 min. The reaction mixture was stirred 3.5 h at ambient temperature, was filtered, evaporated and purified via flash chromatography (10% HN(*i*Pr)$_2$ in cyclohexane). Yield: 1.018 g of **IV** as white crystals (41%). TLC (HN(*i*Pr)$_2$ /c-hexane 1/9): 0.32. $^{1}$H-NMR ($CDCl_3$, 400 MHz): δ 1.23-1.26 (12 H, 2x d, 2x ($CH_3$)$_2$ (*i*Pr)); 3-57-3.66 (2 H, m, 2x CH (*i*Pr)); 3-59-3.68 (2 H, m, 2x CH (*i*Pr)); 4.87 (1 H, dd, $^{3}J_{HP}$ = 19.85 Hz, $^{2}J_{HH}$ = 14.20 Hz, $CH_a$); 5.14 (1 H, dd, $^{2}J_{HH}$ = 14.20 Hz, $^{3}J_{HP}$ = 5.08 Hz, aromat. $CH_b$); 6.90-6.98 (3 H, m, aromat. CH); 7.18 (1 H, txd, $^{3}J_{HH}$ = 8.08 Hz, $^{3}J_{HH}$ = 1.60 Hz, aromat. CH) ppm. $^{31}$P-NMR ($CDCl_3$, 162 MHz): δ 135.78 ppm.

*From salicylic acid by reduction using $LiAlH_4$ [26] .Starting material was dried under high vacuum overnight

**Parafilm over septum

***Schlenk-frit and flask were dried at 110 °C overnight and were subsequently cooled to room temperature under high vacuum. All glassware was flushed with argon before filtration was executed. Filtration of the thick paste turned out to be challenging since plenty of material remained in the reaction flask. Due to the instability of the intermediate compound, no additional ether was used for complete transfer of the chlorophosphane. Take care of constant pressure equilibration

$^{1}$H-NMR (400 MHz, $CDCl_3$) of compound **IV**

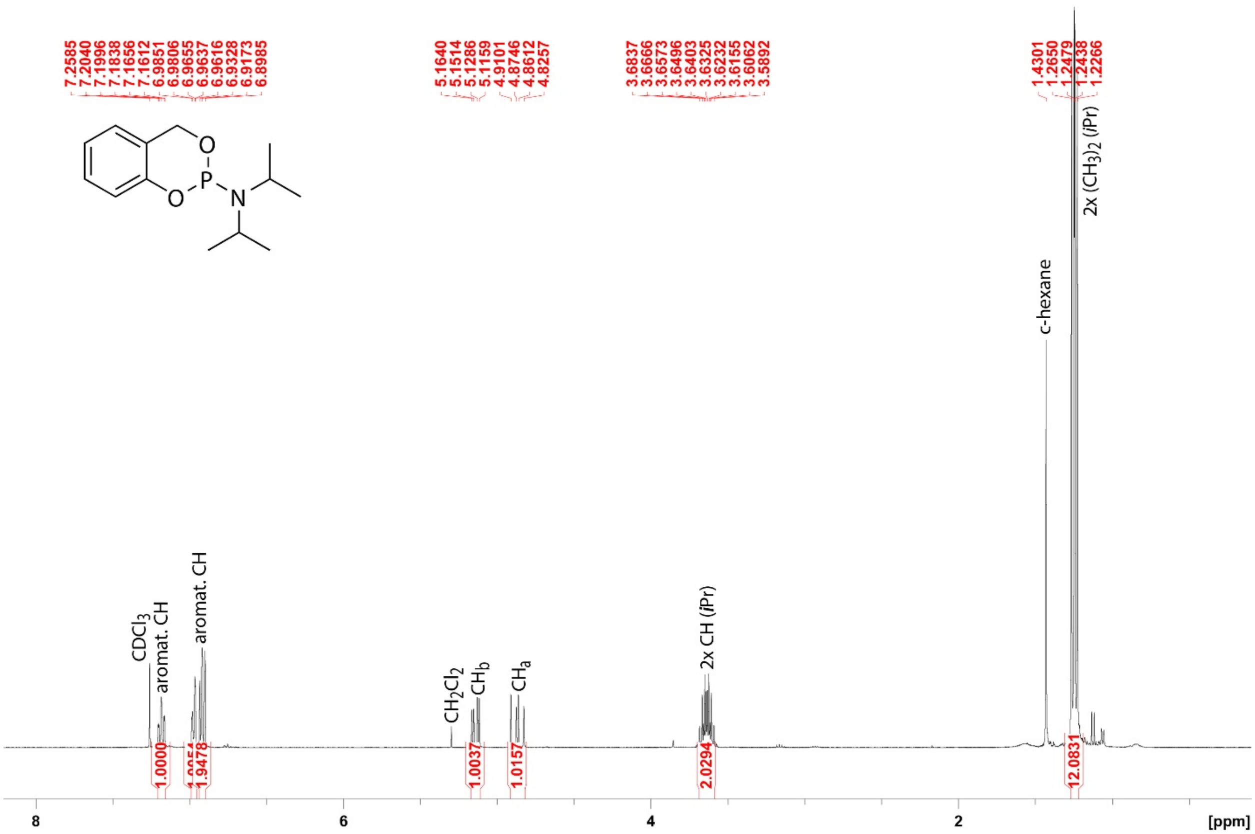


$^{31}$P-NMR (162 MHz, $CDCl_3$) of compound **IV**

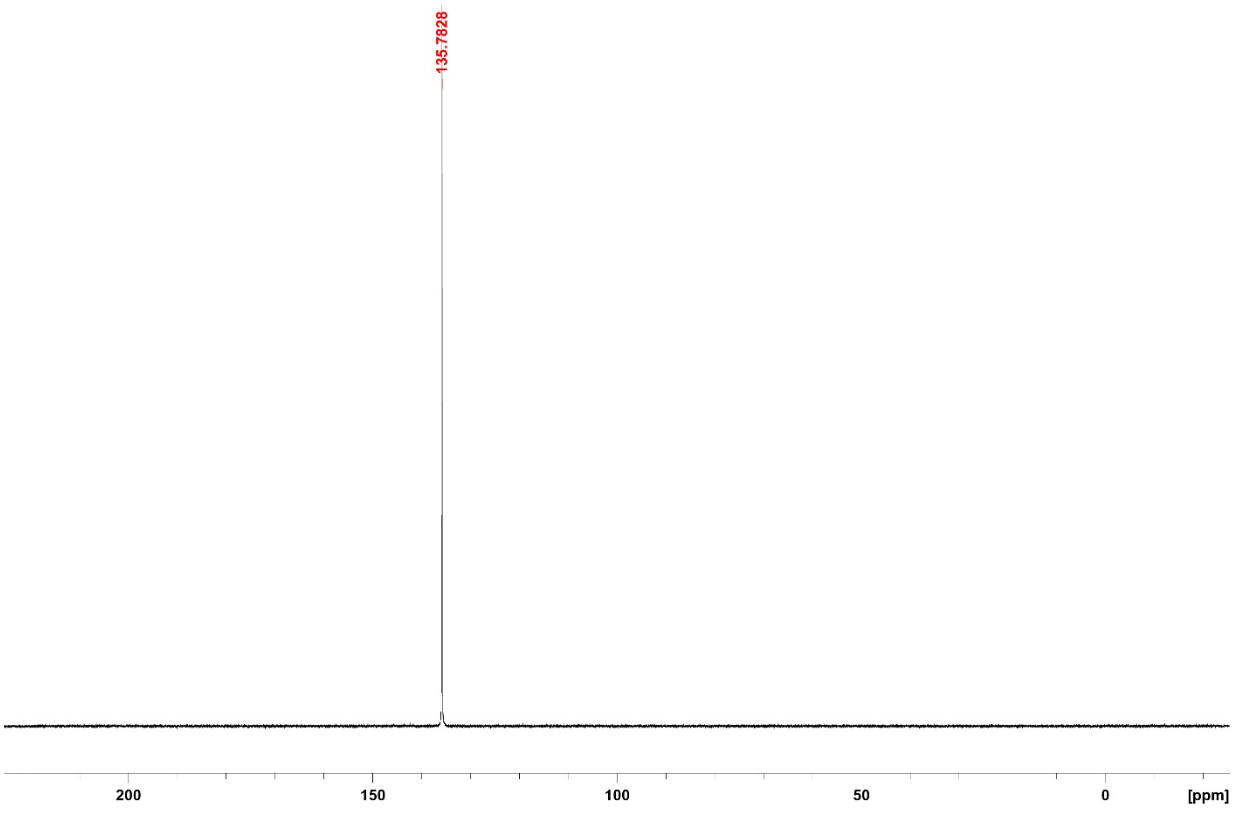

## 6.5. Synthesis of 5-chlorosaligenyl-*N,N*-diisopropylphosphoramidite (V) [24,25,27]

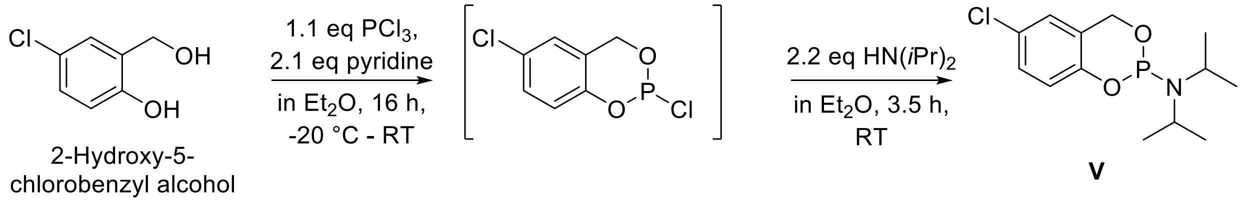


2-Hydroxy-5-chlorobenzyl alcohol

**V**

Phosphorous trichloride (1.1 eq, 5.87 mmol, 0.51 ml) was added slowly to a solution of 2-hydroxy-5-chlorobenzyl alcohol* (846 mg, 5.33 mmol) in diethyl ether (12.0 ml) at -20 °C. The clear solution was stirred 15 min at -20 °C whereupon a solution of pyridine (2.1 eq, 11.20 mmol, 0.91 ml) in diethyl ether (7.5 ml) was added dropwise over 30 min. The solution was slowly warmed to ambient temperature (1.5 h) and was then stirred for further 2.5 h. The flask was sealed** and stored at 0 °C overnight (16 h) for precipitation of pyridinium chloride. Filtration under argon*** and evaporation of the solvent delivers 5-chlorosalicylchlorophosphane (996 mg, 84%) as slightly yellow oil.

The latter was dissolved in diethyl ether (20.0 ml) and diisopropylamine (2.2 eq, 9.85 mmol, 1.38 ml) was added dropwise. The reaction mixture was stirred 3.5 h at ambient temperature, was filtered, evaporated and purified via flash chromatography (10% HN(*i*Pr)$_2$ in cyclohexane). Compound **V** crystallized upon freezing. <u>Yield:</u> 795 mg of **V** as white crystals (52%). <u>TLC</u> (HN(*i*Pr)$_2$ /*c*-hexane 1/9): 0.57. <u>$^1$H-NMR</u> ($CD_3CN$, 400 MHz): δ 1.19-1.22 (12 H, 2x d, 2x $(CH_3)_2$ (*i*Pr)); 3-57-3.66 (2 H, m, 2x CH (*i*Pr)); 4.84 (1 H, ddd, $^3J_{HP}$ = 19.49 Hz, $^2J_{HH}$ = 14.92 Hz, $CH_a$, $^4J_{HH}$ = 0.75 Hz); 5.06 (1 H, ddd, $^2J_{HH}$ = 14.63 Hz, $^3J_{HP}$ = 5.38 Hz, aromat. $CH_b$, $^4J_{HH}$ = 0.91 Hz); 6.85 (1 H, d, $^3J_{HH}$ = 8.28 Hz, aromat. CH); 7.07 (1 H, d, $^4J_{HH}$ = 2.56 Hz, aromat. CH); 7.19 (1 H, ddd, $^3J_{HH}$ = 8.64 Hz, $^4J_{HH}$ = 2.60 Hz, aromat. CH, $^4J_{HH}$ = 0.89 Hz) ppm. <u>$^{31}$P-NMR</u> ($CD_3CN$, 162 MHz): δ 136.23 ppm.

*From the corresponding salicylic acid by reduction using $LiAlH_4$ [28]. Starting material was dried under high vacuum overnight

**Parafilm over septum

*** Schlenk-frit and flask were dried at 110 °C overnight and were subsequently cooled to room temperature under high vacuum. All glassware was flushed with argon before filtration was executed. Filtration of the thick paste turned out to be challenging since plenty of material remained in the reaction flask. Due to the instability of the intermediate compound, no additional ether was used for complete transfer of the chlorophosphane. Take care of permanent pressure equilibration

$^{1}$H-NMR (400 MHz, $CD_3CN$) of compound **V**

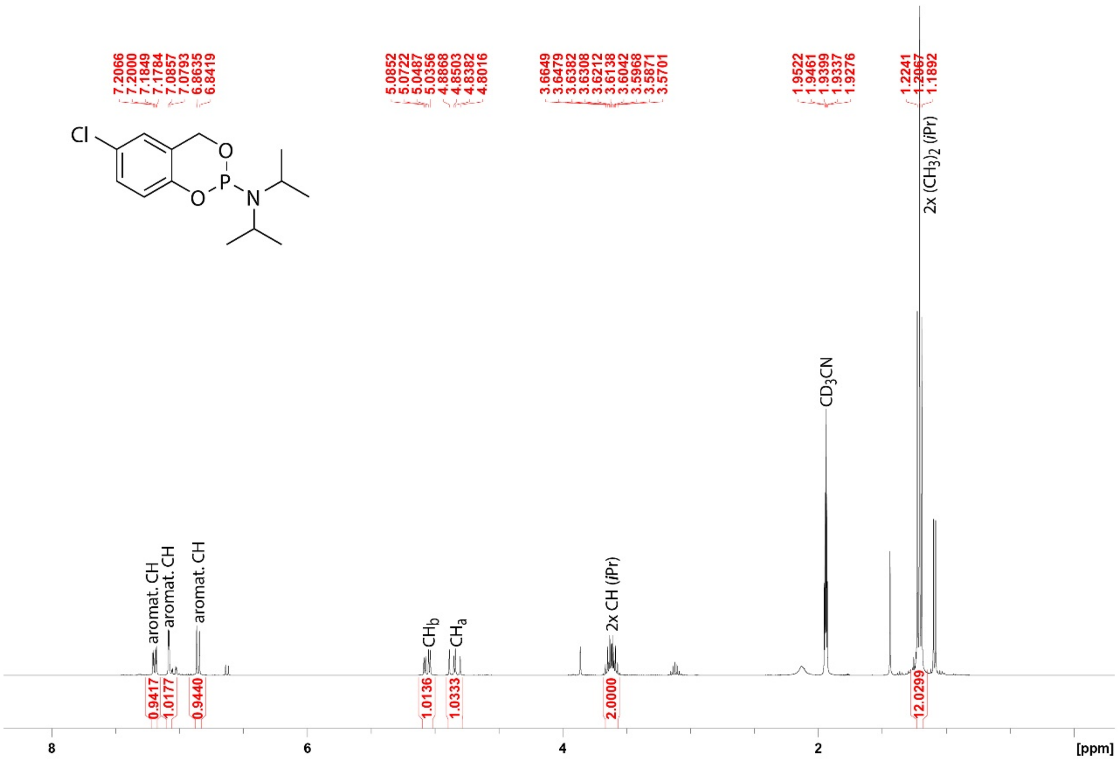


$^{31}$P-NMR (162 MHz, $CD_3CN$) of compound **V**

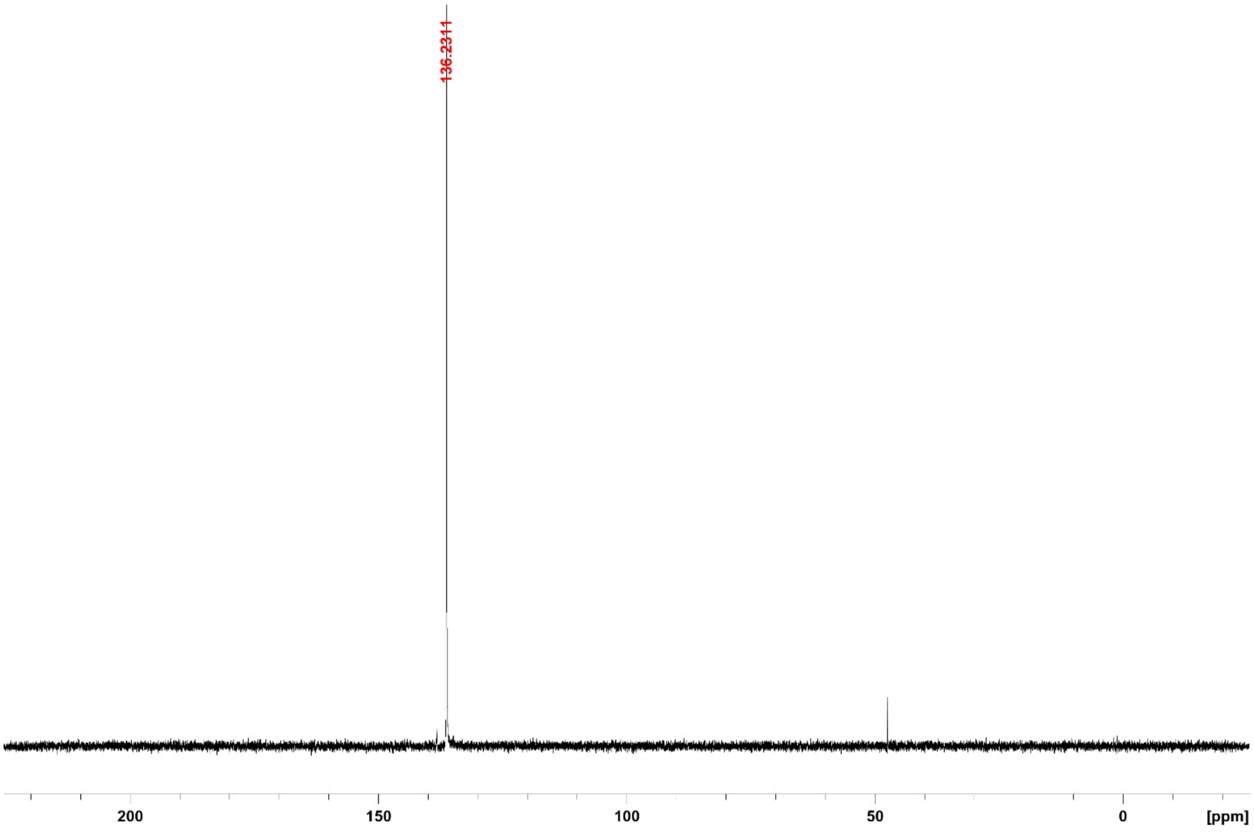

## 6.6. Synthesis of 2-ethylbutyl (*S*)-2-aminopropanoate hydrochloride (VI) [29]

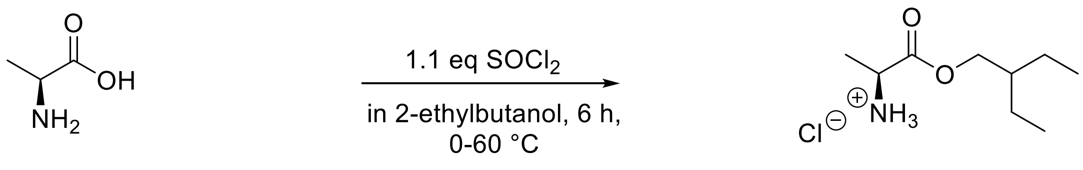


L-alanine

**VI**

Thionyl chloride (1.1 eq, 12.34 mmol, 1.440 g) was added dropwise to an ice-cooled solution of L-alanine (1.000 g, 11.22 mmol) in 2-ethylbutanol (10.0 ml). The reaction mixture was heated to 60 °C and stirred for 6 h. Solvent was removed by high vacuum distillation (60 °C) and the remaining residue was treated with diethyl ether whereby the product precipitates. Yield: 1.750 g of **VI** as white solid (90%). $^{1}$H-NMR ($d_6$-DMSO, 400 MHz): δ 0.86 (6 H, t, $^{3}J_{HH}$ = 7.42 Hz, 2x $CH_3$ (ester)); 1.33 (4 H, m, 2x $CH_2$ (ester)); 1.42 (3 H, d, $^{3}J_{HH}$ = 7.42 Hz, $CH_3$ (Ala)); 1.52 (1 H, m, CH (ester)); 4.08 (3 H, m, CH (Ala); $OCH_2$ (ester)); 8.55 (3 H, s br., $NH_3$) ppm.

$^{1}$H-NMR (400 MHz, $d_6$-DMSO) of compound **VI**

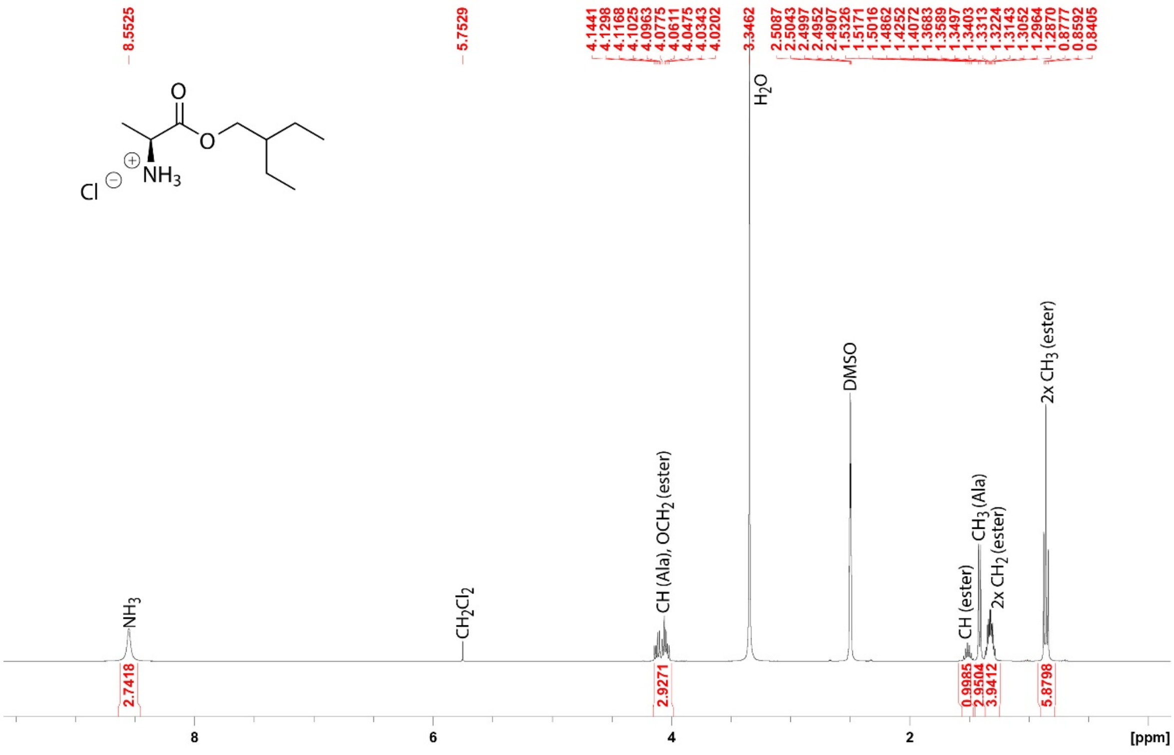

**Supporting Scheme S1.** Synthesis of 4-thiouridine. [a]

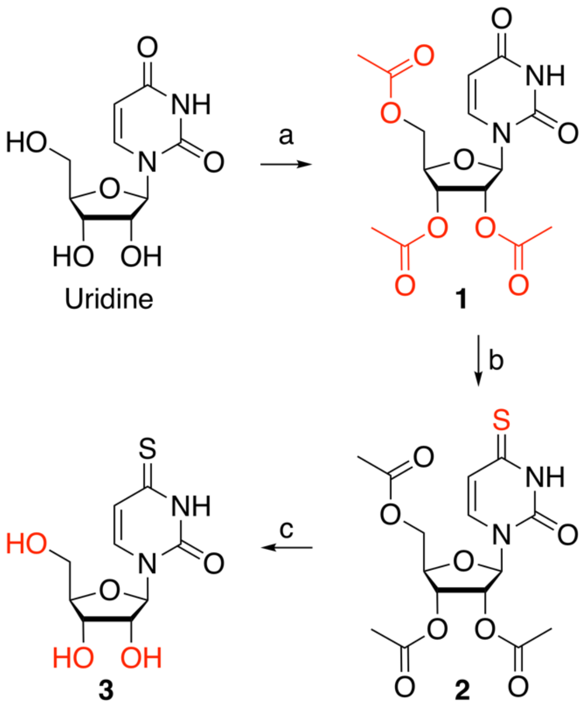


[a] Reaction conditions: **a)** 4.0 eq acetic anhydride, 0.1 eq 4-(dimethylamino)pyridine, 4.0 eq $Et_3N$, acetonitrile, 16 h room temperature (RT), 99%. **b)** 1.5 eq Lawesson reagent, toluene, 2.5 h, 100 °C, 98%. **c)** 7 N $NH_3$ in MeOH, 16 h, RT, 71%. Overall yield: 69%.

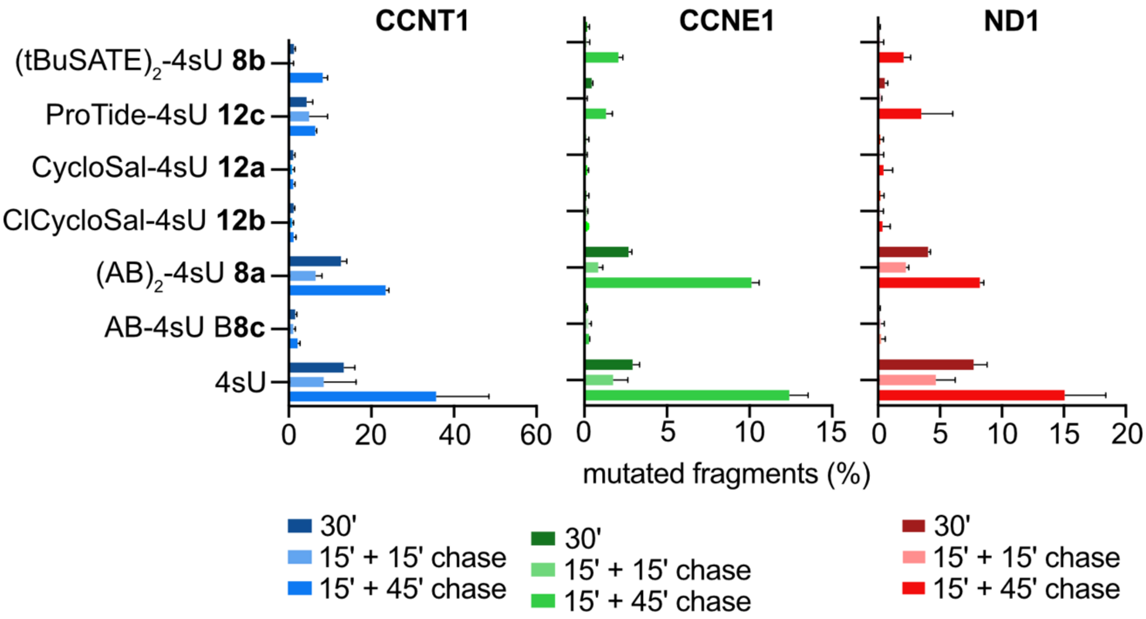


**Supporting Figure S1.** Comparison of mRNA labeling efficiencies using different 4sU prodrugs. HEK293T cells were labeled with 50 µM of the indicated 4sU prodrugs for either 15 min with a subsequent chase period of 15 or 45 min followed by RNA extraction, or for 30 min with subsequent RNA extraction. RNA was treated by TUC-seq chemistry and subjected to amplicon sequencing of the nuclear targets *CCNT1* and *CCNE1* and the mitochondrial transcript ND1. Labeling efficiency was calculated by determining the fraction of reads containing ≥1 U-to-C mutation and subtracting background mutation frequency (vehicle control) from the resulting values. Mean ± SD is shown (N=2).

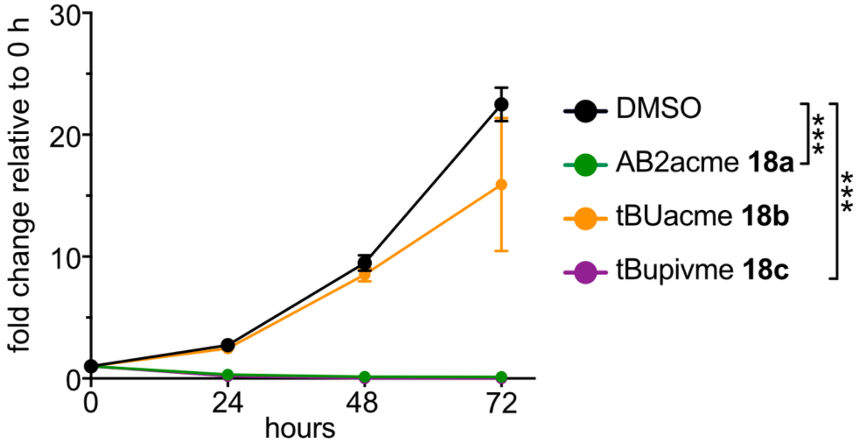


**Supporting Figure S2**. *S*4-protected 4sU prodrugs cause strong cytotoxicity. HEK293T cells were labeled with 50 µM of the indicated prodrugs for 30 min followed by incubation in normal media. Cell viability was determined by ATP measurement at 24, 48 and 72 h. Values were normalized to the DMSO-treated control and mean values ± SD are shown (n=3). Statistical significance was calculated by one-way-ANOVA (* p<0.05, ****p<0.0001). Note that *t*Bupivme-treated cells did not survive beyond 24 h.

**Supporting Table S1**. Sequences of primers used for amplicon generation (barcodes not shown).

| mRNA target | Forward primer | Reverse primer |
|---|---|---|
| *CCNE1* | 5'-AAGATGCACACAACATAC | 5'-CACTGGTGTCTGGAGGTG |
| *CCNT1* | 5'-CTGGCTTAAGTACCCAAAG | 5'-GCATCCAGAGCTGAGGTG |
| *ND1* | 5'-ACAACTACGCAAAGGCCC | 5'-TGATGGCTAGGGTGACTT |

## 8. References


[1] a. Bavaro, T.; Rocchietti, S.; Ubiali, D.; Filice, M.; Terreni, M.; Pregnolato, M. A Versatile Synthesis of 5′-Functionalized Nucleosides Through Regioselective Enzymatic Hydrolysis of Their Peracetylated Precursors. *Eur. J. Org. Chem*. 12 (**2009**): 1967-1975; b. El-Tayeb, A.; Qi, A.; Nicholas, R. A.; Mueller, C. E. *J. Med. Chem.*, 54 (**2011**): 2878–2890.

[2] a. Gupte, A.; Buolamwini, J.K. Novel C2-purine position analogs of nitrobenzylmercaptopurine riboside as human equilibrative nucleoside transporter 1 inhibitors. *Bioorg. Med. Chem.* 15 (**2007**): 7726-7737; b. El-Tayeb, A.; Qi, A.; Nicholas, R. A.; Mueller, C. E. *J. Med. Chem.*, 54 (**2011**): 2878–2890.

[3] Zhang, X.; Wang, J.; Xu, Y.Z. Systematic assignment of NMR spectra of 5-substituted-4-thiopyrimidine nucleosides. *Magn. Res. Chem.* 51 (**2013**): 523-529; b. Fox, J. J. .; Praag, D. V.; Wempen, I.; Doerr, I. L.; Cheong, L.; Knoll, J. E.; Eidinoff, M. L.; Bendich, A.; Brown, G. B. *J. Am. Chem. Soc*. (**1959**): 81, 178-187; c. El-Tayeb, A.; Qi, A.; Nicholas, R. A.; Mueller, C. E. *J. Med. Chem.*, 54 (**2011**): 2878–2890.

[4] Becker, H.F.; Grosjean, H.; Fourrey, J.-L. Chemical synthesis of 4-thiouridine-containing substrate analogues of tRNA: pseudouridine-55 synthase: Photocross-linking studies. *Nucl. Nucl.* 17 (**1998**): 2403-2416.

[5] Pavlovic, I.; Thakor, D.T.; Bigler, L.; Wilson, M.S.C.; Laha, D.; Schaaf, G.; Saiardi, A.; Jessen, H.J. Prometabolites of 5-Diphospho-myo-inositol Pentakisphosphate. *Angew. Chem. Int. Ed.* 54 (**2015**): 9622-9626.

[6] Hauke, S.; Dutta, A. K.; Eisenbeis, V.B.; Bezold, D.; Bittner, T.; Wittwer, C.; Thakor, D.; Pavlovic, I.; Schultz, C.; Jessen, H.J.. Photolysis of cell-permeant caged inositol pyrophosphates controls oscillations of cytosolic calcium in a β-cell line. *Chem. Sci.* 10 (**2019**): 2687-2692.

[7] Inde, T.; Nishizawa, S.; Hattori, Y.; Kanamori ,T.; Yuasa, H.; Seio, K.; Sekine, M.; Ohkubo, A. Synthesis of and triplex formation in oligonucleotides containing 2′-deoxy-6-thioxanthosine. *Bioorg. Med. Chem.* 26 (**2018**): 3785-3790.

[8] Macchione, G.; Maza, S.; Mar Kayser, M.; de Paz, J.L.; Carrera; Nieto, P.M.. Synthesis of chondroitin sulfate oligosaccharides using *N*-(tetrachlorophthaloyl)-and *N*-(trifluoroacetyl) galactosamine building blocks. *Eur. J. Org. Chem*. 18 (**2014**): 3868-3884.

[9] Shen, W.; Kim, J.S.; Kish, P.E.; Zhang,J.; Mitchell,S.; Gentry, B.G.; Breitenbach, J.M.; Drach, J.C.; Hilfinger, J. Design and synthesis of vidarabine prodrugs as antiviral agents. *Bioorg. Med. Chem. Lett.* 19 (**2009**): 792-796.

[10] Liotta, D.C.; Paintner, G.R.; Bluemling, G.R.; de La Rosa, A. Nucleotide and nucleoside therapeutic compositions and uses related thereto (2015). Patent US20160220595A1.

[11] Meier, C.; Lomp, A.; Meerbach, A.; Wutzler, P. Cyclo Sal-BVDUMP Pronucleotides: How to Convert an Antiviral-Inactive Nucleoside Analogue into a Bioactive Compound against EBV. *J. Med. Chem.* 45 (**2002**): 5157-5172.

[12] Wei, S.; Kim, J.-S.; Hilfinger, J. Expedient total synthesis of triciribine and its prodrugs. *Synth. Commun.* 42 (**2012**): 358-374.

[13] Riml, C. Ph.D. thesis, Leopold-Franzens University, Innsbruck, 2017.

[14] Riml, C.; Amort, T.; Rieder, D.; Gasser, C.; Lusser, A.; Micura, R. Osmium-mediated transformation of 4-thiouridine to cytidine as key to study RNA dynamics by sequencing. *Angew. Chem. Int. Ed.* 56 (**2017**): 13479-13483.

[15] Jin, X.; Huang, H.; Vogel, K.W.; Drueckhammer, D.G. The importance of the amide bond nearest the thiol group in enzymatic reactions of coenzyme A. *Bioorg. Chem.* 33 (**2005**): 90-107.

[16] Wiesler, W.T.; Caruthers, M.H. Synthesis of Phosphorodithioate DNA via Sulfur-Linked, Base-Labile Protecting Groups 1. *J. Org. Chem.* 61 (**1996**): 4272-4281.

[17] Paintner, G.R.; Guthrie, D.B.; Bluemling, G.R.; Natchus, M.G. N4-Hydroxycytidine and derivatives and anti-viral uses related thereto. PCT/US2015/066144, WO 2016/106050 Al Patent

[18] Nurminen, E.J.; Mattinen, J.K.; Lönnberg, H. Kinetics and mechanism of tetrazole-catalyzed phosphoramidite alcoholysis. *J. Chem. Soc., Perkin Trans 2*, **7** (**1998**): 1621-1628.

[19] Jessen, H.J.; Schulz, T.; Balzarini, J.; Meier, C. Bioreversible protection of nucleoside diphosphates. *Angew. Chem. Int. Ed.* 47 (**2008**): 8719-8722.

[20] Kuniyoshi, H.; Nakagawa, D.; Matsumoto, T.; Yoshimitsu, Y. Thionucleoside derivative or salt thereof, and pharmaceutical composition. CA2966138C, US 2017 / 0233429 A1 Patent

[21] Lefebvre, I.; Perigaud, C.; Pompon, A.; Aubertin, A.-M.; Girardet, J.-L.; Kirn, A.; Gosselin, G.; Imbach, J.-L. Mononucleoside phosphotriester derivatives with *S*-acyl-2-thioethyl bioreversible phosphate-protecting groups: intracellular delivery of 3'-azido-2',3'-dideoxythymidine 5'-monophosphate. *J. Med. Chem.* 38 (**1995**): 3941-3950.

[22] Routledge, A.; Walker, I.; Freeman,S.; Hay, A.; Mahmood, N. Synthesis, bioactivation and anti-HIV activity of 4-acyloxybenzyl bis (nucleosid-5′-yl)phosphates. *Nucl. Nucl. Nucl. Acids* 14, (**1995**): 1545-1558.

[23] Ducho, C.; Görbig, U.; Jessel, S.; Gisch, N.; Balzarini, J.; Meier, C. Bis-cyclo sal-d4t-monophosphates: Drugs that deliver two molecules of bioactive nucleotides. *J. Med. Chem.* 50, (**2007**): 1335-1346.

[24] Wissner, A.; Carroll, M.L.; Green, K.E.; Kerwar, S.S.; Pickett, W.C.; Schaub, R.E.; Torley, L.W.; Wrenn, S.; Kohler, C.A.. Analogs of platelet activating factor. 6. Mono-and bis-aryl phosphate antagonists of platelet activating factor. *J. Med. Chem.* 35 (**1992**): 1650-1662.

[25] Meier, C.; De Clercq, E.; Balzarini, J. Nucleotide Delivery from cycloSaligenyl-3′-azido-3′-deoxythymidine Monophosphates (cycloSal-AZTMP). *Eur. J. Org. Chem.* 5 (**1998**): 837-846.

[26] Bergner, M.; Duquette, D.C.; Chio, L.; Stoltz, B.M. Exceedingly Efficient Synthesis of (±)-Grandifloracin and Acylated Analogues. *Org. Lett.* 17, (**2015**): 3008-3010.

[27] Sarac, I.; Meier, C. Efficient automated solid-phase synthesis of DNA and RNA 5′-triphosphates. *Chem. Eur. J.* 21 (**2015**): 16421-16426.

[28] Kemp, D.S.; Carey, R.I.; Dewan, J.C.; Galakatos, N.G.; Kerkman, D.; Leung, S.L. Rational design of templates for intramolecular *O*,*N*-acyl transfer via medium-sized cyclic intermediates derived from L-cysteine. Definition of an experimental maximum in effective molarity through the study of tunable templates. *J. Org. Chem.* 54 (**1989**): 1589-1603.

[29] Wang, M.; Zhang, L.; Huo, X.; Zhang, Z.; Yuan, Q.; Li, P.; Chen, J.; Zou, Y.; Wu, Z.; Zhang, W. Catalytic Asymmetric Synthesis of the anti-COVID-19 Drug Remdesivir. *Angew. Chem. Int. Ed*. 59 (**2020**): 20814-20819.
